# Streamlining the use of BOLD specimen data to record species distributions: a case study with ten Nearctic species of Microgastrinae (Hymenoptera: Braconidae)

**DOI:** 10.3897/BDJ.2.e4153

**Published:** 2014-10-29

**Authors:** Jose L Fernandez-Triana, Lyubomir Penev, Sujeevan Ratnasingham, M. Alex Smith, Jayme Sones, Angela Telfer, Jeremy R. deWaard, Paul D. N. Hebert

**Affiliations:** † Canadian National Collection of Insects, Ottawa, and the Biodiversity Institute of Ontario, University of Guelph, Ottawa, Canada; ‡ Pensoft, Sofia, Bulgaria; § University of Guelph, Guelph, Canada; | Department of Integrative Biology, Guelph, Canada; ¶ Biodiversity Institute of Ontario, University of Guelph, Guelph, Canada

**Keywords:** Species distribution records, streamlining data, Barcode of Life Data Systems, Pensoft Writing Tool, Microgastrinae, Nearctic

## Abstract

The Barcode of Life Data Systems (BOLD) is designed to support the generation and application of DNA barcode data, but it also provides a unique source of data with potential for many research uses. This paper explores the streamlining of BOLD specimen data to record species distributions – and its fast publication using the Biodiversity Data Journal (BDJ), and its authoring platform, the Pensoft Writing Tool (PWT). We selected a sample of 630 specimens and 10 species of a highly diverse group of parasitoid wasps (Hymenoptera: Braconidae, Microgastrinae) from the Nearctic region and used the information in BOLD to uncover a significant number of new records (of locality, provinces, territories and states). By converting specimen information (such as locality, collection date, collector, voucher depository) from the BOLD platform to the Excel template provided by the PWT, it is possible to quickly upload and generate long lists of "Material Examined" for papers discussing taxonomy, ecology and/or new distribution records of species. For the vast majority of publications including DNA barcodes, the generation and publication of ancillary data associated with the barcoded material is seldom highlighted and often disregarded, and the analysis of those data sets to uncover new distribution patterns of species has rarely been explored, even though many BOLD records represent new and/or significant discoveries. The introduction of journals specializing in – and streamlining – the release of these datasets, such as the BDJ, should facilitate thorough analysis of these records, as shown in this paper.

## Introduction

The Barcode of Life Data Systems (BOLD) is designed to support the generation and application of DNA barcode data. The platform consists of four main modules: a data portal, a database of barcode clusters, an educational portal, and a data collection workbench. Currently almost 4 million sequences (over 3.4 million of them DNA barcodes) are stored in BOLD, including coverage for more than 143K animal species, 53K plant species, and 16K fungi and other species (http://www.boldsystems.org/, accessed on October 09, 2014). This immense platform provides a unique source of data with potential for many research uses. For example, the use of sequences in BOLD has been greatly expanded in the past few years, and dozens of publications using BOLD exist.

A less known and explored avenue is the use of specimen data (also stored in BOLD), which has a great potential to reveal new biodiversity information as well. This paper explores the streamlining of BOLD specimen data to record species distributions. For that we use the Biodiversity Data Journal (BDJ), and its authoring platform, the Pensoft Writing Tool (PWT), which is a community peer-reviewed, open-access, comprehensive online platform, designed to accelerate publishing, dissemination and sharing of biodiversity-related data of any kind (http://biodiversitydatajournal.com/about#FocusandScope).

A highly diverse group of insects (parasitoid wasps of the family Braconidae, subfamily Microgastrinae) was used as a case study. Microgastrine wasps are among the best represented groups of Hymenoptera in BOLD, and over 20K sequences have been already released and are publicly available (e.g., Smith et al. 2012). Microgastrinae is also one of the largest and most important groups of caterpillar parasitoids, with great importance in biological control programs worldwide (e.g., Whitfield 1997).

## Materials and methods

All specimens used for this study are deposited in the collections of the Biodiversity Institute of Ontario, University of Guelph (BIO), or the Canadian National Collection of Insects, Ottawa (CNC). They represent dozens of collecting events and different research projects carried out by those institutions. Most of the specimens rendered partial or full DNA barcodes (information can be retrieved using the following public DOI: http://dx.doi.org/10.5883/DS-NEAMICRO), and many have associated sequences, images and/or data from their labels in collections (Suppl. materials 1, 2, 3, 4).

We selected a sample of 630 specimens of parasitoid wasps, representing ten species and five genera of Microgastrinae (Hymenoptera: Braconidae) from the Nearctic region (Canada and the United States). The identity of the species was confirmed by JFT against authenticated material deposited in the CNC.

We downloaded the “Data Spreadsheet” associated with those records, including the worksheets “Collection Data” and “Specimens Details” (for a detailed explanation consult the “BOLD Print Handbook for BOLD v3”, freely available online at http://www.boldsystems.org/libhtml_v3/static/BOLD_Handbook_Oct2013.pdf).

The Excel file downloaded from BOLD (Suppl. material 1) was then adapted to the template provided by BDJ (a DarwinCore Compliant Excel file). Fields that were of interest for this study (e.g., locality data, collecting date, collectors, institution storing the specimens) were pasted to the corresponding columns in the BDJ template (Suppl. material 2), and from there directly imported into the Pensoft Writing Tool (the tool used to produce manuscripts for consequent submission to the BDJ). The information was uploaded as part of “Materials” for every individual species included in this study.

We compared the known distributions of the species (using mostly Fernandez-Triana 2010, Fernandez-Triana et al. 2014, Whitfield 2006, Yu et al. 2012, Fernández-Triana and Huber 2010) against the information stored in BOLD. In doing so, a significant number of new records (of locality, provinces, territories and states) were uncovered, which are detailed and discussed for every individual species under the section “Notes”. The distribution records of the specimens involved in this study (Suppl. material 5) were mapped using SimpleMappr (http://www.simplemappr.net).

The ten species analyzed here represented 13 Barcode Index Numbers (BINS), sensu Ratnasingham and Hebert (2013). In three cases, single names were found to contain multiple clusters of COI variation (Suppl. materials 4, 5). Since such variation has often been ascribed to cryptic or hidden species we examine each case under the section "Notes" for those particular species.

A few specimens discussed in this paper did not render any DNA and thus do not have any associated sequence in BOLD. However, their ancillary data (e.g., collecting date, locality, etc.) was still available and thus is presented below.

When discussing the distribution of species, states of the United States and provinces/territories of Canada are abbreviated with acronyms consisting of two capital letters, following Canada Post standards (http://www.canadapost.ca/tools/pg/manual/PGaddress-e.asp).

## Checklists

### Species List

#### Apanteles carpatus

(Say, 1836)

##### Materials

- **Type status:** Other material. **Occurrence:** catalogNumber: CAM0023; recordedBy: H. Goulet; lifeStage: Adult; otherCatalogNumbers: CNCHZ213-09; **Location:** country: Canada; stateProvince: Ontario; county: Ottawa; locality: Ottawa city garden; verbatimLatitude: 45.356; verbatimLongitude: -75.707; **Identification:** identifiedBy: Jose Fernandez-Triana; **Event:** verbatimEventDate: 19-Sep-2007; **Record Level:** institutionID: Canadian National Collection of Insects, Arachnids and Nematodes
- **Type status:** Other material. **Occurrence:** catalogNumber: BIOUG01088-A03; recordedBy: James Sones; lifeStage: Adult; otherCatalogNumbers: JSHYO003-11; **Location:** country: Canada; stateProvince: Ontario; county: Leeds and Grenville; municipality: Elizabethtown-Kitley; locality: 4452 Rowsome Rd., Elizabethtown; verbatimElevation: 112; verbatimLatitude: 44.621; verbatimLongitude: -75.773; **Identification:** identifiedBy: Angela Telfer; **Event:** verbatimEventDate: 17-Jul-2010; **Record Level:** institutionID: Biodiversity Institute of Ontario
- **Type status:** Other material. **Occurrence:** catalogNumber: CAM0036; recordedBy: H. Goulet; lifeStage: Adult; otherCatalogNumbers: CNCHZ226-09; **Location:** country: Canada; stateProvince: Ontario; county: Ottawa; locality: Ottawa city garden; verbatimLatitude: 45.356; verbatimLongitude: -75.707; **Identification:** identifiedBy: Jose Fernandez-Triana; **Event:** verbatimEventDate: 10-Aug-2007; **Record Level:** institutionID: Canadian National Collection of Insects, Arachnids and Nematodes
- **Type status:** Other material. **Occurrence:** catalogNumber: MIC 000034; recordedBy: Dom. Par. Lab.; sex: Female; lifeStage: Adult; otherCatalogNumbers: MHABR034-09; **Location:** country: Canada; stateProvince: Ontario; county: Essex County; locality: Ruthven; **Identification:** identifiedBy: Jose Fernandez-Triana; **Event:** verbatimEventDate: 18-Jul-1947; **Record Level:** institutionID: Canadian National Collection of Insects, Arachnids and Nematodes
- **Type status:** Other material. **Occurrence:** catalogNumber: MIC 000041; recordedBy: N. C. D. A; sex: Male; lifeStage: Adult; otherCatalogNumbers: MHABR041-09; **Location:** country: United States; stateProvince: North Carolina; county: Bertie County; locality: near Cahaba; **Identification:** identifiedBy: Jose Fernandez-Triana; **Event:** verbatimEventDate: 02-Jun-1976; **Record Level:** institutionID: Canadian National Collection of Insects, Arachnids and Nematodes
- **Type status:** Other material. **Occurrence:** catalogNumber: MIC 000036; recordedBy: Phillips; sex: Female; lifeStage: Adult; otherCatalogNumbers: MHABR036-09; **Location:** country: Canada; stateProvince: Ontario; locality: Vineland; **Identification:** identifiedBy: Jose Fernandez-Triana; **Event:** verbatimEventDate: 17-Aug-1974; **Record Level:** institutionID: Canadian National Collection of Insects, Arachnids and Nematodes
- **Type status:** Other material. **Occurrence:** catalogNumber: BIOUG01631-B09; recordedBy: James Sones; lifeStage: Adult; otherCatalogNumbers: JSAUG1452-11; **Location:** country: Canada; stateProvince: Ontario; county: Leeds and Grenville; municipality: Elizabethtown-Kitley; locality: 4452 Rowsome Rd., Elizabethtown; verbatimElevation: 111; verbatimLatitude: 44.618; verbatimLongitude: -75.775; **Identification:** identifiedBy: Angela Telfer; **Event:** verbatimEventDate: 18-Aug-2011; **Record Level:** institutionID: Biodiversity Institute of Ontario
- **Type status:** Other material. **Occurrence:** catalogNumber: CAM0104; recordedBy: H. Goulet; lifeStage: Adult; otherCatalogNumbers: CNCHZ294-09; **Location:** country: Canada; stateProvince: Ontario; county: Ottawa; locality: Ottawa city garden; verbatimLatitude: 45.356; verbatimLongitude: -75.707; **Identification:** identifiedBy: Jose Fernandez-Triana; **Event:** verbatimEventDate: 08-Sep-2007; **Record Level:** institutionID: Canadian National Collection of Insects, Arachnids and Nematodes
- **Type status:** Other material. **Occurrence:** catalogNumber: MIC 000035; recordedBy: J. Vockeroth; sex: Female; lifeStage: Adult; otherCatalogNumbers: MHABR035-09; **Location:** country: Canada; stateProvince: Ontario; county: Ottawa; locality: Ottawa; **Identification:** identifiedBy: Jose Fernandez-Triana; **Event:** verbatimEventDate: 15-Sep-1955; **Record Level:** institutionID: Canadian National Collection of Insects, Arachnids and Nematodes
- **Type status:** Other material. **Occurrence:** catalogNumber: CNCHYM 00081; recordedBy: N. C. D. A; lifeStage: Adult; otherCatalogNumbers: HYCND1790-11; **Location:** country: United States; stateProvince: North Carolina; county: Bertie County; locality: near Cahaba; **Identification:** identifiedBy: Jose Fernandez-Triana; **Event:** verbatimEventDate: 02-Jun-1976; **Record Level:** institutionID: Canadian National Collection of Insects, Arachnids and Nematodes
- **Type status:** Other material. **Occurrence:** catalogNumber: CAM0117; recordedBy: H. Goulet; lifeStage: Adult; otherCatalogNumbers: CNCHZ307-09; **Location:** country: Canada; stateProvince: Ontario; county: Ottawa; locality: Ottawa city garden; verbatimLatitude: 45.356; verbatimLongitude: -75.707; **Identification:** identifiedBy: Jose Fernandez-Triana; **Event:** verbatimEventDate: 30-Jul-2007; **Record Level:** institutionID: Canadian National Collection of Insects, Arachnids and Nematodes
- **Type status:** Other material. **Occurrence:** catalogNumber: CAM0021; recordedBy: H. Goulet; lifeStage: Adult; otherCatalogNumbers: CNCHZ211-09; **Location:** country: Canada; stateProvince: Ontario; county: Ottawa; locality: Ottawa city garden; verbatimLatitude: 45.356; verbatimLongitude: -75.707; **Identification:** identifiedBy: Jose Fernandez-Triana; **Event:** verbatimEventDate: 13-Jul-2007; **Record Level:** institutionID: Canadian National Collection of Insects, Arachnids and Nematodes
- **Type status:** Other material. **Occurrence:** catalogNumber: CAM0046; recordedBy: H. Goulet; lifeStage: Adult; otherCatalogNumbers: CNCHZ236-09; **Location:** country: Canada; stateProvince: Ontario; county: Ottawa; locality: Ottawa city garden; verbatimLatitude: 45.356; verbatimLongitude: -75.707; **Identification:** identifiedBy: Jose Fernandez-Triana; **Event:** verbatimEventDate: 23-Jul-2007; **Record Level:** institutionID: Canadian National Collection of Insects, Arachnids and Nematodes
- **Type status:** Other material. **Occurrence:** catalogNumber: MIC 000040; recordedBy: N. C. D. A; sex: Male; lifeStage: Adult; otherCatalogNumbers: MHABR040-09; **Location:** country: United States; stateProvince: North Carolina; county: Bertie County; locality: near Cahaba; **Identification:** identifiedBy: Jose Fernandez-Triana; **Event:** verbatimEventDate: 02-Jun-1976; **Record Level:** institutionID: Canadian National Collection of Insects, Arachnids and Nematodes
- **Type status:** Other material. **Occurrence:** catalogNumber: CAM0053; recordedBy: H. Goulet; lifeStage: Adult; otherCatalogNumbers: CNCHZ243-09; **Location:** country: Canada; stateProvince: Ontario; county: Ottawa; locality: Ottawa city garden; verbatimLatitude: 45.356; verbatimLongitude: -75.707; **Identification:** identifiedBy: Jose Fernandez-Triana; **Event:** verbatimEventDate: 23-Jul-2007; **Record Level:** institutionID: Canadian National Collection of Insects, Arachnids and Nematodes
- **Type status:** Other material. **Occurrence:** catalogNumber: CAM0063; recordedBy: H. Goulet; lifeStage: Adult; otherCatalogNumbers: CNCHZ253-09; **Location:** country: Canada; stateProvince: Ontario; county: Ottawa; locality: Ottawa city garden; verbatimLatitude: 45.356; verbatimLongitude: -75.707; **Identification:** identifiedBy: Jose Fernandez-Triana; **Event:** verbatimEventDate: 13-Jul-2007; **Record Level:** institutionID: Canadian National Collection of Insects, Arachnids and Nematodes
- **Type status:** Other material. **Occurrence:** catalogNumber: CAM0020; recordedBy: H. Goulet; lifeStage: Adult; otherCatalogNumbers: CNCHZ210-09; **Location:** country: Canada; stateProvince: Ontario; county: Ottawa; locality: Ottawa city garden; verbatimLatitude: 45.356; verbatimLongitude: -75.707; **Identification:** identifiedBy: Jose Fernandez-Triana; **Event:** verbatimEventDate: 13-Jul-2007; **Record Level:** institutionID: Canadian National Collection of Insects, Arachnids and Nematodes
- **Type status:** Other material. **Occurrence:** catalogNumber: CAM0058; recordedBy: H. Goulet; lifeStage: Adult; otherCatalogNumbers: CNCHZ248-09; **Location:** country: Canada; stateProvince: Ontario; county: Ottawa; locality: Ottawa city garden; verbatimLatitude: 45.356; verbatimLongitude: -75.707; **Identification:** identifiedBy: Jose Fernandez-Triana; **Event:** verbatimEventDate: 13-Jul-2007; **Record Level:** institutionID: Canadian National Collection of Insects, Arachnids and Nematodes
- **Type status:** Other material. **Occurrence:** catalogNumber: CAM0026; recordedBy: H. Goulet; lifeStage: Adult; otherCatalogNumbers: CNCHZ216-09; **Location:** country: Canada; stateProvince: Ontario; county: Ottawa; locality: Ottawa city garden; verbatimLatitude: 45.356; verbatimLongitude: -75.707; **Identification:** identifiedBy: Jose Fernandez-Triana; **Event:** verbatimEventDate: 30-Jul-2007; **Record Level:** institutionID: Canadian National Collection of Insects, Arachnids and Nematodes
- **Type status:** Other material. **Occurrence:** catalogNumber: CAM0049; recordedBy: H. Goulet; lifeStage: Adult; otherCatalogNumbers: CNCHZ239-09; **Location:** country: Canada; stateProvince: Ontario; county: Ottawa; locality: Ottawa city garden; verbatimLatitude: 45.356; verbatimLongitude: -75.707; **Identification:** identifiedBy: Jose Fernandez-Triana; **Event:** verbatimEventDate: 23-Jul-2007; **Record Level:** institutionID: Canadian National Collection of Insects, Arachnids and Nematodes
- **Type status:** Other material. **Occurrence:** catalogNumber: MIC 000037; recordedBy: D. Finnamore; sex: Female; lifeStage: Adult; otherCatalogNumbers: MHABR037-09; **Location:** country: Canada; stateProvince: New Brunswick; county: York County; **Identification:** identifiedBy: Jose Fernandez-Triana; **Event:** verbatimEventDate: 21-Oct-1981; **Record Level:** institutionID: Canadian National Collection of Insects, Arachnids and Nematodes
- **Type status:** Other material. **Occurrence:** catalogNumber: MIC 000033; recordedBy: C. Twinn; sex: Female; lifeStage: Adult; otherCatalogNumbers: MHABR033-09; **Location:** country: Canada; stateProvince: Ontario; county: Ottawa; locality: Ottawa; **Identification:** identifiedBy: Jose Fernandez-Triana; **Event:** verbatimEventDate: 08-May-1928; **Record Level:** institutionID: Canadian National Collection of Insects, Arachnids and Nematodes
- **Type status:** Other material. **Occurrence:** catalogNumber: MIC 000038; sex: Female; lifeStage: Adult; otherCatalogNumbers: MHABR038-09; **Location:** country: Canada; stateProvince: British Columbia; locality: Aldergrove, on a poultry barn; **Identification:** identifiedBy: Jose Fernandez-Triana; **Event:** verbatimEventDate: 15-Jul-1978; **Record Level:** institutionID: Canadian National Collection of Insects, Arachnids and Nematodes
- **Type status:** Other material. **Occurrence:** catalogNumber: CAM0042; recordedBy: H. Goulet; lifeStage: Adult; otherCatalogNumbers: CNCHZ232-09; **Location:** country: Canada; stateProvince: Ontario; county: Ottawa; locality: Ottawa city garden; verbatimLatitude: 45.356; verbatimLongitude: -75.707; **Identification:** identifiedBy: Jose Fernandez-Triana; **Event:** verbatimEventDate: 10-Aug-2007; **Record Level:** institutionID: Canadian National Collection of Insects, Arachnids and Nematodes

##### Notes

A cosmopolitan species (Yu et al. 2012), it was recorded from three provinces in Canada (BC, NB, ON) by Fernandez-Triana (2010), but no locality records were provided at the time. Here we present new details about the distribution of this species in Canada, based on examined specimens from the BIO and CNC collections. Data for those specimens was extracted from BOLD.

#### Apanteles conanchetorum

Viereck, 1917

##### Materials

- **Type status:** Other material. **Occurrence:** catalogNumber: CNCHYM 00088; recordedBy: N. C. D. A; lifeStage: Adult; otherCatalogNumbers: HYCND1797-11; **Location:** country: United States; stateProvince: North Carolina; county: Orange County; locality: Chapel Hill; **Identification:** identifiedBy: Jose Fernandez-Triana; **Event:** verbatimEventDate: 14-Jul-1976; **Record Level:** institutionID: Canadian National Collection of Insects, Arachnids and Nematodes
- **Type status:** Other material. **Occurrence:** catalogNumber: MIC 000908; recordedBy: H. Goulet, A. Badiss, C. Boudeault; lifeStage: Adult; otherCatalogNumbers: CNCHZ1100-09; **Location:** country: Canada; stateProvince: Newfoundland and Labrador; locality: St-Andrew`s; verbatimLatitude: 47.793; verbatimLongitude: -59.233; **Identification:** identifiedBy: Jose Fernandez-Triana; **Event:** verbatimEventDate: 18-Jul-2008; **Record Level:** institutionID: Canadian National Collection of Insects, Arachnids and Nematodes
- **Type status:** Other material. **Occurrence:** catalogNumber: BIOUG10353-H05; recordedBy: F.Tremblay; lifeStage: Adult; otherCatalogNumbers: CNFNF286-14; **Location:** country: Canada; stateProvince: Quebec; county: Forillon National Park; municipality: trail off of park compound (i.e. operation centre), 1501 boul. Forillon; locality: mixed forest (spruce, poplar); verbatimElevation: 135; verbatimLatitude: 48.857; verbatimLongitude: -64.376; **Event:** verbatimEventDate: 18-Jul-2013; **Record Level:** institutionID: Biodiversity Institute of Ontario
- **Type status:** Other material. **Occurrence:** catalogNumber: MIC 000905; recordedBy: H. Goulet, A. Badiss, C. Boudeault; lifeStage: Adult; otherCatalogNumbers: CNCHZ1097-09; **Location:** country: Canada; stateProvince: Newfoundland and Labrador; locality: St-Andrew`s; verbatimLatitude: 47.793; verbatimLongitude: -59.233; **Identification:** identifiedBy: Jose Fernandez-Triana; **Event:** verbatimEventDate: 18-Jul-2008; **Record Level:** institutionID: Canadian National Collection of Insects, Arachnids and Nematodes
- **Type status:** Other material. **Occurrence:** catalogNumber: BIOUG00989-E12; recordedBy: Alex Smith; lifeStage: Adult; otherCatalogNumbers: ASGLF313-11; **Location:** country: Canada; stateProvince: Ontario; county: Haldimond-Dunn Townline; municipality: Windy Bluff; verbatimElevation: 179; verbatimLatitude: 42.861; verbatimLongitude: -79.703; **Identification:** identifiedBy: Angela Telfer; **Event:** verbatimEventDate: 19-Sep-2010; **Record Level:** institutionID: Research Collection of M. Alex Smith
- **Type status:** Other material. **Occurrence:** catalogNumber: BIOUG01252-E11; recordedBy: James Sones; lifeStage: Adult; otherCatalogNumbers: JSHYO534-11; **Location:** country: Canada; stateProvince: Ontario; county: Leeds and Grenville; municipality: Elizabethtown-Kitley; locality: 4452 Rowsome Rd., Elizabethtown; verbatimElevation: 112; verbatimLatitude: 44.621; verbatimLongitude: -75.773; **Identification:** identifiedBy: BOLD ID Engine; **Event:** verbatimEventDate: 03-Aug-2010; **Record Level:** institutionID: Biodiversity Institute of Ontario
- **Type status:** Other material. **Occurrence:** catalogNumber: BIOUG07019-B11; recordedBy: Cyndi Smith; lifeStage: Adult; otherCatalogNumbers: CNWLM799-13; **Location:** country: Canada; stateProvince: Alberta; county: Waterton Lakes NP; municipality: Foothills Parkland Region; verbatimElevation: 1338; verbatimLatitude: 49.083; verbatimLongitude: -113.876; **Identification:** identifiedBy: Angela Telfer; **Event:** verbatimEventDate: 19-Jul-2012; **Record Level:** institutionID: Biodiversity Institute of Ontario
- **Type status:** Other material. **Occurrence:** catalogNumber: BIOUG10403-A09; recordedBy: F.Tremblay; lifeStage: Adult; otherCatalogNumbers: CNFNF871-14; **Location:** country: Canada; stateProvince: Quebec; county: Forillon National Park; municipality: trail off of park compound (i.e. operation centre), 1501 boul. Forillon; locality: mixed forest (spruce, poplar); verbatimElevation: 135; verbatimLatitude: 48.857; verbatimLongitude: -64.376; **Event:** verbatimEventDate: 22-Jul-2013; **Record Level:** institutionID: Biodiversity Institute of Ontario
- **Type status:** Other material. **Occurrence:** catalogNumber: MIC 000063; sex: Female; lifeStage: Adult; otherCatalogNumbers: MHABR063-09; **Location:** country: United States; stateProvince: Kansas; locality: Manhattan; **Identification:** identifiedBy: Jose Fernandez-Triana; **Event:** verbatimEventDate: 15-Aug-1945; **Record Level:** institutionID: Canadian National Collection of Insects, Arachnids and Nematodes
- **Type status:** Other material. **Occurrence:** catalogNumber: BIOUG10360-E02; recordedBy: F.Tremblay; lifeStage: Adult; otherCatalogNumbers: CNFNF627-14; **Location:** country: Canada; stateProvince: Quebec; county: Forillon National Park; municipality: trail off of park compound (i.e. operation centre), 1501 boul. Forillon; locality: mixed forest (spruce, poplar); verbatimElevation: 135; verbatimLatitude: 48.857; verbatimLongitude: -64.376; **Event:** verbatimEventDate: 18-Jul-2013; **Record Level:** institutionID: Biodiversity Institute of Ontario
- **Type status:** Other material. **Occurrence:** catalogNumber: BIOUG10358-C12; recordedBy: F.Tremblay; lifeStage: Adult; otherCatalogNumbers: CNFNF423-14; **Location:** country: Canada; stateProvince: Quebec; county: Forillon National Park; municipality: trail off of park compound (i.e. operation centre), 1501 boul. Forillon; locality: mixed forest (spruce, poplar); verbatimElevation: 135; verbatimLatitude: 48.857; verbatimLongitude: -64.376; **Event:** verbatimEventDate: 18-Jul-2013; **Record Level:** institutionID: Biodiversity Institute of Ontario
- **Type status:** Other material. **Occurrence:** catalogNumber: BIOUG00989-F01; recordedBy: Alex Smith; lifeStage: Adult; otherCatalogNumbers: ASGLF314-11; **Location:** country: Canada; stateProvince: Ontario; county: Haldimond-Dunn Townline; municipality: Windy Bluff; verbatimElevation: 179; verbatimLatitude: 42.861; verbatimLongitude: -79.703; **Identification:** identifiedBy: Angela Telfer; **Event:** verbatimEventDate: 19-Sep-2010; **Record Level:** institutionID: Research Collection of M. Alex Smith
- **Type status:** Other material. **Occurrence:** catalogNumber: BIOUG11905-C04; recordedBy: F.Tremblay; lifeStage: Adult; otherCatalogNumbers: CNFNR2363-14; **Location:** country: Canada; stateProvince: Quebec; county: Forillon National Park; municipality: trail off of park compound (i.e. operation centre), 1501 boul. Forillon; locality: mixed forest (spruce, poplar); verbatimElevation: 135; verbatimLatitude: 48.857; verbatimLongitude: -64.376; **Identification:** identifiedBy: Angela Telfer; **Event:** verbatimEventDate: 15-Jul-2013; **Record Level:** institutionID: Biodiversity Institute of Ontario
- **Type status:** Other material. **Occurrence:** catalogNumber: MIC 000902; recordedBy: H. Goulet, A. Badiss, C. Boudeault; lifeStage: Adult; otherCatalogNumbers: CNCHZ1094-09; **Location:** country: Canada; stateProvince: Newfoundland and Labrador; locality: Cap St-George, fallow field; verbatimLatitude: 48.466; verbatimLongitude: -59.260; **Identification:** identifiedBy: Jose Fernandez-Triana; **Event:** verbatimEventDate: 11-Jul-2008; **Record Level:** institutionID: Canadian National Collection of Insects, Arachnids and Nematodes
- **Type status:** Other material. **Occurrence:** catalogNumber: MIC 000058; recordedBy: J. McDunnough; sex: Female; lifeStage: Adult; otherCatalogNumbers: MHABR058-09; **Location:** country: Canada; stateProvince: Nova Scotia; county: Queens County; locality: White Pt. Beach; **Identification:** identifiedBy: Jose Fernandez-Triana; **Event:** verbatimEventDate: 15-Aug-1935; **Record Level:** institutionID: Canadian National Collection of Insects, Arachnids and Nematodes
- **Type status:** Other material. **Occurrence:** catalogNumber: CAM0096; recordedBy: L. Masner; lifeStage: Adult; otherCatalogNumbers: CNCHZ286-09; **Location:** country: Canada; stateProvince: Ontario; county: Ottawa; locality: Ottawa city, Slack Road; **Identification:** identifiedBy: Jose Fernandez-Triana; **Event:** verbatimEventDate: 05-Oct-2007; **Record Level:** institutionID: Canadian National Collection of Insects, Arachnids and Nematodes
- **Type status:** Other material. **Occurrence:** catalogNumber: BIOUG11907-E10; recordedBy: F.Tremblay; lifeStage: Adult; otherCatalogNumbers: CNFNR2583-14; **Location:** country: Canada; stateProvince: Quebec; county: Forillon National Park; municipality: trail off of park compound (i.e. operation centre), 1501 boul. Forillon; locality: mixed forest (spruce, poplar); verbatimElevation: 135; verbatimLatitude: 48.857; verbatimLongitude: -64.376; **Identification:** identifiedBy: Angela Telfer; **Event:** verbatimEventDate: 15-Jul-2013; **Record Level:** institutionID: Biodiversity Institute of Ontario
- **Type status:** Other material. **Occurrence:** catalogNumber: CAM1013; recordedBy: H. Goulet; lifeStage: Adult; otherCatalogNumbers: CNCHX143-09; **Location:** country: Canada; stateProvince: Ontario; county: Ottawa; locality: Ottwa city garden; verbatimLatitude: 45.356; verbatimLongitude: -75.707; **Identification:** identifiedBy: Jose Fernandez-Triana; **Record Level:** institutionID: Canadian National Collection of Insects, Arachnids and Nematodes
- **Type status:** Other material. **Occurrence:** catalogNumber: BIOUG01631-H02; recordedBy: James Sones; lifeStage: Adult; otherCatalogNumbers: JSSEP761-11; **Location:** country: Canada; stateProvince: Ontario; county: Leeds and Grenville; municipality: Elizabethtown-Kitley; locality: 4452 Rowsome Rd., Elizabethtown; verbatimElevation: 111; verbatimLatitude: 44.618; verbatimLongitude: -75.775; **Identification:** identifiedBy: BOLD ID Engine; **Event:** verbatimEventDate: 14-Sep-2011; **Record Level:** institutionID: Biodiversity Institute of Ontario
- **Type status:** Other material. **Occurrence:** catalogNumber: CNCHYM 00089; recordedBy: N. C. D. A; lifeStage: Adult; otherCatalogNumbers: HYCND1798-11; **Location:** country: United States; stateProvince: North Carolina; county: Davie County; locality: near Mocksvile; **Identification:** identifiedBy: Jose Fernandez-Triana; **Event:** verbatimEventDate: 04-Oct-1976; **Record Level:** institutionID: Canadian National Collection of Insects, Arachnids and Nematodes
- **Type status:** Other material. **Occurrence:** catalogNumber: BIOUG04245-B10; recordedBy: Jarret Hardisty; lifeStage: Adult; otherCatalogNumbers: CNWLF652-12; **Location:** country: Canada; stateProvince: Alberta; county: Waterton Lakes NP; municipality: Foothills Parkland Region; verbatimElevation: 1338; verbatimLatitude: 49.083; verbatimLongitude: -113.876; **Event:** verbatimEventDate: 26-Jul-2012; **Record Level:** institutionID: Biodiversity Institute of Ontario
- **Type status:** Other material. **Occurrence:** catalogNumber: BIOUG00989-E09; recordedBy: Alex Smith; lifeStage: Adult; otherCatalogNumbers: ASGLF310-11; **Location:** country: Canada; stateProvince: Ontario; county: Haldimond-Dunn Townline; municipality: Windy Bluff; verbatimElevation: 179; verbatimLatitude: 42.861; verbatimLongitude: -79.703; **Identification:** identifiedBy: Angela Telfer; **Event:** verbatimEventDate: 19-Sep-2010; **Record Level:** institutionID: Research Collection of M. Alex Smith
- **Type status:** Other material. **Occurrence:** catalogNumber: BIOUG11904-F02; recordedBy: F.Tremblay; lifeStage: Adult; otherCatalogNumbers: CNFNR2302-14; **Location:** country: Canada; stateProvince: Quebec; county: Forillon National Park; municipality: trail off of park compound (i.e. operation centre), 1501 boul. Forillon; locality: mixed forest (spruce, poplar); verbatimElevation: 135; verbatimLatitude: 48.857; verbatimLongitude: -64.376; **Identification:** identifiedBy: Angela Telfer; **Event:** verbatimEventDate: 15-Jul-2013; **Record Level:** institutionID: Biodiversity Institute of Ontario
- **Type status:** Other material. **Occurrence:** catalogNumber: 10BBCHY-2243; recordedBy: BIObus 2010; lifeStage: Adult; otherCatalogNumbers: BBHYK285-10; **Location:** country: Canada; stateProvince: Saskatchewan; county: Prince Albert NP; municipality: Red Deer Trl. Red Loop; verbatimElevation: 578; verbatimLatitude: 53.907; verbatimLongitude: -106.075; **Identification:** identifiedBy: Jose Fernandez-Triana; **Event:** verbatimEventDate: 14-Aug-2010; **Record Level:** institutionID: Biodiversity Institute of Ontario
- **Type status:** Other material. **Occurrence:** catalogNumber: MIC 000600; recordedBy: L. Masner; lifeStage: Adult; otherCatalogNumbers: CNCHZ792-09; **Location:** country: Canada; stateProvince: Ontario; locality: Woodlawn; **Identification:** identifiedBy: Jose Fernandez-Triana; **Event:** verbatimEventDate: 06-Aug-2008; **Record Level:** institutionID: Canadian National Collection of Insects, Arachnids and Nematodes
- **Type status:** Other material. **Occurrence:** catalogNumber: PCPP10-0759; recordedBy: T.F.Mitterboeck, C.Vandermeer, V.Junea, C.Sobel; lifeStage: Adult; otherCatalogNumbers: BBHYN214-10; **Location:** country: Canada; stateProvince: Ontario; county: Point Pelee NP; municipality: 15km SE of Leamington; locality: DeLaurier Marsh and Thicket Trail; verbatimElevation: 181; verbatimLatitude: 41.949; verbatimLongitude: -82.521; **Identification:** identifiedBy: Jose Fernandez-Triana; **Event:** verbatimEventDate: 28-Jun-2010; **Record Level:** institutionID: Biodiversity Institute of Ontario
- **Type status:** Other material. **Occurrence:** catalogNumber: BIOUG11909-G05; recordedBy: F.Tremblay; lifeStage: Adult; otherCatalogNumbers: CNFNR2792-14; **Location:** country: Canada; stateProvince: Quebec; county: Forillon National Park; municipality: trail off of park compound (i.e. operation centre), 1501 boul. Forillon; locality: mixed forest (spruce, poplar); verbatimElevation: 135; verbatimLatitude: 48.857; verbatimLongitude: -64.376; **Identification:** identifiedBy: Angela Telfer; **Event:** verbatimEventDate: 15-Jul-2013; **Record Level:** institutionID: Biodiversity Institute of Ontario
- **Type status:** Other material. **Occurrence:** catalogNumber: BIOUG00991-A05; recordedBy: Alex Smith; lifeStage: Adult; otherCatalogNumbers: ASGLF353-11; **Location:** country: Canada; stateProvince: Ontario; county: Haldimond-Dunn Townline; municipality: Windy Bluff; verbatimElevation: 179; verbatimLatitude: 42.861; verbatimLongitude: -79.703; **Identification:** identifiedBy: Angela Telfer; **Event:** verbatimEventDate: 23-Oct-2010; **Record Level:** institutionID: Research Collection of M. Alex Smith
- **Type status:** Other material. **Occurrence:** catalogNumber: BIOUG00914-E04; recordedBy: Alex Smith; lifeStage: Adult; otherCatalogNumbers: ASGLF115-11; **Location:** country: Canada; stateProvince: Ontario; county: Haldimond-Dunn Townline; municipality: Windy Bluff; verbatimElevation: 179; verbatimLatitude: 42.861; verbatimLongitude: -79.703; **Identification:** identifiedBy: Angela Telfer; **Event:** verbatimEventDate: 21-Nov-2010; **Record Level:** institutionID: Research Collection of M. Alex Smith
- **Type status:** Other material. **Occurrence:** catalogNumber: BIOUG00914-E03; recordedBy: Alex Smith; lifeStage: Adult; otherCatalogNumbers: ASGLF114-11; **Location:** country: Canada; stateProvince: Ontario; county: Haldimond-Dunn Townline; municipality: Windy Bluff; verbatimElevation: 179; verbatimLatitude: 42.861; verbatimLongitude: -79.703; **Identification:** identifiedBy: Angela Telfer; **Event:** verbatimEventDate: 21-Nov-2010; **Record Level:** institutionID: Research Collection of M. Alex Smith
- **Type status:** Other material. **Occurrence:** catalogNumber: BIOUG10961-E03; recordedBy: Paul Ayles; lifeStage: Adult; otherCatalogNumbers: CNPEP1205-14; **Location:** country: Canada; stateProvince: Prince Edward Island; county: Prince Edward Island National Park; municipality: Woodland Trail / Long Point; verbatimElevation: 6; verbatimLatitude: 46.412; verbatimLongitude: -63.085; **Event:** verbatimEventDate: 17-Jul-2013; **Record Level:** institutionID: Biodiversity Institute of Ontario
- **Type status:** Other material. **Occurrence:** catalogNumber: BIOUG08661-F09; recordedBy: Amy Fennell; lifeStage: Adult; otherCatalogNumbers: SMTPD5714-13; **Location:** country: Canada; stateProvince: Ontario; county: Campbellville; municipality: Mountsberg Conservation Area; verbatimElevation: 215; verbatimLatitude: 43.457; verbatimLongitude: -80.027; **Event:** verbatimEventDate: 28-Sep-2013; **Record Level:** institutionID: Biodiversity Institute of Ontario
- **Type status:** Other material. **Occurrence:** catalogNumber: BIOUG07067-E03; recordedBy: Emma Sylvester; lifeStage: Adult; otherCatalogNumbers: CNWLN171-13; **Location:** country: Canada; stateProvince: Alberta; county: Waterton Lakes NP; municipality: Foothills Parkland Region; verbatimElevation: 1338; verbatimLatitude: 49.083; verbatimLongitude: -113.876; **Event:** verbatimEventDate: 02-Aug-2012; **Record Level:** institutionID: Biodiversity Institute of Ontario
- **Type status:** Other material. **Occurrence:** catalogNumber: BIOUG01280-G04; recordedBy: James Sones; lifeStage: Adult; otherCatalogNumbers: JSHYP027-11; **Location:** country: Canada; stateProvince: Ontario; county: Leeds and Grenville; municipality: Elizabethtown-Kitley; locality: 4452 Rowsome Rd., Elizabethtown; verbatimElevation: 112; verbatimLatitude: 44.621; verbatimLongitude: -75.773; **Identification:** identifiedBy: BOLD ID Engine; **Event:** verbatimEventDate: 29-Aug-2010; **Record Level:** institutionID: Biodiversity Institute of Ontario
- **Type status:** Other material. **Occurrence:** catalogNumber: CAM0160; recordedBy: H. Goulet; lifeStage: Adult; otherCatalogNumbers: CNCHZ350-09; **Location:** country: Canada; stateProvince: Ontario; county: Ottawa; locality: Ottawa city garden; verbatimLatitude: 45.356; verbatimLongitude: -75.707; **Identification:** identifiedBy: Jose Fernandez-Triana; **Event:** verbatimEventDate: 01-Sep-2007; **Record Level:** institutionID: Canadian National Collection of Insects, Arachnids and Nematodes
- **Type status:** Other material. **Occurrence:** catalogNumber: BIOUG06873-E09; recordedBy: Cyndi Smith; lifeStage: Adult; otherCatalogNumbers: CNWLM287-13; **Location:** country: Canada; stateProvince: Alberta; county: Waterton Lakes NP; municipality: Foothills Parkland Region; verbatimElevation: 1338; verbatimLatitude: 49.083; verbatimLongitude: -113.876; **Event:** verbatimEventDate: 19-Jul-2012; **Record Level:** institutionID: Biodiversity Institute of Ontario
- **Type status:** Other material. **Occurrence:** catalogNumber: BIOUG12293-G01; recordedBy: F.Tremblay; lifeStage: Adult; otherCatalogNumbers: CNFNT1471-14; **Location:** country: Canada; stateProvince: Quebec; county: Forillon National Park; municipality: trail off of park compound (i.e. operation centre), 1501 boul. Forillon; locality: mixed forest (spruce, poplar); verbatimElevation: 135; verbatimLatitude: 48.857; verbatimLongitude: -64.376; **Identification:** identifiedBy: Angela Telfer; **Event:** verbatimEventDate: 12-Aug-2013; **Record Level:** institutionID: Biodiversity Institute of Ontario
- **Type status:** Other material. **Occurrence:** catalogNumber: BIOUG10408-B06; recordedBy: F.Tremblay; lifeStage: Adult; otherCatalogNumbers: CNFNF1260-14; **Location:** country: Canada; stateProvince: Quebec; county: Forillon National Park; municipality: trail off of park compound (i.e. operation centre), 1501 boul. Forillon; locality: mixed forest (spruce, poplar); verbatimElevation: 135; verbatimLatitude: 48.857; verbatimLongitude: -64.376; **Event:** verbatimEventDate: 18-Jul-2013; **Record Level:** institutionID: Biodiversity Institute of Ontario
- **Type status:** Other material. **Occurrence:** catalogNumber: BIOUG00989-E11; recordedBy: Alex Smith; lifeStage: Adult; otherCatalogNumbers: ASGLF312-11; **Location:** country: Canada; stateProvince: Ontario; county: Haldimond-Dunn Townline; municipality: Windy Bluff; verbatimElevation: 179; verbatimLatitude: 42.861; verbatimLongitude: -79.703; **Identification:** identifiedBy: Angela Telfer; **Event:** verbatimEventDate: 19-Sep-2010; **Record Level:** institutionID: Research Collection of M. Alex Smith
- **Type status:** Other material. **Occurrence:** catalogNumber: BIOUG10358-E06; recordedBy: F.Tremblay; lifeStage: Adult; otherCatalogNumbers: CNFNF441-14; **Location:** country: Canada; stateProvince: Quebec; county: Forillon National Park; municipality: trail off of park compound (i.e. operation centre), 1501 boul. Forillon; locality: mixed forest (spruce, poplar); verbatimElevation: 135; verbatimLatitude: 48.857; verbatimLongitude: -64.376; **Event:** verbatimEventDate: 22-Jul-2013; **Record Level:** institutionID: Biodiversity Institute of Ontario
- **Type status:** Other material. **Occurrence:** catalogNumber: BIOUG00861-G06; recordedBy: Paul Hebert; lifeStage: Adult; otherCatalogNumbers: PHMTX962-11; **Location:** country: Canada; stateProvince: Ontario; county: Wellington County; municipality: Puslinch Township; locality: Concession 11/Hume Rd; verbatimElevation: 320; verbatimLatitude: 43.537; verbatimLongitude: -80.134; **Identification:** identifiedBy: BOLD ID Engine; **Event:** verbatimEventDate: 15-Oct-2010; **Record Level:** institutionID: Biodiversity Institute of Ontario
- **Type status:** Other material. **Occurrence:** catalogNumber: BIOUG10550-D04; recordedBy: Dany Brodeau; lifeStage: Adult; otherCatalogNumbers: CNFNG557-14; **Location:** country: Canada; stateProvince: Quebec; county: Forillon National Park; municipality: trail off of park compound (i.e. operation centre), 1501 boul. Forillon; locality: mixed forest (spruce, poplar); verbatimElevation: 135; verbatimLatitude: 48.857; verbatimLongitude: -64.376; **Event:** verbatimEventDate: 05-Aug-2013; **Record Level:** institutionID: Biodiversity Institute of Ontario
- **Type status:** Other material. **Occurrence:** catalogNumber: BIOUG02507-D09; recordedBy: Paul Hebert; lifeStage: Adult; otherCatalogNumbers: HESEP836-12; **Location:** country: Canada; stateProvince: Ontario; county: Wellington County; municipality: Puslinch Township; locality: Concession 11/Hume Rd; verbatimElevation: 320; verbatimLatitude: 43.537; verbatimLongitude: -80.134; **Identification:** identifiedBy: BOLD ID Engine; **Event:** verbatimEventDate: 08-Sep-2010; **Record Level:** institutionID: Biodiversity Institute of Ontario
- **Type status:** Other material. **Occurrence:** catalogNumber: BIOUG10403-A08; recordedBy: F.Tremblay; lifeStage: Adult; otherCatalogNumbers: CNFNF870-14; **Location:** country: Canada; stateProvince: Quebec; county: Forillon National Park; municipality: trail off of park compound (i.e. operation centre), 1501 boul. Forillon; locality: mixed forest (spruce, poplar); verbatimElevation: 135; verbatimLatitude: 48.857; verbatimLongitude: -64.376; **Event:** verbatimEventDate: 18-Jul-2013; **Record Level:** institutionID: Biodiversity Institute of Ontario
- **Type status:** Other material. **Occurrence:** catalogNumber: BIOUG00914-E07; recordedBy: Alex Smith; lifeStage: Adult; otherCatalogNumbers: ASGLF118-11; **Location:** country: Canada; stateProvince: Ontario; county: Haldimond-Dunn Townline; municipality: Windy Bluff; verbatimElevation: 179; verbatimLatitude: 42.861; verbatimLongitude: -79.703; **Identification:** identifiedBy: Angela Telfer; **Event:** verbatimEventDate: 21-Nov-2010; **Record Level:** institutionID: Research Collection of M. Alex Smith
- **Type status:** Other material. **Occurrence:** catalogNumber: CAM0147; recordedBy: H. Goulet; lifeStage: Adult; otherCatalogNumbers: CNCHZ337-09; **Location:** country: Canada; stateProvince: Ontario; county: Ottawa; locality: Ottawa city garden; verbatimLatitude: 45.356; verbatimLongitude: -75.707; **Identification:** identifiedBy: Jose Fernandez-Triana; **Event:** verbatimEventDate: 01-Sep-2007; **Record Level:** institutionID: Canadian National Collection of Insects, Arachnids and Nematodes
- **Type status:** Other material. **Occurrence:** catalogNumber: BIOUG00988-A09; recordedBy: Alex Smith; lifeStage: Adult; otherCatalogNumbers: ASGLF167-11; **Location:** country: Canada; stateProvince: Ontario; county: Guelph; municipality: 25 Division St.; verbatimElevation: 326; verbatimLatitude: 43.554; verbatimLongitude: -80.264; **Identification:** identifiedBy: Angela Telfer; **Event:** verbatimEventDate: 29-Oct-2010; **Record Level:** institutionID: Research Collection of M. Alex Smith
- **Type status:** Other material. **Occurrence:** catalogNumber: BIOUG11910-C01; recordedBy: F.Tremblay; lifeStage: Adult; otherCatalogNumbers: CNFNR2835-14; **Location:** country: Canada; stateProvince: Quebec; county: Forillon National Park; municipality: trail off of park compound (i.e. operation centre), 1501 boul. Forillon; locality: mixed forest (spruce, poplar); verbatimElevation: 135; verbatimLatitude: 48.857; verbatimLongitude: -64.376; **Identification:** identifiedBy: Angela Telfer; **Event:** verbatimEventDate: 15-Jul-2013; **Record Level:** institutionID: Biodiversity Institute of Ontario
- **Type status:** Other material. **Occurrence:** catalogNumber: BIOUG04245-A01; recordedBy: Jarret Hardisty; lifeStage: Adult; otherCatalogNumbers: CNWLF631-12; **Location:** country: Canada; stateProvince: Alberta; county: Waterton Lakes NP; municipality: Foothills Parkland Region; verbatimElevation: 1338; verbatimLatitude: 49.083; verbatimLongitude: -113.876; **Event:** verbatimEventDate: 26-Jul-2012; **Record Level:** institutionID: Biodiversity Institute of Ontario
- **Type status:** Other material. **Occurrence:** catalogNumber: BIOUG00914-F08; recordedBy: Alex Smith; lifeStage: Adult; otherCatalogNumbers: ASGLF131-11; **Location:** country: Canada; stateProvince: Ontario; county: Haldimond-Dunn Townline; municipality: Windy Bluff; verbatimElevation: 179; verbatimLatitude: 42.861; verbatimLongitude: -79.703; **Identification:** identifiedBy: Angela Telfer; **Event:** verbatimEventDate: 21-Nov-2010; **Record Level:** institutionID: Research Collection of M. Alex Smith
- **Type status:** Other material. **Occurrence:** catalogNumber: BIOUG10661-F02; recordedBy: Dany Brodeau; lifeStage: Adult; otherCatalogNumbers: CNFNG1150-14; **Location:** country: Canada; stateProvince: Quebec; county: Forillon National Park; municipality: trail off of park compound (i.e. operation centre), 1501 boul. Forillon; locality: mixed forest (spruce, poplar); verbatimElevation: 135; verbatimLatitude: 48.857; verbatimLongitude: -64.376; **Event:** verbatimEventDate: 05-Aug-2013; **Record Level:** institutionID: Biodiversity Institute of Ontario
- **Type status:** Other material. **Occurrence:** catalogNumber: BIOUG11910-B12; recordedBy: F.Tremblay; lifeStage: Adult; otherCatalogNumbers: CNFNR2834-14; **Location:** country: Canada; stateProvince: Quebec; county: Forillon National Park; municipality: trail off of park compound (i.e. operation centre), 1501 boul. Forillon; locality: mixed forest (spruce, poplar); verbatimElevation: 135; verbatimLatitude: 48.857; verbatimLongitude: -64.376; **Identification:** identifiedBy: Angela Telfer; **Event:** verbatimEventDate: 15-Jul-2013; **Record Level:** institutionID: Biodiversity Institute of Ontario
- **Type status:** Other material. **Occurrence:** catalogNumber: CNCHYM 00093; lifeStage: Adult; otherCatalogNumbers: HYCND1803-11; **Location:** country: United States; stateProvince: Kansas; locality: Manhattan; **Identification:** identifiedBy: Jose Fernandez-Triana; **Event:** verbatimEventDate: 15-Aug-1945; **Record Level:** institutionID: Canadian National Collection of Insects, Arachnids and Nematodes
- **Type status:** Other material. **Occurrence:** catalogNumber: CAM0005; recordedBy: H. Goulet; lifeStage: Adult; otherCatalogNumbers: CNCHZ195-09; **Location:** country: Canada; stateProvince: Ontario; county: Ottawa; locality: Ottawa city garden; verbatimLatitude: 45.356; verbatimLongitude: -75.707; **Identification:** identifiedBy: Jose Fernandez-Triana; **Event:** verbatimEventDate: 19-Sep-2007; **Record Level:** institutionID: Canadian National Collection of Insects, Arachnids and Nematodes
- **Type status:** Other material. **Occurrence:** catalogNumber: CAM0085; recordedBy: H. Goulet; lifeStage: Adult; otherCatalogNumbers: CNCHZ275-09; **Location:** country: Canada; stateProvince: Ontario; county: Ottawa; locality: Ottawa city garden; verbatimLatitude: 45.356; verbatimLongitude: -75.707; **Identification:** identifiedBy: Jose Fernandez-Triana; **Event:** verbatimEventDate: 10-Aug-2007; **Record Level:** institutionID: Canadian National Collection of Insects, Arachnids and Nematodes
- **Type status:** Other material. **Occurrence:** catalogNumber: BIOUG00989-A08; recordedBy: Alex Smith; lifeStage: Adult; otherCatalogNumbers: ASGLF261-11; **Location:** country: Canada; stateProvince: Ontario; county: Guelph; municipality: 25 Division St.; verbatimElevation: 326; verbatimLatitude: 43.554; verbatimLongitude: -80.264; **Identification:** identifiedBy: Angela Telfer; **Event:** verbatimEventDate: 29-Oct-2010; **Record Level:** institutionID: Research Collection of M. Alex Smith
- **Type status:** Other material. **Occurrence:** catalogNumber: BIOUG08192-F10; recordedBy: BIOBus 2012; lifeStage: Adult; otherCatalogNumbers: SSPAC3799-13; **Location:** country: Canada; stateProvince: Saskatchewan; county: Prince Albert NP; municipality: Spruce River Highlands Trail; verbatimElevation: 551; verbatimLatitude: 53.712; verbatimLongitude: -106.048; **Event:** verbatimEventDate: 11-Jul-2012; **Record Level:** institutionID: Biodiversity Institute of Ontario
- **Type status:** Other material. **Occurrence:** catalogNumber: BIOUG01330-D09; recordedBy: James Sones; lifeStage: Adult; otherCatalogNumbers: JSHYP661-12; **Location:** country: Canada; stateProvince: Ontario; county: Leeds and Grenville; municipality: Elizabethtown-Kitley; locality: 4452 Rowsome Rd., Elizabethtown; verbatimElevation: 112; verbatimLatitude: 44.621; verbatimLongitude: -75.773; **Event:** verbatimEventDate: 18-Oct-2010; **Record Level:** institutionID: Biodiversity Institute of Ontario
- **Type status:** Other material. **Occurrence:** catalogNumber: BIOUG10353-G01; recordedBy: F.Tremblay; lifeStage: Adult; otherCatalogNumbers: CNFNF270-14; **Location:** country: Canada; stateProvince: Quebec; county: Forillon National Park; municipality: trail off of park compound (i.e. operation centre), 1501 boul. Forillon; locality: mixed forest (spruce, poplar); verbatimElevation: 135; verbatimLatitude: 48.857; verbatimLongitude: -64.376; **Event:** verbatimEventDate: 18-Jul-2013; **Record Level:** institutionID: Biodiversity Institute of Ontario
- **Type status:** Other material. **Occurrence:** catalogNumber: BIOUG00914-G01; recordedBy: Alex Smith; lifeStage: Adult; otherCatalogNumbers: ASGLF136-11; **Location:** country: Canada; stateProvince: Ontario; county: Haldimond-Dunn Townline; municipality: Windy Bluff; verbatimElevation: 179; verbatimLatitude: 42.861; verbatimLongitude: -79.703; **Identification:** identifiedBy: Angela Telfer; **Event:** verbatimEventDate: 21-Nov-2010; **Record Level:** institutionID: Research Collection of M. Alex Smith
- **Type status:** Other material. **Occurrence:** catalogNumber: BIOUG07067-C07; recordedBy: Cyndi Smith; lifeStage: Adult; otherCatalogNumbers: CNWLM1411-13; **Location:** country: Canada; stateProvince: Alberta; county: Waterton Lakes NP; municipality: Foothills Parkland Region; verbatimElevation: 1338; verbatimLatitude: 49.083; verbatimLongitude: -113.876; **Event:** verbatimEventDate: 19-Jul-2012; **Record Level:** institutionID: Biodiversity Institute of Ontario
- **Type status:** Other material. **Occurrence:** catalogNumber: BIOUG12037-E03; recordedBy: F.Tremblay; lifeStage: Adult; otherCatalogNumbers: CNFNS886-14; **Location:** country: Canada; stateProvince: Quebec; county: Forillon National Park; municipality: trail off of park compound (i.e. operation centre), 1501 boul. Forillon; locality: mixed forest (spruce, poplar); verbatimElevation: 135; verbatimLatitude: 48.857; verbatimLongitude: -64.376; **Identification:** identifiedBy: Angela Telfer; **Event:** verbatimEventDate: 30-Jul-2013; **Record Level:** institutionID: Biodiversity Institute of Ontario
- **Type status:** Other material. **Occurrence:** catalogNumber: MIC 000061; sex: Female; lifeStage: Adult; otherCatalogNumbers: MHABR061-09; **Location:** country: Canada; stateProvince: Ontario; county: St. Lawrence Islands National Park; locality: Grenadier Island Center; **Identification:** identifiedBy: Jose Fernandez-Triana; **Event:** verbatimEventDate: 02-Jul-1975; **Record Level:** institutionID: Canadian National Collection of Insects, Arachnids and Nematodes
- **Type status:** Other material. **Occurrence:** catalogNumber: BIOUG00989-E10; recordedBy: Alex Smith; lifeStage: Adult; otherCatalogNumbers: ASGLF311-11; **Location:** country: Canada; stateProvince: Ontario; county: Haldimond-Dunn Townline; municipality: Windy Bluff; verbatimElevation: 179; verbatimLatitude: 42.861; verbatimLongitude: -79.703; **Identification:** identifiedBy: Angela Telfer; **Event:** verbatimEventDate: 19-Sep-2010; **Record Level:** institutionID: Research Collection of M. Alex Smith
- **Type status:** Other material. **Occurrence:** catalogNumber: BIOUG10405-G04; recordedBy: F.Tremblay; lifeStage: Adult; otherCatalogNumbers: CNFNF1033-14; **Location:** country: Canada; stateProvince: Quebec; county: Forillon National Park; municipality: trail off of park compound (i.e. operation centre), 1501 boul. Forillon; locality: mixed forest (spruce, poplar); verbatimElevation: 135; verbatimLatitude: 48.857; verbatimLongitude: -64.376; **Event:** verbatimEventDate: 22-Jul-2013; **Record Level:** institutionID: Biodiversity Institute of Ontario
- **Type status:** Other material. **Occurrence:** catalogNumber: MIC 000060; sex: Female; lifeStage: Adult; otherCatalogNumbers: MHABR060-09; **Location:** country: Canada; stateProvince: Ontario; county: Aylmer; locality: Aylmer West; **Identification:** identifiedBy: Jose Fernandez-Triana; **Event:** verbatimEventDate: 20-Jul-1972; **Record Level:** institutionID: Canadian National Collection of Insects, Arachnids and Nematodes
- **Type status:** Other material. **Occurrence:** catalogNumber: CAM0159; recordedBy: H. Goulet; lifeStage: Adult; otherCatalogNumbers: CNCHZ349-09; **Location:** country: Canada; stateProvince: Ontario; county: Ottawa; locality: Ottawa city garden; verbatimLatitude: 45.356; verbatimLongitude: -75.707; **Identification:** identifiedBy: Jose Fernandez-Triana; **Event:** verbatimEventDate: 01-Sep-2007; **Record Level:** institutionID: Canadian National Collection of Insects, Arachnids and Nematodes
- **Type status:** Other material. **Occurrence:** catalogNumber: BIOUG13530-A05; recordedBy: BIObus 2013; lifeStage: Adult; otherCatalogNumbers: SSROB2643-14; **Location:** country: Canada; stateProvince: Ontario; county: Rouge National Urban Park; municipality: West of Glen Rouge campground; locality: Marsh scrub along riverside; verbatimElevation: 80; verbatimLatitude: 43.804; verbatimLongitude: -79.146; **Identification:** identifiedBy: Angela Telfer; **Event:** verbatimEventDate: 07-Jun-2013; **Record Level:** institutionID: Biodiversity Institute of Ontario
- **Type status:** Other material. **Occurrence:** catalogNumber: BIOUG00914-F06; recordedBy: Alex Smith; lifeStage: Adult; otherCatalogNumbers: ASGLF129-11; **Location:** country: Canada; stateProvince: Ontario; county: Haldimond-Dunn Townline; municipality: Windy Bluff; verbatimElevation: 179; verbatimLatitude: 42.861; verbatimLongitude: -79.703; **Identification:** identifiedBy: Angela Telfer; **Event:** verbatimEventDate: 21-Nov-2010; **Record Level:** institutionID: Research Collection of M. Alex Smith
- **Type status:** Other material. **Occurrence:** catalogNumber: CNCHYM 03970; recordedBy: N. C. D. A; lifeStage: Adult; otherCatalogNumbers: HYCND1800-11; **Location:** country: United States; stateProvince: North Carolina; county: Avery County; locality: Linville; **Identification:** identifiedBy: Jose Fernandez-Triana; **Event:** verbatimEventDate: 05-Oct-1976; **Record Level:** institutionID: Canadian National Collection of Insects, Arachnids and Nematodes
- **Type status:** Other material. **Occurrence:** catalogNumber: BIOUG04245-C02; recordedBy: Jarret Hardisty; lifeStage: Adult; otherCatalogNumbers: CNWLF656-12; **Location:** country: Canada; stateProvince: Alberta; county: Waterton Lakes NP; municipality: Foothills Parkland Region; verbatimElevation: 1338; verbatimLatitude: 49.083; verbatimLongitude: -113.876; **Event:** verbatimEventDate: 26-Jul-2012; **Record Level:** institutionID: Biodiversity Institute of Ontario
- **Type status:** Other material. **Occurrence:** catalogNumber: BIOUG00989-G12; recordedBy: Alex Smith; lifeStage: Adult; otherCatalogNumbers: ASGLF337-11; **Location:** country: Canada; stateProvince: Ontario; county: Haldimond-Dunn Townline; municipality: Windy Bluff; verbatimElevation: 179; verbatimLatitude: 42.861; verbatimLongitude: -79.703; **Identification:** identifiedBy: Angela Telfer; **Event:** verbatimEventDate: 19-Sep-2010; **Record Level:** institutionID: Research Collection of M. Alex Smith
- **Type status:** Other material. **Occurrence:** catalogNumber: BIOUG01309-D01; recordedBy: Paul Hebert; lifeStage: Adult; otherCatalogNumbers: PHSEP1889-11; **Location:** country: Canada; stateProvince: Ontario; county: Wellington County; municipality: Puslinch Township; locality: Concession 11/Hume Rd; verbatimElevation: 320; verbatimLatitude: 43.537; verbatimLongitude: -80.134; **Identification:** identifiedBy: BOLD ID Engine; **Event:** verbatimEventDate: 23-Sep-2010; **Record Level:** institutionID: Biodiversity Institute of Ontario
- **Type status:** Other material. **Occurrence:** catalogNumber: BIOUG00914-E08; recordedBy: Alex Smith; lifeStage: Adult; otherCatalogNumbers: ASGLF119-11; **Location:** country: Canada; stateProvince: Ontario; county: Haldimond-Dunn Townline; municipality: Windy Bluff; verbatimElevation: 179; verbatimLatitude: 42.861; verbatimLongitude: -79.703; **Identification:** identifiedBy: Angela Telfer; **Event:** verbatimEventDate: 21-Nov-2010; **Record Level:** institutionID: Research Collection of M. Alex Smith
- **Type status:** Other material. **Occurrence:** catalogNumber: BIOUG11908-H02; recordedBy: F.Tremblay; lifeStage: Adult; otherCatalogNumbers: CNFNR2706-14; **Location:** country: Canada; stateProvince: Quebec; county: Forillon National Park; municipality: trail off of park compound (i.e. operation centre), 1501 boul. Forillon; locality: mixed forest (spruce, poplar); verbatimElevation: 135; verbatimLatitude: 48.857; verbatimLongitude: -64.376; **Identification:** identifiedBy: Angela Telfer; **Event:** verbatimEventDate: 15-Jul-2013; **Record Level:** institutionID: Biodiversity Institute of Ontario
- **Type status:** Other material. **Occurrence:** catalogNumber: BIOUG11903-A01; recordedBy: F.Tremblay; lifeStage: Adult; otherCatalogNumbers: CNFNR2146-14; **Location:** country: Canada; stateProvince: Quebec; county: Forillon National Park; municipality: trail off of park compound (i.e. operation centre), 1501 boul. Forillon; locality: mixed forest (spruce, poplar); verbatimElevation: 135; verbatimLatitude: 48.857; verbatimLongitude: -64.376; **Identification:** identifiedBy: Angela Telfer; **Event:** verbatimEventDate: 15-Jul-2013; **Record Level:** institutionID: Biodiversity Institute of Ontario
- **Type status:** Other material. **Occurrence:** catalogNumber: BIOUG12293-B06; recordedBy: F.Tremblay; lifeStage: Adult; otherCatalogNumbers: CNFNT1416-14; **Location:** country: Canada; stateProvince: Quebec; county: Forillon National Park; municipality: trail off of park compound (i.e. operation centre), 1501 boul. Forillon; locality: mixed forest (spruce, poplar); verbatimElevation: 135; verbatimLatitude: 48.857; verbatimLongitude: -64.376; **Identification:** identifiedBy: Angela Telfer; **Event:** verbatimEventDate: 12-Aug-2013; **Record Level:** institutionID: Biodiversity Institute of Ontario
- **Type status:** Other material. **Occurrence:** catalogNumber: BIOUG10405-C03; recordedBy: F.Tremblay; lifeStage: Adult; otherCatalogNumbers: CNFNF984-14; **Location:** country: Canada; stateProvince: Quebec; county: Forillon National Park; municipality: trail off of park compound (i.e. operation centre), 1501 boul. Forillon; locality: mixed forest (spruce, poplar); verbatimElevation: 135; verbatimLatitude: 48.857; verbatimLongitude: -64.376; **Event:** verbatimEventDate: 18-Jul-2013; **Record Level:** institutionID: Biodiversity Institute of Ontario
- **Type status:** Other material. **Occurrence:** catalogNumber: BIOUG06873-G07; recordedBy: Cyndi Smith; lifeStage: Adult; otherCatalogNumbers: CNWLM309-13; **Location:** country: Canada; stateProvince: Alberta; county: Waterton Lakes NP; municipality: Foothills Parkland Region; verbatimElevation: 1338; verbatimLatitude: 49.083; verbatimLongitude: -113.876; **Event:** verbatimEventDate: 19-Jul-2012; **Record Level:** institutionID: Biodiversity Institute of Ontario
- **Type status:** Other material. **Occurrence:** catalogNumber: MIC 000059; recordedBy: J. McDunnough; sex: Female; lifeStage: Adult; otherCatalogNumbers: MHABR059-09; **Location:** country: Canada; stateProvince: Nova Scotia; county: Queens County; locality: White Pt. Beach; **Identification:** identifiedBy: Jose Fernandez-Triana; **Event:** verbatimEventDate: 15-Aug-1935; **Record Level:** institutionID: Canadian National Collection of Insects, Arachnids and Nematodes
- **Type status:** Other material. **Occurrence:** catalogNumber: CNCHYM 00090; recordedBy: N. C. D. A; lifeStage: Adult; otherCatalogNumbers: HYCND1799-11; **Location:** country: United States; stateProvince: North Carolina; county: Martin County; locality: near Williamston; **Identification:** identifiedBy: Jose Fernandez-Triana; **Event:** verbatimEventDate: 16-Jun-1975; **Record Level:** institutionID: Canadian National Collection of Insects, Arachnids and Nematodes
- **Type status:** Other material. **Occurrence:** catalogNumber: MIC 000064; sex: Male; lifeStage: Adult; otherCatalogNumbers: MHABR064-09; **Location:** country: Canada; stateProvince: Ontario; county: St. Lawrence Islands National Park; locality: Thwartway Island; **Identification:** identifiedBy: Jose Fernandez-Triana; **Event:** verbatimEventDate: 19-Jul-1976; **Record Level:** institutionID: Canadian National Collection of Insects, Arachnids and Nematodes
- **Type status:** Other material. **Occurrence:** catalogNumber: BIOUG11904-C07; recordedBy: F.Tremblay; lifeStage: Adult; otherCatalogNumbers: CNFNR2271-14; **Location:** country: Canada; stateProvince: Quebec; county: Forillon National Park; municipality: trail off of park compound (i.e. operation centre), 1501 boul. Forillon; locality: mixed forest (spruce, poplar); verbatimElevation: 135; verbatimLatitude: 48.857; verbatimLongitude: -64.376; **Identification:** identifiedBy: Angela Telfer; **Event:** verbatimEventDate: 15-Jul-2013; **Record Level:** institutionID: Biodiversity Institute of Ontario
- **Type status:** Other material. **Occurrence:** catalogNumber: BIOUG00991-B06; recordedBy: Alex Smith; lifeStage: Adult; otherCatalogNumbers: ASGLF366-11; **Location:** country: Canada; stateProvince: Ontario; county: Haldimond-Dunn Townline; municipality: Windy Bluff; verbatimElevation: 179; verbatimLatitude: 42.861; verbatimLongitude: -79.703; **Identification:** identifiedBy: Angela Telfer; **Event:** verbatimEventDate: 12-Sep-2010; **Record Level:** institutionID: Research Collection of M. Alex Smith
- **Type status:** Other material. **Occurrence:** catalogNumber: BIOUG09238-D12; recordedBy: BIOBus 2012; lifeStage: Adult; otherCatalogNumbers: SSPAC13669-13; **Location:** country: Canada; stateProvince: Saskatchewan; county: Prince Albert NP; municipality: Spruce River Highlands Trail; verbatimElevation: 542; verbatimLatitude: 53.713; verbatimLongitude: -106.048; **Event:** verbatimEventDate: 14-Jul-2012; **Record Level:** institutionID: Biodiversity Institute of Ontario
- **Type status:** Other material. **Occurrence:** catalogNumber: BIOUG12038-B01; recordedBy: F.Tremblay; lifeStage: Adult; otherCatalogNumbers: CNFNS943-14; **Location:** country: Canada; stateProvince: Quebec; county: Forillon National Park; municipality: trail off of park compound (i.e. operation centre), 1501 boul. Forillon; locality: mixed forest (spruce, poplar); verbatimElevation: 135; verbatimLatitude: 48.857; verbatimLongitude: -64.376; **Identification:** identifiedBy: Angela Telfer; **Event:** verbatimEventDate: 30-Jul-2013; **Record Level:** institutionID: Biodiversity Institute of Ontario
- **Type status:** Other material. **Occurrence:** catalogNumber: BIOUG00989-A07; recordedBy: Alex Smith; lifeStage: Adult; otherCatalogNumbers: ASGLF260-11; **Location:** country: Canada; stateProvince: Ontario; county: Guelph; municipality: 25 Division St.; verbatimElevation: 326; verbatimLatitude: 43.554; verbatimLongitude: -80.264; **Identification:** identifiedBy: Angela Telfer; **Event:** verbatimEventDate: 29-Oct-2010; **Record Level:** institutionID: Research Collection of M. Alex Smith
- **Type status:** Other material. **Occurrence:** catalogNumber: BIOUG10479-G03; recordedBy: F.Tremblay; lifeStage: Adult; otherCatalogNumbers: CNFNF2105-14; **Location:** country: Canada; stateProvince: Quebec; county: Forillon National Park; municipality: trail off of park compound (i.e. operation centre), 1501 boul. Forillon; locality: mixed forest (spruce, poplar); verbatimElevation: 135; verbatimLatitude: 48.857; verbatimLongitude: -64.376; **Event:** verbatimEventDate: 18-Jul-2013; **Record Level:** institutionID: Biodiversity Institute of Ontario
- **Type status:** Other material. **Occurrence:** catalogNumber: BIOUG11908-G11; recordedBy: F.Tremblay; lifeStage: Adult; otherCatalogNumbers: CNFNR2703-14; **Location:** country: Canada; stateProvince: Quebec; county: Forillon National Park; municipality: trail off of park compound (i.e. operation centre), 1501 boul. Forillon; locality: mixed forest (spruce, poplar); verbatimElevation: 135; verbatimLatitude: 48.857; verbatimLongitude: -64.376; **Identification:** identifiedBy: Angela Telfer; **Event:** verbatimEventDate: 15-Jul-2013; **Record Level:** institutionID: Biodiversity Institute of Ontario
- **Type status:** Other material. **Occurrence:** catalogNumber: BIOUG03486-E11; recordedBy: Jarret Hardisty; lifeStage: Adult; otherCatalogNumbers: CNWLF272-12; **Location:** country: Canada; stateProvince: Alberta; county: Waterton Lakes NP; municipality: Foothills Parkland Region; verbatimElevation: 1338; verbatimLatitude: 49.083; verbatimLongitude: -113.876; **Event:** verbatimEventDate: 26-Jul-2012; **Record Level:** institutionID: Biodiversity Institute of Ontario
- **Type status:** Other material. **Occurrence:** catalogNumber: WMIC 0240; recordedBy: M. Sharkey; lifeStage: Adult; otherCatalogNumbers: CNCHX240-09; **Location:** country: Canada; stateProvince: Prince Edward Island; county: Georgetown; locality: near Georgetown; verbatimElevation: 8; verbatimLatitude: 46.417; verbatimLongitude: -62.667; **Identification:** identifiedBy: Jose Fernandez-Triana; **Event:** verbatimEventDate: 31-Jul-2005; **Record Level:** institutionID: Canadian National Collection of Insects, Arachnids and Nematodes
- **Type status:** Other material. **Occurrence:** catalogNumber: BIOUG04245-B04; recordedBy: Jarret Hardisty; lifeStage: Adult; otherCatalogNumbers: CNWLF646-12; **Location:** country: Canada; stateProvince: Alberta; county: Waterton Lakes NP; municipality: Foothills Parkland Region; verbatimElevation: 1338; verbatimLatitude: 49.083; verbatimLongitude: -113.876; **Event:** verbatimEventDate: 26-Jul-2012; **Record Level:** institutionID: Biodiversity Institute of Ontario
- **Type status:** Other material. **Occurrence:** catalogNumber: MIC 000062; sex: Female; lifeStage: Adult; otherCatalogNumbers: MHABR062-09; **Location:** country: United States; stateProvince: North Carolina; county: Avery County; locality: Linville; **Identification:** identifiedBy: Jose Fernandez-Triana; **Event:** verbatimEventDate: 26-Aug-1976; **Record Level:** institutionID: Canadian National Collection of Insects, Arachnids and Nematodes
- **Type status:** Other material. **Occurrence:** catalogNumber: BIOUG11907-B04; recordedBy: F.Tremblay; lifeStage: Adult; otherCatalogNumbers: CNFNR2541-14; **Location:** country: Canada; stateProvince: Quebec; county: Forillon National Park; municipality: trail off of park compound (i.e. operation centre), 1501 boul. Forillon; locality: mixed forest (spruce, poplar); verbatimElevation: 135; verbatimLatitude: 48.857; verbatimLongitude: -64.376; **Identification:** identifiedBy: Angela Telfer; **Event:** verbatimEventDate: 15-Jul-2013; **Record Level:** institutionID: Biodiversity Institute of Ontario
- **Type status:** Other material. **Occurrence:** catalogNumber: BIOUG01327-H05; recordedBy: James Sones; lifeStage: Adult; otherCatalogNumbers: JSHYP610-11; **Location:** country: Canada; stateProvince: Ontario; county: Leeds and Grenville; municipality: Elizabethtown-Kitley; locality: 4452 Rowsome Rd., Elizabethtown; verbatimElevation: 112; verbatimLatitude: 44.621; verbatimLongitude: -75.773; **Event:** verbatimEventDate: 04-Oct-2010; **Record Level:** institutionID: Biodiversity Institute of Ontario

##### Notes

This species is widely distributed in central and eastern North America. It was recorded from two provinces in Canada (NS, ON) by Fernandez-Triana (2010), but no locality records were provided at the time. Here we present additional information about the distribution of this species in Canada, including five new provincial records (AB, NL, PE, QC, SK), based on examined specimens from the BIO and CNC collections. Data for those specimens was extracted from BOLD.

The specimens of *A. conanchetorum* that rendered DNA barcodes comprise two BINS: BOLD:AAC5506 (eastern North America) and BOLD:AAC5507 (principally Western Canada, but some records from ON, PE) (Suppl. material 4); whether they represent two different species or not will be studied in an upcoming paper.

#### Apanteles ensiger

(Say, 1836)

##### Materials

- **Type status:** Other material. **Occurrence:** catalogNumber: BIOUG11625-E04; recordedBy: Cavan Harpur; lifeStage: Adult; otherCatalogNumbers: CNPKJ648-14; **Location:** country: Canada; stateProvince: Ontario; county: Heron Bay; municipality: Pukaskwa National Park; verbatimElevation: 631; verbatimLatitude: 48.601; verbatimLongitude: -86.289; **Event:** verbatimEventDate: 26-Aug-2013; **Record Level:** institutionID: Biodiversity Institute of Ontario
- **Type status:** Other material. **Occurrence:** catalogNumber: BIOUG01657-E06; recordedBy: J. Cossey; lifeStage: Adult; otherCatalogNumbers: NCCC149-11; **Location:** country: Canada; stateProvince: Ontario; municipality: Carden Alvar; locality: North Bear; verbatimLatitude: 44.695; verbatimLongitude: -79.039; **Event:** verbatimEventDate: 06-Oct-2011; **Record Level:** institutionID: Biodiversity Institute of Ontario
- **Type status:** Other material. **Occurrence:** catalogNumber: 07PROBE-22328; recordedBy: A.Renaud; lifeStage: Adult; otherCatalogNumbers: ASWAV869-08; **Location:** country: Canada; stateProvince: Manitoba; county: Churchill; locality: 23 km E Churchill, Ramsay Creek; verbatimElevation: 10; verbatimLatitude: 58.731; verbatimLongitude: -93.780; **Identification:** identifiedBy: Jose Fernandez-Triana; **Event:** verbatimEventDate: 02-Aug-2007; **Record Level:** institutionID: Biodiversity Institute of Ontario
- **Type status:** Other material. **Occurrence:** catalogNumber: BIOUG11759-F05; recordedBy: Matt Davis; lifeStage: Adult; otherCatalogNumbers: CNKJF1743-14; **Location:** country: Canada; stateProvince: Nova Scotia; county: Kejimkujik National Park; locality: Jeremy`s Bay Campground, near Ampitheater off of Campfire Circle; verbatimElevation: 116; verbatimLatitude: 44.407; verbatimLongitude: -65.245; **Event:** verbatimEventDate: 25-Jul-2013; **Record Level:** institutionID: Biodiversity Institute of Ontario
- **Type status:** Other material. **Occurrence:** catalogNumber: CAM0960; recordedBy: H. Goulet, A. Badiss, C. Boudeault; lifeStage: Adult; otherCatalogNumbers: CNCHX090-09; **Location:** country: Canada; stateProvince: Newfoundland and Labrador; county: Plum Point; locality: Plum Point, fallow field; verbatimLatitude: 51.064; verbatimLongitude: -56.982; **Identification:** identifiedBy: Jose Fernandez-Triana; **Event:** verbatimEventDate: 16-Jul-2008; **Record Level:** institutionID: Canadian National Collection of Insects, Arachnids and Nematodes
- **Type status:** Other material. **Occurrence:** catalogNumber: BIOUG14172-C09; recordedBy: Cavan Harpur; lifeStage: Adult; otherCatalogNumbers: CNPKO3073-14; **Location:** country: Canada; stateProvince: Ontario; county: Heron Bay; municipality: Pukaskwa National Park; verbatimElevation: 631; verbatimLatitude: 48.601; verbatimLongitude: -86.289; **Identification:** identifiedBy: Angela Telfer; **Event:** verbatimEventDate: 01-Sep-2013; **Record Level:** institutionID: Biodiversity Institute of Ontario
- **Type status:** Other material. **Occurrence:** catalogNumber: BIOUG01631-D07; recordedBy: James Sones; lifeStage: Adult; otherCatalogNumbers: JSSEP718-11; **Location:** country: Canada; stateProvince: Ontario; county: Leeds and Grenville; municipality: Elizabethtown-Kitley; locality: 4452 Rowsome Rd., Elizabethtown; verbatimElevation: 111; verbatimLatitude: 44.618; verbatimLongitude: -75.775; **Identification:** identifiedBy: BOLD ID Engine; **Event:** verbatimEventDate: 14-Sep-2011; **Record Level:** institutionID: Biodiversity Institute of Ontario
- **Type status:** Other material. **Occurrence:** catalogNumber: BIOUG08683-C10; recordedBy: BIO Collections Staff; lifeStage: Adult; otherCatalogNumbers: SMTPD5208-13; **Location:** country: Canada; stateProvince: Ontario; county: Wellington County; municipality: Guelph; locality: Biodiversity Institute of Ontario; verbatimElevation: 335; verbatimLatitude: 43.528; verbatimLongitude: -80.229; **Event:** verbatimEventDate: 27-Sep-2013; **Record Level:** institutionID: Biodiversity Institute of Ontario
- **Type status:** Other material. **Occurrence:** catalogNumber: BIOUG14172-G04; recordedBy: Cavan Harpur; lifeStage: Adult; otherCatalogNumbers: CNPKO3116-14; **Location:** country: Canada; stateProvince: Ontario; county: Heron Bay; municipality: Pukaskwa National Park; verbatimElevation: 631; verbatimLatitude: 48.601; verbatimLongitude: -86.289; **Identification:** identifiedBy: Angela Telfer; **Event:** verbatimEventDate: 01-Sep-2013; **Record Level:** institutionID: Biodiversity Institute of Ontario
- **Type status:** Other material. **Occurrence:** catalogNumber: CAM0009; recordedBy: H. Goulet; lifeStage: Adult; otherCatalogNumbers: CNCHZ199-09; **Location:** country: Canada; stateProvince: Ontario; county: Ottawa; locality: Ottawa city garden; verbatimLatitude: 45.356; verbatimLongitude: -75.707; **Identification:** identifiedBy: Jose Fernandez-Triana; **Event:** verbatimEventDate: 19-Sep-2007; **Record Level:** institutionID: Canadian National Collection of Insects, Arachnids and Nematodes
- **Type status:** Other material. **Occurrence:** catalogNumber: BIOUG08669-D07; recordedBy: BIO Collections Staff; lifeStage: Adult; otherCatalogNumbers: MBIOD945-13; **Location:** country: Canada; stateProvince: Ontario; county: Wellington County; municipality: Guelph; locality: Biodiversity Institute of Ontario; verbatimElevation: 335; verbatimLatitude: 43.528; verbatimLongitude: -80.229; **Event:** verbatimEventDate: 13-Jun-2013; **Record Level:** institutionID: Biodiversity Institute of Ontario
- **Type status:** Other material. **Occurrence:** catalogNumber: CAM0940; recordedBy: H. Goulet, A. Badiss, C. Boudeault; lifeStage: Adult; otherCatalogNumbers: CNCHX070-09; **Location:** country: Canada; stateProvince: Newfoundland and Labrador; county: Corner Brook; locality: Corner Brook, fallow field; verbatimLatitude: 48.956; verbatimLongitude: -57.911; **Identification:** identifiedBy: Jose Fernandez-Triana; **Event:** verbatimEventDate: 17-Jul-2008; **Record Level:** institutionID: Canadian National Collection of Insects, Arachnids and Nematodes
- **Type status:** Other material. **Occurrence:** catalogNumber: MIC 000087; sex: Female; lifeStage: Adult; otherCatalogNumbers: MHABR087-09; **Location:** country: Canada; stateProvince: Ontario; county: Rondeau Provincial Park; **Identification:** identifiedBy: Jose Fernandez-Triana; **Event:** verbatimEventDate: 16-Jul-1972; **Record Level:** institutionID: Canadian National Collection of Insects, Arachnids and Nematodes
- **Type status:** Other material. **Occurrence:** catalogNumber: BIOUG08641-D09; recordedBy: M.Neerhof, S.Lipskie; lifeStage: Adult; otherCatalogNumbers: SMTPD3239-13; **Location:** country: Canada; stateProvince: Ontario; county: Guelph; municipality: John F. Ross Collegiate & Vocational Institute; locality: EQP-CLL-524; verbatimElevation: 329; verbatimLatitude: 43.562; verbatimLongitude: -80.247; **Event:** verbatimEventDate: 27-Sep-2013; **Record Level:** institutionID: Biodiversity Institute of Ontario
- **Type status:** Other material. **Occurrence:** catalogNumber: BIOUG08683-G07; recordedBy: BIO Collections Staff; lifeStage: Adult; otherCatalogNumbers: SMTPD5253-13; **Location:** country: Canada; stateProvince: Ontario; county: Wellington County; municipality: Guelph; locality: Biodiversity Institute of Ontario; verbatimElevation: 335; verbatimLatitude: 43.528; verbatimLongitude: -80.229; **Event:** verbatimEventDate: 27-Sep-2013; **Record Level:** institutionID: Biodiversity Institute of Ontario
- **Type status:** Other material. **Occurrence:** catalogNumber: BIOUG08663-H08; recordedBy: Anne Parkhill; lifeStage: Adult; otherCatalogNumbers: SMTPD4869-13; **Location:** country: Canada; stateProvince: Ontario; county: Paris; municipality: Holy Family School; locality: EQP-CLL-560; verbatimElevation: 250; verbatimLatitude: 43.207; verbatimLongitude: -80.391; **Event:** verbatimEventDate: 27-Sep-2013; **Record Level:** institutionID: Biodiversity Institute of Ontario
- **Type status:** Other material. **Occurrence:** catalogNumber: BIOUG09765-A01; recordedBy: Lydia Attard, Kyla Greenham; lifeStage: Adult; otherCatalogNumbers: CNROS470-13; **Location:** country: Canada; stateProvince: Ontario; county: Toronto; municipality: Rouge National Urban Park, Trap 1; verbatimElevation: 136; verbatimLatitude: 43.822; verbatimLongitude: -79.190; **Event:** verbatimEventDate: 27-Aug-2013; **Record Level:** institutionID: Biodiversity Institute of Ontario
- **Type status:** Other material. **Occurrence:** catalogNumber: BIOUG08683-C04; recordedBy: BIO Collections Staff; lifeStage: Adult; otherCatalogNumbers: SMTPD5202-13; **Location:** country: Canada; stateProvince: Ontario; county: Wellington County; municipality: Guelph; locality: Biodiversity Institute of Ontario; verbatimElevation: 335; verbatimLatitude: 43.528; verbatimLongitude: -80.229; **Event:** verbatimEventDate: 27-Sep-2013; **Record Level:** institutionID: Biodiversity Institute of Ontario
- **Type status:** Other material. **Occurrence:** catalogNumber: BIOUG14108-F04; recordedBy: Cavan Harpur; lifeStage: Adult; otherCatalogNumbers: CNPKO1489-14; **Location:** country: Canada; stateProvince: Ontario; county: Heron Bay; municipality: Pukaskwa National Park; verbatimElevation: 631; verbatimLatitude: 48.601; verbatimLongitude: -86.289; **Identification:** identifiedBy: Angela Telfer; **Event:** verbatimEventDate: 01-Sep-2013; **Record Level:** institutionID: Biodiversity Institute of Ontario
- **Type status:** Other material. **Occurrence:** catalogNumber: BIOUG08647-G04; recordedBy: Lisa Cairncross; lifeStage: Adult; otherCatalogNumbers: SMTPD3828-13; **Location:** country: Canada; stateProvince: Ontario; county: St. Marys; municipality: Little Falls Public School; locality: EQP-CLL-554; verbatimElevation: 305; verbatimLatitude: 43.259; verbatimLongitude: -81.142; **Event:** verbatimEventDate: 27-Sep-2013; **Record Level:** institutionID: Biodiversity Institute of Ontario
- **Type status:** Other material. **Occurrence:** catalogNumber: BIOUG09932-C07; recordedBy: BIO Collections Staff; lifeStage: Adult; otherCatalogNumbers: MBION508-14; **Location:** country: Canada; stateProvince: Ontario; county: Wellington County; municipality: Guelph; locality: Biodiversity Institute of Ontario; verbatimElevation: 335; verbatimLatitude: 43.528; verbatimLongitude: -80.229; **Event:** verbatimEventDate: 04-Oct-2013; **Record Level:** institutionID: Biodiversity Institute of Ontario
- **Type status:** Other material. **Occurrence:** catalogNumber: HYM00001023; recordedBy: Goulet & Boudreault; lifeStage: Adult; otherCatalogNumbers: ASWA309-08; **Location:** country: Canada; stateProvince: Manitoba; locality: Gardenton; verbatimElevation: 292; verbatimLatitude: 49.079; verbatimLongitude: -96.766; **Identification:** identifiedBy: Jose Fernandez-Triana; **Event:** verbatimEventDate: 11-Jun-2007; **Record Level:** institutionID: Canadian National Collection of Insects, Arachnids and Nematodes
- **Type status:** Other material. **Occurrence:** catalogNumber: BIOUG10202-A04; recordedBy: Chris Johnstone; lifeStage: Adult; otherCatalogNumbers: CNGBG1411-14; **Location:** country: Canada; stateProvince: Ontario; county: Georgian Bay Islands National Park; municipality: Beausoleil Island; locality: Cedar Spring Campground, mixed forest; verbatimElevation: 177; verbatimLatitude: 44.852; verbatimLongitude: -79.874; **Event:** verbatimEventDate: 27-Aug-2013; **Record Level:** institutionID: Biodiversity Institute of Ontario
- **Type status:** Other material. **Occurrence:** catalogNumber: CAM0342; recordedBy: Goulet & Fernandez; lifeStage: Adult; otherCatalogNumbers: CNCHZ532-09; **Location:** country: Canada; stateProvince: Ontario; county: Almonte; locality: 5 km NW of Almonte, Hwy 49, Burnt Land, Alvar Prov. Park; verbatimLatitude: 45.255; verbatimLongitude: -76.140; **Identification:** identifiedBy: Jose Fernandez-Triana; **Event:** verbatimEventDate: 29-May-2008; **Record Level:** institutionID: Canadian National Collection of Insects, Arachnids and Nematodes
- **Type status:** Other material. **Occurrence:** catalogNumber: MIC 000903; recordedBy: H. Goulet, A. Badiss, C. Boudeault; lifeStage: Adult; otherCatalogNumbers: CNCHZ1095-09; **Location:** country: Canada; stateProvince: Newfoundland and Labrador; locality: Corner Brook, fallow field; verbatimLatitude: 48.956; verbatimLongitude: -57.911; **Identification:** identifiedBy: Jose Fernandez-Triana; **Event:** verbatimEventDate: 17-Jul-2008; **Record Level:** institutionID: Canadian National Collection of Insects, Arachnids and Nematodes
- **Type status:** Other material. **Occurrence:** catalogNumber: BIOUG11707-A06; recordedBy: Cavan Harpur; lifeStage: Adult; otherCatalogNumbers: CNPKJ2122-14; **Location:** country: Canada; stateProvince: Ontario; county: Heron Bay; municipality: Pukaskwa National Park; verbatimElevation: 631; verbatimLatitude: 48.601; verbatimLongitude: -86.289; **Event:** verbatimEventDate: 26-Aug-2013; **Record Level:** institutionID: Biodiversity Institute of Ontario
- **Type status:** Other material. **Occurrence:** catalogNumber: HYM00001022; recordedBy: Goulet & Boudreault; lifeStage: Adult; otherCatalogNumbers: ASWA308-08; **Location:** country: Canada; stateProvince: Manitoba; locality: Gardenton; verbatimElevation: 292; verbatimLatitude: 49.079; verbatimLongitude: -96.766; **Identification:** identifiedBy: Jose Fernandez-Triana; **Event:** verbatimEventDate: 11-Jun-2007; **Record Level:** institutionID: Canadian National Collection of Insects, Arachnids and Nematodes
- **Type status:** Other material. **Occurrence:** catalogNumber: BIOUG06823-D09; recordedBy: Thousand Islands NP; lifeStage: Adult; otherCatalogNumbers: CNSLS106-13; **Location:** country: Canada; stateProvince: Ontario; county: Thousand Islands NP; municipality: Jones Creek by Mallorytown, County Rd. 5; verbatimElevation: 117; verbatimLatitude: 44.475; verbatimLongitude: -75.865; **Event:** verbatimEventDate: 12-Aug-2012; **Record Level:** institutionID: Biodiversity Institute of Ontario
- **Type status:** Other material. **Occurrence:** catalogNumber: BIOUG08647-C01; recordedBy: Lisa Cairncross; lifeStage: Adult; otherCatalogNumbers: SMTPD3777-13; **Location:** country: Canada; stateProvince: Ontario; county: St. Marys; municipality: Little Falls Public School; locality: EQP-CLL-554; verbatimElevation: 305; verbatimLatitude: 43.259; verbatimLongitude: -81.142; **Event:** verbatimEventDate: 27-Sep-2013; **Record Level:** institutionID: Biodiversity Institute of Ontario
- **Type status:** Other material. **Occurrence:** catalogNumber: MIC 000910; recordedBy: H. Goulet, A. Badiss, C. Boudeault; lifeStage: Adult; otherCatalogNumbers: CNCHZ1102-09; **Location:** country: Canada; stateProvince: Newfoundland and Labrador; locality: Plum Point, fallow field; verbatimLatitude: 51.064; verbatimLongitude: -56.848; **Identification:** identifiedBy: Jose Fernandez-Triana; **Event:** verbatimEventDate: 16-Jul-2008; **Record Level:** institutionID: Canadian National Collection of Insects, Arachnids and Nematodes
- **Type status:** Other material. **Occurrence:** catalogNumber: CAM0526; recordedBy: A. Bennett; lifeStage: Adult; otherCatalogNumbers: CNCHZ1146-09; **Location:** country: Canada; stateProvince: Ontario; county: Ottawa; locality: Mixed forest near Manotick; verbatimLatitude: 45.235; verbatimLongitude: -75.624; **Identification:** identifiedBy: Jose Fernandez-Triana; **Record Level:** institutionID: Canadian National Collection of Insects, Arachnids and Nematodes
- **Type status:** Other material. **Occurrence:** catalogNumber: CAM0962; recordedBy: H. Goulet, A. Badiss, C. Boudeault; lifeStage: Adult; otherCatalogNumbers: CNCHX092-09; **Location:** country: Canada; stateProvince: Newfoundland and Labrador; county: Corner Brook; locality: Corner Brook, fallow field; verbatimLatitude: 48.956; verbatimLongitude: -57.911; **Identification:** identifiedBy: Jose Fernandez-Triana; **Event:** verbatimEventDate: 17-Jul-2008; **Record Level:** institutionID: Canadian National Collection of Insects, Arachnids and Nematodes
- **Type status:** Other material. **Occurrence:** catalogNumber: MIC 000493; recordedBy: H. Goulet, A. Badiss, C. Boudeault; lifeStage: Adult; otherCatalogNumbers: CNCHZ685-09; **Location:** country: Canada; stateProvince: Prince Edward Island; locality: Blooming Point, fallow field; verbatimElevation: 6; verbatimLatitude: 46.408; verbatimLongitude: -62.951; **Identification:** identifiedBy: Jose Fernandez-Triana; **Event:** verbatimEventDate: 23-Jul-2008; **Record Level:** institutionID: Canadian National Collection of Insects, Arachnids and Nematodes
- **Type status:** Other material. **Occurrence:** catalogNumber: BIOUG06549-D07; recordedBy: Nicole Labine; lifeStage: Adult; otherCatalogNumbers: CNWBH1210-13; **Location:** country: Canada; stateProvince: Alberta; county: Wood Buffalo NP; municipality: Benchmark weather station; verbatimElevation: 219; verbatimLatitude: 59.561; verbatimLongitude: -112.261; **Event:** verbatimEventDate: 05-Aug-2012; **Record Level:** institutionID: Biodiversity Institute of Ontario
- **Type status:** Other material. **Occurrence:** catalogNumber: BIOUG03220-A01; recordedBy: Heidi Brown; lifeStage: Adult; otherCatalogNumbers: CNPPC821-12; **Location:** country: Canada; stateProvince: Ontario; county: Point Pelee NP; municipality: Cactus Field; verbatimElevation: 168; verbatimLatitude: 41.939; verbatimLongitude: -82.516; **Event:** verbatimEventDate: 01-Jun-2012; **Record Level:** institutionID: Biodiversity Institute of Ontario
- **Type status:** Other material. **Occurrence:** catalogNumber: CAM0290; recordedBy: H. Goulet, A. Badiss, C. Boudeault; lifeStage: Adult; otherCatalogNumbers: CNCHZ480-09; **Location:** country: Canada; stateProvince: Quebec; county: Belle-Anse; locality: Belle-Anse; verbatimLatitude: 48.625; verbatimLongitude: -64.178; **Identification:** identifiedBy: Jose Fernandez-Triana; **Event:** verbatimEventDate: 26-Jul-2008; **Record Level:** institutionID: Canadian National Collection of Insects, Arachnids and Nematodes
- **Type status:** Other material. **Occurrence:** catalogNumber: MIC 000637; recordedBy: H. Goulet; lifeStage: Adult; otherCatalogNumbers: CNCHZ829-09; **Location:** country: Canada; stateProvince: Manitoba; county: Aweme; locality: Criddle Homestead, mixed-grass prairie; verbatimLatitude: 49.706; verbatimLongitude: -99.576; **Identification:** identifiedBy: Jose Fernandez-Triana; **Event:** verbatimEventDate: 24-Jul-2007; **Record Level:** institutionID: Canadian National Collection of Insects, Arachnids and Nematodes
- **Type status:** Other material. **Occurrence:** catalogNumber: CAM0011; recordedBy: H. Goulet; lifeStage: Adult; otherCatalogNumbers: CNCHZ201-09; **Location:** country: Canada; stateProvince: Ontario; county: Ottawa; locality: Ottawa city garden; verbatimLatitude: 45.356; verbatimLongitude: -75.707; **Identification:** identifiedBy: Jose Fernandez-Triana; **Event:** verbatimEventDate: 30-May-2007; **Record Level:** institutionID: Canadian National Collection of Insects, Arachnids and Nematodes
- **Type status:** Other material. **Occurrence:** catalogNumber: HYM00001027; recordedBy: Goulet & Boudreault; lifeStage: Adult; otherCatalogNumbers: ASWA313-08; **Location:** country: Canada; stateProvince: Manitoba; locality: Gardenton; verbatimElevation: 292; verbatimLatitude: 49.079; verbatimLongitude: -96.766; **Identification:** identifiedBy: Jose Fernandez-Triana; **Event:** verbatimEventDate: 11-Jun-2007; **Record Level:** institutionID: Canadian National Collection of Insects, Arachnids and Nematodes
- **Type status:** Other material. **Occurrence:** catalogNumber: BIOUG06823-E10; recordedBy: Thousand Islands NP; lifeStage: Adult; otherCatalogNumbers: CNSLS119-13; **Location:** country: Canada; stateProvince: Ontario; county: Thousand Islands NP; municipality: Jones Creek by Mallorytown, County Rd. 5; verbatimElevation: 117; verbatimLatitude: 44.475; verbatimLongitude: -75.865; **Event:** verbatimEventDate: 12-Aug-2012; **Record Level:** institutionID: Biodiversity Institute of Ontario
- **Type status:** Other material. **Occurrence:** catalogNumber: CAM0939; recordedBy: H. Goulet, A. Badiss, C. Boudeault; lifeStage: Adult; otherCatalogNumbers: CNCHX069-09; **Location:** country: Canada; stateProvince: Newfoundland and Labrador; county: Corner Brook; locality: Corner Brook, fallow field; verbatimLatitude: 48.956; verbatimLongitude: -57.911; **Identification:** identifiedBy: Jose Fernandez-Triana; **Event:** verbatimEventDate: 17-Jul-2008; **Record Level:** institutionID: Canadian National Collection of Insects, Arachnids and Nematodes
- **Type status:** Other material. **Occurrence:** catalogNumber: BIOUG11771-G06; recordedBy: Cavan Harpur; lifeStage: Adult; otherCatalogNumbers: CNPKJ2479-14; **Location:** country: Canada; stateProvince: Ontario; county: Heron Bay; municipality: Pukaskwa National Park; verbatimElevation: 631; verbatimLatitude: 48.601; verbatimLongitude: -86.289; **Event:** verbatimEventDate: 26-Aug-2013; **Record Level:** institutionID: Biodiversity Institute of Ontario
- **Type status:** Other material. **Occurrence:** catalogNumber: BIOUG08748-E07; recordedBy: BIO Collections Staff; lifeStage: Adult; otherCatalogNumbers: MBIOE1290-13; **Location:** country: Canada; stateProvince: Ontario; county: Wellington County; municipality: Guelph; locality: Biodiversity Institute of Ontario; verbatimElevation: 335; verbatimLatitude: 43.528; verbatimLongitude: -80.229; **Event:** verbatimEventDate: 27-Jun-2013; **Record Level:** institutionID: Biodiversity Institute of Ontario
- **Type status:** Other material. **Occurrence:** catalogNumber: CAM0352; recordedBy: Goulet & Fernandez; lifeStage: Adult; otherCatalogNumbers: CNCHZ542-09; **Location:** country: Canada; stateProvince: Ontario; county: Almonte; locality: 5 km NW of Almonte, Hwy 49, Burnt Land, Alvar Prov. Park; verbatimLatitude: 45.255; verbatimLongitude: -76.140; **Identification:** identifiedBy: Jose Fernandez-Triana; **Event:** verbatimEventDate: 29-May-2008; **Record Level:** institutionID: Canadian National Collection of Insects, Arachnids and Nematodes
- **Type status:** Other material. **Occurrence:** catalogNumber: CAM0448; recordedBy: S. Peck; lifeStage: Adult; otherCatalogNumbers: CNCHZ638-09; **Location:** country: Canada; stateProvince: Ontario; county: Leeds; locality: Leeds-Granville Co., forest; verbatimLatitude: 44.629; verbatimLongitude: -76.359; **Identification:** identifiedBy: Jose Fernandez-Triana; **Event:** verbatimEventDate: 01-Oct-2008; **Record Level:** institutionID: Canadian National Collection of Insects, Arachnids and Nematodes
- **Type status:** Other material. **Occurrence:** catalogNumber: BIOUG09807-F11; recordedBy: BIO Collections Staff; lifeStage: Adult; otherCatalogNumbers: MBIOK461-14; **Location:** country: Canada; stateProvince: Ontario; county: Wellington County; municipality: Guelph; locality: Biodiversity Institute of Ontario; verbatimElevation: 335; verbatimLatitude: 43.528; verbatimLongitude: -80.229; **Event:** verbatimEventDate: 19-Sep-2013; **Record Level:** institutionID: Biodiversity Institute of Ontario
- **Type status:** Other material. **Occurrence:** catalogNumber: BIOUG06471-C10; recordedBy: BIOBus 2012; lifeStage: Adult; otherCatalogNumbers: SSBAF2145-13; **Location:** country: Canada; stateProvince: Alberta; county: Banff NP; municipality: Corral Creek old road; verbatimElevation: 1539; verbatimLatitude: 51.407; verbatimLongitude: -116.154; **Event:** verbatimEventDate: 24-Jul-2012; **Record Level:** institutionID: Biodiversity Institute of Ontario
- **Type status:** Other material. **Occurrence:** catalogNumber: CNCH3194; recordedBy: Goulet & Bouderault; lifeStage: Adult; otherCatalogNumbers: CNCHV264-10; **Location:** country: United States; stateProvince: Alaska; locality: Trapper Creek, Petersville Road; verbatimElevation: 127; verbatimLatitude: 62.316; verbatimLongitude: -150.261; **Identification:** identifiedBy: Jose Fernandez-Triana; **Event:** verbatimEventDate: 17-Jul-2009; **Record Level:** institutionID: Canadian National Collection of Insects, Arachnids and Nematodes
- **Type status:** Other material. **Occurrence:** catalogNumber: BIOUG03220-F05; recordedBy: Heidi Brown; lifeStage: Adult; otherCatalogNumbers: CNPPC885-12; **Location:** country: Canada; stateProvince: Ontario; county: Point Pelee NP; municipality: Cactus Field; verbatimElevation: 168; verbatimLatitude: 41.939; verbatimLongitude: -82.516; **Event:** verbatimEventDate: 01-Jun-2012; **Record Level:** institutionID: Biodiversity Institute of Ontario
- **Type status:** Other material. **Occurrence:** catalogNumber: CAM0289; recordedBy: H. Goulet, A. Badiss, C. Boudeault; lifeStage: Adult; otherCatalogNumbers: CNCHZ479-09; **Location:** country: Canada; stateProvince: Quebec; county: Belle-Anse; locality: Belle-Anse; verbatimLatitude: 48.625; verbatimLongitude: -64.178; **Identification:** identifiedBy: Jose Fernandez-Triana; **Event:** verbatimEventDate: 26-Jul-2008; **Record Level:** institutionID: Canadian National Collection of Insects, Arachnids and Nematodes
- **Type status:** Other material. **Occurrence:** catalogNumber: BIOUG01657-F10; recordedBy: J. Cossey; lifeStage: Adult; otherCatalogNumbers: NCCC165-11; **Location:** country: Canada; stateProvince: Ontario; municipality: Carden Alvar; locality: North Bear; verbatimLatitude: 44.695; verbatimLongitude: -79.039; **Event:** verbatimEventDate: 06-Oct-2011; **Record Level:** institutionID: Biodiversity Institute of Ontario
- **Type status:** Other material. **Occurrence:** catalogNumber: CAM0344; recordedBy: Goulet & Fernandez; lifeStage: Adult; otherCatalogNumbers: CNCHZ534-09; **Location:** country: Canada; stateProvince: Ontario; county: Almonte; locality: 5 km NW of Almonte, Hwy 49, Burnt Land, Alvar Prov. Park; verbatimLatitude: 45.255; verbatimLongitude: -76.140; **Identification:** identifiedBy: Jose Fernandez-Triana; **Event:** verbatimEventDate: 29-May-2008; **Record Level:** institutionID: Canadian National Collection of Insects, Arachnids and Nematodes
- **Type status:** Other material. **Occurrence:** catalogNumber: HYM00001003; recordedBy: Bennet & Barnes; lifeStage: Adult; otherCatalogNumbers: ASWA289-08; **Location:** country: Canada; stateProvince: Ontario; locality: North Gower to Smith Falls, 1 km N of Rd 6 & Montague Bdy Rd; verbatimLatitude: 45.033; verbatimLongitude: -75.900; **Identification:** identifiedBy: Jose Fernandez-Triana; **Event:** verbatimEventDate: 15-Jun-2004; **Record Level:** institutionID: Canadian National Collection of Insects, Arachnids and Nematodes
- **Type status:** Other material. **Occurrence:** catalogNumber: CAM0525; recordedBy: A. Bennett; lifeStage: Adult; otherCatalogNumbers: CNCHZ1145-09; **Location:** country: Canada; stateProvince: Ontario; county: Ottawa; locality: Mixed forest near Manotick; verbatimLatitude: 45.235; verbatimLongitude: -75.624; **Identification:** identifiedBy: Jose Fernandez-Triana; **Record Level:** institutionID: Canadian National Collection of Insects, Arachnids and Nematodes
- **Type status:** Other material. **Occurrence:** catalogNumber: BIOUG08643-F01; recordedBy: Linda Phillips; lifeStage: Adult; otherCatalogNumbers: SMTPD3455-13; **Location:** country: Canada; stateProvince: Ontario; county: Whitby; municipality: Donald A. Wilson Secondary School; locality: EQP-CLL-597; verbatimElevation: 91; verbatimLatitude: 43.894; verbatimLongitude: -78.964; **Event:** verbatimEventDate: 27-Sep-2013; **Record Level:** institutionID: Biodiversity Institute of Ontario
- **Type status:** Other material. **Occurrence:** catalogNumber: MIC 000090; recordedBy: L. Masner; sex: Female; lifeStage: Adult; otherCatalogNumbers: MHABR090-09; **Location:** country: Canada; stateProvince: Quebec; locality: Gatineau Park; **Identification:** identifiedBy: Jose Fernandez-Triana; **Event:** verbatimEventDate: 15-Jun-1977; **Record Level:** institutionID: Canadian National Collection of Insects, Arachnids and Nematodes
- **Type status:** Other material. **Occurrence:** catalogNumber: BIOUG00761-A09; recordedBy: Brandon Laforest; lifeStage: Adult; otherCatalogNumbers: JBHCI617-11; **Location:** country: Canada; stateProvince: Manitoba; county: Churchill; municipality: 10 km ESE Churchill, Launch Road near A-frame; locality: Launch road Fen; verbatimLatitude: 58.754; verbatimLongitude: -93.998; **Identification:** identifiedBy: Jose Fernandez-Triana; **Event:** verbatimEventDate: 31-Jul-2010; **Record Level:** institutionID: Biodiversity Institute of Ontario
- **Type status:** Other material. **Occurrence:** catalogNumber: HYM00000665.2; lifeStage: Adult; otherCatalogNumbers: ASWAY519-08; **Location:** country: Canada; stateProvince: Manitoba; county: Churchill; locality: unspecified locality; verbatimLatitude: 58.740; verbatimLongitude: -93.820; **Identification:** identifiedBy: Jose Fernandez-Triana; **Event:** verbatimEventDate: 17-Aug-2006; **Record Level:** institutionID: Canadian National Collection of Insects, Arachnids and Nematodes
- **Type status:** Other material. **Occurrence:** catalogNumber: BIOUG09925-E09; recordedBy: BIO Collections Staff; lifeStage: Adult; otherCatalogNumbers: MBIOL366-14; **Location:** country: Canada; stateProvince: Ontario; county: Wellington County; municipality: Guelph; locality: Biodiversity Institute of Ontario; verbatimElevation: 335; verbatimLatitude: 43.528; verbatimLongitude: -80.229; **Event:** verbatimEventDate: 27-Sep-2013; **Record Level:** institutionID: Biodiversity Institute of Ontario
- **Type status:** Other material. **Occurrence:** catalogNumber: MIC 000084; recordedBy: L. Milne; sex: Female; lifeStage: Adult; otherCatalogNumbers: MHABR084-09; **Location:** country: Canada; stateProvince: Quebec; locality: Knowlton; **Identification:** identifiedBy: Jose Fernandez-Triana; **Event:** verbatimEventDate: 24-Aug-1929; **Record Level:** institutionID: Canadian National Collection of Insects, Arachnids and Nematodes
- **Type status:** Other material. **Occurrence:** catalogNumber: BIOUG01572-H03; recordedBy: J. Cossey; lifeStage: Adult; otherCatalogNumbers: NCCC087-11; **Location:** country: Canada; stateProvince: Ontario; municipality: Carden Alvar; locality: Prairie Smoke; verbatimLatitude: 44.645; verbatimLongitude: -79.095; **Event:** verbatimEventDate: 06-Oct-2011; **Record Level:** institutionID: Biodiversity Institute of Ontario
- **Type status:** Other material. **Occurrence:** catalogNumber: CAM0114; recordedBy: H. Goulet; lifeStage: Adult; otherCatalogNumbers: CNCHZ304-09; **Location:** country: Canada; stateProvince: Ontario; county: Ottawa; locality: Ottawa city garden; verbatimLatitude: 45.356; verbatimLongitude: -75.707; **Identification:** identifiedBy: Jose Fernandez-Triana; **Event:** verbatimEventDate: 10-Aug-2007; **Record Level:** institutionID: Canadian National Collection of Insects, Arachnids and Nematodes
- **Type status:** Other material. **Occurrence:** catalogNumber: BIOUG14107-D02; recordedBy: Cavan Harpur; lifeStage: Adult; otherCatalogNumbers: CNPKO1368-14; **Location:** country: Canada; stateProvince: Ontario; county: Heron Bay; municipality: Pukaskwa National Park; verbatimElevation: 631; verbatimLatitude: 48.601; verbatimLongitude: -86.289; **Identification:** identifiedBy: Angela Telfer; **Event:** verbatimEventDate: 01-Sep-2013; **Record Level:** institutionID: Biodiversity Institute of Ontario
- **Type status:** Other material. **Occurrence:** catalogNumber: CAM0345; recordedBy: Goulet & Fernandez; lifeStage: Adult; otherCatalogNumbers: CNCHZ535-09; **Location:** country: Canada; stateProvince: Ontario; county: Almonte; locality: 5 km NW of Almonte, Hwy 49, Burnt Land, Alvar Prov. Park; verbatimLatitude: 45.255; verbatimLongitude: -76.140; **Identification:** identifiedBy: Jose Fernandez-Triana; **Event:** verbatimEventDate: 29-May-2008; **Record Level:** institutionID: Canadian National Collection of Insects, Arachnids and Nematodes
- **Type status:** Other material. **Occurrence:** catalogNumber: CAM0963; recordedBy: H. Goulet, A. Badiss, C. Boudeault; lifeStage: Adult; otherCatalogNumbers: CNCHX093-09; **Location:** country: Canada; stateProvince: Newfoundland and Labrador; county: St-David`s; locality: St-David`s, fallow field; verbatimLatitude: 48.205; verbatimLongitude: -58.866; **Identification:** identifiedBy: Jose Fernandez-Triana; **Event:** verbatimEventDate: 10-Jul-2008; **Record Level:** institutionID: Canadian National Collection of Insects, Arachnids and Nematodes
- **Type status:** Other material. **Occurrence:** catalogNumber: CAM0288; recordedBy: H. Goulet, A. Badiss, C. Boudeault; lifeStage: Adult; otherCatalogNumbers: CNCHZ478-09; **Location:** country: Canada; stateProvince: Quebec; county: Belle-Anse; locality: Belle-Anse; verbatimLatitude: 48.625; verbatimLongitude: -64.178; **Identification:** identifiedBy: Jose Fernandez-Triana; **Event:** verbatimEventDate: 26-Jul-2008; **Record Level:** institutionID: Canadian National Collection of Insects, Arachnids and Nematodes
- **Type status:** Other material. **Occurrence:** catalogNumber: CAM0341; recordedBy: Goulet & Fernandez; lifeStage: Adult; otherCatalogNumbers: CNCHZ531-09; **Location:** country: Canada; stateProvince: Ontario; county: Almonte; locality: 5 km NW of Almonte, Hwy 49, Burnt Land, Alvar Prov. Park; verbatimLatitude: 45.255; verbatimLongitude: -76.140; **Identification:** identifiedBy: Jose Fernandez-Triana; **Event:** verbatimEventDate: 29-May-2008; **Record Level:** institutionID: Canadian National Collection of Insects, Arachnids and Nematodes
- **Type status:** Other material. **Occurrence:** catalogNumber: CAM0106; recordedBy: H. Goulet; lifeStage: Adult; otherCatalogNumbers: CNCHZ296-09; **Location:** country: Canada; stateProvince: Ontario; county: Ottawa; locality: Ottawa city garden; verbatimLatitude: 45.356; verbatimLongitude: -75.707; **Identification:** identifiedBy: Jose Fernandez-Triana; **Event:** verbatimEventDate: 08-Sep-2007; **Record Level:** institutionID: Canadian National Collection of Insects, Arachnids and Nematodes
- **Type status:** Other material. **Occurrence:** catalogNumber: BIOUG06823-E06; recordedBy: Thousand Islands NP; lifeStage: Adult; otherCatalogNumbers: CNSLS115-13; **Location:** country: Canada; stateProvince: Ontario; county: Thousand Islands NP; municipality: Jones Creek by Mallorytown, County Rd. 5; verbatimElevation: 117; verbatimLatitude: 44.475; verbatimLongitude: -75.865; **Event:** verbatimEventDate: 12-Aug-2012; **Record Level:** institutionID: Biodiversity Institute of Ontario
- **Type status:** Other material. **Occurrence:** catalogNumber: HYM00001019; recordedBy: Goulet & Boudreault; lifeStage: Adult; otherCatalogNumbers: ASWA305-08; **Location:** country: Canada; stateProvince: Manitoba; locality: Gardenton; verbatimElevation: 292; verbatimLatitude: 49.079; verbatimLongitude: -96.766; **Identification:** identifiedBy: Jose Fernandez-Triana; **Event:** verbatimEventDate: 11-Jun-2007; **Record Level:** institutionID: Canadian National Collection of Insects, Arachnids and Nematodes
- **Type status:** Other material. **Occurrence:** catalogNumber: CAM0154; recordedBy: H. Goulet; lifeStage: Adult; otherCatalogNumbers: CNCHZ344-09; **Location:** country: Canada; stateProvince: Ontario; county: Ottawa; locality: Ottawa city garden; verbatimLatitude: 45.356; verbatimLongitude: -75.707; **Identification:** identifiedBy: Jose Fernandez-Triana; **Event:** verbatimEventDate: 01-Sep-2007; **Record Level:** institutionID: Canadian National Collection of Insects, Arachnids and Nematodes
- **Type status:** Other material. **Occurrence:** catalogNumber: CAM0340; recordedBy: Goulet & Fernandez; lifeStage: Adult; otherCatalogNumbers: CNCHZ530-09; **Location:** country: Canada; stateProvince: Ontario; county: Almonte; locality: 5 km NW of Almonte, Hwy 49, Burnt Land, Alvar Prov. Park; verbatimLatitude: 45.255; verbatimLongitude: -76.140; **Identification:** identifiedBy: Jose Fernandez-Triana; **Event:** verbatimEventDate: 29-May-2008; **Record Level:** institutionID: Canadian National Collection of Insects, Arachnids and Nematodes
- **Type status:** Other material. **Occurrence:** catalogNumber: HYM00001028; recordedBy: Goulet & Boudreault; lifeStage: Adult; otherCatalogNumbers: ASWA314-08; **Location:** country: Canada; stateProvince: Manitoba; locality: Vita Tallgrass prairie; verbatimLatitude: 49.147; verbatimLongitude: -96.681; **Identification:** identifiedBy: Jose Fernandez-Triana; **Event:** verbatimEventDate: 11-Jun-2007; **Record Level:** institutionID: Canadian National Collection of Insects, Arachnids and Nematodes
- **Type status:** Other material. **Occurrence:** catalogNumber: HYM00001024; recordedBy: Goulet & Boudreault; lifeStage: Adult; otherCatalogNumbers: ASWA310-08; **Location:** country: Canada; stateProvince: Manitoba; locality: Gardenton; verbatimElevation: 292; verbatimLatitude: 49.079; verbatimLongitude: -96.766; **Identification:** identifiedBy: Jose Fernandez-Triana; **Event:** verbatimEventDate: 11-Jun-2007; **Record Level:** institutionID: Canadian National Collection of Insects, Arachnids and Nematodes
- **Type status:** Other material. **Occurrence:** catalogNumber: CAM0161; recordedBy: H. Goulet; lifeStage: Adult; otherCatalogNumbers: CNCHZ351-09; **Location:** country: Canada; stateProvince: Ontario; county: Ottawa; locality: Ottawa city garden; verbatimLatitude: 45.356; verbatimLongitude: -75.707; **Identification:** identifiedBy: Jose Fernandez-Triana; **Event:** verbatimEventDate: 01-Sep-2007; **Record Level:** institutionID: Canadian National Collection of Insects, Arachnids and Nematodes
- **Type status:** Other material. **Occurrence:** catalogNumber: BIOUG12486-D10; recordedBy: Park Staff; lifeStage: Adult; otherCatalogNumbers: CNMIG236-14; **Location:** country: Canada; stateProvince: Quebec; county: Mingan Archipelago National Park Reserve; municipality: Quarry Island; locality: bog near forest edge; verbatimLatitude: 50.214; verbatimLongitude: -63.798; **Identification:** identifiedBy: Angela Telfer; **Event:** verbatimEventDate: 06-Aug-2013; **Record Level:** institutionID: Biodiversity Institute of Ontario
- **Type status:** Other material. **Occurrence:** catalogNumber: PCPP10-0466; recordedBy: T.F.Mitterboeck, C.Vandermeer, V.Junea, C.Sobel; lifeStage: Adult; otherCatalogNumbers: BBHYN072-10; **Location:** country: Canada; stateProvince: Ontario; county: Point Pelee NP; municipality: 15km SE of Leamington; locality: Cactus Patch Meadow off Bike Trail on Route to Visitors Centre; verbatimElevation: 174; verbatimLatitude: 41.934; verbatimLongitude: -82.514; **Identification:** identifiedBy: Julie K. Stahlhut; **Event:** verbatimEventDate: 23-Jun-2010; **Record Level:** institutionID: Biodiversity Institute of Ontario
- **Type status:** Other material. **Occurrence:** catalogNumber: BIOUG12736-B05; recordedBy: J.Garber, M.Davis; lifeStage: Adult; otherCatalogNumbers: CNKJN622-14; **Location:** country: Canada; stateProvince: Nova Scotia; county: Kejimkujik National Park; locality: Jeremy`s Bay Campground, near Ampitheater off of Campfire Circle; verbatimElevation: 116; verbatimLatitude: 44.407; verbatimLongitude: -65.245; **Identification:** identifiedBy: Angela Telfer; **Event:** verbatimEventDate: 18-Jul-2013; **Record Level:** institutionID: Biodiversity Institute of Ontario
- **Type status:** Other material. **Occurrence:** catalogNumber: 10BBHYM-1225; recordedBy: Biobus 2010; lifeStage: Adult; otherCatalogNumbers: BBHYH202-10; **Location:** country: United States; stateProvince: Florida; county: Kissimmee Prairie Preserve SP; locality: mesic hammock; verbatimElevation: 15; verbatimLatitude: 27.582; verbatimLongitude: -81.048; **Identification:** identifiedBy: Jose Fernandez-Triana; **Event:** verbatimEventDate: 07-Mar-2010; **Record Level:** institutionID: Biodiversity Institute of Ontario
- **Type status:** Other material. **Occurrence:** catalogNumber: HYM00001004; recordedBy: Goulet & Boudreault; lifeStage: Adult; otherCatalogNumbers: ASWA290-08; **Location:** country: Canada; stateProvince: Manitoba; municipality: Gardenton; verbatimElevation: 292; verbatimLatitude: 49.079; verbatimLongitude: -96.766; **Identification:** identifiedBy: Jose Fernandez-Triana; **Event:** verbatimEventDate: 11-Jun-2007; **Record Level:** institutionID: Canadian National Collection of Insects, Arachnids and Nematodes
- **Type status:** Other material. **Occurrence:** catalogNumber: CAM0008; recordedBy: H. Goulet; lifeStage: Adult; otherCatalogNumbers: CNCHZ198-09; **Location:** country: Canada; stateProvince: Ontario; county: Ottawa; locality: Ottawa city garden; verbatimLatitude: 45.356; verbatimLongitude: -75.707; **Identification:** identifiedBy: Jose Fernandez-Triana; **Event:** verbatimEventDate: 19-Sep-2007; **Record Level:** institutionID: Canadian National Collection of Insects, Arachnids and Nematodes
- **Type status:** Other material. **Occurrence:** catalogNumber: BIOUG08799-G08; recordedBy: BIO Collections Staff; lifeStage: Adult; otherCatalogNumbers: MBIOE1410-13; **Location:** country: Canada; stateProvince: Ontario; county: Wellington County; municipality: Guelph; locality: Biodiversity Institute of Ontario; verbatimElevation: 335; verbatimLatitude: 43.528; verbatimLongitude: -80.229; **Event:** verbatimEventDate: 27-Jun-2013; **Record Level:** institutionID: Biodiversity Institute of Ontario
- **Type status:** Other material. **Occurrence:** catalogNumber: CAM0521; recordedBy: A. Bennett; lifeStage: Adult; otherCatalogNumbers: CNCHZ1141-09; **Location:** country: Canada; stateProvince: Ontario; county: Ottawa; locality: Mixed forest near Manotick; verbatimLatitude: 45.235; verbatimLongitude: -75.624; **Identification:** identifiedBy: Jose Fernandez-Triana; **Record Level:** institutionID: Canadian National Collection of Insects, Arachnids and Nematodes
- **Type status:** Other material. **Occurrence:** catalogNumber: BIOUG04041-A02; recordedBy: Thousand Islands NP; lifeStage: Adult; otherCatalogNumbers: CNSLK104-12; **Location:** country: Canada; stateProvince: Ontario; county: Thousand Islands NP; municipality: Jones Creek by Mallorytown, County Rd. 5; verbatimElevation: 117; verbatimLatitude: 44.475; verbatimLongitude: -75.865; **Event:** verbatimEventDate: 15-Sep-2012; **Record Level:** institutionID: Biodiversity Institute of Ontario
- **Type status:** Other material. **Occurrence:** catalogNumber: BIOUG03979-A07; recordedBy: Thousand Islands NP; lifeStage: Adult; otherCatalogNumbers: CNSLI393-12; **Location:** country: Canada; stateProvince: Ontario; county: Thousand Islands NP; municipality: Jones Creek by Mallorytown, County Rd. 5; verbatimElevation: 117; verbatimLatitude: 44.475; verbatimLongitude: -75.865; **Event:** verbatimEventDate: 21-Aug-2012; **Record Level:** institutionID: Biodiversity Institute of Ontario
- **Type status:** Other material. **Occurrence:** catalogNumber: BIOUG03979-B04; recordedBy: Thousand Islands NP; lifeStage: Adult; otherCatalogNumbers: CNSLI402-12; **Location:** country: Canada; stateProvince: Ontario; county: Thousand Islands NP; municipality: Jones Creek by Mallorytown, County Rd. 5; verbatimElevation: 117; verbatimLatitude: 44.475; verbatimLongitude: -75.865; **Event:** verbatimEventDate: 21-Aug-2012; **Record Level:** institutionID: Biodiversity Institute of Ontario
- **Type status:** Other material. **Occurrence:** catalogNumber: BIOUG00991-B03; recordedBy: Alex Smith; lifeStage: Adult; otherCatalogNumbers: ASGLF363-11; **Location:** country: Canada; stateProvince: Ontario; county: Haldimond-Dunn Townline; municipality: Windy Bluff; verbatimElevation: 179; verbatimLatitude: 42.861; verbatimLongitude: -79.703; **Identification:** identifiedBy: Jose Fernandez-Triana; **Event:** verbatimEventDate: 01-Aug-2010; **Record Level:** institutionID: Research Collection of M. Alex Smith
- **Type status:** Other material. **Occurrence:** catalogNumber: CAM0166; recordedBy: H. Goulet; lifeStage: Adult; otherCatalogNumbers: CNCHZ356-09; **Location:** country: Canada; stateProvince: Ontario; county: Ottawa; locality: Ottawa city garden; verbatimLatitude: 45.356; verbatimLongitude: -75.707; **Identification:** identifiedBy: Jose Fernandez-Triana; **Event:** verbatimEventDate: 01-Sep-2007; **Record Level:** institutionID: Canadian National Collection of Insects, Arachnids and Nematodes
- **Type status:** Other material. **Occurrence:** catalogNumber: CAM0103; recordedBy: H. Goulet; lifeStage: Adult; otherCatalogNumbers: CNCHZ293-09; **Location:** country: Canada; stateProvince: Ontario; county: Ottawa; locality: Ottawa city garden; verbatimLatitude: 45.356; verbatimLongitude: -75.707; **Identification:** identifiedBy: Jose Fernandez-Triana; **Event:** verbatimEventDate: 08-Sep-2007; **Record Level:** institutionID: Canadian National Collection of Insects, Arachnids and Nematodes
- **Type status:** Other material. **Occurrence:** catalogNumber: BIOUG07898-C04; recordedBy: BIO Team; lifeStage: Adult; otherCatalogNumbers: RBINA1006-13; **Location:** country: Canada; stateProvince: Ontario; county: Rouge National Urban Park; locality: Sector 7; verbatimElevation: 135; verbatimLatitude: 43.825; verbatimLongitude: -79.203; **Event:** verbatimEventDate: 15-Sep-2013; **Record Level:** institutionID: Biodiversity Institute of Ontario
- **Type status:** Other material. **Occurrence:** catalogNumber: BIOUG09931-A07; recordedBy: BIO Collections Staff; lifeStage: Adult; otherCatalogNumbers: MBION389-14; **Location:** country: Canada; stateProvince: Ontario; county: Wellington County; municipality: Guelph; locality: Biodiversity Institute of Ontario; verbatimElevation: 335; verbatimLatitude: 43.528; verbatimLongitude: -80.229; **Event:** verbatimEventDate: 04-Oct-2013; **Record Level:** institutionID: Biodiversity Institute of Ontario
- **Type status:** Other material. **Occurrence:** catalogNumber: BIOUG12977-D11; recordedBy: Medo Toure; lifeStage: Adult; otherCatalogNumbers: CNLMG1235-14; **Location:** country: Canada; stateProvince: Quebec; county: La Mauricie National Park; municipality: Saint-Mathieu west park entrance near Visitor Centre; locality: primarily conifer forest stand (balsam fir, Epicea glauca, birch); verbatimElevation: 219; verbatimLatitude: 46.651; verbatimLongitude: -72.970; **Identification:** identifiedBy: Angela Telfer; **Event:** verbatimEventDate: 01-Aug-2013; **Record Level:** institutionID: Biodiversity Institute of Ontario
- **Type status:** Other material. **Occurrence:** catalogNumber: BIOUG08641-G07; recordedBy: M.Neerhof, S.Lipskie; lifeStage: Adult; otherCatalogNumbers: SMTPD3273-13; **Location:** country: Canada; stateProvince: Ontario; county: Guelph; municipality: John F. Ross Collegiate & Vocational Institute; locality: EQP-CLL-524; verbatimElevation: 329; verbatimLatitude: 43.562; verbatimLongitude: -80.247; **Event:** verbatimEventDate: 27-Sep-2013; **Record Level:** institutionID: Biodiversity Institute of Ontario
- **Type status:** Other material. **Occurrence:** catalogNumber: CNCHYM 00107; recordedBy: N.C.D.A; lifeStage: Adult; otherCatalogNumbers: HYCND1817-11; **Location:** country: United States; stateProvince: North Carolina; county: Swain County; locality: Smokemont; **Identification:** identifiedBy: Jose Fernandez-Triana; **Event:** verbatimEventDate: 07-Jun-1976; **Record Level:** institutionID: Canadian National Collection of Insects, Arachnids and Nematodes
- **Type status:** Other material. **Occurrence:** catalogNumber: CAM0938; recordedBy: H. Goulet, A. Badiss, C. Boudeault; lifeStage: Adult; otherCatalogNumbers: CNCHX068-09; **Location:** country: Canada; stateProvince: Newfoundland and Labrador; county: St-Andrew`s; locality: St-Andrew`s, fallow field; verbatimLatitude: 47.793; verbatimLongitude: -59.233; **Identification:** identifiedBy: Jose Fernandez-Triana; **Event:** verbatimEventDate: 18-Jul-2008; **Record Level:** institutionID: Canadian National Collection of Insects, Arachnids and Nematodes
- **Type status:** Other material. **Occurrence:** catalogNumber: CAM0348; recordedBy: Goulet & Fernandez; lifeStage: Adult; otherCatalogNumbers: CNCHZ538-09; **Location:** country: Canada; stateProvince: Ontario; county: Almonte; locality: 5 km NW of Almonte, Hwy 49, Burnt Land, Alvar Prov. Park; verbatimLatitude: 45.255; verbatimLongitude: -76.140; **Identification:** identifiedBy: Jose Fernandez-Triana; **Event:** verbatimEventDate: 29-May-2008; **Record Level:** institutionID: Canadian National Collection of Insects, Arachnids and Nematodes
- **Type status:** Other material. **Occurrence:** catalogNumber: BIOUG08647-G07; recordedBy: Lisa Cairncross; lifeStage: Adult; otherCatalogNumbers: SMTPD3831-13; **Location:** country: Canada; stateProvince: Ontario; county: St. Marys; municipality: Little Falls Public School; locality: EQP-CLL-554; verbatimElevation: 305; verbatimLatitude: 43.259; verbatimLongitude: -81.142; **Event:** verbatimEventDate: 27-Sep-2013; **Record Level:** institutionID: Biodiversity Institute of Ontario
- **Type status:** Other material. **Occurrence:** catalogNumber: BIOUG03979-B03; recordedBy: Thousand Islands NP; lifeStage: Adult; otherCatalogNumbers: CNSLI401-12; **Location:** country: Canada; stateProvince: Ontario; county: Thousand Islands NP; municipality: Jones Creek by Mallorytown, County Rd. 5; verbatimElevation: 117; verbatimLatitude: 44.475; verbatimLongitude: -75.865; **Event:** verbatimEventDate: 21-Aug-2012; **Record Level:** institutionID: Biodiversity Institute of Ontario
- **Type status:** Other material. **Occurrence:** catalogNumber: CAM0346; recordedBy: Goulet & Fernandez; lifeStage: Adult; otherCatalogNumbers: CNCHZ536-09; **Location:** country: Canada; stateProvince: Ontario; county: Almonte; locality: 5 km NW of Almonte, Hwy 49, Burnt Land, Alvar Prov. Park; verbatimLatitude: 45.255; verbatimLongitude: -76.140; **Identification:** identifiedBy: Jose Fernandez-Triana; **Event:** verbatimEventDate: 29-May-2008; **Record Level:** institutionID: Canadian National Collection of Insects, Arachnids and Nematodes
- **Type status:** Other material. **Occurrence:** catalogNumber: BIOUG01795-D07; recordedBy: J. Cossey; lifeStage: Adult; otherCatalogNumbers: NCCC726-11; **Location:** country: Canada; stateProvince: Ontario; locality: Scheck Nature Reserve; verbatimLatitude: 44.349; verbatimLongitude: -76.895; **Identification:** identifiedBy: BOLD ID Engine; **Event:** verbatimEventDate: 05-Oct-2011; **Record Level:** institutionID: Biodiversity Institute of Ontario
- **Type status:** Other material. **Occurrence:** catalogNumber: BIOUG11996-F03; recordedBy: Cavan Harpur; lifeStage: Adult; otherCatalogNumbers: CNPKK1009-14; **Location:** country: Canada; stateProvince: Ontario; county: Heron Bay; municipality: Pukaskwa National Park; verbatimElevation: 631; verbatimLatitude: 48.601; verbatimLongitude: -86.289; **Event:** verbatimEventDate: 09-Sep-2013; **Record Level:** institutionID: Biodiversity Institute of Ontario
- **Type status:** Other material. **Occurrence:** catalogNumber: BIOUG11597-E04; recordedBy: D.Crossland, K.Rowter; lifeStage: Adult; otherCatalogNumbers: CNKJE1961-14; **Location:** country: Canada; stateProvince: Nova Scotia; county: Kejimkujik National Park; locality: Jeremy`s Bay Campground, near Ampitheater off of Campfire Circle; verbatimElevation: 116; verbatimLatitude: 44.407; verbatimLongitude: -65.245; **Event:** verbatimEventDate: 11-Jul-2013; **Record Level:** institutionID: Biodiversity Institute of Ontario
- **Type status:** Other material. **Occurrence:** catalogNumber: CAM0445; recordedBy: S. Peck; lifeStage: Adult; otherCatalogNumbers: CNCHZ635-09; **Location:** country: Canada; stateProvince: Ontario; county: Leeds; locality: Leeds-Granville Co., forest; verbatimLatitude: 44.629; verbatimLongitude: -76.359; **Identification:** identifiedBy: Jose Fernandez-Triana; **Event:** verbatimEventDate: 01-Oct-2008; **Record Level:** institutionID: Canadian National Collection of Insects, Arachnids and Nematodes
- **Type status:** Other material. **Occurrence:** catalogNumber: BIOUG07903-F12; recordedBy: BIO Team; lifeStage: Adult; otherCatalogNumbers: RBINA3743-13; **Location:** country: Canada; stateProvince: Ontario; county: Rouge National Urban Park; locality: Sector 8; verbatimElevation: 167; verbatimLatitude: 43.859; verbatimLongitude: -79.209; **Event:** verbatimEventDate: 15-Sep-2013; **Record Level:** institutionID: Biodiversity Institute of Ontario
- **Type status:** Other material. **Occurrence:** catalogNumber: CAM0527; recordedBy: A. Bennett; lifeStage: Adult; otherCatalogNumbers: CNCHZ1147-09; **Location:** country: Canada; stateProvince: Ontario; county: Ottawa; locality: Mixed forest near Manotick; verbatimLatitude: 45.235; verbatimLongitude: -75.624; **Identification:** identifiedBy: Jose Fernandez-Triana; **Record Level:** institutionID: Canadian National Collection of Insects, Arachnids and Nematodes
- **Type status:** Other material. **Occurrence:** catalogNumber: BIOUG01657-C11; recordedBy: J. Cossey; lifeStage: Adult; otherCatalogNumbers: NCCC130-11; **Location:** country: Canada; stateProvince: Ontario; municipality: Carden Alvar; locality: North Bear; verbatimLatitude: 44.695; verbatimLongitude: -79.039; **Event:** verbatimEventDate: 06-Oct-2011; **Record Level:** institutionID: Biodiversity Institute of Ontario
- **Type status:** Other material. **Occurrence:** catalogNumber: 09BBEHY-2030; recordedBy: BIObus 2009; lifeStage: Adult; otherCatalogNumbers: BBHYF031-10; **Location:** country: Canada; stateProvince: New Brunswick; county: Kouchibouguac NP; municipality: Research House; verbatimElevation: 52; verbatimLatitude: 46.771; verbatimLongitude: -65.006; **Identification:** identifiedBy: Jose Fernandez-Triana; **Event:** verbatimEventDate: 20-Aug-2009; **Record Level:** institutionID: Biodiversity Institute of Ontario
- **Type status:** Other material. **Occurrence:** catalogNumber: BIOUG09770-A04; recordedBy: Lydia Attard, Kyla Greenham; lifeStage: Adult; otherCatalogNumbers: CNROT251-13; **Location:** country: Canada; stateProvince: Ontario; county: Toronto; municipality: Rouge National Urban Park, Trap 1; verbatimElevation: 136; verbatimLatitude: 43.822; verbatimLongitude: -79.190; **Event:** verbatimEventDate: 14-Sep-2013; **Record Level:** institutionID: Biodiversity Institute of Ontario
- **Type status:** Other material. **Occurrence:** catalogNumber: CAM0531; recordedBy: A. Bennett; lifeStage: Adult; otherCatalogNumbers: CNCHZ1151-09; **Location:** country: Canada; stateProvince: Ontario; county: Ottawa; locality: Mixed forest near Manotick; verbatimLatitude: 45.235; verbatimLongitude: -75.624; **Identification:** identifiedBy: Jose Fernandez-Triana; **Record Level:** institutionID: Canadian National Collection of Insects, Arachnids and Nematodes
- **Type status:** Other material. **Occurrence:** catalogNumber: CAM0007; recordedBy: H. Goulet; lifeStage: Adult; otherCatalogNumbers: CNCHZ197-09; **Location:** country: Canada; stateProvince: Ontario; county: Ottawa; locality: Ottawa city garden; verbatimLatitude: 45.356; verbatimLongitude: -75.707; **Identification:** identifiedBy: Jose Fernandez-Triana; **Event:** verbatimEventDate: 19-Sep-2007; **Record Level:** institutionID: Canadian National Collection of Insects, Arachnids and Nematodes
- **Type status:** Other material. **Occurrence:** catalogNumber: BIOUG12487-D03; recordedBy: Park Staff; lifeStage: Adult; otherCatalogNumbers: CNMIG324-14; **Location:** country: Canada; stateProvince: Quebec; county: Mingan Archipelago National Park Reserve; municipality: Quarry Island; locality: bog near forest edge; verbatimLatitude: 50.214; verbatimLongitude: -63.798; **Identification:** identifiedBy: Angela Telfer; **Event:** verbatimEventDate: 06-Aug-2013; **Record Level:** institutionID: Biodiversity Institute of Ontario
- **Type status:** Other material. **Occurrence:** catalogNumber: MIC 000086; sex: Female; lifeStage: Adult; otherCatalogNumbers: MHABR086-09; **Location:** country: Canada; stateProvince: Ontario; locality: Brighton; **Identification:** identifiedBy: Jose Fernandez-Triana; **Event:** verbatimEventDate: 24-Jul-1956; **Record Level:** institutionID: Canadian National Collection of Insects, Arachnids and Nematodes
- **Type status:** Other material. **Occurrence:** catalogNumber: ASGLE-0880; recordedBy: Alex Smith; lifeStage: Adult; otherCatalogNumbers: ASGLE690-10; **Location:** country: Canada; stateProvince: Ontario; county: Guelph; municipality: 25 Division St.; verbatimElevation: 326; verbatimLatitude: 43.554; verbatimLongitude: -80.264; **Identification:** identifiedBy: Jose Fernandez-Triana; **Event:** verbatimEventDate: 01-Nov-2009; **Record Level:** institutionID: Research Collection of M. Alex Smith
- **Type status:** Other material. **Occurrence:** catalogNumber: BIOUG03887-F12; recordedBy: Thousand Islands NP; lifeStage: Adult; otherCatalogNumbers: CNSLJ045-12; **Location:** country: Canada; stateProvince: Ontario; county: Thousand Islands NP; municipality: Jones Creek by Mallorytown, County Rd. 5; verbatimElevation: 117; verbatimLatitude: 44.475; verbatimLongitude: -75.865; **Event:** verbatimEventDate: 02-Sep-2012; **Record Level:** institutionID: Biodiversity Institute of Ontario
- **Type status:** Other material. **Occurrence:** catalogNumber: BIOUG08665-D12; recordedBy: Anne Parkhill; lifeStage: Adult; otherCatalogNumbers: SMTPD4966-13; **Location:** country: Canada; stateProvince: Ontario; county: Paris; municipality: Holy Family School; locality: EQP-CLL-560; verbatimElevation: 250; verbatimLatitude: 43.207; verbatimLongitude: -80.391; **Event:** verbatimEventDate: 27-Sep-2013; **Record Level:** institutionID: Biodiversity Institute of Ontario
- **Type status:** Other material. **Occurrence:** catalogNumber: BIOUG09515-E11; recordedBy: BIO Collections Staff; lifeStage: Adult; otherCatalogNumbers: MBIOI1222-13; **Location:** country: Canada; stateProvince: Ontario; county: Wellington County; municipality: Guelph; locality: Biodiversity Institute of Ontario; verbatimElevation: 335; verbatimLatitude: 43.528; verbatimLongitude: -80.229; **Event:** verbatimEventDate: 22-Aug-2013; **Record Level:** institutionID: Biodiversity Institute of Ontario
- **Type status:** Other material. **Occurrence:** catalogNumber: CAM0022; recordedBy: H. Goulet; lifeStage: Adult; otherCatalogNumbers: CNCHZ212-09; **Location:** country: Canada; stateProvince: Ontario; county: Ottawa; locality: Ottawa city garden; verbatimLatitude: 45.356; verbatimLongitude: -75.707; **Identification:** identifiedBy: Jose Fernandez-Triana; **Event:** verbatimEventDate: 13-Jul-2007; **Record Level:** institutionID: Canadian National Collection of Insects, Arachnids and Nematodes
- **Type status:** Other material. **Occurrence:** catalogNumber: CAM0446; recordedBy: S. Peck; lifeStage: Adult; otherCatalogNumbers: CNCHZ636-09; **Location:** country: Canada; stateProvince: Ontario; county: Leeds; locality: Leeds-Granville Co., forest; verbatimLatitude: 44.629; verbatimLongitude: -76.359; **Identification:** identifiedBy: Jose Fernandez-Triana; **Event:** verbatimEventDate: 01-Oct-2008; **Record Level:** institutionID: Canadian National Collection of Insects, Arachnids and Nematodes
- **Type status:** Other material. **Occurrence:** catalogNumber: BIOUG01631-A09; recordedBy: James Sones; lifeStage: Adult; otherCatalogNumbers: JSAUG1440-11; **Location:** country: Canada; stateProvince: Ontario; county: Leeds and Grenville; municipality: Elizabethtown-Kitley; locality: 4452 Rowsome Rd., Elizabethtown; verbatimElevation: 111; verbatimLatitude: 44.618; verbatimLongitude: -75.775; **Identification:** identifiedBy: BOLD ID Engine; **Event:** verbatimEventDate: 18-Aug-2011; **Record Level:** institutionID: Biodiversity Institute of Ontario
- **Type status:** Other material. **Occurrence:** catalogNumber: CAM0635; recordedBy: N. Islam; lifeStage: Adult; otherCatalogNumbers: ASCNC020-09; **Location:** country: Canada; stateProvince: Ontario; county: Cochrane; locality: Cochrane & Kapuscaning Forest Reserves; verbatimLatitude: 49.398; verbatimLongitude: -80.472; **Identification:** identifiedBy: Jose Fernandez-Triana; **Event:** verbatimEventDate: 12-Aug-2007; **Record Level:** institutionID: Canadian National Collection of Insects, Arachnids and Nematodes
- **Type status:** Other material. **Occurrence:** catalogNumber: CAM0335; recordedBy: Goulet & Fernandez; lifeStage: Adult; otherCatalogNumbers: CNCHZ525-09; **Location:** country: Canada; stateProvince: Ontario; county: Almonte; locality: 5 km NW of Almonte, Hwy 49, Burnt Land, Alvar Prov. Park; verbatimLatitude: 45.255; verbatimLongitude: -76.140; **Identification:** identifiedBy: Jose Fernandez-Triana; **Event:** verbatimEventDate: 29-May-2008; **Record Level:** institutionID: Canadian National Collection of Insects, Arachnids and Nematodes
- **Type status:** Other material. **Occurrence:** catalogNumber: GOU 0570; recordedBy: J. Fernandez; lifeStage: Adult; otherCatalogNumbers: WOMIA340-11; **Location:** country: Canada; stateProvince: Nova Scotia; locality: Kejimkujik National Park, Grafton Lake; verbatimLatitude: 44.383; verbatimLongitude: -65.202; **Identification:** identifiedBy: Jose Fernandez-Triana; **Event:** verbatimEventDate: 04-Aug-2009; **Record Level:** institutionID: Canadian National Collection of Insects, Arachnids and Nematodes
- **Type status:** Other material. **Occurrence:** catalogNumber: BIOUG07902-D08; recordedBy: BIO Team; lifeStage: Adult; otherCatalogNumbers: RBINA3620-13; **Location:** country: Canada; stateProvince: Ontario; county: Rouge National Urban Park; locality: Sector 8; verbatimElevation: 167; verbatimLatitude: 43.859; verbatimLongitude: -79.209; **Event:** verbatimEventDate: 15-Sep-2013; **Record Level:** institutionID: Biodiversity Institute of Ontario
- **Type status:** Other material. **Occurrence:** catalogNumber: HYM00001026; recordedBy: Goulet & Boudreault; lifeStage: Adult; otherCatalogNumbers: ASWA312-08; **Location:** country: Canada; stateProvince: Manitoba; locality: Gardenton; verbatimElevation: 292; verbatimLatitude: 49.079; verbatimLongitude: -96.766; **Identification:** identifiedBy: Jose Fernandez-Triana; **Event:** verbatimEventDate: 11-Jun-2007; **Record Level:** institutionID: Canadian National Collection of Insects, Arachnids and Nematodes
- **Type status:** Other material. **Occurrence:** catalogNumber: CAM0523; recordedBy: A. Bennett; lifeStage: Adult; otherCatalogNumbers: CNCHZ1143-09; **Location:** country: Canada; stateProvince: Ontario; county: Ottawa; locality: Mixed forest near Manotick; verbatimLatitude: 45.235; verbatimLongitude: -75.624; **Identification:** identifiedBy: Jose Fernandez-Triana; **Record Level:** institutionID: Canadian National Collection of Insects, Arachnids and Nematodes
- **Type status:** Other material. **Occurrence:** catalogNumber: CAM0356; recordedBy: Goulet & Fernandez; lifeStage: Adult; otherCatalogNumbers: CNCHZ546-09; **Location:** country: Canada; stateProvince: Ontario; county: Almonte; locality: 5 km NW of Almonte, Hwy 49, Burnt Land, Alvar Prov. Park; verbatimLatitude: 45.255; verbatimLongitude: -76.140; **Identification:** identifiedBy: Jose Fernandez-Triana; **Event:** verbatimEventDate: 29-May-2008; **Record Level:** institutionID: Canadian National Collection of Insects, Arachnids and Nematodes
- **Type status:** Other material. **Occurrence:** catalogNumber: CNCH1358; recordedBy: Goulet & Boudreault; lifeStage: Adult; otherCatalogNumbers: CNCHW328-09; **Location:** country: United States; stateProvince: Alaska; locality: Anchorage; verbatimElevation: 33; verbatimLatitude: 61.166; verbatimLongitude: -149.766; **Identification:** identifiedBy: Jose Fernandez-Triana; **Event:** verbatimEventDate: 08-Jun-2003; **Record Level:** institutionID: Canadian National Collection of Insects, Arachnids and Nematodes
- **Type status:** Other material. **Occurrence:** catalogNumber: MIC 000083; recordedBy: O. Peck; sex: Male; lifeStage: Adult; otherCatalogNumbers: MHABR083-09; **Location:** country: Canada; stateProvince: Ontario; county: Ottawa; locality: Blackburn; **Identification:** identifiedBy: Jose Fernandez-Triana; **Event:** verbatimEventDate: 09-Jun-1939; **Record Level:** institutionID: Canadian National Collection of Insects, Arachnids and Nematodes
- **Type status:** Other material. **Occurrence:** catalogNumber: BIOUG12977-D01; recordedBy: Medo Toure; lifeStage: Adult; otherCatalogNumbers: CNLMG1225-14; **Location:** country: Canada; stateProvince: Quebec; county: La Mauricie National Park; municipality: Saint-Mathieu west park entrance near Visitor Centre; locality: primarily conifer forest stand (balsam fir, Epicea glauca, birch); verbatimElevation: 219; verbatimLatitude: 46.651; verbatimLongitude: -72.970; **Identification:** identifiedBy: Angela Telfer; **Event:** verbatimEventDate: 01-Aug-2013; **Record Level:** institutionID: Biodiversity Institute of Ontario
- **Type status:** Other material. **Occurrence:** catalogNumber: BIOUG11331-F04; recordedBy: J. Howard, C. Johnstone; lifeStage: Adult; otherCatalogNumbers: CNGBN1394-14; **Location:** country: Canada; stateProvince: Ontario; county: Georgian Bay Islands National Park; municipality: Beausoleil Island, Cedar Spring Campground; locality: Mixed forest; verbatimElevation: 177; verbatimLatitude: 44.852; verbatimLongitude: -79.874; **Identification:** identifiedBy: Angela Telfer; **Event:** verbatimEventDate: 19-Aug-2013; **Record Level:** institutionID: Biodiversity Institute of Ontario
- **Type status:** Other material. **Occurrence:** catalogNumber: BIOUG03863-G08; recordedBy: Thousand Islands NP; lifeStage: Adult; otherCatalogNumbers: CNSLI089-12; **Location:** country: Canada; stateProvince: Ontario; county: Thousand Islands NP; municipality: Jones Creek by Mallorytown, County Rd. 5; verbatimElevation: 117; verbatimLatitude: 44.475; verbatimLongitude: -75.865; **Event:** verbatimEventDate: 21-Aug-2012; **Record Level:** institutionID: Biodiversity Institute of Ontario
- **Type status:** Other material. **Occurrence:** catalogNumber: BIOUG09765-B03; recordedBy: Lydia Attard, Kyla Greenham; lifeStage: Adult; otherCatalogNumbers: CNROS484-13; **Location:** country: Canada; stateProvince: Ontario; county: Toronto; municipality: Rouge National Urban Park, Trap 1; verbatimElevation: 136; verbatimLatitude: 43.822; verbatimLongitude: -79.190; **Event:** verbatimEventDate: 27-Aug-2013; **Record Level:** institutionID: Biodiversity Institute of Ontario
- **Type status:** Other material. **Occurrence:** catalogNumber: CAM0410; recordedBy: Goulet, Boudreault & Fernandez; lifeStage: Adult; otherCatalogNumbers: CNCHZ600-09; **Location:** country: Canada; stateProvince: Ontario; county: Manitoulin Island; locality: Little Current, prairie alvar; verbatimLatitude: 45.975; verbatimLongitude: -81.904; **Identification:** identifiedBy: Jose Fernandez-Triana; **Event:** verbatimEventDate: 04-Jun-2008; **Record Level:** institutionID: Canadian National Collection of Insects, Arachnids and Nematodes
- **Type status:** Other material. **Occurrence:** catalogNumber: CAM0113; recordedBy: H. Goulet; lifeStage: Adult; otherCatalogNumbers: CNCHZ303-09; **Location:** country: Canada; stateProvince: Ontario; county: Ottawa; locality: Ottawa city garden; verbatimLatitude: 45.356; verbatimLongitude: -75.707; **Identification:** identifiedBy: Jose Fernandez-Triana; **Event:** verbatimEventDate: 10-Aug-2007; **Record Level:** institutionID: Canadian National Collection of Insects, Arachnids and Nematodes
- **Type status:** Other material. **Occurrence:** catalogNumber: WMIC 0517; recordedBy: L. Gualtieri; lifeStage: Adult; otherCatalogNumbers: CNCHX802-09; **Location:** country: Canada; stateProvince: Ontario; locality: London; verbatimLatitude: 43.030; verbatimLongitude: -81.206; **Identification:** identifiedBy: Jose Fernandez-Triana; **Event:** verbatimEventDate: 07-Jul-2003; **Record Level:** institutionID: Canadian National Collection of Insects, Arachnids and Nematodes
- **Type status:** Other material. **Occurrence:** catalogNumber: BIOUG11999-F03; recordedBy: Cavan Harpur; lifeStage: Adult; otherCatalogNumbers: CNPKK1294-14; **Location:** country: Canada; stateProvince: Ontario; county: Heron Bay; municipality: Pukaskwa National Park; verbatimElevation: 631; verbatimLatitude: 48.601; verbatimLongitude: -86.289; **Event:** verbatimEventDate: 09-Sep-2013; **Record Level:** institutionID: Biodiversity Institute of Ontario
- **Type status:** Other material. **Occurrence:** catalogNumber: BIOUG00726-B07; recordedBy: Brandon Laforest; lifeStage: Adult; otherCatalogNumbers: JBHCI247-11; **Location:** country: Canada; stateProvince: Manitoba; county: Churchill; municipality: 23 km SE Churchill, Twin Lakes Road fen; locality: fen grasses and willows, 5km down Twin lakes rd; verbatimLatitude: 58.672; verbatimLongitude: -93.839; **Identification:** identifiedBy: Jose Fernandez-Triana; **Event:** verbatimEventDate: 29-Jul-2010; **Record Level:** institutionID: Biodiversity Institute of Ontario
- **Type status:** Other material. **Occurrence:** catalogNumber: CAM0151; recordedBy: H. Goulet; lifeStage: Adult; otherCatalogNumbers: CNCHZ341-09; **Location:** country: Canada; stateProvince: Ontario; county: Ottawa; locality: Ottawa city garden; verbatimLatitude: 45.356; verbatimLongitude: -75.707; **Identification:** identifiedBy: Jose Fernandez-Triana; **Event:** verbatimEventDate: 01-Sep-2007; **Record Level:** institutionID: Canadian National Collection of Insects, Arachnids and Nematodes
- **Type status:** Other material. **Occurrence:** catalogNumber: CAM0524; recordedBy: A. Bennett; lifeStage: Adult; otherCatalogNumbers: CNCHZ1144-09; **Location:** country: Canada; stateProvince: Ontario; county: Ottawa; locality: Mixed forest near Manotick; verbatimLatitude: 45.235; verbatimLongitude: -75.624; **Identification:** identifiedBy: Jose Fernandez-Triana; **Record Level:** institutionID: Canadian National Collection of Insects, Arachnids and Nematodes
- **Type status:** Other material. **Occurrence:** catalogNumber: BIOUG03123-B05; recordedBy: Thousand Islands NP; lifeStage: Adult; otherCatalogNumbers: CNSLE365-12; **Location:** country: Canada; stateProvince: Ontario; county: Thousand Islands NP; municipality: Jones Creek by Mallorytown, County Rd. 5; verbatimElevation: 117; verbatimLatitude: 44.475; verbatimLongitude: -75.865; **Event:** verbatimEventDate: 24-Jun-2012; **Record Level:** institutionID: Biodiversity Institute of Ontario
- **Type status:** Other material. **Occurrence:** catalogNumber: CAM0013; recordedBy: H. Goulet; lifeStage: Adult; otherCatalogNumbers: CNCHZ203-09; **Location:** country: Canada; stateProvince: Ontario; county: Ottawa; locality: Ottawa city garden; verbatimLatitude: 45.356; verbatimLongitude: -75.707; **Identification:** identifiedBy: Jose Fernandez-Triana; **Event:** verbatimEventDate: 30-May-2007; **Record Level:** institutionID: Canadian National Collection of Insects, Arachnids and Nematodes
- **Type status:** Other material. **Occurrence:** catalogNumber: MIC 000082; recordedBy: G. Shewell; sex: Male; lifeStage: Adult; otherCatalogNumbers: MHABR082-09; **Location:** country: Canada; stateProvince: Ontario; county: Ottawa; locality: Britannia; **Identification:** identifiedBy: Jose Fernandez-Triana; **Event:** verbatimEventDate: 20-Jun-1947; **Record Level:** institutionID: Canadian National Collection of Insects, Arachnids and Nematodes
- **Type status:** Other material. **Occurrence:** catalogNumber: BIOUG14171-B01; recordedBy: Cavan Harpur; lifeStage: Adult; otherCatalogNumbers: CNPKO2958-14; **Location:** country: Canada; stateProvince: Ontario; county: Heron Bay; municipality: Pukaskwa National Park; verbatimElevation: 631; verbatimLatitude: 48.601; verbatimLongitude: -86.289; **Identification:** identifiedBy: Angela Telfer; **Event:** verbatimEventDate: 01-Sep-2013; **Record Level:** institutionID: Biodiversity Institute of Ontario
- **Type status:** Other material. **Occurrence:** catalogNumber: CAM0942; recordedBy: H. Goulet, A. Badiss, C. Boudeault; lifeStage: Adult; otherCatalogNumbers: CNCHX072-09; **Location:** country: Canada; stateProvince: Newfoundland and Labrador; county: St-Andrew`s; locality: St-Andrew`s, fallow field; verbatimLatitude: 47.793; verbatimLongitude: -59.233; **Identification:** identifiedBy: Jose Fernandez-Triana; **Event:** verbatimEventDate: 18-Jul-2008; **Record Level:** institutionID: Canadian National Collection of Insects, Arachnids and Nematodes
- **Type status:** Other material. **Occurrence:** catalogNumber: BIOUG09803-F06; recordedBy: BIO Collections Staff; lifeStage: Adult; otherCatalogNumbers: MBIOK076-14; **Location:** country: Canada; stateProvince: Ontario; county: Wellington County; municipality: Guelph; locality: Biodiversity Institute of Ontario; verbatimElevation: 335; verbatimLatitude: 43.528; verbatimLongitude: -80.229; **Event:** verbatimEventDate: 19-Sep-2013; **Record Level:** institutionID: Biodiversity Institute of Ontario
- **Type status:** Other material. **Occurrence:** catalogNumber: BIOUG07004-C04; recordedBy: Thousand Islands NP; lifeStage: Adult; otherCatalogNumbers: CNSLQ251-13; **Location:** country: Canada; stateProvince: Ontario; county: Thousand Islands NP; municipality: Jones Creek by Mallorytown, County Rd. 5; verbatimElevation: 117; verbatimLatitude: 44.475; verbatimLongitude: -75.865; **Event:** verbatimEventDate: 15-Jul-2012; **Record Level:** institutionID: Biodiversity Institute of Ontario
- **Type status:** Other material. **Occurrence:** catalogNumber: CAM0529; recordedBy: A. Bennett; lifeStage: Adult; otherCatalogNumbers: CNCHZ1149-09; **Location:** country: Canada; stateProvince: Ontario; county: Ottawa; locality: Mixed forest near Manotick; verbatimLatitude: 45.235; verbatimLongitude: -75.624; **Identification:** identifiedBy: Jose Fernandez-Triana; **Record Level:** institutionID: Canadian National Collection of Insects, Arachnids and Nematodes
- **Type status:** Other material. **Occurrence:** catalogNumber: CAM0074; recordedBy: H. Goulet; lifeStage: Adult; otherCatalogNumbers: CNCHZ264-09; **Location:** country: Canada; stateProvince: Ontario; county: Ottawa; locality: Ottawa city garden; verbatimLatitude: 45.356; verbatimLongitude: -75.707; **Identification:** identifiedBy: Jose Fernandez-Triana; **Event:** verbatimEventDate: 26-Jun-2007; **Record Level:** institutionID: Canadian National Collection of Insects, Arachnids and Nematodes
- **Type status:** Other material. **Occurrence:** catalogNumber: BIOUG06823-E08; recordedBy: Thousand Islands NP; lifeStage: Adult; otherCatalogNumbers: CNSLS117-13; **Location:** country: Canada; stateProvince: Ontario; county: Thousand Islands NP; municipality: Jones Creek by Mallorytown, County Rd. 5; verbatimElevation: 117; verbatimLatitude: 44.475; verbatimLongitude: -75.865; **Event:** verbatimEventDate: 12-Aug-2012; **Record Level:** institutionID: Biodiversity Institute of Ontario
- **Type status:** Other material. **Occurrence:** catalogNumber: BIOUG08665-A11; recordedBy: Anne Parkhill; lifeStage: Adult; otherCatalogNumbers: SMTPD4929-13; **Location:** country: Canada; stateProvince: Ontario; county: Paris; municipality: Holy Family School; locality: EQP-CLL-560; verbatimElevation: 250; verbatimLatitude: 43.207; verbatimLongitude: -80.391; **Event:** verbatimEventDate: 27-Sep-2013; **Record Level:** institutionID: Biodiversity Institute of Ontario
- **Type status:** Other material. **Occurrence:** catalogNumber: BIOUG01631-G11; recordedBy: James Sones; lifeStage: Adult; otherCatalogNumbers: JSSEP758-11; **Location:** country: Canada; stateProvince: Ontario; county: Leeds and Grenville; municipality: Elizabethtown-Kitley; locality: 4452 Rowsome Rd., Elizabethtown; verbatimElevation: 111; verbatimLatitude: 44.618; verbatimLongitude: -75.775; **Identification:** identifiedBy: BOLD ID Engine; **Event:** verbatimEventDate: 14-Sep-2011; **Record Level:** institutionID: Biodiversity Institute of Ontario
- **Type status:** Other material. **Occurrence:** catalogNumber: BIOUG12974-A03; recordedBy: Medo Toure; lifeStage: Adult; otherCatalogNumbers: CNLMG906-14; **Location:** country: Canada; stateProvince: Quebec; county: La Mauricie National Park; municipality: Saint-Mathieu west park entrance near Visitor Centre; locality: primarily conifer forest stand (balsam fir, Epicea glauca, birch); verbatimElevation: 219; verbatimLatitude: 46.651; verbatimLongitude: -72.970; **Identification:** identifiedBy: Angela Telfer; **Event:** verbatimEventDate: 01-Aug-2013; **Record Level:** institutionID: Biodiversity Institute of Ontario
- **Type status:** Other material. **Occurrence:** catalogNumber: CAM0447; recordedBy: S. Peck; lifeStage: Adult; otherCatalogNumbers: CNCHZ637-09; **Location:** country: Canada; stateProvince: Ontario; county: Leeds; locality: Leeds-Granville Co., forest; verbatimLatitude: 44.629; verbatimLongitude: -76.359; **Identification:** identifiedBy: Jose Fernandez-Triana; **Event:** verbatimEventDate: 01-Oct-2008; **Record Level:** institutionID: Canadian National Collection of Insects, Arachnids and Nematodes
- **Type status:** Other material. **Occurrence:** catalogNumber: BIOUG01597-F04; recordedBy: James Sones; lifeStage: Adult; otherCatalogNumbers: JSAUG449-11; **Location:** country: Canada; stateProvince: Ontario; county: Leeds and Grenville; municipality: Elizabethtown-Kitley; locality: 4452 Rowsome Rd., Elizabethtown; verbatimElevation: 111; verbatimLatitude: 44.618; verbatimLongitude: -75.775; **Identification:** identifiedBy: BOLD ID Engine; **Event:** verbatimEventDate: 18-Aug-2011; **Record Level:** institutionID: Biodiversity Institute of Ontario
- **Type status:** Other material. **Occurrence:** catalogNumber: BIOUG08647-D01; recordedBy: Lisa Cairncross; lifeStage: Adult; otherCatalogNumbers: SMTPD3789-13; **Location:** country: Canada; stateProvince: Ontario; county: St. Marys; municipality: Little Falls Public School; locality: EQP-CLL-554; verbatimElevation: 305; verbatimLatitude: 43.259; verbatimLongitude: -81.142; **Event:** verbatimEventDate: 27-Sep-2013; **Record Level:** institutionID: Biodiversity Institute of Ontario
- **Type status:** Other material. **Occurrence:** catalogNumber: BIOUG01657-F02; recordedBy: J. Cossey; lifeStage: Adult; otherCatalogNumbers: NCCC157-11; **Location:** country: Canada; stateProvince: Ontario; municipality: Carden Alvar; locality: North Bear; verbatimLatitude: 44.695; verbatimLongitude: -79.039; **Event:** verbatimEventDate: 06-Oct-2011; **Record Level:** institutionID: Biodiversity Institute of Ontario
- **Type status:** Other material. **Occurrence:** catalogNumber: BIOUG09765-A12; recordedBy: Lydia Attard, Kyla Greenham; lifeStage: Adult; otherCatalogNumbers: CNROS481-13; **Location:** country: Canada; stateProvince: Ontario; county: Toronto; municipality: Rouge National Urban Park, Trap 1; verbatimElevation: 136; verbatimLatitude: 43.822; verbatimLongitude: -79.190; **Event:** verbatimEventDate: 27-Aug-2013; **Record Level:** institutionID: Biodiversity Institute of Ontario
- **Type status:** Other material. **Occurrence:** catalogNumber: BIOUG03979-H08; recordedBy: Thousand Islands NP; lifeStage: Adult; otherCatalogNumbers: CNSLJ189-12; **Location:** country: Canada; stateProvince: Ontario; county: Thousand Islands NP; municipality: Jones Creek by Mallorytown, County Rd. 5; verbatimElevation: 117; verbatimLatitude: 44.475; verbatimLongitude: -75.865; **Event:** verbatimEventDate: 02-Sep-2012; **Record Level:** institutionID: Biodiversity Institute of Ontario
- **Type status:** Other material. **Occurrence:** catalogNumber: CAM0350; recordedBy: Goulet & Fernandez; lifeStage: Adult; otherCatalogNumbers: CNCHZ540-09; **Location:** country: Canada; stateProvince: Ontario; county: Almonte; locality: 5 km NW of Almonte, Hwy 49, Burnt Land, Alvar Prov. Park; verbatimLatitude: 45.255; verbatimLongitude: -76.140; **Identification:** identifiedBy: Jose Fernandez-Triana; **Event:** verbatimEventDate: 29-May-2008; **Record Level:** institutionID: Canadian National Collection of Insects, Arachnids and Nematodes
- **Type status:** Other material. **Occurrence:** catalogNumber: BIOUG03931-F01; recordedBy: H.Brown; lifeStage: Adult; otherCatalogNumbers: CNPPJ1314-12; **Location:** country: Canada; stateProvince: Ontario; county: Point Pelee NP; municipality: Cactus Field; verbatimElevation: 168; verbatimLatitude: 41.939; verbatimLongitude: -82.516; **Event:** verbatimEventDate: 07-Sep-2012; **Record Level:** institutionID: Biodiversity Institute of Ontario
- **Type status:** Other material. **Occurrence:** catalogNumber: BIOUG09930-G10; recordedBy: BIO Collections Staff; lifeStage: Adult; otherCatalogNumbers: MBION369-14; **Location:** country: Canada; stateProvince: Ontario; county: Wellington County; municipality: Guelph; locality: Biodiversity Institute of Ontario; verbatimElevation: 335; verbatimLatitude: 43.528; verbatimLongitude: -80.229; **Event:** verbatimEventDate: 04-Oct-2013; **Record Level:** institutionID: Biodiversity Institute of Ontario
- **Type status:** Other material. **Occurrence:** catalogNumber: BIOUG08648-H10; recordedBy: Lisa Cairncross; lifeStage: Adult; otherCatalogNumbers: SMTPD3941-13; **Location:** country: Canada; stateProvince: Ontario; county: St. Marys; municipality: Little Falls Public School; locality: EQP-CLL-554; verbatimElevation: 305; verbatimLatitude: 43.259; verbatimLongitude: -81.142; **Event:** verbatimEventDate: 27-Sep-2013; **Record Level:** institutionID: Biodiversity Institute of Ontario
- **Type status:** Other material. **Occurrence:** catalogNumber: BIOUG08752-A08; recordedBy: Lydia Attard, Kyla Greenham; lifeStage: Adult; otherCatalogNumbers: CNROJ908-13; **Location:** country: Canada; stateProvince: Ontario; county: Toronto; municipality: Rouge National Urban Park, Trap 1; verbatimElevation: 136; verbatimLatitude: 43.822; verbatimLongitude: -79.190; **Event:** verbatimEventDate: 03-Sep-2013; **Record Level:** institutionID: Biodiversity Institute of Ontario
- **Type status:** Other material. **Occurrence:** catalogNumber: BIOUG12497-F12; recordedBy: Park Staff; lifeStage: Adult; otherCatalogNumbers: CNMIG1286-14; **Location:** country: Canada; stateProvince: Quebec; county: Mingan Archipelago National Park Reserve; municipality: Quarry Island; locality: bog near forest edge; verbatimLatitude: 50.214; verbatimLongitude: -63.798; **Identification:** identifiedBy: Angela Telfer; **Event:** verbatimEventDate: 06-Aug-2013; **Record Level:** institutionID: Biodiversity Institute of Ontario
- **Type status:** Other material. **Occurrence:** catalogNumber: BIOUG08665-F02; recordedBy: Anne Parkhill; lifeStage: Adult; otherCatalogNumbers: SMTPD4980-13; **Location:** country: Canada; stateProvince: Ontario; county: Paris; municipality: Holy Family School; locality: EQP-CLL-560; verbatimElevation: 250; verbatimLatitude: 43.207; verbatimLongitude: -80.391; **Event:** verbatimEventDate: 27-Sep-2013; **Record Level:** institutionID: Biodiversity Institute of Ontario
- **Type status:** Other material. **Occurrence:** catalogNumber: BIOUG09930-E06; recordedBy: BIO Collections Staff; lifeStage: Adult; otherCatalogNumbers: MBION341-14; **Location:** country: Canada; stateProvince: Ontario; county: Wellington County; municipality: Guelph; locality: Biodiversity Institute of Ontario; verbatimElevation: 335; verbatimLatitude: 43.528; verbatimLongitude: -80.229; **Event:** verbatimEventDate: 04-Oct-2013; **Record Level:** institutionID: Biodiversity Institute of Ontario
- **Type status:** Other material. **Occurrence:** catalogNumber: CAM0120; recordedBy: H. Goulet; lifeStage: Adult; otherCatalogNumbers: CNCHZ310-09; **Location:** country: Canada; stateProvince: Ontario; county: Ottawa; locality: Ottawa city garden; verbatimLatitude: 45.356; verbatimLongitude: -75.707; **Identification:** identifiedBy: Jose Fernandez-Triana; **Event:** verbatimEventDate: 30-Jul-2007; **Record Level:** institutionID: Canadian National Collection of Insects, Arachnids and Nematodes
- **Type status:** Other material. **Occurrence:** catalogNumber: BIOUG01661-E11; recordedBy: J. Cossey; lifeStage: Adult; otherCatalogNumbers: NCCC249-11; **Location:** country: Canada; stateProvince: Ontario; municipality: Carden Alvar; locality: North Bear; verbatimLatitude: 44.695; verbatimLongitude: -79.039; **Event:** verbatimEventDate: 06-Oct-2011; **Record Level:** institutionID: Biodiversity Institute of Ontario
- **Type status:** Other material. **Occurrence:** catalogNumber: BIOUG09765-A06; recordedBy: Lydia Attard, Kyla Greenham; lifeStage: Adult; otherCatalogNumbers: CNROS475-13; **Location:** country: Canada; stateProvince: Ontario; county: Toronto; municipality: Rouge National Urban Park, Trap 1; verbatimElevation: 136; verbatimLatitude: 43.822; verbatimLongitude: -79.190; **Event:** verbatimEventDate: 27-Aug-2013; **Record Level:** institutionID: Biodiversity Institute of Ontario
- **Type status:** Other material. **Occurrence:** catalogNumber: CAM0389; recordedBy: Boudreault, Goulet & Fernandez; lifeStage: Adult; otherCatalogNumbers: CNCHZ579-09; **Location:** country: Canada; stateProvince: Ontario; county: Manitoulin Island; locality: Misery Bay Prov. Park; verbatimElevation: 185; verbatimLatitude: 45.792; verbatimLongitude: -82.750; **Identification:** identifiedBy: Jose Fernandez-Triana; **Event:** verbatimEventDate: 03-Jun-2008; **Record Level:** institutionID: Canadian National Collection of Insects, Arachnids and Nematodes
- **Type status:** Other material. **Occurrence:** catalogNumber: HYM00001020; recordedBy: Goulet & Boudreault; lifeStage: Adult; otherCatalogNumbers: ASWA306-08; **Location:** country: Canada; stateProvince: Manitoba; locality: Gardenton; verbatimElevation: 292; verbatimLatitude: 49.079; verbatimLongitude: -96.766; **Identification:** identifiedBy: Jose Fernandez-Triana; **Event:** verbatimEventDate: 11-Jun-2007; **Record Level:** institutionID: Canadian National Collection of Insects, Arachnids and Nematodes
- **Type status:** Other material. **Occurrence:** catalogNumber: CAM0286; recordedBy: H. Goulet, A. Badiss, C. Boudeault; lifeStage: Adult; otherCatalogNumbers: CNCHZ476-09; **Location:** country: Canada; stateProvince: Quebec; county: Belle-Anse; locality: Belle-Anse; verbatimLatitude: 48.625; verbatimLongitude: -64.178; **Identification:** identifiedBy: Jose Fernandez-Triana; **Event:** verbatimEventDate: 26-Jul-2008; **Record Level:** institutionID: Canadian National Collection of Insects, Arachnids and Nematodes
- **Type status:** Other material. **Occurrence:** catalogNumber: CAM0163; recordedBy: H. Goulet; lifeStage: Adult; otherCatalogNumbers: CNCHZ353-09; **Location:** country: Canada; stateProvince: Ontario; county: Ottawa; locality: Ottawa city garden; verbatimLatitude: 45.356; verbatimLongitude: -75.707; **Identification:** identifiedBy: Jose Fernandez-Triana; **Event:** verbatimEventDate: 01-Sep-2007; **Record Level:** institutionID: Canadian National Collection of Insects, Arachnids and Nematodes
- **Type status:** Other material. **Occurrence:** catalogNumber: CAM0351; recordedBy: Goulet & Fernandez; lifeStage: Adult; otherCatalogNumbers: CNCHZ541-09; **Location:** country: Canada; stateProvince: Ontario; county: Almonte; locality: 5 km NW of Almonte, Hwy 49, Burnt Land, Alvar Prov. Park; verbatimLatitude: 45.255; verbatimLongitude: -76.140; **Identification:** identifiedBy: Jose Fernandez-Triana; **Event:** verbatimEventDate: 29-May-2008; **Record Level:** institutionID: Canadian National Collection of Insects, Arachnids and Nematodes
- **Type status:** Other material. **Occurrence:** catalogNumber: TDWG-0384; recordedBy: BIObus: A.Borisenko, R.Labbee, V.Levesque-Beaudin, M.Milton, J.Smith, S.Ratnasingham; lifeStage: Adult; otherCatalogNumbers: TDWGB248-10; **Location:** country: United States; stateProvince: Massachusetts; county: Barnstable Country; municipality: Woods Hole|Woods Hole Village; locality: Urban parkland: Meadow/Anthropogenic; verbatimLatitude: 41.529; verbatimLongitude: -70.670; **Event:** verbatimEventDate: 29-Sep-2010; **Record Level:** institutionID: Biodiversity Institute of Ontario
- **Type status:** Other material. **Occurrence:** catalogNumber: CAM0413; recordedBy: Boudreault, Goulet & Fernandez; lifeStage: Adult; otherCatalogNumbers: CNCHZ603-09; **Location:** country: Canada; stateProvince: Ontario; county: Manitoulin Island; locality: Misery Bay Prov. Park; verbatimElevation: 185; verbatimLatitude: 45.792; verbatimLongitude: -82.750; **Identification:** identifiedBy: Jose Fernandez-Triana; **Event:** verbatimEventDate: 03-Jun-2008; **Record Level:** institutionID: Canadian National Collection of Insects, Arachnids and Nematodes
- **Type status:** Other material. **Occurrence:** catalogNumber: BIOUG06823-D07; recordedBy: Thousand Islands NP; lifeStage: Adult; otherCatalogNumbers: CNSLS104-13; **Location:** country: Canada; stateProvince: Ontario; county: Thousand Islands NP; municipality: Jones Creek by Mallorytown, County Rd. 5; verbatimElevation: 117; verbatimLatitude: 44.475; verbatimLongitude: -75.865; **Event:** verbatimEventDate: 12-Aug-2012; **Record Level:** institutionID: Biodiversity Institute of Ontario
- **Type status:** Other material. **Occurrence:** catalogNumber: CNCHYM 00106; recordedBy: F. Naumann; lifeStage: Adult; otherCatalogNumbers: HYCND1816-11; **Location:** country: United States; stateProvince: Georgia; locality: Forsyth; **Identification:** identifiedBy: Jose Fernandez-Triana; **Event:** verbatimEventDate: 08-Oct-1970; **Record Level:** institutionID: Canadian National Collection of Insects, Arachnids and Nematodes
- **Type status:** Other material. **Occurrence:** catalogNumber: BIOUG08499-C11; recordedBy: Kristen McGuire; lifeStage: Adult; otherCatalogNumbers: SMTPD1460-13; **Location:** country: Canada; stateProvince: Ontario; county: Teeswater; municipality: Sacred Heart School; locality: EQP-CLL-568; verbatimElevation: 314; verbatimLatitude: 43.992; verbatimLongitude: -81.282; **Event:** verbatimEventDate: 27-Sep-2013; **Record Level:** institutionID: Biodiversity Institute of Ontario
- **Type status:** Other material. **Occurrence:** catalogNumber: CAM0360; recordedBy: Goulet & Fernandez; lifeStage: Adult; otherCatalogNumbers: CNCHZ550-09; **Location:** country: Canada; stateProvince: Ontario; county: Almonte; locality: 5 km NW of Almonte, Hwy 49, Burnt Land, Alvar Prov. Park; verbatimLatitude: 45.255; verbatimLongitude: -76.140; **Identification:** identifiedBy: Jose Fernandez-Triana; **Event:** verbatimEventDate: 29-May-2008; **Record Level:** institutionID: Canadian National Collection of Insects, Arachnids and Nematodes
- **Type status:** Other material. **Occurrence:** catalogNumber: CAM0349; recordedBy: Goulet & Fernandez; lifeStage: Adult; otherCatalogNumbers: CNCHZ539-09; **Location:** country: Canada; stateProvince: Ontario; county: Almonte; locality: 5 km NW of Almonte, Hwy 49, Burnt Land, Alvar Prov. Park; verbatimLatitude: 45.255; verbatimLongitude: -76.140; **Identification:** identifiedBy: Jose Fernandez-Triana; **Event:** verbatimEventDate: 29-May-2008; **Record Level:** institutionID: Canadian National Collection of Insects, Arachnids and Nematodes
- **Type status:** Other material. **Occurrence:** catalogNumber: CAM0115; recordedBy: H. Goulet; lifeStage: Adult; otherCatalogNumbers: CNCHZ305-09; **Location:** country: Canada; stateProvince: Ontario; county: Ottawa; locality: Ottawa city garden; verbatimLatitude: 45.356; verbatimLongitude: -75.707; **Identification:** identifiedBy: Jose Fernandez-Triana; **Event:** verbatimEventDate: 10-Aug-2007; **Record Level:** institutionID: Canadian National Collection of Insects, Arachnids and Nematodes
- **Type status:** Other material. **Occurrence:** catalogNumber: MIC 000085; recordedBy: H. Boyce; sex: Female; lifeStage: Adult; otherCatalogNumbers: MHABR085-09; **Location:** country: Canada; stateProvince: Ontario; locality: Grimsby; **Identification:** identifiedBy: Jose Fernandez-Triana; **Event:** verbatimEventDate: 09-Jun-1944; **Record Level:** institutionID: Canadian National Collection of Insects, Arachnids and Nematodes
- **Type status:** Other material. **Occurrence:** catalogNumber: BIOUG03979-G06; recordedBy: Thousand Islands NP; lifeStage: Adult; otherCatalogNumbers: CNSLJ175-12; **Location:** country: Canada; stateProvince: Ontario; county: Thousand Islands NP; municipality: Jones Creek by Mallorytown, County Rd. 5; verbatimElevation: 117; verbatimLatitude: 44.475; verbatimLongitude: -75.865; **Event:** verbatimEventDate: 02-Sep-2012; **Record Level:** institutionID: Biodiversity Institute of Ontario
- **Type status:** Other material. **Occurrence:** catalogNumber: CAM0964; recordedBy: H. Goulet, A. Badiss, C. Boudeault; lifeStage: Adult; otherCatalogNumbers: CNCHX094-09; **Location:** country: Canada; stateProvince: Newfoundland and Labrador; county: St-David`s; locality: St-David`s, fallow field; verbatimLatitude: 48.205; verbatimLongitude: -58.866; **Identification:** identifiedBy: Jose Fernandez-Triana; **Event:** verbatimEventDate: 10-Jul-2008; **Record Level:** institutionID: Canadian National Collection of Insects, Arachnids and Nematodes
- **Type status:** Other material. **Occurrence:** catalogNumber: CAM0025; recordedBy: H. Goulet; lifeStage: Adult; otherCatalogNumbers: CNCHZ215-09; **Location:** country: Canada; stateProvince: Ontario; county: Ottawa; locality: Ottawa city garden; verbatimLatitude: 45.356; verbatimLongitude: -75.707; **Identification:** identifiedBy: Jose Fernandez-Triana; **Event:** verbatimEventDate: 30-Jul-2007; **Record Level:** institutionID: Canadian National Collection of Insects, Arachnids and Nematodes
- **Type status:** Other material. **Occurrence:** catalogNumber: MIC 000088; sex: Female; lifeStage: Adult; otherCatalogNumbers: MHABR088-09; **Location:** country: Canada; stateProvince: Ontario; county: St. Lawrence Islands National Park; locality: Grenadier Island Center; **Identification:** identifiedBy: Jose Fernandez-Triana; **Event:** verbatimEventDate: 21-Jul-1975; **Record Level:** institutionID: Canadian National Collection of Insects, Arachnids and Nematodes
- **Type status:** Other material. **Occurrence:** catalogNumber: BIOUG08807-A09; recordedBy: BIO Collections Staff; lifeStage: Adult; otherCatalogNumbers: MBIOE2286-13; **Location:** country: Canada; stateProvince: Ontario; county: Wellington County; municipality: Guelph; locality: Biodiversity Institute of Ontario; verbatimElevation: 335; verbatimLatitude: 43.528; verbatimLongitude: -80.229; **Event:** verbatimEventDate: 27-Jun-2013; **Record Level:** institutionID: Biodiversity Institute of Ontario
- **Type status:** Other material. **Occurrence:** catalogNumber: CAM0101; recordedBy: H. Goulet; lifeStage: Adult; otherCatalogNumbers: CNCHZ291-09; **Location:** country: Canada; stateProvince: Ontario; county: Ottawa; locality: Ottawa city garden; verbatimLatitude: 45.356; verbatimLongitude: -75.707; **Identification:** identifiedBy: Jose Fernandez-Triana; **Event:** verbatimEventDate: 08-Sep-2007; **Record Level:** institutionID: Canadian National Collection of Insects, Arachnids and Nematodes
- **Type status:** Other material. **Occurrence:** catalogNumber: BIOUG09925-F01; recordedBy: BIO Collections Staff; lifeStage: Adult; otherCatalogNumbers: MBIOL370-14; **Location:** country: Canada; stateProvince: Ontario; county: Wellington County; municipality: Guelph; locality: Biodiversity Institute of Ontario; verbatimElevation: 335; verbatimLatitude: 43.528; verbatimLongitude: -80.229; **Event:** verbatimEventDate: 27-Sep-2013; **Record Level:** institutionID: Biodiversity Institute of Ontario
- **Type status:** Other material. **Occurrence:** catalogNumber: BIOUG09345-D07; recordedBy: BIO Collections Staff; lifeStage: Adult; otherCatalogNumbers: MBIOH323-13; **Location:** country: Canada; stateProvince: Ontario; county: Wellington County; municipality: Guelph; locality: Biodiversity Institute of Ontario; verbatimElevation: 335; verbatimLatitude: 43.528; verbatimLongitude: -80.229; **Event:** verbatimEventDate: 08-Aug-2013; **Record Level:** institutionID: Biodiversity Institute of Ontario
- **Type status:** Other material. **Occurrence:** catalogNumber: BIOUG09925-A01; recordedBy: BIO Collections Staff; lifeStage: Adult; otherCatalogNumbers: MBIOL310-14; **Location:** country: Canada; stateProvince: Ontario; county: Wellington County; municipality: Guelph; locality: Biodiversity Institute of Ontario; verbatimElevation: 335; verbatimLatitude: 43.528; verbatimLongitude: -80.229; **Event:** verbatimEventDate: 27-Sep-2013; **Record Level:** institutionID: Biodiversity Institute of Ontario
- **Type status:** Other material. **Occurrence:** catalogNumber: BIOUG14261-G11; recordedBy: Medo Toure; lifeStage: Adult; otherCatalogNumbers: CNLMP1391-14; **Location:** country: Canada; stateProvince: Quebec; county: La Mauricie National Park; municipality: Saint-Mathieu west park entrance near Visitor Centre; locality: primarily conifer forest stand (balsam fir, Epicea glauca, birch); verbatimElevation: 219; verbatimLatitude: 46.651; verbatimLongitude: -72.970; **Identification:** identifiedBy: Angela Telfer; **Event:** verbatimEventDate: 23-Jul-2013; **Record Level:** institutionID: Biodiversity Institute of Ontario
- **Type status:** Other material. **Occurrence:** catalogNumber: CAM0062; recordedBy: H. Goulet; lifeStage: Adult; otherCatalogNumbers: CNCHZ252-09; **Location:** country: Canada; stateProvince: Ontario; county: Ottawa; locality: Ottawa city garden; verbatimLatitude: 45.356; verbatimLongitude: -75.707; **Identification:** identifiedBy: Jose Fernandez-Triana; **Event:** verbatimEventDate: 26-Jun-2007; **Record Level:** institutionID: Canadian National Collection of Insects, Arachnids and Nematodes
- **Type status:** Other material. **Occurrence:** catalogNumber: BIOUG08665-D10; recordedBy: Anne Parkhill; lifeStage: Adult; otherCatalogNumbers: SMTPD4964-13; **Location:** country: Canada; stateProvince: Ontario; county: Paris; municipality: Holy Family School; locality: EQP-CLL-560; verbatimElevation: 250; verbatimLatitude: 43.207; verbatimLongitude: -80.391; **Event:** verbatimEventDate: 27-Sep-2013; **Record Level:** institutionID: Biodiversity Institute of Ontario
- **Type status:** Other material. **Occurrence:** catalogNumber: CAM0076; recordedBy: H. Goulet; lifeStage: Adult; otherCatalogNumbers: CNCHZ266-09; **Location:** country: Canada; stateProvince: Ontario; county: Ottawa; locality: Ottawa city garden; verbatimLatitude: 45.356; verbatimLongitude: -75.707; **Identification:** identifiedBy: Jose Fernandez-Triana; **Event:** verbatimEventDate: 26-Jun-2007; **Record Level:** institutionID: Canadian National Collection of Insects, Arachnids and Nematodes
- **Type status:** Other material. **Occurrence:** catalogNumber: BIOUG11771-G09; recordedBy: Cavan Harpur; lifeStage: Adult; otherCatalogNumbers: CNPKJ2482-14; **Location:** country: Canada; stateProvince: Ontario; county: Heron Bay; municipality: Pukaskwa National Park; verbatimElevation: 631; verbatimLatitude: 48.601; verbatimLongitude: -86.289; **Event:** verbatimEventDate: 26-Aug-2013; **Record Level:** institutionID: Biodiversity Institute of Ontario
- **Type status:** Other material. **Occurrence:** catalogNumber: BIOUG09807-D05; recordedBy: BIO Collections Staff; lifeStage: Adult; otherCatalogNumbers: MBIOK431-14; **Location:** country: Canada; stateProvince: Ontario; county: Wellington County; municipality: Guelph; locality: Biodiversity Institute of Ontario; verbatimElevation: 335; verbatimLatitude: 43.528; verbatimLongitude: -80.229; **Event:** verbatimEventDate: 19-Sep-2013; **Record Level:** institutionID: Biodiversity Institute of Ontario
- **Type status:** Other material. **Occurrence:** catalogNumber: CAM0530; recordedBy: A. Bennett; lifeStage: Adult; otherCatalogNumbers: CNCHZ1150-09; **Location:** country: Canada; stateProvince: Ontario; county: Ottawa; locality: Mixed forest near Manotick; verbatimLatitude: 45.235; verbatimLongitude: -75.624; **Identification:** identifiedBy: Jose Fernandez-Triana; **Record Level:** institutionID: Canadian National Collection of Insects, Arachnids and Nematodes
- **Type status:** Other material. **Occurrence:** catalogNumber: HYM00001021; recordedBy: Goulet & Boudreault; lifeStage: Adult; otherCatalogNumbers: ASWA307-08; **Location:** country: Canada; stateProvince: Manitoba; locality: Gardenton; verbatimElevation: 292; verbatimLatitude: 49.079; verbatimLongitude: -96.766; **Identification:** identifiedBy: Jose Fernandez-Triana; **Event:** verbatimEventDate: 11-Jun-2007; **Record Level:** institutionID: Canadian National Collection of Insects, Arachnids and Nematodes
- **Type status:** Other material. **Occurrence:** catalogNumber: CAM0528; recordedBy: A. Bennett; lifeStage: Adult; otherCatalogNumbers: CNCHZ1148-09; **Location:** country: Canada; stateProvince: Ontario; county: Ottawa; locality: Mixed forest near Manotick; verbatimLatitude: 45.235; verbatimLongitude: -75.624; **Identification:** identifiedBy: Jose Fernandez-Triana; **Record Level:** institutionID: Canadian National Collection of Insects, Arachnids and Nematodes
- **Type status:** Other material. **Occurrence:** catalogNumber: 10BBCHY-1304; recordedBy: BIObus 2010; lifeStage: Adult; otherCatalogNumbers: BBHYJ345-10; **Location:** country: Canada; stateProvince: Saskatchewan; county: Prince Albert NP; municipality: Boundary Bog Trl.; verbatimElevation: 568; verbatimLatitude: 53.906; verbatimLongitude: -106.030; **Identification:** identifiedBy: Julie K. Stahlhut; **Event:** verbatimEventDate: 13-Aug-2010; **Record Level:** institutionID: Biodiversity Institute of Ontario
- **Type status:** Other material. **Occurrence:** catalogNumber: CNCH1025; recordedBy: L. Masner; lifeStage: Adult; otherCatalogNumbers: CNCHX1040-09; **Location:** country: Canada; stateProvince: Ontario; county: Ottawa; locality: Ottwa city garden; verbatimLatitude: 45.356; verbatimLongitude: -75.707; **Identification:** identifiedBy: Jose Fernandez-Triana; **Event:** verbatimEventDate: 06-Jul-2009; **Record Level:** institutionID: Canadian National Collection of Insects, Arachnids and Nematodes
- **Type status:** Other material. **Occurrence:** catalogNumber: CAM0399; recordedBy: Boudreault, Goulet & Fernandez; lifeStage: Adult; otherCatalogNumbers: CNCHZ589-09; **Location:** country: Canada; stateProvince: Ontario; county: Manitoulin Island; locality: Misery Bay Prov. Park; verbatimElevation: 185; verbatimLatitude: 45.792; verbatimLongitude: -82.750; **Identification:** identifiedBy: Jose Fernandez-Triana; **Event:** verbatimEventDate: 03-Jun-2008; **Record Level:** institutionID: Canadian National Collection of Insects, Arachnids and Nematodes
- **Type status:** Other material. **Occurrence:** catalogNumber: BIOUG07902-D06; recordedBy: BIO Team; lifeStage: Adult; otherCatalogNumbers: RBINA3618-13; **Location:** country: Canada; stateProvince: Ontario; county: Rouge National Urban Park; locality: Sector 8; verbatimElevation: 167; verbatimLatitude: 43.859; verbatimLongitude: -79.209; **Event:** verbatimEventDate: 15-Sep-2013; **Record Level:** institutionID: Biodiversity Institute of Ontario
- **Type status:** Other material. **Occurrence:** catalogNumber: MIC 000089; sex: Female; lifeStage: Adult; otherCatalogNumbers: MHABR089-09; **Location:** country: Canada; stateProvince: Ontario; county: St. Lawrence Islands National Park; locality: Thwartway Island; **Identification:** identifiedBy: Jose Fernandez-Triana; **Event:** verbatimEventDate: 15-Aug-1976; **Record Level:** institutionID: Canadian National Collection of Insects, Arachnids and Nematodes
- **Type status:** Other material. **Occurrence:** catalogNumber: BIOUG01631-A05; recordedBy: James Sones; lifeStage: Adult; otherCatalogNumbers: JSAUG1436-11; **Location:** country: Canada; stateProvince: Ontario; county: Leeds and Grenville; municipality: Elizabethtown-Kitley; locality: 4452 Rowsome Rd., Elizabethtown; verbatimElevation: 111; verbatimLatitude: 44.618; verbatimLongitude: -75.775; **Identification:** identifiedBy: BOLD ID Engine; **Event:** verbatimEventDate: 18-Aug-2011; **Record Level:** institutionID: Biodiversity Institute of Ontario
- **Type status:** Other material. **Occurrence:** catalogNumber: CAM0941; recordedBy: H. Goulet, A. Badiss, C. Boudeault; lifeStage: Adult; otherCatalogNumbers: CNCHX071-09; **Location:** country: Canada; stateProvince: Newfoundland and Labrador; county: Corner Brook; locality: Corner Brook, fallow field; verbatimLatitude: 48.956; verbatimLongitude: -57.911; **Identification:** identifiedBy: Jose Fernandez-Triana; **Event:** verbatimEventDate: 17-Jul-2008; **Record Level:** institutionID: Canadian National Collection of Insects, Arachnids and Nematodes
- **Type status:** Other material. **Occurrence:** catalogNumber: CAM0961; recordedBy: H. Goulet, A. Badiss, C. Boudeault; lifeStage: Adult; otherCatalogNumbers: CNCHX091-09; **Location:** country: Canada; stateProvince: Newfoundland and Labrador; county: St-David`s; locality: St-David`s, fallow field; verbatimLatitude: 48.205; verbatimLongitude: -58.866; **Identification:** identifiedBy: Jose Fernandez-Triana; **Event:** verbatimEventDate: 10-Jul-2008; **Record Level:** institutionID: Canadian National Collection of Insects, Arachnids and Nematodes
- **Type status:** Other material. **Occurrence:** catalogNumber: BIOUG10641-G12; recordedBy: Chris Johnstone; lifeStage: Adult; otherCatalogNumbers: CNGBJ1514-14; **Location:** country: Canada; stateProvince: Ontario; county: Georgian Bay Islands National Park; municipality: Georgian Bay Islands National Park, Administration Office, 901 Wye Valley Rd.; locality: Marsh; verbatimElevation: 190; verbatimLatitude: 44.742; verbatimLongitude: -79.850; **Event:** verbatimEventDate: 23-May-2013; **Record Level:** institutionID: Biodiversity Institute of Ontario
- **Type status:** Other material. **Occurrence:** catalogNumber: BIOUG09631-C02; recordedBy: BIO Collections Staff; lifeStage: Adult; otherCatalogNumbers: MBIOI1284-13; **Location:** country: Canada; stateProvince: Ontario; county: Wellington County; municipality: Guelph; locality: Biodiversity Institute of Ontario; verbatimElevation: 335; verbatimLatitude: 43.528; verbatimLongitude: -80.229; **Event:** verbatimEventDate: 22-Aug-2013; **Record Level:** institutionID: Biodiversity Institute of Ontario
- **Type status:** Other material. **Occurrence:** catalogNumber: BIOUG14099-G10; recordedBy: Cavan Harpur; lifeStage: Adult; otherCatalogNumbers: CNPKO652-14; **Location:** country: Canada; stateProvince: Ontario; county: Heron Bay; municipality: Pukaskwa National Park; verbatimElevation: 631; verbatimLatitude: 48.601; verbatimLongitude: -86.289; **Identification:** identifiedBy: Angela Telfer; **Event:** verbatimEventDate: 01-Sep-2013; **Record Level:** institutionID: Biodiversity Institute of Ontario
- **Type status:** Other material. **Occurrence:** catalogNumber: BIOUG11758-F08; recordedBy: Matt Davis; lifeStage: Adult; otherCatalogNumbers: CNKJF1651-14; **Location:** country: Canada; stateProvince: Nova Scotia; county: Kejimkujik National Park; locality: Jeremy`s Bay Campground, near Ampitheater off of Campfire Circle; verbatimElevation: 116; verbatimLatitude: 44.407; verbatimLongitude: -65.245; **Event:** verbatimEventDate: 25-Jul-2013; **Record Level:** institutionID: Biodiversity Institute of Ontario
- **Type status:** Other material. **Occurrence:** catalogNumber: 10BBCHY-1305; recordedBy: BIObus 2010; lifeStage: Adult; otherCatalogNumbers: BBHYJ346-10; **Location:** country: Canada; stateProvince: Saskatchewan; county: Prince Albert NP; municipality: Boundary Bog Trl.; verbatimElevation: 568; verbatimLatitude: 53.906; verbatimLongitude: -106.030; **Identification:** identifiedBy: Julie K. Stahlhut; **Event:** verbatimEventDate: 13-Aug-2010; **Record Level:** institutionID: Biodiversity Institute of Ontario
- **Type status:** Other material. **Occurrence:** catalogNumber: CAM0522; recordedBy: A. Bennett; lifeStage: Adult; otherCatalogNumbers: CNCHZ1142-09; **Location:** country: Canada; stateProvince: Ontario; county: Ottawa; locality: Mixed forest near Manotick; verbatimLatitude: 45.235; verbatimLongitude: -75.624; **Identification:** identifiedBy: Jose Fernandez-Triana; **Record Level:** institutionID: Canadian National Collection of Insects, Arachnids and Nematodes
- **Type status:** Other material. **Occurrence:** catalogNumber: CAM0347; recordedBy: Goulet & Fernandez; lifeStage: Adult; otherCatalogNumbers: CNCHZ537-09; **Location:** country: Canada; stateProvince: Ontario; county: Almonte; locality: 5 km NW of Almonte, Hwy 49, Burnt Land, Alvar Prov. Park; verbatimLatitude: 45.255; verbatimLongitude: -76.140; **Identification:** identifiedBy: Jose Fernandez-Triana; **Event:** verbatimEventDate: 29-May-2008; **Record Level:** institutionID: Canadian National Collection of Insects, Arachnids and Nematodes
- **Type status:** Other material. **Occurrence:** catalogNumber: CNCHYM 00105; recordedBy: D. Shanek; lifeStage: Adult; otherCatalogNumbers: HYCND1815-11; **Location:** country: United States; stateProvince: Louisiana; county: Evangeline County; locality: Bayou Chicot; **Identification:** identifiedBy: Jose Fernandez-Triana; **Event:** verbatimEventDate: 23-Apr-1972; **Record Level:** institutionID: Canadian National Collection of Insects, Arachnids and Nematodes
- **Type status:** Other material. **Occurrence:** catalogNumber: BIOUG14170-E05; recordedBy: Cavan Harpur; lifeStage: Adult; otherCatalogNumbers: CNPKO2903-14; **Location:** country: Canada; stateProvince: Ontario; county: Heron Bay; municipality: Pukaskwa National Park; verbatimElevation: 631; verbatimLatitude: 48.601; verbatimLongitude: -86.289; **Identification:** identifiedBy: Angela Telfer; **Event:** verbatimEventDate: 01-Sep-2013; **Record Level:** institutionID: Biodiversity Institute of Ontario
- **Type status:** Other material. **Occurrence:** catalogNumber: CAM0054; recordedBy: H. Goulet; lifeStage: Adult; otherCatalogNumbers: CNCHZ244-09; **Location:** country: Canada; stateProvince: Ontario; county: Ottawa; locality: Ottawa city garden; verbatimLatitude: 45.356; verbatimLongitude: -75.707; **Identification:** identifiedBy: Jose Fernandez-Triana; **Event:** verbatimEventDate: 23-Jul-2007; **Record Level:** institutionID: Canadian National Collection of Insects, Arachnids and Nematodes
- **Type status:** Other material. **Occurrence:** catalogNumber: HYM00000665; lifeStage: Adult; otherCatalogNumbers: DSWAS984-07; **Location:** country: Canada; stateProvince: Manitoba; county: Churchill; verbatimLatitude: 58.740; verbatimLongitude: -93.820; **Identification:** identifiedBy: Jose Fernandez-Triana; **Event:** verbatimEventDate: 17-Aug-2006; **Record Level:** institutionID: Canadian National Collection of Insects, Arachnids and Nematodes
- **Type status:** Other material. **Occurrence:** catalogNumber: BIOUG08749-G01; recordedBy: Lydia Attard, Kyla Greenham; lifeStage: Adult; otherCatalogNumbers: CNROJ688-13; **Location:** country: Canada; stateProvince: Ontario; county: Toronto; municipality: Rouge National Urban Park, Trap 1; verbatimElevation: 136; verbatimLatitude: 43.822; verbatimLongitude: -79.190; **Event:** verbatimEventDate: 03-Sep-2013; **Record Level:** institutionID: Biodiversity Institute of Ontario
- **Type status:** Other material. **Occurrence:** catalogNumber: TDWG-0167; recordedBy: BIObus: A.Borisenko, R.Labbee, V.Levesque-Beaudin, M.Milton, J.Smith, S.Ratnasingham; lifeStage: Adult; otherCatalogNumbers: TDWGB052-10; **Location:** country: United States; stateProvince: Massachusetts; county: Barnstable Country; municipality: Woods Hole|near memoriall circle cottage #24; locality: Mixed deciduous; verbatimElevation: 16; verbatimLatitude: 41.530; verbatimLongitude: -70.655; **Event:** verbatimEventDate: 29-Sep-2010; **Record Level:** institutionID: Biodiversity Institute of Ontario
- **Type status:** Other material. **Occurrence:** catalogNumber: 07PROBE-23393; recordedBy: A.Renaud; lifeStage: Adult; otherCatalogNumbers: ASWAX959-08; **Location:** country: Canada; stateProvince: Manitoba; county: Churchill; municipality: 23 km E Churchill, Ramsay Creek; verbatimElevation: 10; verbatimLatitude: 58.731; verbatimLongitude: -93.780; **Identification:** identifiedBy: Jose Fernandez-Triana; **Event:** verbatimEventDate: 23-Jul-2007; **Record Level:** institutionID: Biodiversity Institute of Ontario
- **Type status:** Other material. **Occurrence:** catalogNumber: BIOUG07902-D03; recordedBy: BIO Team; lifeStage: Adult; otherCatalogNumbers: RBINA3615-13; **Location:** country: Canada; stateProvince: Ontario; county: Rouge National Urban Park; locality: Sector 8; verbatimElevation: 167; verbatimLatitude: 43.859; verbatimLongitude: -79.209; **Event:** verbatimEventDate: 15-Sep-2013; **Record Level:** institutionID: Biodiversity Institute of Ontario
- **Type status:** Other material. **Occurrence:** catalogNumber: CAM0099; recordedBy: H. Goulet; lifeStage: Adult; otherCatalogNumbers: CNCHZ289-09; **Location:** country: Canada; stateProvince: Ontario; county: Ottawa; locality: Ottawa city garden; verbatimLatitude: 45.356; verbatimLongitude: -75.707; **Identification:** identifiedBy: Jose Fernandez-Triana; **Event:** verbatimEventDate: 08-Sep-2007; **Record Level:** institutionID: Canadian National Collection of Insects, Arachnids and Nematodes
- **Type status:** Other material. **Occurrence:** catalogNumber: BIOUG08565-E09; recordedBy: Barb Maschke; lifeStage: Adult; otherCatalogNumbers: SMTPD1793-13; **Location:** country: Canada; stateProvince: Ontario; county: Perth East; municipality: Mornington Central School; locality: EQP-CLL-556; verbatimElevation: 380; verbatimLatitude: 43.614; verbatimLongitude: -80.885; **Event:** verbatimEventDate: 27-Sep-2013; **Record Level:** institutionID: Biodiversity Institute of Ontario
- **Type status:** Other material. **Occurrence:** catalogNumber: MIC 000666; recordedBy: Goulet & Fernandez; lifeStage: Adult; otherCatalogNumbers: CNCHZ858-09; **Location:** country: Canada; stateProvince: Ontario; county: Almonte; locality: 5 km NW of Almonte, Hwy 49, Burnt Land, Alvar Prov. Park; verbatimLatitude: 45.255; verbatimLongitude: -76.140; **Identification:** identifiedBy: Jose Fernandez-Triana; **Event:** verbatimEventDate: 29-May-2008; **Record Level:** institutionID: Canadian National Collection of Insects, Arachnids and Nematodes
- **Type status:** Other material. **Occurrence:** catalogNumber: CAM0146; recordedBy: H. Goulet; lifeStage: Adult; otherCatalogNumbers: CNCHZ336-09; **Location:** country: Canada; stateProvince: Ontario; county: Ottawa; locality: Ottawa city garden; verbatimLatitude: 45.356; verbatimLongitude: -75.707; **Identification:** identifiedBy: Jose Fernandez-Triana; **Event:** verbatimEventDate: 01-Sep-2007; **Record Level:** institutionID: Canadian National Collection of Insects, Arachnids and Nematodes
- **Type status:** Other material. **Occurrence:** catalogNumber: BIOUG09801-C03; recordedBy: BIO Collections Staff; lifeStage: Adult; otherCatalogNumbers: MBIOJ817-13; **Location:** country: Canada; stateProvince: Ontario; county: Wellington County; municipality: Guelph; locality: Biodiversity Institute of Ontario; verbatimElevation: 335; verbatimLatitude: 43.528; verbatimLongitude: -80.229; **Event:** verbatimEventDate: 05-Sep-2013; **Record Level:** institutionID: Biodiversity Institute of Ontario
- **Type status:** Other material. **Occurrence:** catalogNumber: MIC 000488; recordedBy: H. Goulet, A. Badiss, C. Boudeault; lifeStage: Adult; otherCatalogNumbers: CNCHZ680-09; **Location:** country: Canada; stateProvince: Prince Edward Island; locality: Blooming Point, fallow field; verbatimElevation: 6; verbatimLatitude: 46.408; verbatimLongitude: -62.951; **Identification:** identifiedBy: Jose Fernandez-Triana; **Event:** verbatimEventDate: 23-Jul-2008; **Record Level:** institutionID: Canadian National Collection of Insects, Arachnids and Nematodes
- **Type status:** Other material. **Occurrence:** catalogNumber: BIOUG01572-D01; recordedBy: J. Cossey; lifeStage: Adult; otherCatalogNumbers: NCCC037-11; **Location:** country: Canada; stateProvince: Ontario; municipality: Carden Alvar; locality: Prairie Smoke; verbatimLatitude: 44.645; verbatimLongitude: -79.095; **Event:** verbatimEventDate: 06-Oct-2011; **Record Level:** institutionID: Biodiversity Institute of Ontario
- **Type status:** Other material. **Occurrence:** catalogNumber: HYM00001025; recordedBy: Goulet & Boudreault; lifeStage: Adult; otherCatalogNumbers: ASWA311-08; **Location:** country: Canada; stateProvince: Manitoba; locality: Gardenton; verbatimElevation: 292; verbatimLatitude: 49.079; verbatimLongitude: -96.766; **Identification:** identifiedBy: Jose Fernandez-Triana; **Event:** verbatimEventDate: 11-Jun-2007; **Record Level:** institutionID: Canadian National Collection of Insects, Arachnids and Nematodes
- **Type status:** Other material. **Occurrence:** catalogNumber: CAM0072; recordedBy: H. Goulet; lifeStage: Adult; otherCatalogNumbers: CNCHZ262-09; **Location:** country: Canada; stateProvince: Ontario; county: Ottawa; locality: Ottawa city garden; verbatimLatitude: 45.356; verbatimLongitude: -75.707; **Identification:** identifiedBy: Jose Fernandez-Triana; **Event:** verbatimEventDate: 26-Jun-2007; **Record Level:** institutionID: Canadian National Collection of Insects, Arachnids and Nematodes
- **Type status:** Other material. **Occurrence:** catalogNumber: CAM0024; recordedBy: H. Goulet; lifeStage: Adult; otherCatalogNumbers: CNCHZ214-09; **Location:** country: Canada; stateProvince: Ontario; county: Ottawa; locality: Ottawa city garden; verbatimLatitude: 45.356; verbatimLongitude: -75.707; **Identification:** identifiedBy: Jose Fernandez-Triana; **Event:** verbatimEventDate: 30-Jul-2007; **Record Level:** institutionID: Canadian National Collection of Insects, Arachnids and Nematodes
- **Type status:** Other material. **Occurrence:** catalogNumber: BIOUG11726-F01; recordedBy: J. Howard, C. Johnstone; lifeStage: Adult; otherCatalogNumbers: CNGBN2850-14; **Location:** country: Canada; stateProvince: Ontario; county: Georgian Bay Islands National Park; municipality: Beausoleil Island, Cedar Spring Campground; locality: Mixed forest; verbatimElevation: 177; verbatimLatitude: 44.852; verbatimLongitude: -79.874; **Event:** verbatimEventDate: 19-Aug-2013; **Record Level:** institutionID: Biodiversity Institute of Ontario
- **Type status:** Other material. **Occurrence:** catalogNumber: BIOUG12460-D06; recordedBy: Park Staff; lifeStage: Adult; otherCatalogNumbers: CNMIG137-14; **Location:** country: Canada; stateProvince: Quebec; county: Mingan Archipelago National Park Reserve; municipality: Quarry Island; locality: bog near forest edge; verbatimLatitude: 50.214; verbatimLongitude: -63.798; **Identification:** identifiedBy: Angela Telfer; **Event:** verbatimEventDate: 06-Aug-2013; **Record Level:** institutionID: Biodiversity Institute of Ontario
- **Type status:** Other material. **Occurrence:** catalogNumber: BIOUG09930-G04; recordedBy: BIO Collections Staff; lifeStage: Adult; otherCatalogNumbers: MBION363-14; **Location:** country: Canada; stateProvince: Ontario; county: Wellington County; municipality: Guelph; locality: Biodiversity Institute of Ontario; verbatimElevation: 335; verbatimLatitude: 43.528; verbatimLongitude: -80.229; **Event:** verbatimEventDate: 04-Oct-2013; **Record Level:** institutionID: Biodiversity Institute of Ontario
- **Type status:** Other material. **Occurrence:** catalogNumber: BIOUG03863-E06; recordedBy: Thousand Islands NP; lifeStage: Adult; otherCatalogNumbers: CNSLH124-12; **Location:** country: Canada; stateProvince: Ontario; county: Thousand Islands NP; municipality: Jones Creek by Mallorytown, County Rd. 5; verbatimElevation: 117; verbatimLatitude: 44.475; verbatimLongitude: -75.865; **Event:** verbatimEventDate: 05-Aug-2012; **Record Level:** institutionID: Biodiversity Institute of Ontario
- **Type status:** Other material. **Occurrence:** catalogNumber: CAM0039; recordedBy: H. Goulet; lifeStage: Adult; otherCatalogNumbers: CNCHZ229-09; **Location:** country: Canada; stateProvince: Ontario; county: Ottawa; locality: Ottawa city garden; verbatimLatitude: 45.356; verbatimLongitude: -75.707; **Identification:** identifiedBy: Jose Fernandez-Triana; **Event:** verbatimEventDate: 10-Aug-2007; **Record Level:** institutionID: Canadian National Collection of Insects, Arachnids and Nematodes
- **Type status:** Other material. **Occurrence:** catalogNumber: CAM0409; recordedBy: Goulet, Boudreault & Fernandez; lifeStage: Adult; otherCatalogNumbers: CNCHZ599-09; **Location:** country: Canada; stateProvince: Ontario; county: Manitoulin Island; locality: Little Current, prairie alvar; verbatimLatitude: 45.975; verbatimLongitude: -81.904; **Identification:** identifiedBy: Jose Fernandez-Triana; **Event:** verbatimEventDate: 04-Jun-2008; **Record Level:** institutionID: Canadian National Collection of Insects, Arachnids and Nematodes

##### Notes

This species is widely distributed in central and eastern North America. It was recorded from four provinces in Canada (MB, NS, ON, QC) by Fernandez-Triana (2010), but no locality records were provided at that time. Recently four more Canadian provinces and territories (AB, NB, NT, SK) and one state (AK) from the United States were added (Fernandez-Triana et al. 2014). Here we present additional information about the distribution of this species in the Nearctic, including two new provincial records for Canada (NL, PE) and one state (FL) from the United States, based on examined specimens from the BIO and CNC collections. Data for those specimens was extracted from BOLD.

The specimens of *A. ensiger* that rendered DNA barcodes comprise two BINS, BOLD:ACE6783 (ON, MB) and BOLD:AAA3764 (AB, ON, SK and some localities of southern US) (Suppl. material 4); whether they represent two different species or not will be studied in an upcoming paper.

#### Apanteles sodalis

(Haliday, 1834)

##### Materials

- **Type status:** Other material. **Occurrence:** catalogNumber: CBRA0492; recordedBy: J. Cossentine; lifeStage: Adult; otherCatalogNumbers: CNCHV584-10; **Location:** country: Canada; stateProvince: British Columbia; locality: 11 km S of Cawston; verbatimElevation: 303; **Identification:** identifiedBy: Jose Fernandez-Triana; **Event:** verbatimEventDate: 10-Jun-1998; **Record Level:** institutionID: Canadian National Collection of Insects, Arachnids and Nematodes
- **Type status:** Other material. **Occurrence:** catalogNumber: CBRA0493; recordedBy: H. Fry; sex: Female; lifeStage: Adult; otherCatalogNumbers: CNCHV585-10; **Location:** country: Canada; stateProvince: Newfoundland and Labrador; locality: St. John`s; **Identification:** identifiedBy: Jose Fernandez-Triana; **Event:** verbatimEventDate: 05-Aug-2004; **Record Level:** institutionID: Canadian National Collection of Insects, Arachnids and Nematodes
- **Type status:** Other material. **Occurrence:** catalogNumber: CNCHYM 00215; recordedBy: Doganlar; lifeStage: Adult; otherCatalogNumbers: HYCNE025-11; **Location:** country: Canada; stateProvince: British Columbia; locality: Chilliwack; **Identification:** identifiedBy: Jose Fernandez-Triana; **Event:** verbatimEventDate: 27-Jun-1978; **Record Level:** institutionID: Canadian National Collection of Insects, Arachnids and Nematodes
- **Type status:** Other material. **Occurrence:** catalogNumber: CBRA0491; recordedBy: J. Cossentine; lifeStage: Adult; otherCatalogNumbers: CNCHV583-10; **Location:** country: Canada; stateProvince: British Columbia; locality: 11 km S of Cawston; verbatimElevation: 303; **Identification:** identifiedBy: Jose Fernandez-Triana; **Record Level:** institutionID: Canadian National Collection of Insects, Arachnids and Nematodes
- **Type status:** Other material. **Occurrence:** catalogNumber: MIC 000009; recordedBy: T. Renault; sex: Female; lifeStage: Adult; otherCatalogNumbers: MHABR009-09; **Location:** country: Canada; stateProvince: New Brunswick; locality: SE of Upsalq; **Identification:** identifiedBy: Jose Fernandez-Triana; **Event:** verbatimEventDate: 19-Jul-1961; **Record Level:** institutionID: Canadian National Collection of Insects, Arachnids and Nematodes
- **Type status:** Other material. **Occurrence:** catalogNumber: CBRA0490; recordedBy: J. Cossentine; lifeStage: Adult; otherCatalogNumbers: CNCHV582-10; **Location:** country: Canada; stateProvince: British Columbia; locality: 11 km S of Cawston; verbatimElevation: 303; **Identification:** identifiedBy: Jose Fernandez-Triana; **Event:** verbatimEventDate: 14-May-1998; **Record Level:** institutionID: Canadian National Collection of Insects, Arachnids and Nematodes
- **Type status:** Other material. **Occurrence:** catalogNumber: MIC 000007; recordedBy: F. Peters; sex: Female; lifeStage: Adult; otherCatalogNumbers: MHABR007-09; **Location:** country: Canada; stateProvince: British Columbia; locality: Burnaby; **Identification:** identifiedBy: Jose Fernandez-Triana; **Event:** verbatimEventDate: 07-May-1977; **Record Level:** institutionID: Canadian National Collection of Insects, Arachnids and Nematodes
- **Type status:** Other material. **Occurrence:** catalogNumber: MIC 000013; recordedBy: H. Fry; sex: Female; lifeStage: Adult; otherCatalogNumbers: MHABR013-09; **Location:** country: Canada; stateProvince: Newfoundland and Labrador; locality: St. John`s; **Identification:** identifiedBy: Jose Fernandez-Triana; **Event:** verbatimEventDate: 05-Aug-2004; **Record Level:** institutionID: Canadian National Collection of Insects, Arachnids and Nematodes
- **Type status:** Other material. **Occurrence:** catalogNumber: MIC 000010; recordedBy: T. Renault; sex: Female; lifeStage: Adult; otherCatalogNumbers: MHABR010-09; **Location:** country: Canada; stateProvince: New Brunswick; locality: Green River Lab.; **Identification:** identifiedBy: Jose Fernandez-Triana; **Event:** verbatimEventDate: 24-Jul-1961; **Record Level:** institutionID: Canadian National Collection of Insects, Arachnids and Nematodes
- **Type status:** Other material. **Occurrence:** catalogNumber: MIC 000011; recordedBy: J. Cossentine; sex: Female; lifeStage: Adult; otherCatalogNumbers: MHABR011-09; **Location:** country: Canada; stateProvince: British Columbia; locality: 11 km S of Cawston; verbatimElevation: 303; **Identification:** identifiedBy: Jose Fernandez-Triana; **Event:** verbatimEventDate: 14-May-1998; **Record Level:** institutionID: Canadian National Collection of Insects, Arachnids and Nematodes
- **Type status:** Other material. **Occurrence:** catalogNumber: MIC 000014; recordedBy: H. Fry; sex: Male; lifeStage: Adult; otherCatalogNumbers: MHABR014-09; **Location:** country: Canada; stateProvince: Newfoundland and Labrador; locality: St. John`s; **Identification:** identifiedBy: Jose Fernandez-Triana; **Event:** verbatimEventDate: 05-Aug-2004; **Record Level:** institutionID: Canadian National Collection of Insects, Arachnids and Nematodes
- **Type status:** Other material. **Occurrence:** catalogNumber: MIC 000005; recordedBy: Docanlar; sex: Female; lifeStage: Adult; otherCatalogNumbers: MHABR005-09; **Location:** country: Canada; stateProvince: British Columbia; locality: Burnaby; **Identification:** identifiedBy: Jose Fernandez-Triana; **Event:** verbatimEventDate: 07-May-1977; **Record Level:** institutionID: Canadian National Collection of Insects, Arachnids and Nematodes
- **Type status:** Other material. **Occurrence:** catalogNumber: CBRA0495; recordedBy: H. Fry; sex: Female; lifeStage: Adult; otherCatalogNumbers: CNCHV587-10; **Location:** country: Canada; stateProvince: Newfoundland and Labrador; locality: St. John`s; **Identification:** identifiedBy: Jose Fernandez-Triana; **Event:** verbatimEventDate: 05-Aug-2004; **Record Level:** institutionID: Canadian National Collection of Insects, Arachnids and Nematodes
- **Type status:** Other material. **Occurrence:** catalogNumber: CBRA0494; recordedBy: H. Fry; sex: Female; lifeStage: Adult; otherCatalogNumbers: CNCHV586-10; **Location:** country: Canada; stateProvince: Newfoundland and Labrador; locality: St. John`s; **Identification:** identifiedBy: Jose Fernandez-Triana; **Event:** verbatimEventDate: 05-Aug-2004; **Record Level:** institutionID: Canadian National Collection of Insects, Arachnids and Nematodes
- **Type status:** Other material. **Occurrence:** catalogNumber: MIC 000012; recordedBy: J. Cossentine; sex: Male; lifeStage: Adult; otherCatalogNumbers: MHABR012-09; **Location:** country: Canada; stateProvince: British Columbia; locality: 11 km S of Cawston; verbatimElevation: 303; **Identification:** identifiedBy: Jose Fernandez-Triana; **Event:** verbatimEventDate: 13-Jul-1998; **Record Level:** institutionID: Canadian National Collection of Insects, Arachnids and Nematodes
- **Type status:** Other material. **Occurrence:** catalogNumber: MIC 000006; recordedBy: Doganlar; sex: Male; lifeStage: Adult; otherCatalogNumbers: MHABR006-09; **Location:** country: Canada; stateProvince: British Columbia; locality: Burnaby; **Identification:** identifiedBy: Jose Fernandez-Triana; **Event:** verbatimEventDate: 07-May-1977; **Record Level:** institutionID: Canadian National Collection of Insects, Arachnids and Nematodes
- **Type status:** Other material. **Occurrence:** catalogNumber: MIC 000008; recordedBy: F. Peters; sex: Male; lifeStage: Adult; otherCatalogNumbers: MHABR008-09; **Location:** country: Canada; stateProvince: British Columbia; locality: Summerland; **Identification:** identifiedBy: Jose Fernandez-Triana; **Event:** verbatimEventDate: 02-Jun-1978; **Record Level:** institutionID: Canadian National Collection of Insects, Arachnids and Nematodes

##### Notes

This species is widely distributed in the Holarctic and it was recorded from three provinces in Canada (BC, NB, NL) by Fernandez-Triana (2010), but no locality records were provided at the time. Here we present new information about the distribution of this species in Canada, based on examined specimens from the BIO and CNC collections. Data for those specimens was extracted from BOLD.

The specimens of *A. sodalis* that rendered DNA barcodes comprise two BINS: BOLD:AAM7223 (BC, NL) and BOLD:AAN1859 (BC) (Suppl. material 4); whether they represent two different species or not will be studied in an upcoming paper.

#### Apanteles xanthostigma

(Haliday, 1834)

##### Materials

- **Type status:** Other material. **Occurrence:** catalogNumber: BIOUG10637-H02; recordedBy: Anderson; lifeStage: Adult; otherCatalogNumbers: CNGMI700-14; **Location:** country: Canada; stateProvince: Newfoundland and Labrador; county: Gros Morne National Park; municipality: James Callaghan Trail (aka Gros Morne Trail); locality: mature conifer stand, balsam fir, wind damage; verbatimElevation: 39; verbatimLatitude: 49.569; verbatimLongitude: -57.830; **Event:** verbatimEventDate: 17-Sep-2013; **Record Level:** institutionID: Biodiversity Institute of Ontario
- **Type status:** Other material. **Occurrence:** catalogNumber: 07PROBE-22321; recordedBy: A.Renaud; lifeStage: Adult; otherCatalogNumbers: ASWAV862-08; **Location:** country: Canada; stateProvince: Manitoba; county: Churchill; municipality: 16 km SW Churchill, Churchill River Pump House; verbatimLatitude: 58.626; verbatimLongitude: -94.229; **Identification:** identifiedBy: Jose Fernandez-Triana; **Event:** verbatimEventDate: 12-Jul-2007; **Record Level:** institutionID: Biodiversity Institute of Ontario
- **Type status:** Other material. **Occurrence:** catalogNumber: BIOUG04486-G10; recordedBy: Rod Thompson, Eric Knight; lifeStage: Adult; otherCatalogNumbers: CNPAD006-13; **Location:** country: Canada; stateProvince: Saskatchewan; county: Prince Albert NP; verbatimElevation: 627; verbatimLatitude: 53.850; verbatimLongitude: -106.078; **Identification:** identifiedBy: Jose Fernandez-Triana; **Event:** verbatimEventDate: 10-Jul-2012; **Record Level:** institutionID: Biodiversity Institute of Ontario
- **Type status:** Other material. **Occurrence:** catalogNumber: MIC 000638; recordedBy: Goulet & Boudreault; lifeStage: Adult; otherCatalogNumbers: CNCHZ830-09; **Location:** country: Canada; stateProvince: British Columbia; county: Sorrento; locality: Sorrento, fallow field; verbatimLatitude: 50.779; verbatimLongitude: -119.502; **Identification:** identifiedBy: Jose Fernandez-Triana; **Event:** verbatimEventDate: 28-May-2005; **Record Level:** institutionID: Canadian National Collection of Insects, Arachnids and Nematodes
- **Type status:** Other material. **Occurrence:** catalogNumber: 07PROBE-20921; lifeStage: Adult; otherCatalogNumbers: ASWAT273-08; **Location:** country: Canada; stateProvince: Manitoba; county: Churchill; municipality: 16 km SW Churchill, Churchill River Pump House; verbatimLatitude: 58.626; verbatimLongitude: -94.229; **Identification:** identifiedBy: Jose Fernandez-Triana; **Event:** verbatimEventDate: 07-Jul-2007; **Record Level:** institutionID: Biodiversity Institute of Ontario

##### Notes

Previously, this species was known to be widely distributed in the Palaearctic, with one record from tropical Africa (Yu et al. 2012). It was recently recorded from three provinces in Canada (BC, MB, SK) by Fernandez-Triana et al. (2014). A new provincial record (NL) is added here, altogether with detailed information about the distribution of this species in Canada, based on examined specimens from the BIO and CNC collections. Data for those specimens was extracted from BOLD.

#### Cotesia crambi

(Weed, 1887)

##### Materials

- **Type status:** Other material. **Occurrence:** catalogNumber: CNCHYM 00401; recordedBy: Mailloux; lifeStage: Adult; otherCatalogNumbers: HYCNE211-11; **Location:** country: United States; stateProvince: New Jersey; locality: Metuchen; **Identification:** identifiedBy: Jose Fernandez-Triana; **Event:** verbatimEventDate: 19-Sep-1973; **Record Level:** institutionID: Canadian National Collection of Insects, Arachnids and Nematodes
- **Type status:** Other material. **Occurrence:** catalogNumber: BIOUG08665-E05; recordedBy: Anne Parkhill; lifeStage: Adult; otherCatalogNumbers: SMTPD4971-13; **Location:** country: Canada; stateProvince: Ontario; county: Paris; municipality: Holy Family School; locality: EQP-CLL-560; verbatimElevation: 250; verbatimLatitude: 43.207; verbatimLongitude: -80.391; **Identification:** identifiedBy: Angela Telfer; **Event:** verbatimEventDate: 27-Sep-2013; **Record Level:** institutionID: Biodiversity Institute of Ontario
- **Type status:** Other material. **Occurrence:** catalogNumber: BIOUG01258-H06; recordedBy: James Sones; lifeStage: Adult; otherCatalogNumbers: JSHYO850-11; **Location:** country: Canada; stateProvince: Ontario; county: Leeds and Grenville; municipality: Elizabethtown-Kitley; locality: 4452 Rowsome Rd., Elizabethtown; verbatimElevation: 112; verbatimLatitude: 44.621; verbatimLongitude: -75.773; **Identification:** identifiedBy: Angela Telfer; **Event:** verbatimEventDate: 15-Aug-2010; **Record Level:** institutionID: Biodiversity Institute of Ontario
- **Type status:** Other material. **Occurrence:** catalogNumber: CAM0542; recordedBy: A. Bennett; lifeStage: Adult; otherCatalogNumbers: CNCHZ1162-09; **Location:** country: Canada; stateProvince: Ontario; county: Ottawa; locality: Mixed forest near Manotick; verbatimLatitude: 45.235; verbatimLongitude: -75.624; **Identification:** identifiedBy: Jose Fernandez-Triana; **Record Level:** institutionID: Canadian National Collection of Insects, Arachnids and Nematodes
- **Type status:** Other material. **Occurrence:** catalogNumber: BIOUG08742-H10; recordedBy: BIO Collections Staff; lifeStage: Adult; otherCatalogNumbers: MBIOE759-13; **Location:** country: Canada; stateProvince: Ontario; county: Wellington County; municipality: Guelph; locality: Biodiversity Institute of Ontario; verbatimElevation: 335; verbatimLatitude: 43.528; verbatimLongitude: -80.229; **Identification:** identifiedBy: Angela Telfer; **Event:** verbatimEventDate: 27-Jun-2013; **Record Level:** institutionID: Biodiversity Institute of Ontario
- **Type status:** Other material. **Occurrence:** catalogNumber: CNCHYM 00404; recordedBy: H. T. Streu; lifeStage: Adult; otherCatalogNumbers: HYCNE214-11; **Location:** country: United States; stateProvince: New Jersey; locality: Metuchen, Red Fescue; **Identification:** identifiedBy: Jose Fernandez-Triana; **Event:** verbatimEventDate: 01-Jul-1974; **Record Level:** institutionID: Canadian National Collection of Insects, Arachnids and Nematodes
- **Type status:** Other material. **Occurrence:** catalogNumber: CNCHYM 00403; lifeStage: Adult; otherCatalogNumbers: HYCNE213-11; **Location:** country: United States; stateProvince: New Jersey; locality: Metuchen; **Identification:** identifiedBy: Jose Fernandez-Triana; **Event:** verbatimEventDate: 19-Sep-1973; **Record Level:** institutionID: Canadian National Collection of Insects, Arachnids and Nematodes
- **Type status:** Other material. **Occurrence:** catalogNumber: CNCHYM 00402; lifeStage: Adult; otherCatalogNumbers: HYCNE212-11; **Location:** country: United States; stateProvince: New Jersey; locality: Metuchen; **Identification:** identifiedBy: Jose Fernandez-Triana; **Event:** verbatimEventDate: 07-Sep-1973; **Record Level:** institutionID: Canadian National Collection of Insects, Arachnids and Nematodes
- **Type status:** Other material. **Occurrence:** catalogNumber: CAM1034; recordedBy: H. Goulet; lifeStage: Adult; otherCatalogNumbers: CNCHX164-09; **Location:** country: Canada; stateProvince: Ontario; county: Ottawa; locality: Ottwa city garden; verbatimLatitude: 45.356; verbatimLongitude: -75.707; **Identification:** identifiedBy: Jose Fernandez-Triana; **Record Level:** institutionID: Canadian National Collection of Insects, Arachnids and Nematodes
- **Type status:** Other material. **Occurrence:** catalogNumber: BIOUG03438-B06; recordedBy: Heidi Brown; lifeStage: Adult; otherCatalogNumbers: CNPPD2118-12; **Location:** country: Canada; stateProvince: Ontario; county: Point Pelee NP; municipality: Cactus Field; verbatimElevation: 168; verbatimLatitude: 41.939; verbatimLongitude: -82.516; **Identification:** identifiedBy: Angela Telfer; **Event:** verbatimEventDate: 15-Jun-2012; **Record Level:** institutionID: Biodiversity Institute of Ontario
- **Type status:** Other material. **Occurrence:** catalogNumber: CAM0893; recordedBy: H. Goulet; lifeStage: Adult; otherCatalogNumbers: CNCHX023-09; **Location:** country: Canada; stateProvince: Ontario; county: Ottawa; locality: Ottwa city garden; verbatimLatitude: 45.356; verbatimLongitude: -75.707; **Identification:** identifiedBy: Jose Fernandez-Triana; **Record Level:** institutionID: Canadian National Collection of Insects, Arachnids and Nematodes
- **Type status:** Other material. **Occurrence:** catalogNumber: BIOUG03445-F01; recordedBy: Tyler Peters; lifeStage: Adult; otherCatalogNumbers: CNPPE479-12; **Location:** country: Canada; stateProvince: Ontario; county: Point Pelee NP; municipality: Cactus Field; verbatimElevation: 168; verbatimLatitude: 41.939; verbatimLongitude: -82.516; **Identification:** identifiedBy: Angela Telfer; **Event:** verbatimEventDate: 29-Jun-2012; **Record Level:** institutionID: Biodiversity Institute of Ontario

##### Notes

This species has been recorded mainly from northeast and central United States (Yu et al. 2012, Fernandez-Triana et al. 2014). Until now it was only known from two localities in Canada (ON, QC) (Fernandez-Triana 2010, Fernandez-Triana et al. 2014). Here we present new and detailed information about four additional localities in ON, based on examined specimens from the BIO and CNC collections. Data for those specimens was extracted from BOLD.

#### Dolichogenidea longicauda

(Wesmael, 1837)

##### Materials

- **Type status:** Other material. **Occurrence:** catalogNumber: CNCHYM 01091; recordedBy: Doganlar; lifeStage: Adult; otherCatalogNumbers: HYCNE902-11; **Location:** country: Canada; stateProvince: British Columbia; locality: Burnaby; **Identification:** identifiedBy: Jose Fernandez-Triana; **Event:** verbatimEventDate: 09-Jul-1978; **Record Level:** institutionID: Canadian National Collection of Insects, Arachnids and Nematodes
- **Type status:** Other material. **Occurrence:** catalogNumber: CNCHYM 01093; lifeStage: Adult; otherCatalogNumbers: HYCNE904-11; **Location:** country: Canada; stateProvince: British Columbia; locality: New Westminster; **Identification:** identifiedBy: Jose Fernandez-Triana; **Event:** verbatimEventDate: 05-May-1978; **Record Level:** institutionID: Canadian National Collection of Insects, Arachnids and Nematodes
- **Type status:** Other material. **Occurrence:** catalogNumber: CNCHYM 01089; recordedBy: Doganlar; lifeStage: Adult; otherCatalogNumbers: HYCNE900-11; **Location:** country: Canada; stateProvince: British Columbia; locality: Burnaby; **Identification:** identifiedBy: Jose Fernandez-Triana; **Event:** verbatimEventDate: 01-Jul-1977; **Record Level:** institutionID: Canadian National Collection of Insects, Arachnids and Nematodes
- **Type status:** Other material. **Occurrence:** catalogNumber: CNCHYM 00216; recordedBy: Doganlar; lifeStage: Adult; otherCatalogNumbers: HYCNE026-11; **Location:** country: Canada; stateProvince: British Columbia; locality: Burnaby; **Identification:** identifiedBy: Jose Fernandez-Triana; **Event:** verbatimEventDate: 01-Jul-1977; **Record Level:** institutionID: Canadian National Collection of Insects, Arachnids and Nematodes

##### Notes

This species is widely distributed in the Palaearctic (Yu et al. 2012). It was recorded from one province in Canada (BC) by Fernandez-Triana (2010), Fernández-Triana and Huber (2010), but no locality records were provided at the time. Here we present new information about the distribution of this species in Canada (BC), as well as the first record of this species from the United States (WA) based on examined specimens from the BIO and CNC collections. Data for those specimens was extracted from BOLD.

#### Hygroplitis melligaster

(Provancher, 1886)

##### Materials

- **Type status:** Other material. **Occurrence:** catalogNumber: CNCHYM 01363; recordedBy: D. B. Lyons; lifeStage: Adult; otherCatalogNumbers: HYCNE1175-11; **Location:** country: Canada; stateProvince: New Brunswick; locality: Kouchibouguac N.P.; **Identification:** identifiedBy: Jose Fernandez-Triana; **Event:** verbatimEventDate: 18-Aug-1978; **Record Level:** institutionID: Canadian National Collection of Insects, Arachnids and Nematodes
- **Type status:** Other material. **Occurrence:** catalogNumber: CNCHYM 01366; recordedBy: W. R. Richards; lifeStage: Adult; otherCatalogNumbers: HYCNE1178-11; **Location:** country: United States; stateProvince: North Carolina; locality: Wayah Bald; verbatimElevation: 1249.7; **Identification:** identifiedBy: Jose Fernandez-Triana; **Event:** verbatimEventDate: 29-Jul-1957; **Record Level:** institutionID: Canadian National Collection of Insects, Arachnids and Nematodes
- **Type status:** Other material. **Occurrence:** catalogNumber: CNCHYM 01375; recordedBy: W. Mason; lifeStage: Adult; otherCatalogNumbers: HYCNE1187-11; **Location:** country: Canada; stateProvince: Ontario; locality: Kemptville; **Identification:** identifiedBy: Jose Fernandez-Triana; **Event:** verbatimEventDate: 28-Jul-1983; **Record Level:** institutionID: Canadian National Collection of Insects, Arachnids and Nematodes
- **Type status:** Other material. **Occurrence:** catalogNumber: GOU 0314; recordedBy: Sharkey & Read; lifeStage: Adult; otherCatalogNumbers: CNCBA789-10; **Location:** country: Canada; stateProvince: Ontario; locality: 2 km SW of Innisville; **Identification:** identifiedBy: Jose Fernandez-Triana; **Event:** verbatimEventDate: 26-Jun-1991; **Record Level:** institutionID: Canadian National Collection of Insects, Arachnids and Nematodes
- **Type status:** Other material. **Occurrence:** catalogNumber: CNCHYM 01365; recordedBy: J. R. Vockeroth; lifeStage: Adult; otherCatalogNumbers: HYCNE1177-11; **Location:** country: Canada; stateProvince: Nova Scotia; locality: Lockeport; **Identification:** identifiedBy: Jose Fernandez-Triana; **Event:** verbatimEventDate: 09-Aug-1958; **Record Level:** institutionID: Canadian National Collection of Insects, Arachnids and Nematodes
- **Type status:** Other material. **Occurrence:** catalogNumber: CAM0473; recordedBy: H. Goulet, A. Badiss, C. Boudeault; lifeStage: Adult; otherCatalogNumbers: CNCHZ663-09; **Location:** country: Canada; stateProvince: Prince Edward Island; locality: Ten Mile House, salt marsh; verbatimLatitude: 46.313; verbatimLongitude: -62.981; **Identification:** identifiedBy: Jose Fernandez-Triana; **Event:** verbatimEventDate: 23-Jul-2008; **Record Level:** institutionID: Canadian National Collection of Insects, Arachnids and Nematodes
- **Type status:** Other material. **Occurrence:** catalogNumber: CNCHYM 01369; recordedBy: G. E. Shewell; lifeStage: Adult; otherCatalogNumbers: HYCNE1181-11; **Location:** country: Canada; stateProvince: New Brunswick; locality: St. Andrews; **Identification:** identifiedBy: Jose Fernandez-Triana; **Event:** verbatimEventDate: 08-Aug-1959; **Record Level:** institutionID: Canadian National Collection of Insects, Arachnids and Nematodes
- **Type status:** Other material. **Occurrence:** catalogNumber: CAM0470; recordedBy: H. Goulet, A. Badiss, C. Boudeault; lifeStage: Adult; otherCatalogNumbers: CNCHZ660-09; **Location:** country: Canada; stateProvince: Prince Edward Island; locality: Ten Mile House, salt marsh; verbatimLatitude: 46.313; verbatimLongitude: -62.981; **Identification:** identifiedBy: Jose Fernandez-Triana; **Event:** verbatimEventDate: 23-Jul-2008; **Record Level:** institutionID: Canadian National Collection of Insects, Arachnids and Nematodes
- **Type status:** Other material. **Occurrence:** catalogNumber: CAM0471; recordedBy: H. Goulet, A. Badiss, C. Boudeault; lifeStage: Adult; otherCatalogNumbers: CNCHZ661-09; **Location:** country: Canada; stateProvince: Prince Edward Island; locality: Ten Mile House, salt marsh; verbatimLatitude: 46.313; verbatimLongitude: -62.981; **Identification:** identifiedBy: Jose Fernandez-Triana; **Event:** verbatimEventDate: 23-Jul-2008; **Record Level:** institutionID: Canadian National Collection of Insects, Arachnids and Nematodes
- **Type status:** Other material. **Occurrence:** catalogNumber: CAM0468; recordedBy: H. Goulet, A. Badiss, C. Boudeault; lifeStage: Adult; otherCatalogNumbers: CNCHZ658-09; **Location:** country: Canada; stateProvince: Prince Edward Island; locality: Ten Mile House, salt marsh; verbatimLatitude: 46.313; verbatimLongitude: -62.981; **Identification:** identifiedBy: Jose Fernandez-Triana; **Event:** verbatimEventDate: 23-Jul-2008; **Record Level:** institutionID: Canadian National Collection of Insects, Arachnids and Nematodes
- **Type status:** Other material. **Occurrence:** catalogNumber: CNCHYM 01370; recordedBy: J. G. Chillcott; lifeStage: Adult; otherCatalogNumbers: HYCNE1182-11; **Location:** country: United States; stateProvince: North Carolina; locality: Highlands; verbatimElevation: 1158.25; **Identification:** identifiedBy: Jose Fernandez-Triana; **Event:** verbatimEventDate: 12-Jul-1957; **Record Level:** institutionID: Canadian National Collection of Insects, Arachnids and Nematodes
- **Type status:** Other material. **Occurrence:** catalogNumber: CAM0463; recordedBy: H. Goulet, A. Badiss, C. Boudeault; lifeStage: Adult; otherCatalogNumbers: CNCHZ653-09; **Location:** country: Canada; stateProvince: Prince Edward Island; locality: Ten Mile House, salt marsh; verbatimLatitude: 46.313; verbatimLongitude: -62.981; **Identification:** identifiedBy: Jose Fernandez-Triana; **Event:** verbatimEventDate: 23-Jul-2008; **Record Level:** institutionID: Canadian National Collection of Insects, Arachnids and Nematodes
- **Type status:** Other material. **Occurrence:** catalogNumber: CAM0465; recordedBy: H. Goulet, A. Badiss, C. Boudeault; lifeStage: Adult; otherCatalogNumbers: CNCHZ655-09; **Location:** country: Canada; stateProvince: Prince Edward Island; locality: Ten Mile House, salt marsh; verbatimLatitude: 46.313; verbatimLongitude: -62.981; **Identification:** identifiedBy: Jose Fernandez-Triana; **Event:** verbatimEventDate: 23-Jul-2008; **Record Level:** institutionID: Canadian National Collection of Insects, Arachnids and Nematodes
- **Type status:** Other material. **Occurrence:** catalogNumber: CAM0464; recordedBy: H. Goulet, A. Badiss, C. Boudeault; lifeStage: Adult; otherCatalogNumbers: CNCHZ654-09; **Location:** country: Canada; stateProvince: Prince Edward Island; locality: Ten Mile House, salt marsh; verbatimLatitude: 46.313; verbatimLongitude: -62.981; **Identification:** identifiedBy: Jose Fernandez-Triana; **Event:** verbatimEventDate: 23-Jul-2008; **Record Level:** institutionID: Canadian National Collection of Insects, Arachnids and Nematodes
- **Type status:** Other material. **Occurrence:** catalogNumber: CNCHYM 01368; recordedBy: R. B. Madge; lifeStage: Adult; otherCatalogNumbers: HYCNE1180-11; **Location:** country: Canada; stateProvince: Manitoba; locality: Brandon; **Identification:** identifiedBy: Jose Fernandez-Triana; **Event:** verbatimEventDate: 14-Aug-1958; **Record Level:** institutionID: Canadian National Collection of Insects, Arachnids and Nematodes
- **Type status:** Other material. **Occurrence:** catalogNumber: CNCHYM 01367; recordedBy: McMillan; lifeStage: Adult; otherCatalogNumbers: HYCNE1179-11; **Location:** country: Canada; stateProvince: Ontario; county: St. Lawrence Islands National Park; locality: Grenadier Island Centre; **Identification:** identifiedBy: Jose Fernandez-Triana; **Event:** verbatimEventDate: 29-Jul-1975; **Record Level:** institutionID: Canadian National Collection of Insects, Arachnids and Nematodes
- **Type status:** Other material. **Occurrence:** catalogNumber: CAM0462; recordedBy: H. Goulet, A. Badiss, C. Boudeault; lifeStage: Adult; otherCatalogNumbers: CNCHZ652-09; **Location:** country: Canada; stateProvince: Prince Edward Island; locality: Ten Mile House, salt marsh; verbatimLatitude: 46.313; verbatimLongitude: -62.981; **Identification:** identifiedBy: Jose Fernandez-Triana; **Event:** verbatimEventDate: 23-Jul-2008; **Record Level:** institutionID: Canadian National Collection of Insects, Arachnids and Nematodes
- **Type status:** Other material. **Occurrence:** catalogNumber: CNCHYM 01371; recordedBy: J. R. Vockeroth; lifeStage: Adult; otherCatalogNumbers: HYCNE1183-11; **Location:** country: United States; stateProvince: New York; locality: Paul Smiths, Franklin Co.; **Identification:** identifiedBy: Jose Fernandez-Triana; **Event:** verbatimEventDate: 20-Jul-1962; **Record Level:** institutionID: Canadian National Collection of Insects, Arachnids and Nematodes
- **Type status:** Other material. **Occurrence:** catalogNumber: CAM0469; recordedBy: H. Goulet, A. Badiss, C. Boudeault; lifeStage: Adult; otherCatalogNumbers: CNCHZ659-09; **Location:** country: Canada; stateProvince: Prince Edward Island; locality: Ten Mile House, salt marsh; verbatimLatitude: 46.313; verbatimLongitude: -62.981; **Identification:** identifiedBy: Jose Fernandez-Triana; **Event:** verbatimEventDate: 23-Jul-2008; **Record Level:** institutionID: Canadian National Collection of Insects, Arachnids and Nematodes
- **Type status:** Other material. **Occurrence:** catalogNumber: CNCHYM 01376; lifeStage: Adult; otherCatalogNumbers: HYCNE1188-11; **Location:** country: Canada; stateProvince: Ontario; locality: Aylmer West; **Identification:** identifiedBy: Jose Fernandez-Triana; **Event:** verbatimEventDate: 22-Jul-1972; **Record Level:** institutionID: Canadian National Collection of Insects, Arachnids and Nematodes
- **Type status:** Other material. **Occurrence:** catalogNumber: CNCHYM 01364; recordedBy: W. R. M. Mason; lifeStage: Adult; otherCatalogNumbers: HYCNE1176-11; **Location:** country: Canada; stateProvince: Ontario; locality: Stittsville; **Identification:** identifiedBy: Jose Fernandez-Triana; **Event:** verbatimEventDate: 22-Aug-1963; **Record Level:** institutionID: Canadian National Collection of Insects, Arachnids and Nematodes
- **Type status:** Other material. **Occurrence:** catalogNumber: CNCHYM 01377; lifeStage: Adult; otherCatalogNumbers: HYCNE1189-11; **Location:** country: Canada; stateProvince: Ontario; locality: Rondeau Prov. Pk.; **Identification:** identifiedBy: Jose Fernandez-Triana; **Event:** verbatimEventDate: 31-Jul-1972; **Record Level:** institutionID: Canadian National Collection of Insects, Arachnids and Nematodes
- **Type status:** Other material. **Occurrence:** catalogNumber: CNCHYM 01378; recordedBy: L. Dumouchel; lifeStage: Adult; otherCatalogNumbers: HYCNE1190-11; **Location:** country: Canada; stateProvince: Ontario; county: Ottawa; locality: Mer Bleue; **Identification:** identifiedBy: Jose Fernandez-Triana; **Event:** verbatimEventDate: 09-Jul-1982; **Record Level:** institutionID: Canadian National Collection of Insects, Arachnids and Nematodes
- **Type status:** Other material. **Occurrence:** catalogNumber: BIOUG01082-E12; recordedBy: James Sones; lifeStage: Adult; otherCatalogNumbers: JSHYN833-11; **Location:** country: Canada; stateProvince: Ontario; county: Leeds and Grenville; municipality: Elizabethtown-Kitley; locality: 4452 Rowsome Rd., Elizabethtown; verbatimElevation: 112; verbatimLatitude: 44.621; verbatimLongitude: -75.773; **Identification:** identifiedBy: Jose Fernandez-Triana; **Event:** verbatimEventDate: 17-Jul-2010; **Record Level:** institutionID: Biodiversity Institute of Ontario
- **Type status:** Other material. **Occurrence:** catalogNumber: CAM0474; recordedBy: H. Goulet, A. Badiss, C. Boudeault; lifeStage: Adult; otherCatalogNumbers: CNCHZ664-09; **Location:** country: Canada; stateProvince: Prince Edward Island; locality: Ten Mile House, salt marsh; verbatimLatitude: 46.313; verbatimLongitude: -62.981; **Identification:** identifiedBy: Jose Fernandez-Triana; **Event:** verbatimEventDate: 23-Jul-2008; **Record Level:** institutionID: Canadian National Collection of Insects, Arachnids and Nematodes
- **Type status:** Other material. **Occurrence:** catalogNumber: CAM0466; recordedBy: H. Goulet, A. Badiss, C. Boudeault; lifeStage: Adult; otherCatalogNumbers: CNCHZ656-09; **Location:** country: Canada; stateProvince: Prince Edward Island; locality: Ten Mile House, salt marsh; verbatimLatitude: 46.313; verbatimLongitude: -62.981; **Identification:** identifiedBy: Jose Fernandez-Triana; **Event:** verbatimEventDate: 23-Jul-2008; **Record Level:** institutionID: Canadian National Collection of Insects, Arachnids and Nematodes
- **Type status:** Other material. **Occurrence:** catalogNumber: CAM0472; recordedBy: H. Goulet, A. Badiss, C. Boudeault; lifeStage: Adult; otherCatalogNumbers: CNCHZ662-09; **Location:** country: Canada; stateProvince: Prince Edward Island; locality: Ten Mile House, salt marsh; verbatimLatitude: 46.313; verbatimLongitude: -62.981; **Identification:** identifiedBy: Jose Fernandez-Triana; **Event:** verbatimEventDate: 23-Jul-2008; **Record Level:** institutionID: Canadian National Collection of Insects, Arachnids and Nematodes
- **Type status:** Other material. **Occurrence:** catalogNumber: CAM0467; recordedBy: H. Goulet, A. Badiss, C. Boudeault; lifeStage: Adult; otherCatalogNumbers: CNCHZ657-09; **Location:** country: Canada; stateProvince: Prince Edward Island; locality: Ten Mile House, salt marsh; verbatimLatitude: 46.313; verbatimLongitude: -62.981; **Identification:** identifiedBy: Jose Fernandez-Triana; **Event:** verbatimEventDate: 23-Jul-2008; **Record Level:** institutionID: Canadian National Collection of Insects, Arachnids and Nematodes

##### Notes

This species is relatively widely distributed in northeast North America, extending up to central Canada (Yu et al. 2012). It was recorded from five provinces in Canada (MB, NB, NS, ON, QC) by Fernandez-Triana (2010), but no locality records were provided at the time. Here we present additional information about the distribution of the species in Canada, including one new provincial record (PE), based on examined specimens from the BIO and CNC collections. Data for those specimens was extracted from BOLD.

#### Pholetesor bedelliae

(Viereck, 1911)

##### Materials

- **Type status:** Other material. **Occurrence:** catalogNumber: CAM0278; recordedBy: H. Goulet; lifeStage: Adult; otherCatalogNumbers: CNCHZ468-09; **Location:** country: Canada; stateProvince: Ontario; county: Ottawa; locality: Ottawa city garden; verbatimLatitude: 45.356; verbatimLongitude: -75.707; **Identification:** identifiedBy: Jose Fernandez-Triana; **Event:** verbatimEventDate: 30-May-2007; **Record Level:** institutionID: Canadian National Collection of Insects, Arachnids and Nematodes
- **Type status:** Other material. **Occurrence:** catalogNumber: BIOUG04442-A12; recordedBy: BIOBus 2012; lifeStage: Adult; otherCatalogNumbers: SSBAD5451-13; **Location:** country: Canada; stateProvince: Alberta; county: Banff NP; municipality: Storm Mountain; locality: Adjacent to train track and Bow River; verbatimElevation: 1462; verbatimLatitude: 51.282; verbatimLongitude: -115.944; **Event:** verbatimEventDate: 25-Jul-2012; **Record Level:** institutionID: Biodiversity Institute of Ontario
- **Type status:** Other material. **Occurrence:** catalogNumber: HYM00000958; recordedBy: Goulet & Boudrealt; lifeStage: Adult; otherCatalogNumbers: DSWAS929-07; **Location:** country: Canada; stateProvince: Yukon Territory; locality: Fish Lake road; verbatimElevation: 1107; verbatimLatitude: 60.688; verbatimLongitude: -135.238; **Identification:** identifiedBy: Jose Fernandez-Triana; **Event:** verbatimEventDate: 05-Jul-2006; **Record Level:** institutionID: Canadian National Collection of Insects, Arachnids and Nematodes
- **Type status:** Other material. **Occurrence:** catalogNumber: CNCH3205; recordedBy: Goulet & Bouderault; lifeStage: Adult; otherCatalogNumbers: CNCHV275-10; **Location:** country: United States; stateProvince: Alaska; locality: Anderson, Hwy 3; verbatimElevation: 235; verbatimLatitude: 64.295; verbatimLongitude: -149.084; **Identification:** identifiedBy: Jose Fernandez-Triana; **Event:** verbatimEventDate: 18-Jul-2009; **Record Level:** institutionID: Canadian National Collection of Insects, Arachnids and Nematodes
- **Type status:** Other material. **Occurrence:** catalogNumber: HYM00000818; lifeStage: Adult; otherCatalogNumbers: DSWAS761-07; **Location:** country: Canada; stateProvince: Manitoba; county: Churchill; locality: 26 km SE Churchill, Twin Lakes Burn site; verbatimElevation: 38; verbatimLatitude: 58.617; verbatimLongitude: -93.812; **Identification:** identifiedBy: Jose Fernandez-Triana; **Event:** verbatimEventDate: 19-Aug-2006; **Record Level:** institutionID: Canadian National Collection of Insects, Arachnids and Nematodes
- **Type status:** Other material. **Occurrence:** catalogNumber: CNCH3212; recordedBy: Goulet & Bouderault; lifeStage: Adult; otherCatalogNumbers: CNCHV282-10; **Location:** country: United States; stateProvince: Alaska; locality: Anderson, Hwy 3; verbatimElevation: 235; verbatimLatitude: 64.295; verbatimLongitude: -149.084; **Identification:** identifiedBy: Jose Fernandez-Triana; **Event:** verbatimEventDate: 18-Jul-2009; **Record Level:** institutionID: Canadian National Collection of Insects, Arachnids and Nematodes
- **Type status:** Other material. **Occurrence:** catalogNumber: BIOUG07357-A10; recordedBy: Chrystal Simon; lifeStage: Adult; otherCatalogNumbers: NGNAC1789-13; **Location:** country: Canada; stateProvince: British Columbia; county: 10 km W Kamloops; municipality: New Afton Mine; locality: Wetland, Site 1; verbatimElevation: 759; verbatimLatitude: 50.643; verbatimLongitude: -120.516; **Event:** verbatimEventDate: 11-Jul-2013; **Record Level:** institutionID: Biodiversity Institute of Ontario
- **Type status:** Other material. **Occurrence:** catalogNumber: HYM00000994; recordedBy: Goulet & Boudrealt; lifeStage: Adult; otherCatalogNumbers: DSWAS965-07; **Location:** country: Canada; stateProvince: Yukon Territory; locality: Pelly Crossing; verbatimElevation: 495; verbatimLatitude: 62.831; verbatimLongitude: -136.585; **Identification:** identifiedBy: Jose Fernandez-Triana; **Event:** verbatimEventDate: 16-Jul-2006; **Record Level:** institutionID: Canadian National Collection of Insects, Arachnids and Nematodes
- **Type status:** Other material. **Occurrence:** catalogNumber: HYM00000974; recordedBy: Goulet & Boudrealt; lifeStage: Adult; otherCatalogNumbers: DSWAS945-07; **Location:** country: Canada; stateProvince: Yukon Territory; locality: Champagne; verbatimElevation: 733; verbatimLatitude: 60.790; verbatimLongitude: -136.437; **Identification:** identifiedBy: Jose Fernandez-Triana; **Event:** verbatimEventDate: 08-Jul-2006; **Record Level:** institutionID: Canadian National Collection of Insects, Arachnids and Nematodes
- **Type status:** Other material. **Occurrence:** catalogNumber: HYM00000953; recordedBy: Goulet & Boudrealt; lifeStage: Adult; otherCatalogNumbers: DSWAS924-07; **Location:** country: Canada; stateProvince: Yukon Territory; locality: Pelly Crossing; verbatimElevation: 495; verbatimLatitude: 62.831; verbatimLongitude: -136.585; **Identification:** identifiedBy: Jose Fernandez-Triana; **Event:** verbatimEventDate: 15-Jul-2006; **Record Level:** institutionID: Canadian National Collection of Insects, Arachnids and Nematodes
- **Type status:** Other material. **Occurrence:** catalogNumber: HYM00000821.2; recordedBy: R.Gwatkin; lifeStage: Adult; otherCatalogNumbers: ASWAY564-08; **Location:** country: Canada; stateProvince: Manitoba; county: Churchill; locality: unspecified locality; verbatimLatitude: 58.740; verbatimLongitude: -93.820; **Identification:** identifiedBy: Jose Fernandez-Triana; **Event:** verbatimEventDate: 17-Jul-1949; **Record Level:** institutionID: Canadian National Collection of Insects, Arachnids and Nematodes
- **Type status:** Other material. **Occurrence:** catalogNumber: CNCHYM 03136; recordedBy: F. I. S.; lifeStage: Adult; otherCatalogNumbers: HYCNE1821-11; **Location:** country: Canada; stateProvince: Ontario; locality: Faleyet; **Identification:** identifiedBy: J. Whitfield; **Event:** verbatimEventDate: 07-Sep-1963; **Record Level:** institutionID: Canadian National Collection of Insects, Arachnids and Nematodes
- **Type status:** Other material. **Occurrence:** catalogNumber: HYM00000821; recordedBy: R.Gwatkin; lifeStage: Adult; otherCatalogNumbers: DSWAS764-07; **Location:** country: Canada; stateProvince: Manitoba; county: Churchill; locality: unspecified locality; verbatimLatitude: 58.740; verbatimLongitude: -93.820; **Identification:** identifiedBy: Jose Fernandez-Triana; **Event:** verbatimEventDate: 17-Jul-1949; **Record Level:** institutionID: Canadian National Collection of Insects, Arachnids and Nematodes
- **Type status:** Other material. **Occurrence:** catalogNumber: HYM00000941; recordedBy: Goulet & Boudrealt; lifeStage: Adult; otherCatalogNumbers: DSWAS912-07; **Location:** country: Canada; stateProvince: Yukon Territory; locality: Champagne; verbatimElevation: 733; verbatimLatitude: 60.790; verbatimLongitude: -136.437; **Identification:** identifiedBy: Jose Fernandez-Triana; **Event:** verbatimEventDate: 08-Jul-2006; **Record Level:** institutionID: Canadian National Collection of Insects, Arachnids and Nematodes
- **Type status:** Other material. **Occurrence:** catalogNumber: CNCH3209; recordedBy: Goulet & Bouderault; lifeStage: Adult; otherCatalogNumbers: CNCHV279-10; **Location:** country: United States; stateProvince: Alaska; locality: Anderson, Hwy 3; verbatimElevation: 235; verbatimLatitude: 64.295; verbatimLongitude: -149.084; **Identification:** identifiedBy: Jose Fernandez-Triana; **Event:** verbatimEventDate: 18-Jul-2009; **Record Level:** institutionID: Canadian National Collection of Insects, Arachnids and Nematodes
- **Type status:** Other material. **Occurrence:** catalogNumber: CNCH3215; recordedBy: Goulet & Bouderault; lifeStage: Adult; otherCatalogNumbers: CNCHV380-10; **Location:** country: United States; stateProvince: Alaska; locality: Sutton on Glen Highway; verbatimElevation: 188; verbatimLatitude: 61.711; verbatimLongitude: -148.910; **Identification:** identifiedBy: Jose Fernandez-Triana; **Event:** verbatimEventDate: 22-Jul-2009; **Record Level:** institutionID: Canadian National Collection of Insects, Arachnids and Nematodes
- **Type status:** Other material. **Occurrence:** catalogNumber: CNCHYM 03143; recordedBy: F. I. S.; lifeStage: Adult; otherCatalogNumbers: HYCNE1828-11; **Location:** country: Canada; stateProvince: British Columbia; locality: 5 Miles West of Lumby; **Identification:** identifiedBy: J. Whitfield; **Event:** verbatimEventDate: 26-May-1958; **Record Level:** institutionID: Canadian National Collection of Insects, Arachnids and Nematodes
- **Type status:** Other material. **Occurrence:** catalogNumber: HYM00000996; recordedBy: Goulet & Boudrealt; lifeStage: Adult; otherCatalogNumbers: DSWAS967-07; **Location:** country: Canada; stateProvince: Yukon Territory; locality: Pelly Crossing; verbatimElevation: 495; verbatimLatitude: 62.831; verbatimLongitude: -136.585; **Identification:** identifiedBy: Jose Fernandez-Triana; **Event:** verbatimEventDate: 16-Jul-2006; **Record Level:** institutionID: Canadian National Collection of Insects, Arachnids and Nematodes
- **Type status:** Other material. **Occurrence:** catalogNumber: CNCH0955; recordedBy: Goulet & Boudreault; lifeStage: Adult; otherCatalogNumbers: CNCHX970-09; **Location:** country: United States; stateProvince: Alaska; locality: Delta Junction; verbatimElevation: 315; verbatimLatitude: 64.044; verbatimLongitude: -145.524; **Identification:** identifiedBy: Jose Fernandez-Triana; **Event:** verbatimEventDate: 20-Jul-2009; **Record Level:** institutionID: Canadian National Collection of Insects, Arachnids and Nematodes
- **Type status:** Other material. **Occurrence:** catalogNumber: HYM00000948; recordedBy: Goulet & Boudrealt; lifeStage: Adult; otherCatalogNumbers: DSWAS919-07; **Location:** country: Canada; stateProvince: Yukon Territory; locality: Pelly Crossing; verbatimElevation: 495; verbatimLatitude: 62.831; verbatimLongitude: -136.585; **Identification:** identifiedBy: Jose Fernandez-Triana; **Event:** verbatimEventDate: 15-Jul-2006; **Record Level:** institutionID: Canadian National Collection of Insects, Arachnids and Nematodes
- **Type status:** Other material. **Occurrence:** catalogNumber: CNCH3208; recordedBy: Goulet & Bouderault; lifeStage: Adult; otherCatalogNumbers: CNCHV278-10; **Location:** country: United States; stateProvince: Alaska; locality: Anderson, Hwy 3; verbatimElevation: 235; verbatimLatitude: 64.295; verbatimLongitude: -149.084; **Identification:** identifiedBy: Jose Fernandez-Triana; **Event:** verbatimEventDate: 18-Jul-2009; **Record Level:** institutionID: Canadian National Collection of Insects, Arachnids and Nematodes
- **Type status:** Other material. **Occurrence:** catalogNumber: HYM00000986; recordedBy: Goulet & Boudrealt; lifeStage: Adult; otherCatalogNumbers: DSWAS957-07; **Location:** country: Canada; stateProvince: Yukon Territory; locality: Champagne; verbatimElevation: 733; verbatimLatitude: 60.790; verbatimLongitude: -136.437; **Identification:** identifiedBy: Jose Fernandez-Triana; **Event:** verbatimEventDate: 08-Jul-2006; **Record Level:** institutionID: Canadian National Collection of Insects, Arachnids and Nematodes
- **Type status:** Other material. **Occurrence:** catalogNumber: HYM00000820; recordedBy: J.G.Chillcot; lifeStage: Adult; otherCatalogNumbers: DSWAS763-07; **Location:** country: Canada; stateProvince: Manitoba; county: Churchill; locality: Mile 505, Hudson Bay Railway; **Identification:** identifiedBy: Jose Fernandez-Triana; **Event:** verbatimEventDate: 16-Jul-1952; **Record Level:** institutionID: Canadian National Collection of Insects, Arachnids and Nematodes
- **Type status:** Other material. **Occurrence:** catalogNumber: HYM00000971; recordedBy: Goulet & Boudrealt; lifeStage: Adult; otherCatalogNumbers: DSWAS942-07; **Location:** country: Canada; stateProvince: Yukon Territory; locality: Champagne; verbatimElevation: 733; verbatimLatitude: 60.790; verbatimLongitude: -136.437; **Identification:** identifiedBy: Jose Fernandez-Triana; **Event:** verbatimEventDate: 08-Jul-2006; **Record Level:** institutionID: Canadian National Collection of Insects, Arachnids and Nematodes
- **Type status:** Other material. **Occurrence:** catalogNumber: HYM00000955; recordedBy: Goulet & Boudrealt; lifeStage: Adult; otherCatalogNumbers: DSWAS926-07; **Location:** country: Canada; stateProvince: Yukon Territory; locality: Pelly Crossing; verbatimElevation: 495; verbatimLatitude: 62.831; verbatimLongitude: -136.585; **Identification:** identifiedBy: Jose Fernandez-Triana; **Event:** verbatimEventDate: 15-Jul-2006; **Record Level:** institutionID: Canadian National Collection of Insects, Arachnids and Nematodes
- **Type status:** Other material. **Occurrence:** catalogNumber: CNCH3306; recordedBy: Goulet & Bouderault; lifeStage: Adult; otherCatalogNumbers: CNCHV375-10; **Location:** country: United States; stateProvince: Alaska; locality: Sutton on Glen Highway; verbatimElevation: 188; verbatimLatitude: 61.711; verbatimLongitude: -148.910; **Identification:** identifiedBy: Jose Fernandez-Triana; **Event:** verbatimEventDate: 22-Jul-2009; **Record Level:** institutionID: Canadian National Collection of Insects, Arachnids and Nematodes
- **Type status:** Other material. **Occurrence:** catalogNumber: HYM00000972; recordedBy: Goulet & Boudrealt; lifeStage: Adult; otherCatalogNumbers: DSWAS943-07; **Location:** country: Canada; stateProvince: Yukon Territory; locality: Champagne; verbatimElevation: 733; verbatimLatitude: 60.790; verbatimLongitude: -136.437; **Identification:** identifiedBy: Jose Fernandez-Triana; **Event:** verbatimEventDate: 08-Jul-2006; **Record Level:** institutionID: Canadian National Collection of Insects, Arachnids and Nematodes
- **Type status:** Other material. **Occurrence:** catalogNumber: HYM00000947; recordedBy: Goulet & Boudrealt; lifeStage: Adult; otherCatalogNumbers: DSWAS918-07; **Location:** country: Canada; stateProvince: Yukon Territory; locality: Pelly Crossing; verbatimElevation: 495; verbatimLatitude: 62.831; verbatimLongitude: -136.585; **Identification:** identifiedBy: Jose Fernandez-Triana; **Event:** verbatimEventDate: 15-Jul-2006; **Record Level:** institutionID: Canadian National Collection of Insects, Arachnids and Nematodes
- **Type status:** Other material. **Occurrence:** catalogNumber: CNCH3310; recordedBy: Goulet & Bouderault; lifeStage: Adult; otherCatalogNumbers: CNCHV379-10; **Location:** country: United States; stateProvince: Alaska; locality: Sutton on Glen Highway; verbatimElevation: 188; verbatimLatitude: 61.711; verbatimLongitude: -148.910; **Identification:** identifiedBy: Jose Fernandez-Triana; **Event:** verbatimEventDate: 22-Jul-2009; **Record Level:** institutionID: Canadian National Collection of Insects, Arachnids and Nematodes
- **Type status:** Other material. **Occurrence:** catalogNumber: HYM00000931; recordedBy: Goulet & Boudrealt; lifeStage: Adult; otherCatalogNumbers: DSWAS902-07; **Location:** country: Canada; stateProvince: Yukon Territory; locality: Pelly Crossing; verbatimElevation: 495; verbatimLatitude: 62.831; verbatimLongitude: -136.585; **Identification:** identifiedBy: Jose Fernandez-Triana; **Event:** verbatimEventDate: 15-Jul-2006; **Record Level:** institutionID: Canadian National Collection of Insects, Arachnids and Nematodes
- **Type status:** Other material. **Occurrence:** catalogNumber: HYM00000985; recordedBy: Goulet & Boudrealt; lifeStage: Adult; otherCatalogNumbers: DSWAS956-07; **Location:** country: Canada; stateProvince: Yukon Territory; locality: Champagne; verbatimElevation: 733; verbatimLatitude: 60.790; verbatimLongitude: -136.437; **Identification:** identifiedBy: Jose Fernandez-Triana; **Event:** verbatimEventDate: 08-Jul-2006; **Record Level:** institutionID: Canadian National Collection of Insects, Arachnids and Nematodes
- **Type status:** Other material. **Occurrence:** catalogNumber: CNCH3203; recordedBy: Goulet & Bouderault; lifeStage: Adult; otherCatalogNumbers: CNCHV273-10; **Location:** country: United States; stateProvince: Alaska; locality: Anderson, Hwy 3; verbatimElevation: 235; verbatimLatitude: 64.295; verbatimLongitude: -149.084; **Identification:** identifiedBy: Jose Fernandez-Triana; **Event:** verbatimEventDate: 18-Jul-2009; **Record Level:** institutionID: Canadian National Collection of Insects, Arachnids and Nematodes
- **Type status:** Other material. **Occurrence:** catalogNumber: CNCHYM 03141; recordedBy: H. B. Poole; lifeStage: Adult; otherCatalogNumbers: HYCNE1826-11; **Location:** country: United States; stateProvince: California; locality: Fallen Leaf, Eldorado co.; verbatimElevation: 1981.22; **Identification:** identifiedBy: Jose Fernandez-Triana; **Event:** verbatimEventDate: 13-Jul-1961; **Record Level:** institutionID: Canadian National Collection of Insects, Arachnids and Nematodes
- **Type status:** Other material. **Occurrence:** catalogNumber: CNCH0995; recordedBy: Goulet & Boudreault; lifeStage: Adult; otherCatalogNumbers: CNCHX1010-09; **Location:** country: United States; stateProvince: Alaska; locality: Delta Junction; verbatimElevation: 315; verbatimLatitude: 64.044; verbatimLongitude: -145.524; **Identification:** identifiedBy: Jose Fernandez-Triana; **Event:** verbatimEventDate: 20-Jul-2009; **Record Level:** institutionID: Canadian National Collection of Insects, Arachnids and Nematodes
- **Type status:** Other material. **Occurrence:** catalogNumber: HYM00000331; recordedBy: Boudreault & Goulet; lifeStage: Adult; otherCatalogNumbers: CNCHY059-07; **Location:** country: Canada; stateProvince: Yukon Territory; locality: Whitehorse, fallow field; verbatimElevation: 689; verbatimLatitude: 60.710; verbatimLongitude: -135.055; **Identification:** identifiedBy: Jose Fernandez-Triana; **Event:** verbatimEventDate: 10-Jul-2006; **Record Level:** institutionID: Canadian National Collection of Insects, Arachnids and Nematodes
- **Type status:** Other material. **Occurrence:** catalogNumber: HYM00000939; recordedBy: Goulet & Boudrealt; lifeStage: Adult; otherCatalogNumbers: DSWAS910-07; **Location:** country: Canada; stateProvince: Yukon Territory; locality: Champagne; verbatimElevation: 733; verbatimLatitude: 60.790; verbatimLongitude: -136.437; **Identification:** identifiedBy: Jose Fernandez-Triana; **Event:** verbatimEventDate: 08-Jul-2006; **Record Level:** institutionID: Canadian National Collection of Insects, Arachnids and Nematodes
- **Type status:** Other material. **Occurrence:** catalogNumber: HYM00000969; recordedBy: Goulet & Boudrealt; lifeStage: Adult; otherCatalogNumbers: DSWAS940-07; **Location:** country: Canada; stateProvince: Yukon Territory; locality: Champagne; verbatimElevation: 733; verbatimLatitude: 60.790; verbatimLongitude: -136.437; **Identification:** identifiedBy: Jose Fernandez-Triana; **Event:** verbatimEventDate: 08-Jul-2006; **Record Level:** institutionID: Canadian National Collection of Insects, Arachnids and Nematodes
- **Type status:** Other material. **Occurrence:** catalogNumber: CNCH0993; recordedBy: Goulet & Boudreault; lifeStage: Adult; otherCatalogNumbers: CNCHX1008-09; **Location:** country: United States; stateProvince: Alaska; locality: Delta Junction; verbatimElevation: 315; verbatimLatitude: 64.044; verbatimLongitude: -145.524; **Identification:** identifiedBy: Jose Fernandez-Triana; **Event:** verbatimEventDate: 20-Jul-2009; **Record Level:** institutionID: Canadian National Collection of Insects, Arachnids and Nematodes
- **Type status:** Other material. **Occurrence:** catalogNumber: CNCHYM 03145; recordedBy: C. M. Yoshimoto; lifeStage: Adult; otherCatalogNumbers: HYCNE1830-11; **Location:** country: Canada; stateProvince: New Brunswick; locality: Fredericton; **Identification:** identifiedBy: J. Whitfield; **Event:** verbatimEventDate: 09-Jul-1970; **Record Level:** institutionID: Canadian National Collection of Insects, Arachnids and Nematodes
- **Type status:** Other material. **Occurrence:** catalogNumber: HYM00000819; lifeStage: Adult; otherCatalogNumbers: DSWAS762-07; **Location:** country: Canada; stateProvince: Manitoba; county: Churchill; locality: 26 km SE Churchill, Twin Lakes Burn site; verbatimElevation: 38; verbatimLatitude: 58.617; verbatimLongitude: -93.812; **Identification:** identifiedBy: Jose Fernandez-Triana; **Event:** verbatimEventDate: 19-Aug-2006; **Record Level:** institutionID: Canadian National Collection of Insects, Arachnids and Nematodes
- **Type status:** Other material. **Occurrence:** catalogNumber: CNCHYM 03138; recordedBy: F. I. S.; lifeStage: Adult; otherCatalogNumbers: HYCNE1823-11; **Location:** country: Canada; stateProvince: British Columbia; locality: Langford; **Identification:** identifiedBy: J. Whitfield; **Event:** verbatimEventDate: 22-Jun-1962; **Record Level:** institutionID: Canadian National Collection of Insects, Arachnids and Nematodes
- **Type status:** Other material. **Occurrence:** catalogNumber: CNCH3204; recordedBy: Goulet & Bouderault; lifeStage: Adult; otherCatalogNumbers: CNCHV274-10; **Location:** country: United States; stateProvince: Alaska; locality: Anderson, Hwy 3; verbatimElevation: 235; verbatimLatitude: 64.295; verbatimLongitude: -149.084; **Identification:** identifiedBy: Jose Fernandez-Triana; **Event:** verbatimEventDate: 18-Jul-2009; **Record Level:** institutionID: Canadian National Collection of Insects, Arachnids and Nematodes
- **Type status:** Other material. **Occurrence:** catalogNumber: CNCH3213; recordedBy: Goulet & Bouderault; lifeStage: Adult; otherCatalogNumbers: CNCHV283-10; **Location:** country: United States; stateProvince: Alaska; locality: Anderson, Hwy 3; verbatimElevation: 235; verbatimLatitude: 64.295; verbatimLongitude: -149.084; **Identification:** identifiedBy: Jose Fernandez-Triana; **Event:** verbatimEventDate: 18-Jul-2009; **Record Level:** institutionID: Canadian National Collection of Insects, Arachnids and Nematodes
- **Type status:** Other material. **Occurrence:** catalogNumber: BIOUG00989-E07; recordedBy: Alex Smith; lifeStage: Adult; otherCatalogNumbers: ASGLF308-11; **Location:** country: Canada; stateProvince: Ontario; county: Haldimond-Dunn Townline; municipality: Windy Bluff; verbatimElevation: 179; verbatimLatitude: 42.861; verbatimLongitude: -79.703; **Identification:** identifiedBy: Jose Fernandez-Triana; **Event:** verbatimEventDate: 19-Sep-2010; **Record Level:** institutionID: Research Collection of M. Alex Smith
- **Type status:** Other material. **Occurrence:** catalogNumber: BIOUG03931-A10; recordedBy: H.Brown; lifeStage: Adult; otherCatalogNumbers: CNPPJ1263-12; **Location:** country: Canada; stateProvince: Ontario; county: Point Pelee NP; municipality: Cactus Field; verbatimElevation: 168; verbatimLatitude: 41.939; verbatimLongitude: -82.516; **Event:** verbatimEventDate: 07-Sep-2012; **Record Level:** institutionID: Biodiversity Institute of Ontario
- **Type status:** Other material. **Occurrence:** catalogNumber: CNCH0965; recordedBy: Goulet & Boudreault; lifeStage: Adult; otherCatalogNumbers: CNCHX980-09; **Location:** country: United States; stateProvince: Alaska; locality: Delta Junction; verbatimElevation: 315; verbatimLatitude: 64.044; verbatimLongitude: -145.524; **Identification:** identifiedBy: Jose Fernandez-Triana; **Event:** verbatimEventDate: 20-Jul-2009; **Record Level:** institutionID: Canadian National Collection of Insects, Arachnids and Nematodes
- **Type status:** Other material. **Occurrence:** catalogNumber: HYM00001322; recordedBy: Goulet, Boudreault & Fernandez; lifeStage: Adult; otherCatalogNumbers: ASWA608-08; **Location:** country: Canada; stateProvince: Saskatchewan; locality: Grassland National Park; verbatimElevation: 830; verbatimLatitude: 49.074; verbatimLongitude: -106.529; **Identification:** identifiedBy: Jose Fernandez-Triana; **Event:** verbatimEventDate: 04-Jun-2007; **Record Level:** institutionID: Canadian National Collection of Insects, Arachnids and Nematodes
- **Type status:** Other material. **Occurrence:** catalogNumber: CAM0437; recordedBy: Goulet & Fernandez; lifeStage: Adult; otherCatalogNumbers: CNCHZ627-09; **Location:** country: Canada; stateProvince: Ontario; county: Almonte; locality: 5 km NW of Almonte, Hwy 49, Burnt Land, Alvar Prov. Park; verbatimLatitude: 45.255; verbatimLongitude: -76.140; **Identification:** identifiedBy: Jose Fernandez-Triana; **Event:** verbatimEventDate: 29-May-2008; **Record Level:** institutionID: Canadian National Collection of Insects, Arachnids and Nematodes
- **Type status:** Other material. **Occurrence:** catalogNumber: HYM00000990; recordedBy: Goulet & Boudrealt; lifeStage: Adult; otherCatalogNumbers: DSWAS961-07; **Location:** country: Canada; stateProvince: Yukon Territory; locality: Pelly Crossing; verbatimElevation: 495; verbatimLatitude: 62.831; verbatimLongitude: -136.585; **Identification:** identifiedBy: Jose Fernandez-Triana; **Event:** verbatimEventDate: 15-Jul-2006; **Record Level:** institutionID: Canadian National Collection of Insects, Arachnids and Nematodes
- **Type status:** Other material. **Occurrence:** catalogNumber: CNCHYM 03139; recordedBy: F. I. S.; lifeStage: Adult; otherCatalogNumbers: HYCNE1824-11; **Location:** country: Canada; stateProvince: Ontario; locality: Macdiarmid; **Identification:** identifiedBy: J. Whitfield; **Event:** verbatimEventDate: 03-Aug-1962; **Record Level:** institutionID: Canadian National Collection of Insects, Arachnids and Nematodes
- **Type status:** Other material. **Occurrence:** catalogNumber: HYM00000975; recordedBy: Goulet & Boudrealt; lifeStage: Adult; otherCatalogNumbers: DSWAS946-07; **Location:** country: Canada; stateProvince: Yukon Territory; locality: Champagne; verbatimElevation: 733; verbatimLatitude: 60.790; verbatimLongitude: -136.437; **Identification:** identifiedBy: Jose Fernandez-Triana; **Event:** verbatimEventDate: 08-Jul-2006; **Record Level:** institutionID: Canadian National Collection of Insects, Arachnids and Nematodes
- **Type status:** Other material. **Occurrence:** catalogNumber: CNCH3206; recordedBy: Goulet & Bouderault; lifeStage: Adult; otherCatalogNumbers: CNCHV276-10; **Location:** country: United States; stateProvince: Alaska; locality: Anderson, Hwy 3; verbatimElevation: 235; verbatimLatitude: 64.295; verbatimLongitude: -149.084; **Identification:** identifiedBy: Jose Fernandez-Triana; **Event:** verbatimEventDate: 18-Jul-2009; **Record Level:** institutionID: Canadian National Collection of Insects, Arachnids and Nematodes
- **Type status:** Other material. **Occurrence:** catalogNumber: HYM00000935; recordedBy: Goulet & Boudrealt; lifeStage: Adult; otherCatalogNumbers: DSWAS906-07; **Location:** country: Canada; stateProvince: Yukon Territory; locality: Pelly Crossing; verbatimElevation: 495; verbatimLatitude: 62.831; verbatimLongitude: -136.585; **Identification:** identifiedBy: Jose Fernandez-Triana; **Event:** verbatimEventDate: 15-Jul-2006; **Record Level:** institutionID: Canadian National Collection of Insects, Arachnids and Nematodes
- **Type status:** Other material. **Occurrence:** catalogNumber: CNCH1008; recordedBy: Goulet & Boudreault; lifeStage: Adult; otherCatalogNumbers: CNCHX1023-09; **Location:** country: United States; stateProvince: Alaska; locality: Delta Junction; verbatimElevation: 315; verbatimLatitude: 64.044; verbatimLongitude: -145.524; **Identification:** identifiedBy: Jose Fernandez-Triana; **Event:** verbatimEventDate: 20-Jul-2009; **Record Level:** institutionID: Canadian National Collection of Insects, Arachnids and Nematodes
- **Type status:** Other material. **Occurrence:** catalogNumber: HYM00000987; recordedBy: Goulet & Boudrealt; lifeStage: Adult; otherCatalogNumbers: DSWAS958-07; **Location:** country: Canada; stateProvince: Yukon Territory; locality: Champagne; verbatimElevation: 733; verbatimLatitude: 60.790; verbatimLongitude: -136.437; **Identification:** identifiedBy: Jose Fernandez-Triana; **Event:** verbatimEventDate: 08-Jul-2006; **Record Level:** institutionID: Canadian National Collection of Insects, Arachnids and Nematodes
- **Type status:** Other material. **Occurrence:** catalogNumber: CNCH0987; recordedBy: Goulet & Boudreault; lifeStage: Adult; otherCatalogNumbers: CNCHX1002-09; **Location:** country: United States; stateProvince: Alaska; locality: Delta Junction; verbatimElevation: 315; verbatimLatitude: 64.044; verbatimLongitude: -145.524; **Identification:** identifiedBy: Jose Fernandez-Triana; **Event:** verbatimEventDate: 20-Jul-2009; **Record Level:** institutionID: Canadian National Collection of Insects, Arachnids and Nematodes
- **Type status:** Other material. **Occurrence:** catalogNumber: 08BBHYM-1368; recordedBy: J.Cossey, N.Jeffery, J.Straka; lifeStage: Adult; otherCatalogNumbers: BBHYM324-09; **Location:** country: Canada; stateProvince: Saskatchewan; county: Grasslands NP; municipality: East Block badlands; verbatimElevation: 848; verbatimLatitude: 49.071; verbatimLongitude: -106.531; **Identification:** identifiedBy: Jose Fernandez-Triana; **Event:** verbatimEventDate: 16-Jul-2008; **Record Level:** institutionID: Biodiversity Institute of Ontario
- **Type status:** Other material. **Occurrence:** catalogNumber: HYM00000968; recordedBy: Goulet & Boudrealt; lifeStage: Adult; otherCatalogNumbers: DSWAS939-07; **Location:** country: Canada; stateProvince: Yukon Territory; locality: Champagne; verbatimElevation: 733; verbatimLatitude: 60.790; verbatimLongitude: -136.437; **Identification:** identifiedBy: Jose Fernandez-Triana; **Event:** verbatimEventDate: 08-Jul-2006; **Record Level:** institutionID: Canadian National Collection of Insects, Arachnids and Nematodes
- **Type status:** Other material. **Occurrence:** catalogNumber: CNCHYM 03144; lifeStage: Adult; otherCatalogNumbers: HYCNE1829-11; **Location:** country: United States; stateProvince: California; locality: Los Angeles; **Identification:** identifiedBy: Jose Fernandez-Triana; **Event:** verbatimEventDate: 01-Dec-1944; **Record Level:** institutionID: Canadian National Collection of Insects, Arachnids and Nematodes
- **Type status:** Other material. **Occurrence:** catalogNumber: HYM00000936; recordedBy: Goulet & Boudrealt; lifeStage: Adult; otherCatalogNumbers: DSWAS907-07; **Location:** country: Canada; stateProvince: Yukon Territory; locality: Pelly Crossing; verbatimElevation: 495; verbatimLatitude: 62.831; verbatimLongitude: -136.585; **Identification:** identifiedBy: Jose Fernandez-Triana; **Event:** verbatimEventDate: 15-Jul-2006; **Record Level:** institutionID: Canadian National Collection of Insects, Arachnids and Nematodes
- **Type status:** Other material. **Occurrence:** catalogNumber: HYM00000951; recordedBy: Goulet & Boudrealt; lifeStage: Adult; otherCatalogNumbers: DSWAS922-07; **Location:** country: Canada; stateProvince: Yukon Territory; locality: Pelly Crossing; verbatimElevation: 495; verbatimLatitude: 62.831; verbatimLongitude: -136.585; **Identification:** identifiedBy: Jose Fernandez-Triana; **Event:** verbatimEventDate: 15-Jul-2006; **Record Level:** institutionID: Canadian National Collection of Insects, Arachnids and Nematodes
- **Type status:** Other material. **Occurrence:** catalogNumber: HYM00001320; recordedBy: Goulet, Boudreault & Fernandez; lifeStage: Adult; otherCatalogNumbers: ASWA606-08; **Location:** country: Canada; stateProvince: Saskatchewan; locality: Grassland National Park; verbatimElevation: 830; verbatimLatitude: 49.074; verbatimLongitude: -106.529; **Identification:** identifiedBy: Jose Fernandez-Triana; **Event:** verbatimEventDate: 04-Jun-2007; **Record Level:** institutionID: Canadian National Collection of Insects, Arachnids and Nematodes
- **Type status:** Other material. **Occurrence:** catalogNumber: HYM00000920; lifeStage: Adult; otherCatalogNumbers: DSWAS863-07; **Location:** country: Canada; stateProvince: Manitoba; county: Churchill; locality: 26 km SE Churchill, Twin Lakes Burn site; verbatimElevation: 38; verbatimLatitude: 58.617; verbatimLongitude: -93.812; **Identification:** identifiedBy: Jose Fernandez-Triana; **Event:** verbatimEventDate: 17-Aug-2006; **Record Level:** institutionID: Canadian National Collection of Insects, Arachnids and Nematodes
- **Type status:** Other material. **Occurrence:** catalogNumber: CNCHYM 03137; lifeStage: Adult; otherCatalogNumbers: HYCNE1822-11; **Location:** country: Canada; stateProvince: Ontario; locality: Hearst; **Identification:** identifiedBy: Jose Fernandez-Triana; **Event:** verbatimEventDate: 03-Aug-1971; **Record Level:** institutionID: Canadian National Collection of Insects, Arachnids and Nematodes
- **Type status:** Other material. **Occurrence:** catalogNumber: HYM00000820.2; recordedBy: J.G.Chillcot; lifeStage: Adult; otherCatalogNumbers: ASWAY563-08; **Location:** country: Canada; stateProvince: Manitoba; county: Churchill; locality: Mile 505, Hudson Bay Railway; **Identification:** identifiedBy: Jose Fernandez-Triana; **Event:** verbatimEventDate: 16-Jul-1952; **Record Level:** institutionID: Canadian National Collection of Insects, Arachnids and Nematodes
- **Type status:** Other material. **Occurrence:** catalogNumber: HYM00000976; recordedBy: Goulet & Boudrealt; lifeStage: Adult; otherCatalogNumbers: DSWAS947-07; **Location:** country: Canada; stateProvince: Yukon Territory; locality: Champagne; verbatimElevation: 733; verbatimLatitude: 60.790; verbatimLongitude: -136.437; **Identification:** identifiedBy: Jose Fernandez-Triana; **Event:** verbatimEventDate: 08-Jul-2006; **Record Level:** institutionID: Canadian National Collection of Insects, Arachnids and Nematodes
- **Type status:** Other material. **Occurrence:** catalogNumber: HYM00000943; recordedBy: Goulet & Boudrealt; lifeStage: Adult; otherCatalogNumbers: DSWAS914-07; **Location:** country: Canada; stateProvince: Yukon Territory; locality: Champagne; verbatimElevation: 733; verbatimLatitude: 60.790; verbatimLongitude: -136.437; **Identification:** identifiedBy: Jose Fernandez-Triana; **Event:** verbatimEventDate: 08-Jul-2006; **Record Level:** institutionID: Canadian National Collection of Insects, Arachnids and Nematodes
- **Type status:** Other material. **Occurrence:** catalogNumber: HYM00000934; recordedBy: Goulet & Boudrealt; lifeStage: Adult; otherCatalogNumbers: DSWAS905-07; **Location:** country: Canada; stateProvince: Yukon Territory; locality: Pelly Crossing; verbatimElevation: 495; verbatimLatitude: 62.831; verbatimLongitude: -136.585; **Identification:** identifiedBy: Jose Fernandez-Triana; **Event:** verbatimEventDate: 15-Jul-2006; **Record Level:** institutionID: Canadian National Collection of Insects, Arachnids and Nematodes
- **Type status:** Other material. **Occurrence:** catalogNumber: HYM00000938; recordedBy: Goulet & Boudrealt; lifeStage: Adult; otherCatalogNumbers: DSWAS909-07; **Location:** country: Canada; stateProvince: Yukon Territory; locality: Champagne; verbatimElevation: 733; verbatimLatitude: 60.790; verbatimLongitude: -136.437; **Identification:** identifiedBy: Jose Fernandez-Triana; **Event:** verbatimEventDate: 08-Jul-2006; **Record Level:** institutionID: Canadian National Collection of Insects, Arachnids and Nematodes
- **Type status:** Other material. **Occurrence:** catalogNumber: HYM00000950; recordedBy: Goulet & Boudrealt; lifeStage: Adult; otherCatalogNumbers: DSWAS921-07; **Location:** country: Canada; stateProvince: Yukon Territory; locality: Pelly Crossing; verbatimElevation: 495; verbatimLatitude: 62.831; verbatimLongitude: -136.585; **Identification:** identifiedBy: Jose Fernandez-Triana; **Event:** verbatimEventDate: 15-Jul-2006; **Record Level:** institutionID: Canadian National Collection of Insects, Arachnids and Nematodes
- **Type status:** Other material. **Occurrence:** catalogNumber: HYM00000956; recordedBy: Goulet & Boudrealt; lifeStage: Adult; otherCatalogNumbers: DSWAS927-07; **Location:** country: Canada; stateProvince: Yukon Territory; locality: Champagne; verbatimElevation: 733; verbatimLatitude: 60.790; verbatimLongitude: -136.437; **Identification:** identifiedBy: Jose Fernandez-Triana; **Event:** verbatimEventDate: 08-Jul-2006; **Record Level:** institutionID: Canadian National Collection of Insects, Arachnids and Nematodes
- **Type status:** Other material. **Occurrence:** catalogNumber: HYM00000973; recordedBy: Goulet & Boudrealt; lifeStage: Adult; otherCatalogNumbers: DSWAS944-07; **Location:** country: Canada; stateProvince: Yukon Territory; locality: Champagne; verbatimElevation: 733; verbatimLatitude: 60.790; verbatimLongitude: -136.437; **Identification:** identifiedBy: Jose Fernandez-Triana; **Event:** verbatimEventDate: 08-Jul-2006; **Record Level:** institutionID: Canadian National Collection of Insects, Arachnids and Nematodes
- **Type status:** Other material. **Occurrence:** catalogNumber: HYM00000970; recordedBy: Goulet & Boudrealt; lifeStage: Adult; otherCatalogNumbers: DSWAS941-07; **Location:** country: Canada; stateProvince: Yukon Territory; locality: Champagne; verbatimElevation: 733; verbatimLatitude: 60.790; verbatimLongitude: -136.437; **Identification:** identifiedBy: Jose Fernandez-Triana; **Event:** verbatimEventDate: 08-Jul-2006; **Record Level:** institutionID: Canadian National Collection of Insects, Arachnids and Nematodes
- **Type status:** Other material. **Occurrence:** catalogNumber: HYM00000940; recordedBy: Goulet & Boudrealt; lifeStage: Adult; otherCatalogNumbers: DSWAS911-07; **Location:** country: Canada; stateProvince: Yukon Territory; locality: Champagne; verbatimElevation: 733; verbatimLatitude: 60.790; verbatimLongitude: -136.437; **Identification:** identifiedBy: Jose Fernandez-Triana; **Event:** verbatimEventDate: 08-Jul-2006; **Record Level:** institutionID: Canadian National Collection of Insects, Arachnids and Nematodes
- **Type status:** Other material. **Occurrence:** catalogNumber: CNCHYM 03142; recordedBy: J. F. McAlpine; lifeStage: Adult; otherCatalogNumbers: HYCNE1827-11; **Location:** country: Canada; stateProvince: Newfoundland and Labrador; locality: Agric.Exp.Sta.St.John`s; **Identification:** identifiedBy: Jose Fernandez-Triana; **Event:** verbatimEventDate: 20-Jul-1967; **Record Level:** institutionID: Canadian National Collection of Insects, Arachnids and Nematodes
- **Type status:** Other material. **Occurrence:** catalogNumber: HYM00000942; recordedBy: Goulet & Boudrealt; lifeStage: Adult; otherCatalogNumbers: DSWAS913-07; **Location:** country: Canada; stateProvince: Yukon Territory; locality: Champagne; verbatimElevation: 733; verbatimLatitude: 60.790; verbatimLongitude: -136.437; **Identification:** identifiedBy: Jose Fernandez-Triana; **Event:** verbatimEventDate: 08-Jul-2006; **Record Level:** institutionID: Canadian National Collection of Insects, Arachnids and Nematodes
- **Type status:** Other material. **Occurrence:** catalogNumber: CNCHYM 03140; recordedBy: B. Poole; lifeStage: Adult; otherCatalogNumbers: HYCNE1825-11; **Location:** country: United States; stateProvince: Colorado; locality: Muddy Pass Jackson Co.; verbatimElevation: 2682.27; **Identification:** identifiedBy: Jose Fernandez-Triana; **Event:** verbatimEventDate: 15-Aug-1961; **Record Level:** institutionID: Canadian National Collection of Insects, Arachnids and Nematodes
- **Type status:** Other material. **Occurrence:** catalogNumber: 07PROBE-20072; recordedBy: A.Renaud; lifeStage: Adult; otherCatalogNumbers: ASWA270-08; **Location:** country: Canada; stateProvince: Manitoba; county: Churchill; municipality: 26 km SE Churchill, Twin Lakes; verbatimLatitude: 58.632; verbatimLongitude: -93.788; **Identification:** identifiedBy: Jose Fernandez-Triana; **Event:** verbatimEventDate: 07-Jul-2007; **Record Level:** institutionID: Biodiversity Institute of Ontario

##### Notes

This species is widely distributed within the Nearctic (e.g., Whitfield 2006, Fernandez-Triana 2010, Yu et al. 2012, Fernandez-Triana et al. 2014), and is also reported from Finland (Hellén 1977), Peru (de Huiza 1995), and was introduced to Hawai (Yu et al. 2012). Here we present additional information about the distribution of this species in Canada, including one new provincial record (AB), based on examined specimens from the BIO and CNC collections. Data for those specimens was extracted from BOLD.

#### Pholetesor viminetorum

(Wesmael, 1837)

##### Materials

- **Type status:** Other material. **Occurrence:** catalogNumber: CNCHYM 03217; recordedBy: S. M. Clark; lifeStage: Adult; otherCatalogNumbers: HYCNF002-11; **Location:** country: United States; stateProvince: Colorado; locality: Doolittle Ranch Mt. Evans; verbatimElevation: 2987.08; **Identification:** identifiedBy: J. Whitfield; **Event:** verbatimEventDate: 09-Aug-1961; **Record Level:** institutionID: Canadian National Collection of Insects, Arachnids and Nematodes
- **Type status:** Other material. **Occurrence:** catalogNumber: HYM00000806; recordedBy: J.G.Chillcot; lifeStage: Adult; otherCatalogNumbers: DSWAS749-07; **Location:** country: Canada; stateProvince: Manitoba; county: Churchill; locality: 23 km E Churchill, Fort Churchill; verbatimElevation: 27; verbatimLatitude: 58.734; verbatimLongitude: -93.789; **Identification:** identifiedBy: Jose Fernandez-Triana; **Event:** verbatimEventDate: 18-Jul-1952; **Record Level:** institutionID: Canadian National Collection of Insects, Arachnids and Nematodes
- **Type status:** Other material. **Occurrence:** catalogNumber: 07PROBE-22423; recordedBy: A.Renaud; lifeStage: Adult; otherCatalogNumbers: ASWAV964-08; **Location:** country: Canada; stateProvince: Manitoba; county: Churchill; locality: 23 km E Churchill, Ramsay Creek; verbatimElevation: 10; verbatimLatitude: 58.731; verbatimLongitude: -93.780; **Identification:** identifiedBy: Jose Fernandez-Triana; **Event:** verbatimEventDate: 16-Jul-2007; **Record Level:** institutionID: Biodiversity Institute of Ontario
- **Type status:** Other material. **Occurrence:** catalogNumber: BIOUG14184-G04; recordedBy: BIObus 2013; lifeStage: Adult; otherCatalogNumbers: SSGBB2792-14; **Location:** country: Canada; stateProvince: Newfoundland and Labrador; county: Gros Morne NP; municipality: Lomond River Trail; locality: Balsam fir, larch, fern and wildflowers (Ladyslipper orchid); verbatimElevation: 35; verbatimLatitude: 49.429; verbatimLongitude: -57.741; **Identification:** identifiedBy: Angela Telfer; **Event:** verbatimEventDate: 19-Jul-2013; **Record Level:** institutionID: Biodiversity Institute of Ontario
- **Type status:** Other material. **Occurrence:** catalogNumber: HYM00000809.2; recordedBy: J. G. Chillcot; lifeStage: Adult; otherCatalogNumbers: ASWAY600-08; **Location:** country: Canada; stateProvince: Manitoba; county: Churchill; locality: Fort Churchill; verbatimLatitude: 58.734; verbatimLongitude: -93.820; **Identification:** identifiedBy: Jose Fernandez-Triana; **Event:** verbatimEventDate: 11-Jul-1952; **Record Level:** institutionID: Canadian National Collection of Insects, Arachnids and Nematodes
- **Type status:** Other material. **Occurrence:** catalogNumber: 10PROBE-29276; recordedBy: Brandon Laforest; lifeStage: Adult; otherCatalogNumbers: JBHCH968-10; **Location:** country: Canada; stateProvince: Manitoba; county: Churchill; municipality: 12 km ESE Churchill, Launch Road; locality: Launch road Fen; verbatimLatitude: 58.754; verbatimLongitude: -93.998; **Identification:** identifiedBy: Jose Fernandez-Triana; **Event:** verbatimEventDate: 19-Jul-2010; **Record Level:** institutionID: Biodiversity Institute of Ontario
- **Type status:** Other material. **Occurrence:** catalogNumber: HYM00000798; recordedBy: J.G.Chillcot; lifeStage: Adult; otherCatalogNumbers: ASWAS997-07; **Location:** country: Canada; stateProvince: Manitoba; county: Churchill; locality: 23 km E Churchill, Fort Churchill; verbatimElevation: 27; verbatimLatitude: 58.734; verbatimLongitude: -93.789; **Identification:** identifiedBy: Jose Fernandez-Triana; **Event:** verbatimEventDate: 07-Aug-1952; **Record Level:** institutionID: Canadian National Collection of Insects, Arachnids and Nematodes
- **Type status:** Other material. **Occurrence:** catalogNumber: CAM0958; recordedBy: H. Goulet, A. Badiss, C. Boudeault; lifeStage: Adult; otherCatalogNumbers: CNCHX088-09; **Location:** country: Canada; stateProvince: Newfoundland and Labrador; county: Cap St-George; locality: Cap St-George, fallowfield & coastal tundra; verbatimLatitude: 48.466; verbatimLongitude: -59.260; **Identification:** identifiedBy: Jose Fernandez-Triana; **Event:** verbatimEventDate: 11-Jul-2008; **Record Level:** institutionID: Canadian National Collection of Insects, Arachnids and Nematodes
- **Type status:** Other material. **Occurrence:** catalogNumber: HYM00000981; recordedBy: Goulet & Boudrealt; lifeStage: Adult; otherCatalogNumbers: DSWAS952-07; **Location:** country: Canada; stateProvince: Yukon Territory; locality: Tahkini River road; verbatimElevation: 693; verbatimLatitude: 60.875; verbatimLongitude: -135.386; **Identification:** identifiedBy: Jose Fernandez-Triana; **Event:** verbatimEventDate: 06-Jul-2006; **Record Level:** institutionID: Canadian National Collection of Insects, Arachnids and Nematodes
- **Type status:** Other material. **Occurrence:** catalogNumber: BIOUG03191-A01; recordedBy: Stephanie Church; lifeStage: Adult; otherCatalogNumbers: CNEIB1042-12; **Location:** country: Canada; stateProvince: Alberta; county: Elk Island NP; municipality: penninsula in Astotin Lake; locality: The Point near administration/warden office; verbatimElevation: 719; verbatimLatitude: 53.685; verbatimLongitude: -112.860; **Identification:** identifiedBy: Angela Telfer; **Event:** verbatimEventDate: 10-Jun-2012; **Record Level:** institutionID: Biodiversity Institute of Ontario
- **Type status:** Other material. **Occurrence:** catalogNumber: HYM00000792.2; recordedBy: J. G. Chillcot; lifeStage: Adult; otherCatalogNumbers: ASWAY590-08; **Location:** country: Canada; stateProvince: Manitoba; county: Churchill; locality: Fort Churchill; verbatimLatitude: 58.734; verbatimLongitude: -93.820; **Identification:** identifiedBy: Jose Fernandez-Triana; **Event:** verbatimEventDate: 21-Jul-1952; **Record Level:** institutionID: Canadian National Collection of Insects, Arachnids and Nematodes
- **Type status:** Other material. **Occurrence:** catalogNumber: CAM0427; recordedBy: Goulet & Fernandez; lifeStage: Adult; otherCatalogNumbers: CNCHZ617-09; **Location:** country: Canada; stateProvince: Ontario; county: Almonte; locality: 5 km NW of Almonte, Hwy 49, Burnt Land, Alvar Prov. Park; verbatimLatitude: 45.255; verbatimLongitude: -76.140; **Identification:** identifiedBy: Jose Fernandez-Triana; **Event:** verbatimEventDate: 29-May-2008; **Record Level:** institutionID: Canadian National Collection of Insects, Arachnids and Nematodes
- **Type status:** Other material. **Occurrence:** catalogNumber: HYM00000795; recordedBy: J.G.Chillcot; lifeStage: Adult; otherCatalogNumbers: ASWAS994-07; **Location:** country: Canada; stateProvince: Manitoba; county: Churchill; locality: 23 km E Churchill, Fort Churchill; verbatimElevation: 27; verbatimLatitude: 58.734; verbatimLongitude: -93.789; **Identification:** identifiedBy: Jose Fernandez-Triana; **Event:** verbatimEventDate: 21-Jun-1952; **Record Level:** institutionID: Canadian National Collection of Insects, Arachnids and Nematodes
- **Type status:** Other material. **Occurrence:** catalogNumber: HYM00000813; recordedBy: J.G.Chillcot; lifeStage: Adult; otherCatalogNumbers: DSWAS756-07; **Location:** country: Canada; stateProvince: Manitoba; county: Churchill; locality: 23 km E Churchill, Fort Churchill; verbatimElevation: 27; verbatimLatitude: 58.734; verbatimLongitude: -93.789; **Identification:** identifiedBy: Jose Fernandez-Triana; **Event:** verbatimEventDate: 07-Nov-1952; **Record Level:** institutionID: Canadian National Collection of Insects, Arachnids and Nematodes
- **Type status:** Other material. **Occurrence:** catalogNumber: GOU 0582; recordedBy: J. Fernandez; lifeStage: Adult; otherCatalogNumbers: WOMIA352-11; **Location:** country: Canada; stateProvince: Nova Scotia; locality: Kejimkujik National Park, Peter`s Point; verbatimLatitude: 44.366; verbatimLongitude: -65.196; **Identification:** identifiedBy: Jose Fernandez-Triana; **Event:** verbatimEventDate: 02-Aug-2009; **Record Level:** institutionID: Canadian National Collection of Insects, Arachnids and Nematodes
- **Type status:** Other material. **Occurrence:** catalogNumber: 07PROBE-23399; recordedBy: A.Renaud; lifeStage: Adult; otherCatalogNumbers: ASWAX965-08; **Location:** country: Canada; stateProvince: Manitoba; county: Churchill; municipality: 23 km E Churchill, Ramsay Creek; verbatimElevation: 10; verbatimLatitude: 58.731; verbatimLongitude: -93.780; **Identification:** identifiedBy: Angela Telfer; **Event:** verbatimEventDate: 23-Jul-2007; **Record Level:** institutionID: Biodiversity Institute of Ontario
- **Type status:** Other material. **Occurrence:** catalogNumber: HYM00000922; recordedBy: J.G.Chillcot; lifeStage: Adult; otherCatalogNumbers: DSWAS865-07; **Location:** country: Canada; stateProvince: Manitoba; county: Churchill; locality: 23 km E Churchill, Fort Churchill; verbatimElevation: 27; verbatimLatitude: 58.734; verbatimLongitude: -93.789; **Identification:** identifiedBy: Jose Fernandez-Triana; **Event:** verbatimEventDate: 11-Jul-1952; **Record Level:** institutionID: Canadian National Collection of Insects, Arachnids and Nematodes
- **Type status:** Other material. **Occurrence:** catalogNumber: CAM0412; recordedBy: Goulet, Boudreault & Fernandez; lifeStage: Adult; otherCatalogNumbers: CNCHZ602-09; **Location:** country: Canada; stateProvince: Ontario; county: Manitoulin Island; locality: Little Current prairie Alvar; verbatimLatitude: 45.975; verbatimLongitude: -81.904; **Identification:** identifiedBy: Jose Fernandez-Triana; **Event:** verbatimEventDate: 04-Jun-2008; **Record Level:** institutionID: Canadian National Collection of Insects, Arachnids and Nematodes
- **Type status:** Other material. **Occurrence:** catalogNumber: 07PROBE-23413; recordedBy: A.Renaud; lifeStage: Adult; otherCatalogNumbers: ASWAX979-08; **Location:** country: Canada; stateProvince: Manitoba; county: Churchill; municipality: 23 km E Churchill, Ramsay Creek; verbatimElevation: 10; verbatimLatitude: 58.731; verbatimLongitude: -93.780; **Identification:** identifiedBy: Jose Fernandez-Triana; **Event:** verbatimEventDate: 23-Jul-2007; **Record Level:** institutionID: Biodiversity Institute of Ontario
- **Type status:** Other material. **Occurrence:** catalogNumber: 10BBCHY-2887; recordedBy: BIObus 2010; lifeStage: Adult; otherCatalogNumbers: BBHYK928-10; **Location:** country: Canada; stateProvince: British Columbia; county: Yoho NP; municipality: Kicking Horse Cmpgrd.; locality: Amphitheatre flowered meadow; verbatimElevation: 1317; verbatimLatitude: 51.424; verbatimLongitude: -116.429; **Identification:** identifiedBy: Julie K. Stahlhut; **Event:** verbatimEventDate: 21-Jul-2010; **Record Level:** institutionID: Biodiversity Institute of Ontario
- **Type status:** Other material. **Occurrence:** catalogNumber: CAM0422; recordedBy: Goulet & Fernandez; lifeStage: Adult; otherCatalogNumbers: CNCHZ612-09; **Location:** country: Canada; stateProvince: Ontario; county: Almonte; locality: 5 km NW of Almonte, Hwy 49, Burnt Land, Alvar Prov. Park; verbatimLatitude: 45.255; verbatimLongitude: -76.140; **Identification:** identifiedBy: Jose Fernandez-Triana; **Event:** verbatimEventDate: 29-May-2008; **Record Level:** institutionID: Canadian National Collection of Insects, Arachnids and Nematodes
- **Type status:** Other material. **Occurrence:** catalogNumber: HYM00000945; recordedBy: Goulet & Boudrealt; lifeStage: Adult; otherCatalogNumbers: DSWAS916-07; **Location:** country: Canada; stateProvince: Yukon Territory; locality: Pelly Crossing; verbatimElevation: 495; verbatimLatitude: 62.831; verbatimLongitude: -136.585; **Identification:** identifiedBy: Jose Fernandez-Triana; **Event:** verbatimEventDate: 15-Jul-2006; **Record Level:** institutionID: Canadian National Collection of Insects, Arachnids and Nematodes
- **Type status:** Other material. **Occurrence:** catalogNumber: HYM00000814.2; recordedBy: J.G.Chillcot; lifeStage: Adult; otherCatalogNumbers: ASWAY595-08; **Location:** country: Canada; stateProvince: Manitoba; county: Churchill; locality: 23 km E Churchill, Fort Churchill; verbatimElevation: 27; verbatimLatitude: 58.734; verbatimLongitude: -93.789; **Identification:** identifiedBy: Jose Fernandez-Triana; **Event:** verbatimEventDate: 21-Jul-1952; **Record Level:** institutionID: Canadian National Collection of Insects, Arachnids and Nematodes
- **Type status:** Other material. **Occurrence:** catalogNumber: CAM0435; recordedBy: Goulet & Fernandez; lifeStage: Adult; otherCatalogNumbers: CNCHZ625-09; **Location:** country: Canada; stateProvince: Ontario; county: Almonte; locality: 5 km NW of Almonte, Hwy 49, Burnt Land, Alvar Prov. Park; verbatimLatitude: 45.255; verbatimLongitude: -76.140; **Identification:** identifiedBy: Jose Fernandez-Triana; **Event:** verbatimEventDate: 29-May-2008; **Record Level:** institutionID: Canadian National Collection of Insects, Arachnids and Nematodes
- **Type status:** Other material. **Occurrence:** catalogNumber: HYM00000801; recordedBy: J.G.Chillcot; lifeStage: Adult; otherCatalogNumbers: DSWAS744-07; **Location:** country: Canada; stateProvince: Manitoba; county: Churchill; locality: 23 km E Churchill, Fort Churchill; verbatimElevation: 27; verbatimLatitude: 58.734; verbatimLongitude: -93.789; **Identification:** identifiedBy: Jose Fernandez-Triana; **Event:** verbatimEventDate: 07-Aug-1952; **Record Level:** institutionID: Canadian National Collection of Insects, Arachnids and Nematodes
- **Type status:** Other material. **Occurrence:** catalogNumber: CAM0959; recordedBy: H. Goulet, A. Badiss, C. Boudeault; lifeStage: Adult; otherCatalogNumbers: CNCHX089-09; **Location:** country: Canada; stateProvince: Newfoundland and Labrador; county: Cap St-George; locality: Cap St-George, fallowfield & coastal tundra; verbatimLatitude: 48.466; verbatimLongitude: -59.260; **Identification:** identifiedBy: Jose Fernandez-Triana; **Event:** verbatimEventDate: 11-Jul-2008; **Record Level:** institutionID: Canadian National Collection of Insects, Arachnids and Nematodes
- **Type status:** Other material. **Occurrence:** catalogNumber: HYM00000964; recordedBy: Goulet & Boudrealt; lifeStage: Adult; otherCatalogNumbers: DSWAS935-07; **Location:** country: Canada; stateProvince: Yukon Territory; locality: Tahkini River road; verbatimElevation: 693; verbatimLatitude: 60.875; verbatimLongitude: -135.386; **Identification:** identifiedBy: Jose Fernandez-Triana; **Event:** verbatimEventDate: 06-Jul-2006; **Record Level:** institutionID: Canadian National Collection of Insects, Arachnids and Nematodes
- **Type status:** Other material. **Occurrence:** catalogNumber: HYM00000794; recordedBy: J.G.Chillcot; lifeStage: Adult; otherCatalogNumbers: ASWAS993-07; **Location:** country: Canada; stateProvince: Manitoba; county: Churchill; locality: 23 km E Churchill, Fort Churchill; verbatimElevation: 27; verbatimLatitude: 58.734; verbatimLongitude: -93.789; **Identification:** identifiedBy: Jose Fernandez-Triana; **Event:** verbatimEventDate: 16-Jul-1952; **Record Level:** institutionID: Canadian National Collection of Insects, Arachnids and Nematodes
- **Type status:** Other material. **Occurrence:** catalogNumber: 07PROBE-22417; recordedBy: A.Renaud; lifeStage: Adult; otherCatalogNumbers: ASWAV958-08; **Location:** country: Canada; stateProvince: Manitoba; county: Churchill; municipality: 23 km E Churchill, Ramsay Creek; verbatimElevation: 10; verbatimLatitude: 58.731; verbatimLongitude: -93.780; **Identification:** identifiedBy: Angela Telfer; **Event:** verbatimEventDate: 16-Jul-2007; **Record Level:** institutionID: Biodiversity Institute of Ontario
- **Type status:** Other material. **Occurrence:** catalogNumber: GOU 0590; recordedBy: J. Fernandez; lifeStage: Adult; otherCatalogNumbers: WOMIA360-11; **Location:** country: Canada; stateProvince: Nova Scotia; locality: Kejimkujik National Park, Peter`s Point; verbatimLatitude: 44.366; verbatimLongitude: -65.196; **Identification:** identifiedBy: Jose Fernandez-Triana; **Event:** verbatimEventDate: 02-Aug-2009; **Record Level:** institutionID: Canadian National Collection of Insects, Arachnids and Nematodes
- **Type status:** Other material. **Occurrence:** catalogNumber: CAM0936; recordedBy: H. Goulet, A. Badiss, C. Boudeault; lifeStage: Adult; otherCatalogNumbers: CNCHX066-09; **Location:** country: Canada; stateProvince: Newfoundland and Labrador; county: Plum Point; locality: Plum Point, fallow field; verbatimLatitude: 51.064; verbatimLongitude: -56.982; **Identification:** identifiedBy: Jose Fernandez-Triana; **Event:** verbatimEventDate: 16-Jul-2008; **Record Level:** institutionID: Canadian National Collection of Insects, Arachnids and Nematodes
- **Type status:** Other material. **Occurrence:** catalogNumber: 07PROBE-20806; recordedBy: A.Renaud; lifeStage: Adult; otherCatalogNumbers: ASWAT346-08; **Location:** country: Canada; stateProvince: Manitoba; county: Churchill; municipality: 23 km E Churchill, Ramsay Creek; verbatimElevation: 10; verbatimLatitude: 58.731; verbatimLongitude: -93.780; **Identification:** identifiedBy: Jose Fernandez-Triana; **Event:** verbatimEventDate: 21-Jul-2007; **Record Level:** institutionID: Biodiversity Institute of Ontario
- **Type status:** Other material. **Occurrence:** catalogNumber: CAM0434; recordedBy: Goulet & Fernandez; lifeStage: Adult; otherCatalogNumbers: CNCHZ624-09; **Location:** country: Canada; stateProvince: Ontario; county: Almonte; locality: 5 km NW of Almonte, Hwy 49, Burnt Land, Alvar Prov. Park; verbatimLatitude: 45.255; verbatimLongitude: -76.140; **Identification:** identifiedBy: Jose Fernandez-Triana; **Event:** verbatimEventDate: 29-May-2008; **Record Level:** institutionID: Canadian National Collection of Insects, Arachnids and Nematodes
- **Type status:** Other material. **Occurrence:** catalogNumber: CAM0948; recordedBy: H. Goulet, A. Badiss, C. Boudeault; lifeStage: Adult; otherCatalogNumbers: CNCHX078-09; **Location:** country: Canada; stateProvince: Newfoundland and Labrador; county: Sally`s Cove; locality: Sally`s Cove, fallow field; verbatimLatitude: 49.739; verbatimLongitude: -57.925; **Identification:** identifiedBy: Jose Fernandez-Triana; **Event:** verbatimEventDate: 13-Jul-2008; **Record Level:** institutionID: Canadian National Collection of Insects, Arachnids and Nematodes
- **Type status:** Other material. **Occurrence:** catalogNumber: HYM00000802; recordedBy: J.G.Chillcot; lifeStage: Adult; otherCatalogNumbers: DSWAS745-07; **Location:** country: Canada; stateProvince: Manitoba; county: Churchill; locality: 23 km E Churchill, Fort Churchill; verbatimElevation: 27; verbatimLatitude: 58.734; verbatimLongitude: -93.789; **Identification:** identifiedBy: Jose Fernandez-Triana; **Event:** verbatimEventDate: 04-Aug-1952; **Record Level:** institutionID: Canadian National Collection of Insects, Arachnids and Nematodes
- **Type status:** Other material. **Occurrence:** catalogNumber: HYM00001682; recordedBy: Goulet & Boudreault; lifeStage: Adult; otherCatalogNumbers: ASWAU310-08; **Location:** country: Canada; stateProvince: Yukon Territory; locality: Fish Lake Rd; verbatimElevation: 1107; verbatimLatitude: 60.686; verbatimLongitude: -135.236; **Identification:** identifiedBy: Jose Fernandez-Triana; **Event:** verbatimEventDate: 05-Jul-2006; **Record Level:** institutionID: Canadian National Collection of Insects, Arachnids and Nematodes
- **Type status:** Other material. **Occurrence:** catalogNumber: CAM0421; recordedBy: Goulet & Fernandez; lifeStage: Adult; otherCatalogNumbers: CNCHZ611-09; **Location:** country: Canada; stateProvince: Ontario; county: Almonte; locality: 5 km NW of Almonte, Hwy 49, Burnt Land, Alvar Prov. Park; verbatimLatitude: 45.255; verbatimLongitude: -76.140; **Identification:** identifiedBy: Jose Fernandez-Triana; **Event:** verbatimEventDate: 29-May-2008; **Record Level:** institutionID: Canadian National Collection of Insects, Arachnids and Nematodes
- **Type status:** Other material. **Occurrence:** catalogNumber: CAM0416; recordedBy: Goulet & Fernandez; lifeStage: Adult; otherCatalogNumbers: CNCHZ606-09; **Location:** country: Canada; stateProvince: Ontario; county: Almonte; locality: 5 km NW of Almonte, Hwy 49, Burnt Land, Alvar Prov. Park; verbatimLatitude: 45.255; verbatimLongitude: -76.140; **Identification:** identifiedBy: Jose Fernandez-Triana; **Event:** verbatimEventDate: 29-May-2008; **Record Level:** institutionID: Canadian National Collection of Insects, Arachnids and Nematodes
- **Type status:** Other material. **Occurrence:** catalogNumber: HYM00000815; recordedBy: J.G.Chillcot; lifeStage: Adult; otherCatalogNumbers: DSWAS758-07; **Location:** country: Canada; stateProvince: Manitoba; county: Churchill; locality: 23 km E Churchill, Fort Churchill; verbatimElevation: 27; verbatimLatitude: 58.734; verbatimLongitude: -93.789; **Identification:** identifiedBy: Jose Fernandez-Triana; **Event:** verbatimEventDate: 01-Jan-1952; **Record Level:** institutionID: Canadian National Collection of Insects, Arachnids and Nematodes
- **Type status:** Other material. **Occurrence:** catalogNumber: CAM0428; recordedBy: Goulet & Fernandez; lifeStage: Adult; otherCatalogNumbers: CNCHZ618-09; **Location:** country: Canada; stateProvince: Ontario; county: Almonte; locality: 5 km NW of Almonte, Hwy 49, Burnt Land, Alvar Prov. Park; verbatimLatitude: 45.255; verbatimLongitude: -76.140; **Identification:** identifiedBy: Jose Fernandez-Triana; **Event:** verbatimEventDate: 29-May-2008; **Record Level:** institutionID: Canadian National Collection of Insects, Arachnids and Nematodes
- **Type status:** Other material. **Occurrence:** catalogNumber: HYM00000816; recordedBy: J.G.Chillcot; lifeStage: Adult; otherCatalogNumbers: DSWAS759-07; **Location:** country: Canada; stateProvince: Manitoba; county: Churchill; locality: Warkworth Creek nr. Churchill; verbatimLatitude: 58.700; verbatimLongitude: -94.100; **Identification:** identifiedBy: Jose Fernandez-Triana; **Event:** verbatimEventDate: 08-May-1952; **Record Level:** institutionID: Canadian National Collection of Insects, Arachnids and Nematodes
- **Type status:** Other material. **Occurrence:** catalogNumber: 07PROBE-20818; recordedBy: A.Renaud; lifeStage: Adult; otherCatalogNumbers: ASWAT358-08; **Location:** country: Canada; stateProvince: Manitoba; county: Churchill; municipality: 23 km E Churchill, Ramsay Creek; verbatimElevation: 10; verbatimLatitude: 58.731; verbatimLongitude: -93.780; **Identification:** identifiedBy: Jose Fernandez-Triana; **Event:** verbatimEventDate: 21-Jul-2007; **Record Level:** institutionID: Biodiversity Institute of Ontario
- **Type status:** Other material. **Occurrence:** catalogNumber: CAM0386; recordedBy: Goulet, Boudreault & Fernandez; lifeStage: Adult; otherCatalogNumbers: CNCHZ576-09; **Location:** country: Canada; stateProvince: Ontario; county: Manitoulin Island; locality: Little Current prairie Alvar; verbatimLatitude: 45.975; verbatimLongitude: -81.904; **Identification:** identifiedBy: Jose Fernandez-Triana; **Event:** verbatimEventDate: 04-Jun-2008; **Record Level:** institutionID: Canadian National Collection of Insects, Arachnids and Nematodes
- **Type status:** Other material. **Occurrence:** catalogNumber: CAM0387; recordedBy: Goulet, Boudreault & Fernandez; lifeStage: Adult; otherCatalogNumbers: CNCHZ577-09; **Location:** country: Canada; stateProvince: Ontario; county: Manitoulin Island; locality: Little Current prairie Alvar; verbatimLatitude: 45.975; verbatimLongitude: -81.904; **Identification:** identifiedBy: Jose Fernandez-Triana; **Event:** verbatimEventDate: 04-Jun-2008; **Record Level:** institutionID: Canadian National Collection of Insects, Arachnids and Nematodes
- **Type status:** Other material. **Occurrence:** catalogNumber: CAM0944; recordedBy: H. Goulet, A. Badiss, C. Boudeault; lifeStage: Adult; otherCatalogNumbers: CNCHX074-09; **Location:** country: Canada; stateProvince: Newfoundland and Labrador; county: Sally`s Cove; locality: Sally`s Cove, fallow field; verbatimLatitude: 49.739; verbatimLongitude: -57.925; **Identification:** identifiedBy: Jose Fernandez-Triana; **Event:** verbatimEventDate: 13-Jul-2008; **Record Level:** institutionID: Canadian National Collection of Insects, Arachnids and Nematodes
- **Type status:** Other material. **Occurrence:** catalogNumber: CNCHYM 03216; recordedBy: B. H. Poole; lifeStage: Adult; otherCatalogNumbers: HYCNF001-11; **Location:** country: United States; stateProvince: Colorado; locality: Boulder; verbatimElevation: 1828.82; **Identification:** identifiedBy: J. Whitfield; **Event:** verbatimEventDate: 31-May-1961; **Record Level:** institutionID: Canadian National Collection of Insects, Arachnids and Nematodes
- **Type status:** Other material. **Occurrence:** catalogNumber: CAM0952; recordedBy: H. Goulet, A. Badiss, C. Boudeault; lifeStage: Adult; otherCatalogNumbers: CNCHX082-09; **Location:** country: Canada; stateProvince: Newfoundland and Labrador; county: South Branch; locality: South Branch, fallow field; verbatimLatitude: 47.922; verbatimLongitude: -59.014; **Identification:** identifiedBy: Jose Fernandez-Triana; **Event:** verbatimEventDate: 10-Jul-2008; **Record Level:** institutionID: Canadian National Collection of Insects, Arachnids and Nematodes
- **Type status:** Other material. **Occurrence:** catalogNumber: HYM00000815.2; recordedBy: J.G.Chillcot; lifeStage: Adult; otherCatalogNumbers: ASWAY594-08; **Location:** country: Canada; stateProvince: Manitoba; county: Churchill; locality: 23 km E Churchill, Fort Churchill; verbatimElevation: 27; verbatimLatitude: 58.734; verbatimLongitude: -93.789; **Identification:** identifiedBy: Jose Fernandez-Triana; **Event:** verbatimEventDate: 01-Jul-1952; **Record Level:** institutionID: Canadian National Collection of Insects, Arachnids and Nematodes
- **Type status:** Other material. **Occurrence:** catalogNumber: CAM0426; recordedBy: Goulet & Fernandez; lifeStage: Adult; otherCatalogNumbers: CNCHZ616-09; **Location:** country: Canada; stateProvince: Ontario; county: Almonte; locality: 5 km NW of Almonte, Hwy 49, Burnt Land, Alvar Prov. Park; verbatimLatitude: 45.255; verbatimLongitude: -76.140; **Identification:** identifiedBy: Jose Fernandez-Triana; **Event:** verbatimEventDate: 29-May-2008; **Record Level:** institutionID: Canadian National Collection of Insects, Arachnids and Nematodes
- **Type status:** Other material. **Occurrence:** catalogNumber: 10PROBE-28643; recordedBy: Brandon Laforest; lifeStage: Adult; otherCatalogNumbers: JBHCH335-10; **Location:** country: Canada; stateProvince: Manitoba; county: Churchill; municipality: 11 km S Churchill, Goose Creek; locality: Goose Creek Second Train Tracks; verbatimLatitude: 58.718; verbatimLongitude: -94.122; **Identification:** identifiedBy: Jose Fernandez-Triana; **Event:** verbatimEventDate: 16-Jul-2010; **Record Level:** institutionID: Biodiversity Institute of Ontario
- **Type status:** Other material. **Occurrence:** catalogNumber: HYM00000811.2; recordedBy: J.G.Chillcot; lifeStage: Adult; otherCatalogNumbers: ASWAY598-08; **Location:** country: Canada; stateProvince: Manitoba; county: Churchill; locality: 23 km E Churchill, Fort Churchill; verbatimElevation: 27; verbatimLatitude: 58.734; verbatimLongitude: -93.789; **Identification:** identifiedBy: Jose Fernandez-Triana; **Event:** verbatimEventDate: 11-Jul-1952; **Record Level:** institutionID: Canadian National Collection of Insects, Arachnids and Nematodes
- **Type status:** Other material. **Occurrence:** catalogNumber: CAM0492; recordedBy: H. Goulet, A. Badiss, C. Boudeault; lifeStage: Adult; otherCatalogNumbers: CNCHZ1139-09; **Location:** country: Canada; stateProvince: Newfoundland and Labrador; locality: Sally`s Cove, fallow field; verbatimLatitude: 49.739; verbatimLongitude: -57.925; **Identification:** identifiedBy: Jose Fernandez-Triana; **Event:** verbatimEventDate: 13-Jul-2008; **Record Level:** institutionID: Canadian National Collection of Insects, Arachnids and Nematodes
- **Type status:** Other material. **Occurrence:** catalogNumber: CAM0945; recordedBy: H. Goulet, A. Badiss, C. Boudeault; lifeStage: Adult; otherCatalogNumbers: CNCHX075-09; **Location:** country: Canada; stateProvince: Newfoundland and Labrador; county: Sally`s Cove; locality: Sally`s Cove, fallow field; verbatimLatitude: 49.739; verbatimLongitude: -57.925; **Identification:** identifiedBy: Jose Fernandez-Triana; **Event:** verbatimEventDate: 13-Jul-2008; **Record Level:** institutionID: Canadian National Collection of Insects, Arachnids and Nematodes
- **Type status:** Other material. **Occurrence:** catalogNumber: HYM00000603; recordedBy: Goulet & Boudrealt; lifeStage: Adult; otherCatalogNumbers: CNCHY331-07; **Location:** country: Canada; stateProvince: Yukon Territory; locality: Fish Lake road; verbatimElevation: 1107; verbatimLatitude: 60.688; verbatimLongitude: -135.238; **Identification:** identifiedBy: Jose Fernandez-Triana; **Event:** verbatimEventDate: 05-Jul-2006; **Record Level:** institutionID: Canadian National Collection of Insects, Arachnids and Nematodes
- **Type status:** Other material. **Occurrence:** catalogNumber: CAM0951; recordedBy: H. Goulet, A. Badiss, C. Boudeault; lifeStage: Adult; otherCatalogNumbers: CNCHX081-09; **Location:** country: Canada; stateProvince: Newfoundland and Labrador; county: Plum Point; locality: Plum Point, fallow field; verbatimLatitude: 51.064; verbatimLongitude: -56.982; **Identification:** identifiedBy: Jose Fernandez-Triana; **Event:** verbatimEventDate: 16-Jul-2008; **Record Level:** institutionID: Canadian National Collection of Insects, Arachnids and Nematodes
- **Type status:** Other material. **Occurrence:** catalogNumber: HYM00000989; recordedBy: Goulet & Boudrealt; lifeStage: Adult; otherCatalogNumbers: DSWAS960-07; **Location:** country: Canada; stateProvince: Yukon Territory; locality: Fish Lake road; verbatimElevation: 1107; verbatimLatitude: 60.688; verbatimLongitude: -135.238; **Identification:** identifiedBy: Jose Fernandez-Triana; **Event:** verbatimEventDate: 05-Jul-2006; **Record Level:** institutionID: Canadian National Collection of Insects, Arachnids and Nematodes
- **Type status:** Other material. **Occurrence:** catalogNumber: MIC 000900; recordedBy: H. Goulet, A. Badiss, C. Boudeault; lifeStage: Adult; otherCatalogNumbers: CNCHZ1092-09; **Location:** country: Canada; stateProvince: Newfoundland and Labrador; locality: Sally`s Cove, fallow field; verbatimLatitude: 49.739; verbatimLongitude: -57.925; **Identification:** identifiedBy: Jose Fernandez-Triana; **Event:** verbatimEventDate: 13-Jul-2008; **Record Level:** institutionID: Canadian National Collection of Insects, Arachnids and Nematodes
- **Type status:** Other material. **Occurrence:** catalogNumber: 07PROBE-20808; recordedBy: A.Renaud; lifeStage: Adult; otherCatalogNumbers: ASWAT348-08; **Location:** country: Canada; stateProvince: Manitoba; county: Churchill; municipality: 23 km E Churchill, Ramsay Creek; verbatimElevation: 10; verbatimLatitude: 58.731; verbatimLongitude: -93.780; **Identification:** identifiedBy: Jose Fernandez-Triana; **Event:** verbatimEventDate: 21-Jul-2007; **Record Level:** institutionID: Biodiversity Institute of Ontario
- **Type status:** Other material. **Occurrence:** catalogNumber: CAM0365; recordedBy: Goulet & Fernandez; lifeStage: Adult; otherCatalogNumbers: CNCHZ555-09; **Location:** country: Canada; stateProvince: Ontario; county: Almonte; locality: 5 km NW of Almonte, Hwy 49, Burnt Land, Alvar Prov. Park; verbatimLatitude: 45.255; verbatimLongitude: -76.140; **Identification:** identifiedBy: Jose Fernandez-Triana; **Event:** verbatimEventDate: 29-May-2008; **Record Level:** institutionID: Canadian National Collection of Insects, Arachnids and Nematodes
- **Type status:** Other material. **Occurrence:** catalogNumber: CAM0384; recordedBy: Goulet, Boudreault & Fernandez; lifeStage: Adult; otherCatalogNumbers: CNCHZ574-09; **Location:** country: Canada; stateProvince: Ontario; county: Manitoulin Island; locality: Little Current, prairie alvar; verbatimLatitude: 45.975; verbatimLongitude: -81.904; **Identification:** identifiedBy: Jose Fernandez-Triana; **Event:** verbatimEventDate: 04-Jun-2008; **Record Level:** institutionID: Canadian National Collection of Insects, Arachnids and Nematodes
- **Type status:** Other material. **Occurrence:** catalogNumber: CAM0431; recordedBy: Goulet & Fernandez; lifeStage: Adult; otherCatalogNumbers: CNCHZ621-09; **Location:** country: Canada; stateProvince: Ontario; county: Almonte; locality: 5 km NW of Almonte, Hwy 49, Burnt Land, Alvar Prov. Park; verbatimLatitude: 45.255; verbatimLongitude: -76.140; **Identification:** identifiedBy: Jose Fernandez-Triana; **Event:** verbatimEventDate: 29-May-2008; **Record Level:** institutionID: Canadian National Collection of Insects, Arachnids and Nematodes

##### Notes

This species is widely distributed in the Holarctic (e.g., Whitfield 2006, Fernandez-Triana 2010, Yu et al. 2012, Fernandez-Triana et al. 2014). Here we present new and detailed information about the distribution of the species in Canada, including one new provincial record (ON), based on examined specimens from the BIO and CNC collections. Data for those specimens was extracted from BOLD.

## Discussion

In addition to being a diverse platform for the generation, analysis and permanent storage of DNA barcodes, the Barcode of Life Data Systems (BOLD) houses an immense amount of collateral data that are only starting to be explored and exploited. Validated species occurrences records are one such data element, critical for various fields and applications from environmental niche modeling in ecology to risk assessments in conservation biology. This paper demonstrates the potential of using BOLD occurrence data for these purposes – to define and refine the distributions of species – and presents a model for their rapid dissemination in the primary literature.

The key to this model is the seamless and dynamic transition of data between source and journal. By converting specimen information (such as locality, collection date, collector, voucher depository) from the BOLD platform to the Excel template provided by the Pensoft Writing Tool (PWT), the authoring platform of the Biodiversity Data Journal (BDJ), it is possible to quickly upload and generate long lists of "Material Examined" for papers discussing taxonomy, ecology and/or new distribution records of species. Additional functions in BOLD allow verification of this material, for instance the ability to generate high-res image libraries (e.g., Suppl. material 3) or Kimura 2-Parameter (K2P) phylograms using the corresponding sequences (e.g., Suppl. material 4). For the vast majority of publications including DNA barcodes, the generation and publication of ancillary data associated with the barcoded material is seldom highlighted and often disregarded. The analysis of those data sets to uncover new distribution patterns of species has rarely been explored, even though many BOLD records represent new and/or significant discoveries (but see Smith et al. (2012) for a discussion of this topic). The introduction of journals specializing in – and streamlining – the release of these datasets, such as the BDJ, should facilitate thorough analysis of these records, even large datasets. This paper used a moderately large data set (630 specimens comprising 10 species) and revealed several new distribution records, as well as the workflow that permitted their release (i.e., from a database to a publication).

The logical next step would be to create a simple interface and module at the source database (BOLD) that will submit data selected by the author, "at the click of a button", into the PWT, using the Application Programming Interface (API) of the latter. Within the PWT environment, the submitting author can complete the manuscript by adding additional text, figures, images, citations, references and so on. The author can also invite co-authors and/or "contributors" (e.g., linguistic editors, mentors, colleagues who are not formal co-authors) to collaboratively work on the manuscript. After completion, the manuscript is submitted to BDJ, again "at the click of a button", where it undergoes a community peer-review process and subsequent publication upon acceptance (e.g., Smith et al. 2013). The published article will contain dynamic links to external sources (Penev et al. 2010), including to BOLD, and will also be available in highly structured XML format which ensures machine readability of the text and data. For example, the occurrence records will automatically be exported to GBIF and appear there with their original source (e.g., BOLD) and publication source (reference for citation), bringing additional visibility and usage tracking statistics to both the source database and the authors. Visibility and usability are essential here, particularly for collateral data such as species occurrence records residing on BOLD, as the consumers and eventual uses of this biodiversity data are nearly as diverse as the organisms they represent.

## Supplementary Material

Supplementary material 1 Supplemental Appendix 1 Data type: Complete Specimen Data downloaded from BOLD
File: oo_32170.xls Fernandez-Triana et al. 2014

Supplementary material 2 Supplemental Appendix 2 Data type: Data from BOLD modified for BDJ template
File: oo_32171.xls Fernandez-Triana et al. 2014

Supplementary material 3 Supplemental Appendix 3 Data type: Image Library of specimens studied
File: oo_32328.pdf Fernández-Triana et al. 2014

Supplementary material 4 Supplemental Appendix 4 Data type: K2P tree of sequences studied
File: oo_32327.pdf Fernández-Triana et al. 2014

Supplementary material 5 Distribution of 10 Microgastrinae species based on BOLD records Data type: Distribution records
File: oo_32527.pdf Fernández-Triana et al. 2014

XML Treatment for Apanteles carpatus

XML Treatment for Apanteles conanchetorum

XML Treatment for Apanteles ensiger

XML Treatment for Apanteles sodalis

XML Treatment for Apanteles xanthostigma

XML Treatment for Cotesia crambi

XML Treatment for Dolichogenidea longicauda

XML Treatment for Hygroplitis melligaster

XML Treatment for Pholetesor bedelliae

XML Treatment for Pholetesor viminetorum
