# Supplementary material for: Streamlining the use of BOLD specimen data to record species distributions: a case study with ten Nearctic species of Microgastrinae (Hymenoptera: Braconidae)
Source: Biodivers Data J. 2014 Oct 29;(2):e4153. doi: 10.3897/BDJ.2.e4153 (PMC4251541; doi:10.3897/BDJ.2.e4153)
Supplement: Supplementary material 3 — Supplemental Appendix 3 [file biodiversity_data_journal-2-e4153-s003.pdf]

Figure 2. BOLD Image library of 134 specimens from 10 species

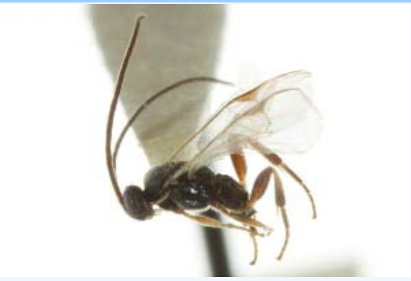

CNCHYM 00081 [Lateral]
Apanteles carpatus

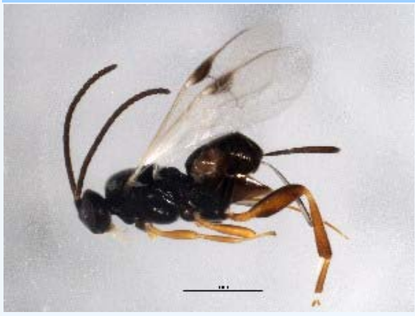

BIOUG01631-B09 [Lateral]
Apanteles carpatus
BIN URI: BOLD:AAC2372

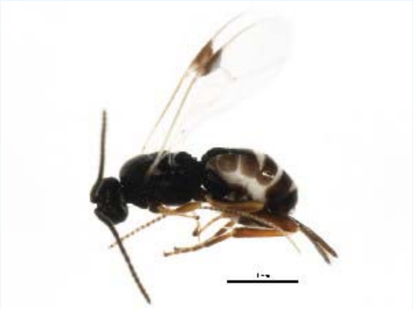

BIOUG01088-A03 [Lateral]
Apanteles carpatus
BIN URI: BOLD:AAC2372

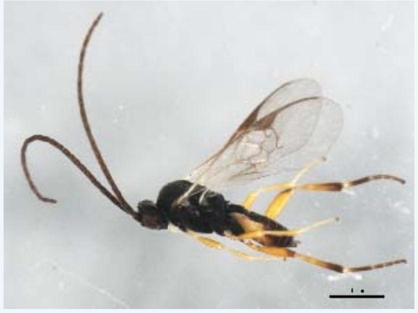

BIOUG00989-G12 [Lateral]
Apanteles conanchetorum
BIN URI: BOLD:AAC5506

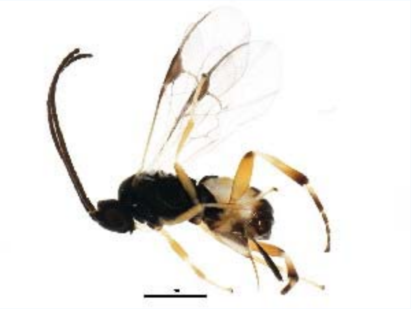

BIOUG01327-H05 [Lateral]
Apanteles conanchetorum
BIN URI: BOLD:AAC5506

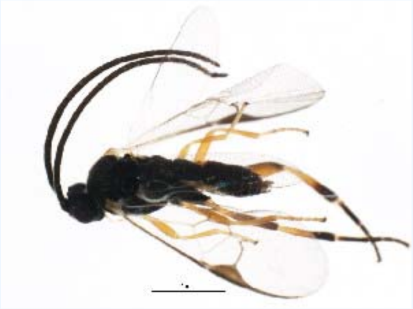

BIOUG01252-E11 [Lateral]
Apanteles conanchetorum
BIN URI: BOLD:AAC5506

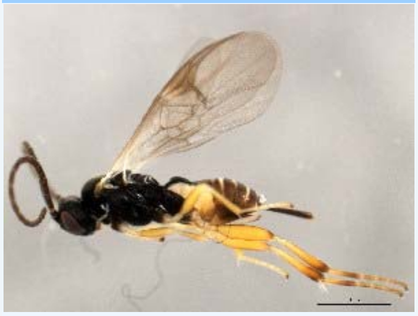

10BBCHY-2243 [Lateral]
Apanteles conanchetorum
BIN URI: BOLD:AAC5507

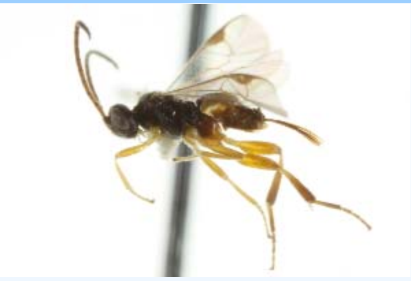

CNCHYM 00089 [Lateral]
Apanteles conanchetorum

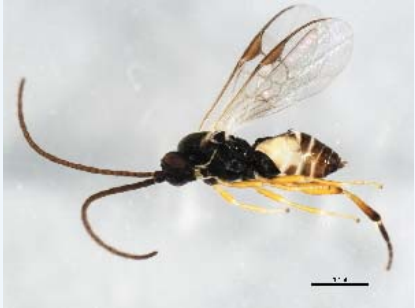

BIOUG00989-E10 [Lateral]
Apanteles conanchetorum
BIN URI: BOLD:AAC5506

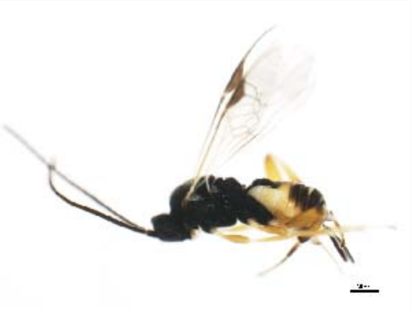

BIOUG01280-G04 [Lateral]
Apanteles conanchetorum
BIN URI: BOLD:AAC5506

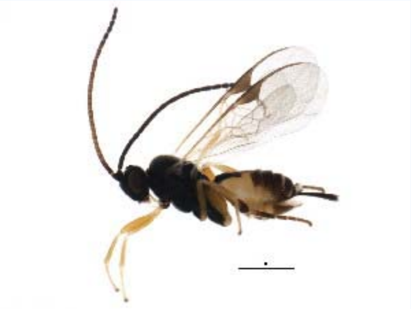

BIOUG00914-E08 [Lateral]
Apanteles conanchetorum
BIN URI: BOLD:AAC5506

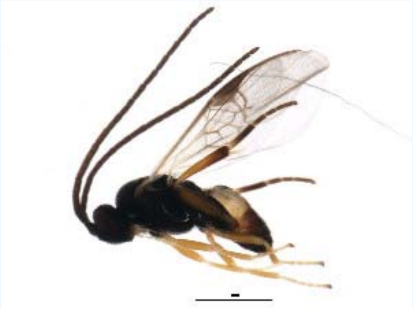

BIOUG00914-F06 [Lateral]
Apanteles conanchetorum
BIN URI: BOLD:AAC5506

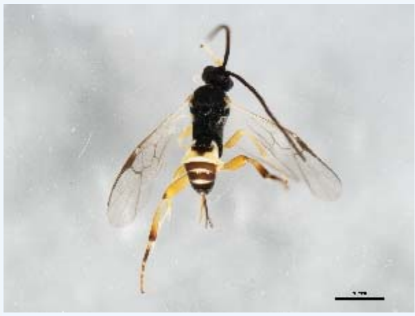

BIOUG00989-E12 [Dorsal]
Apanteles conanchetorum
BIN URI: BOLD:AAC5506

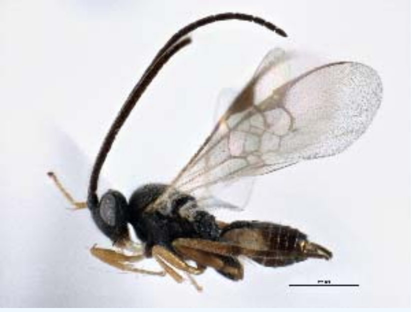

BIOUG04245-B04 [Lateral]
Apanteles conanchetorum
BIN URI: BOLD:AAC5507

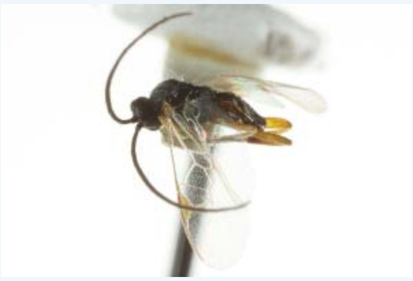

CNCHYM 00093 [Lateral]
Apanteles conanchetorum

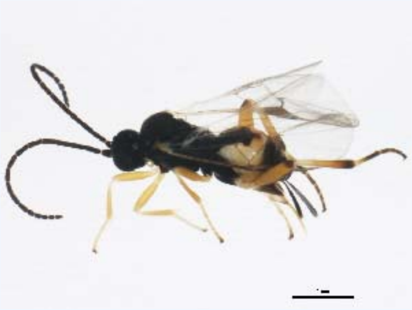

BIOUG01330-D09 [Lateral]
Apanteles conanchetorum
BIN URI: BOLD:AAC5506

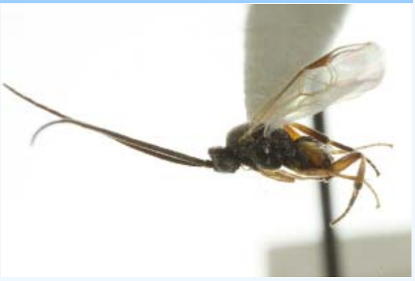

CNCHYM 03970 [Lateral]
Apanteles conanchetorum

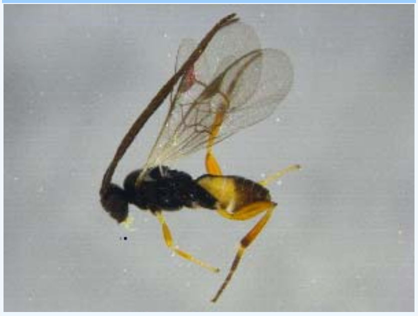

BIOUG03486-E11 [Lateral]
Apanteles conanchetorum
BIN URI: BOLD:AAC5507

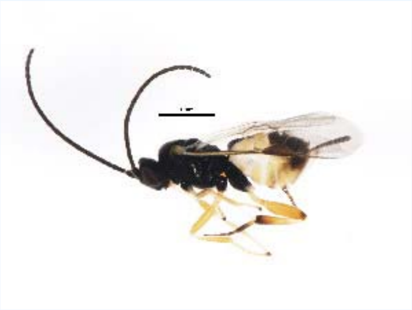

BIOUG00861-G06 [Lateral]
Apanteles conanchetorum
BIN URI: BOLD:AAC5506

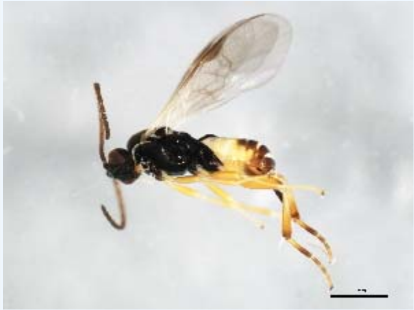

BIOUG00989-E11 [Lateral]
Apanteles conanchetorum
BIN URI: BOLD:AAC5506

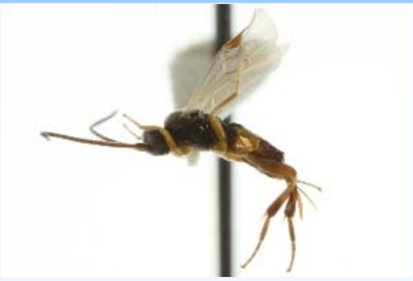

CNCHYM 00090 [Lateral]
Apanteles conanchetorum

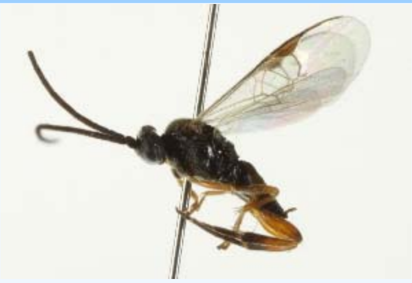

PCPP10-0759 [Lateral]
Apanteles conanchetorum
BIN URI: BOLD:AAC5506

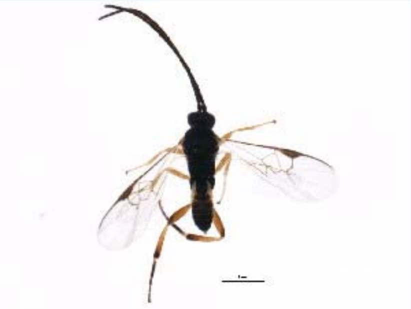

BIOUG00914-F08 [Dorsal]
Apanteles conanchetorum
BIN URI: BOLD:AAC5506

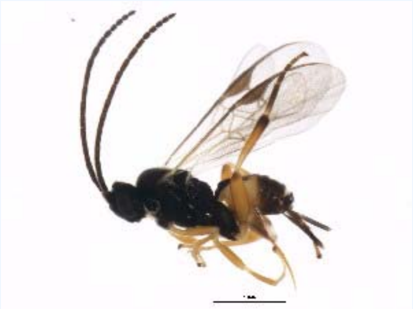

BIOUG00991-B06 [Lateral]
Apanteles conanchetorum
BIN URI: BOLD:AAC5506

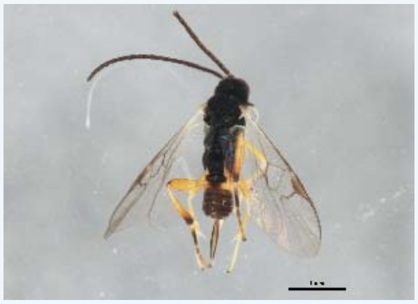
BIOUG00989-A08 [Dorsal]
Apanteles conanchetorum
BIN URI: BOLD:AAC5506

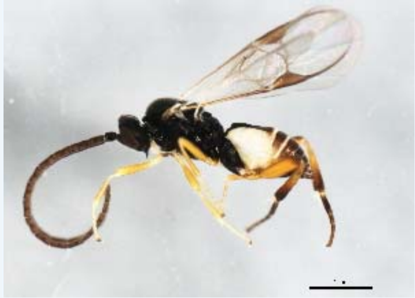
BIOUG00989-E09 [Lateral]
Apanteles conanchetorum
BIN URI: BOLD:AAC5506

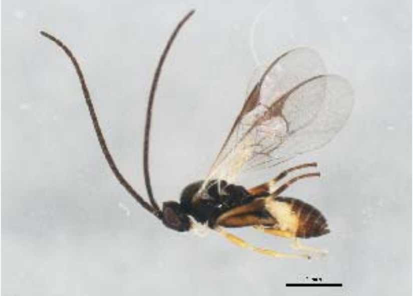
BIOUG00989-A07 [Lateral]
Apanteles conanchetorum
BIN URI: BOLD:AAC5506

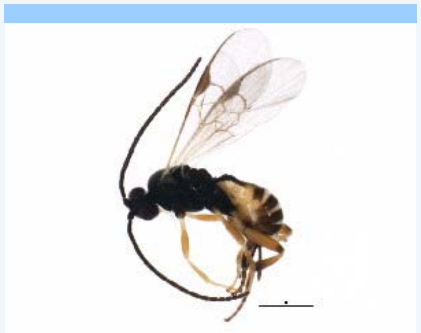
BIOUG00914-E04 [Lateral]
Apanteles conanchetorum
BIN URI: BOLD:AAC5506

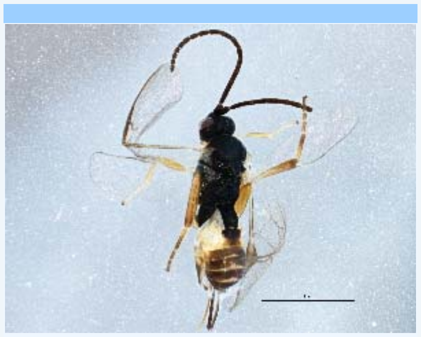
BIOUG04245-A01 [Lateral]
Apanteles conanchetorum
BIN URI: BOLD:AAC5507

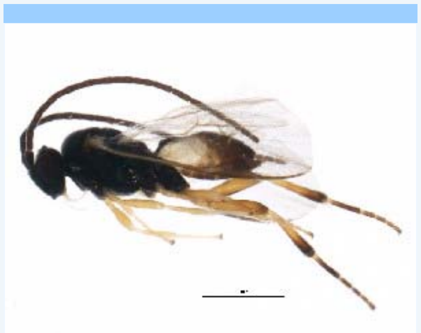
BIOUG00914-G01 [Lateral]
Apanteles conanchetorum
BIN URI: BOLD:AAC5506

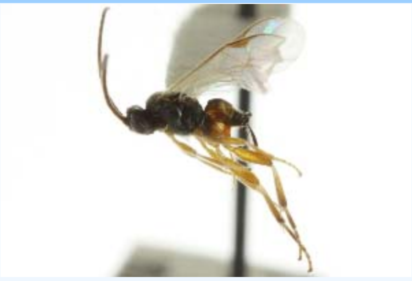
CNCHYM 00088 [Lateral]
Apanteles conanchetorum

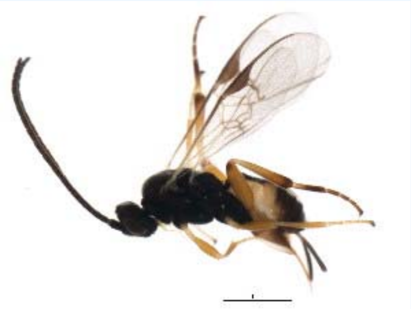
BIOUG00914-E03 [Lateral]
Apanteles conanchetorum
BIN URI: BOLD:AAC5506

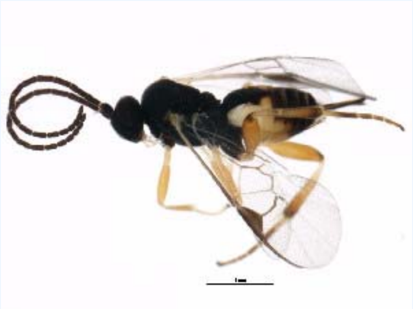
BIOUG00991-A05 [Lateral]
Apanteles conanchetorum
BIN URI: BOLD:AAC5506

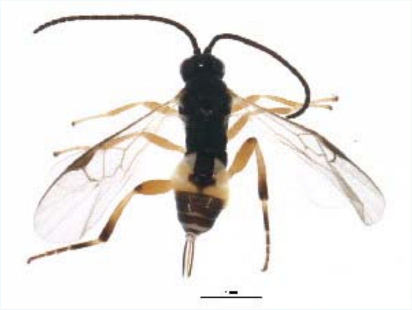
BIOUG00914-E07 [Dorsal]
Apanteles conanchetorum
BIN URI: BOLD:AAC5506

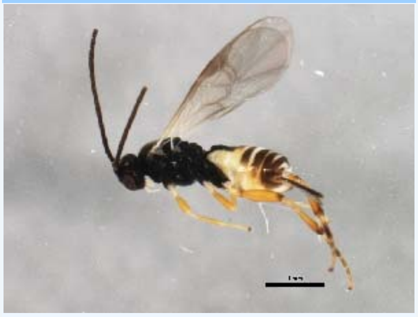
BIOUG00988-A09 [Lateral]
Apanteles conanchetorum
BIN URI: BOLD:AAC5506

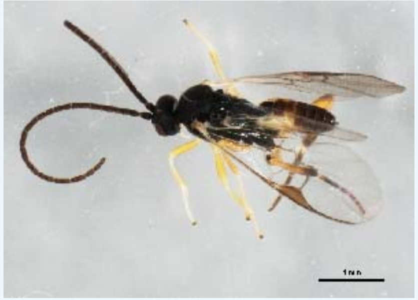
BIOUG00989-F01 [Lateral]
Apanteles conanchetorum
BIN URI: BOLD:AAC5506

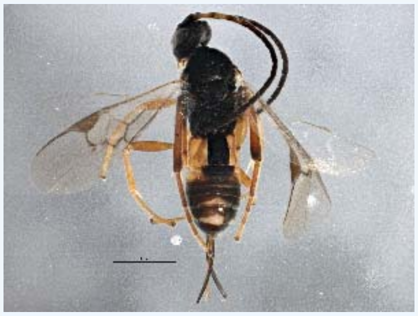

BIOUG03220-F05 [Dorsal]
Apanteles ensiger
BIN URI: BOLD:ACE6783

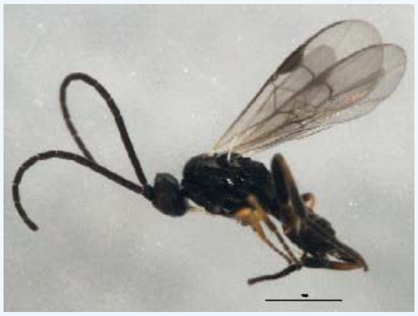

10BBCHY-1304 [Lateral]
Apanteles ensiger
BIN URI: BOLD:AAA3764

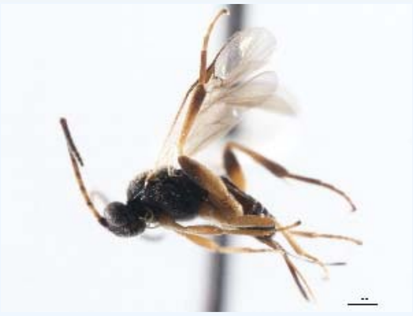

CNCHYM 00107 [Lateral]
Apanteles ensiger

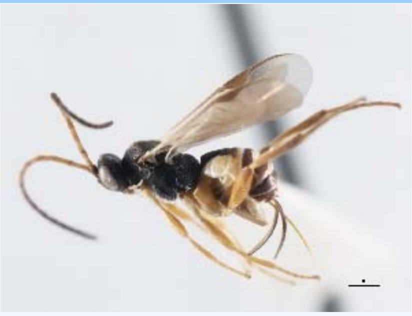

CNCHYM 00105 [Lateral]
Apanteles ensiger

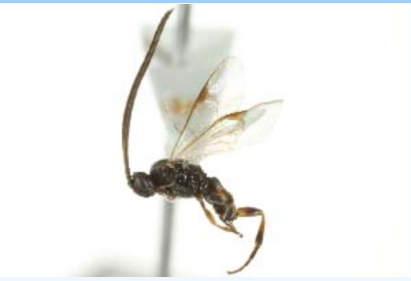

HYM00000665 [Lateral]
Apanteles ensiger

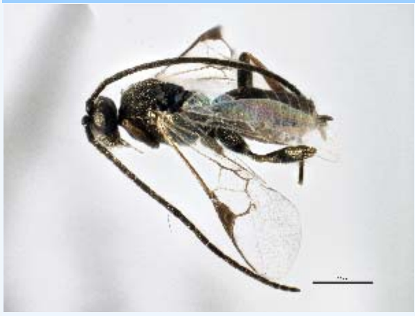

BIOUG08647-G07 [Lateral]
Apanteles ensiger
BIN URI: BOLD:ACE6783

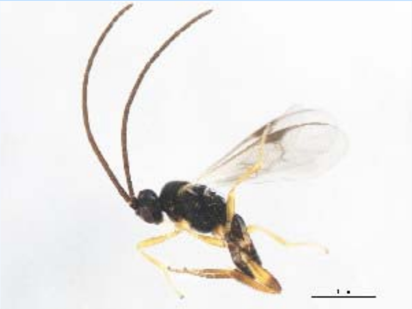

10BBHYM-1225 [Lateral]
Apanteles ensiger
BIN URI: BOLD:AAA3764

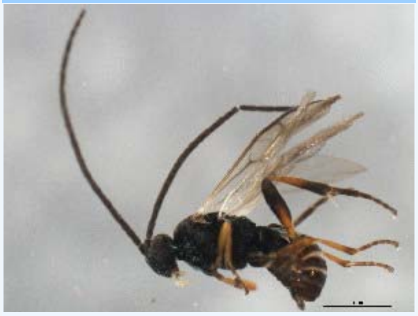

BIOUG00726-B07 [Lateral]
Apanteles ensiger
BIN URI: BOLD:AAA3764

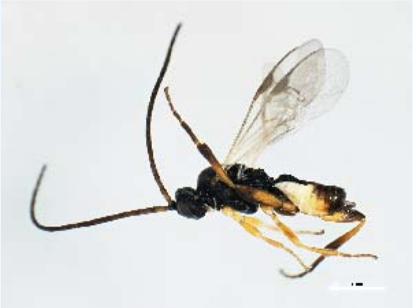

BIOUG00761-A09 [Lateral]
Apanteles ensiger
BIN URI: BOLD:AAA3764

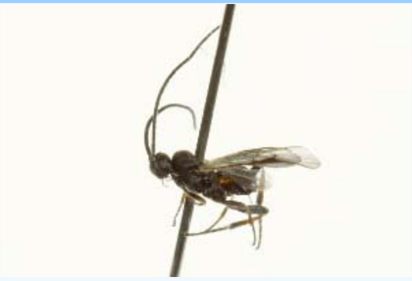

TDWG-0167 [Lateral]
Apanteles ensiger
BIN URI: BOLD:ACE6783

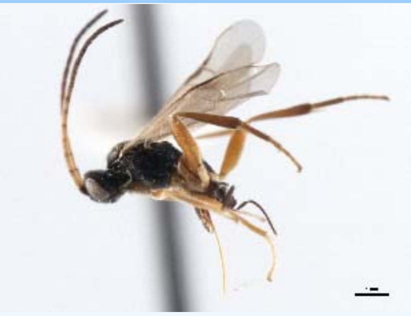

CNCHYM 00106 [Lateral]
Apanteles ensiger

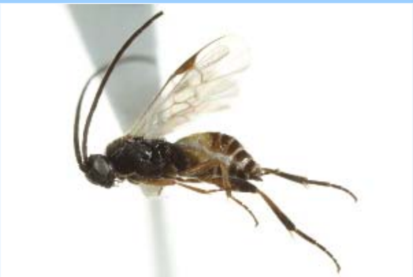

07PROBE-22328 [Lateral]
Apanteles ensiger
BIN URI: BOLD:AAA3764

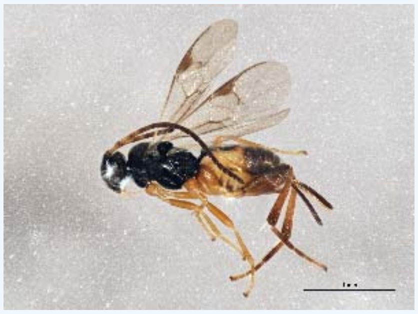

BIOUG03220-A01 [Lateral]
Apanteles ensiger
BIN URI: BOLD:AAA3764

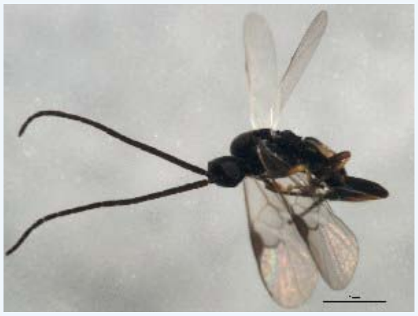

10BBCHY-1305 [Lateral]
Apanteles ensiger
BIN URI: BOLD:AAA3764

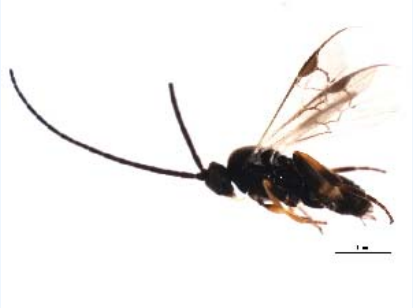

09BBEHY-2030 [Lateral]
Apanteles ensiger
BIN URI: BOLD:AAA3764

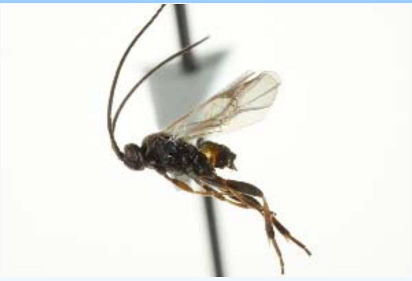

07PROBE-23393 [Lateral]
Apanteles ensiger
BIN URI: BOLD:AAA3764

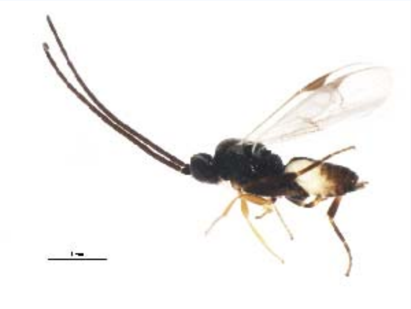

PCPP10-0466 [Lateral]
Apanteles ensiger
BIN URI: BOLD:AAA3764

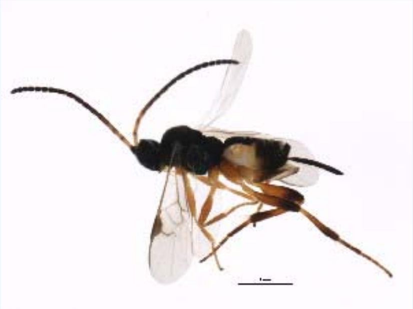

BIOUG00991-B03 [Lateral]
Apanteles ensiger
BIN URI: BOLD:AAA3764

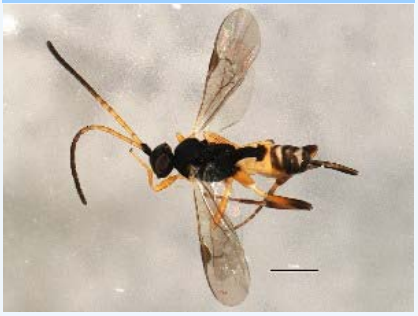

ASGLE-0880 [Lateral]
Apanteles ensiger
BIN URI: BOLD:AAA3764

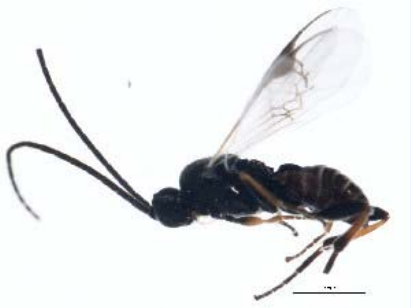

TDWG-0384 [Lateral]
Apanteles ensiger
BIN URI: BOLD:ACE6783

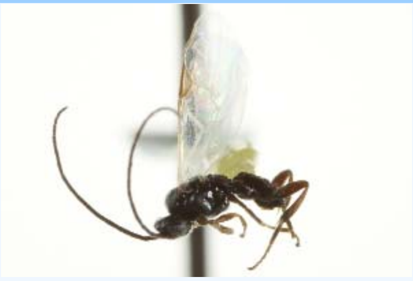

CBRA0491 [Lateral]
Apanteles sodalis

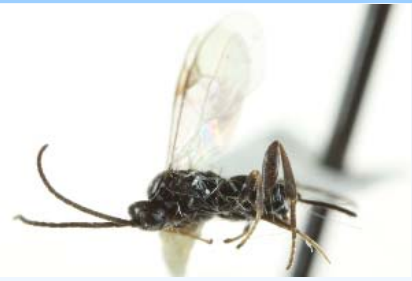

CBRA0494 [Lateral]
Apanteles sodalis
BIN URI: BOLD:AAM7223

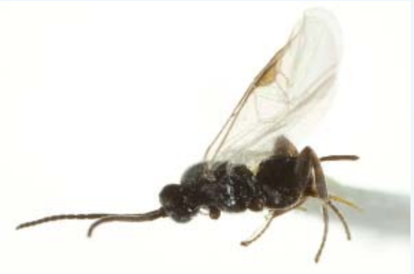

CBRA0492 [Lateral]
Apanteles sodalis

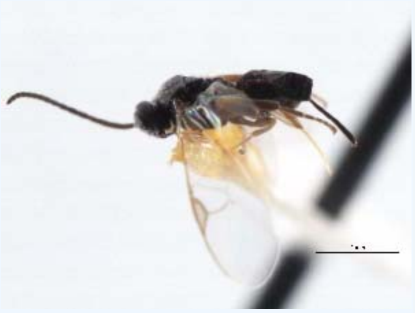

CNCHYM 00215 [Lateral]
Apanteles sodalis
BIN URI: BOLD:AAM7223

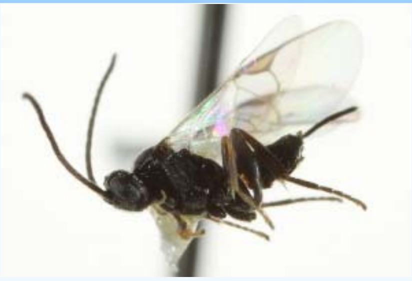

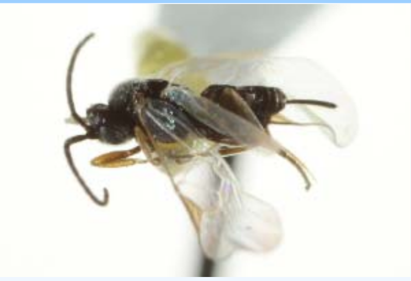

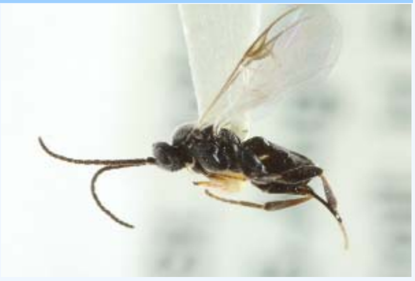

CBRA0493 [Lateral]
Apanteles sodalis
BIN URI: BOLD:AAM7223

CBRA0490 [Lateral]
Apanteles sodalis
BIN URI: BOLD:AAN1859

CBRA0495 [Lateral]
Apanteles sodalis
BIN URI: BOLD:AAM7223

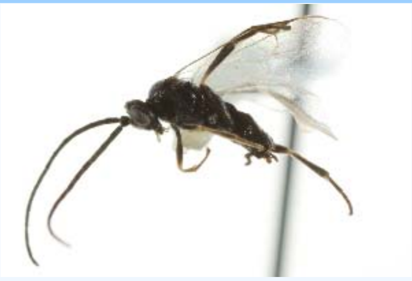

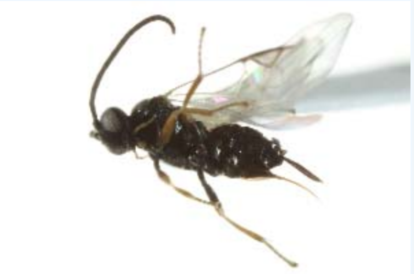

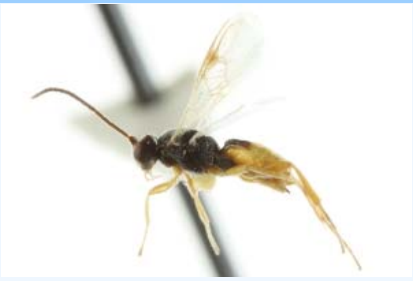

07PROBE-22321 [Lateral]
Apanteles xanthostigma
BIN URI: BOLD:AAB1922

07PROBE-20921 [Lateral]
Apanteles xanthostigma
BIN URI: BOLD:AAB1922

CNCHYM 00401 [Lateral]
Cotesia crambi

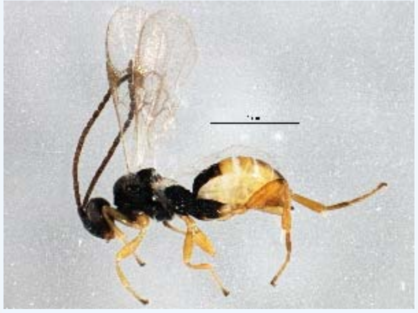

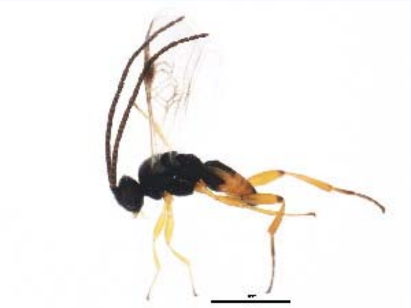

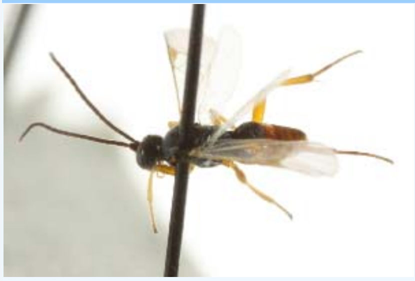

BIOUG03438-B06 [Lateral]
Cotesia crambi
BIN URI: BOLD:AAH1026

BIOUG01258-H06 [Lateral]
Cotesia crambi
BIN URI: BOLD:AAH1026

CNCHYM 00402 [Lateral]
Cotesia crambi
BIN URI: BOLD:AAH1026

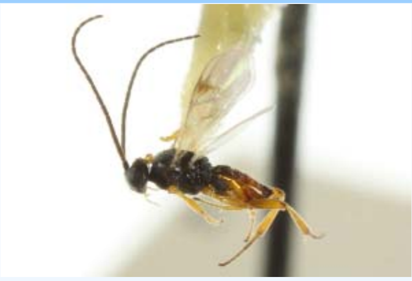

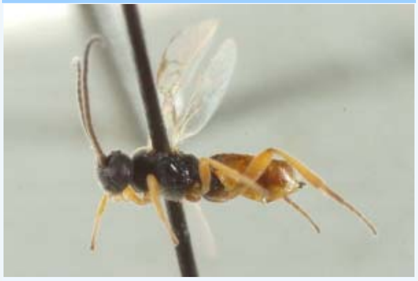

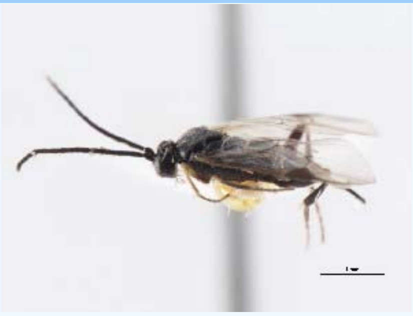

CNCHYM 00404 [Lateral]
Cotesia crambi

CNCHYM 00403 [Lateral]
Cotesia crambi
BIN URI: BOLD:AAH1026

CNCHYM 01091 [Lateral]
Dolichogenidea longicauda
BIN URI: BOLD:AAA5826

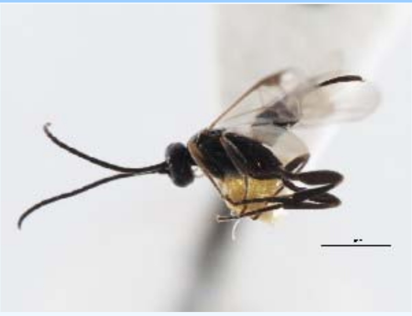

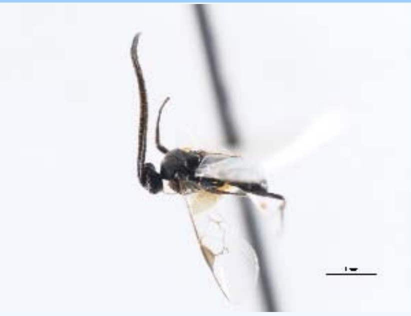

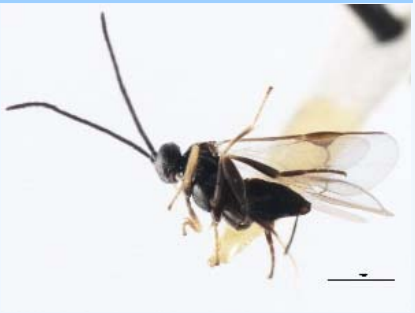

CNCHYM 01089 [Lateral]
Dolichogenidea longicauda
BIN URI: BOLD:AAA5826

CNCHYM 01093 [Lateral]
Dolichogenidea longicauda
BIN URI: BOLD:AAA5826

CNCHYM 00216 [Lateral]
Dolichogenidea longicauda
BIN URI: BOLD:AAA5826

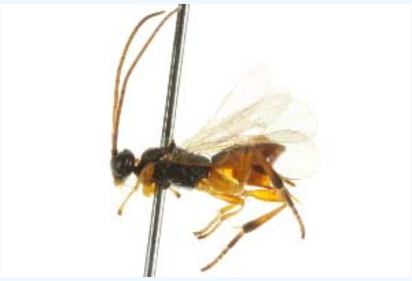
CNCHYM 01366 [Lateral]
Hygroplitis melligaster

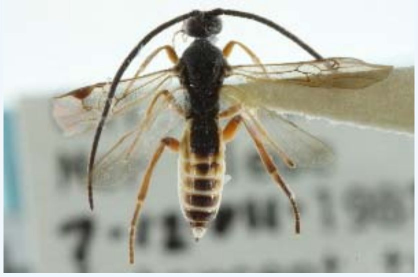
CNCHYM 01378 [Dorsal]
Hygroplitis melligaster

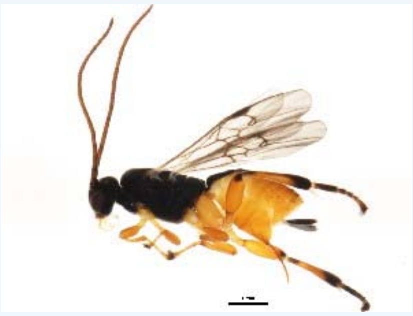
BIOUG01082-E12 [Lateral]
Hygroplitis melligaster
BIN URI: BOLD:AAC1797

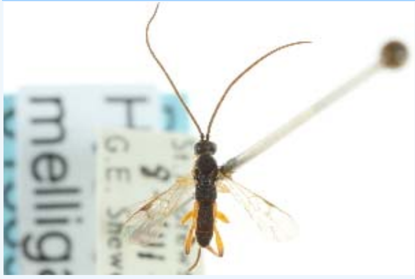
CNCHYM 01369 [Dorsal]
Hygroplitis melligaster

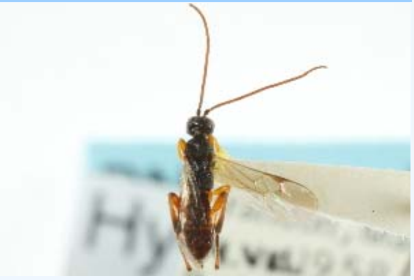
CNCHYM 01368 [Dorsal]
Hygroplitis melligaster
BIN URI: BOLD:AAC1797

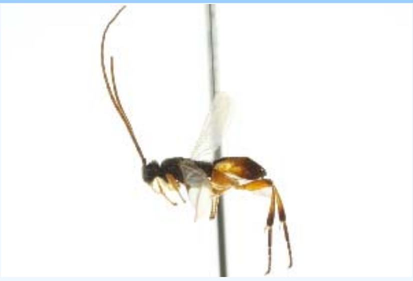
CNCHYM 01371 [Lateral]
Hygroplitis melligaster

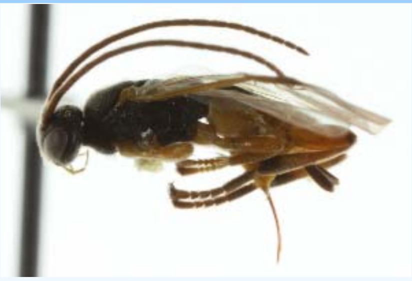
CNCHYM 01377 [Lateral]
Hygroplitis melligaster

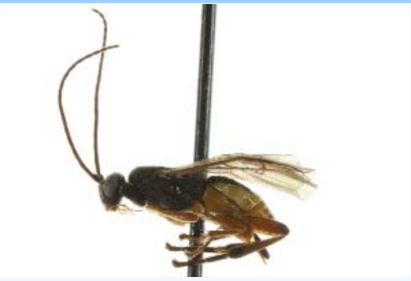
CNCHYM 01375 [Lateral]
Hygroplitis melligaster

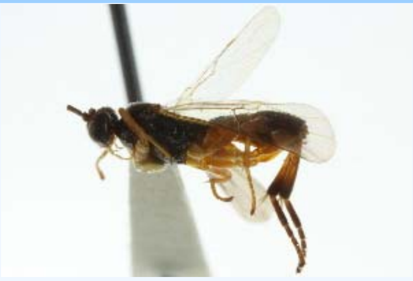
CNCHYM 01370 [Lateral]
Hygroplitis melligaster

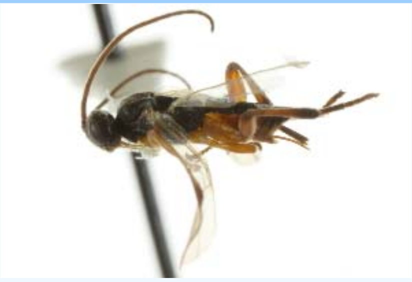
CNCHYM 01363 [Lateral]
Hygroplitis melligaster
BIN URI: BOLD:AAC1797

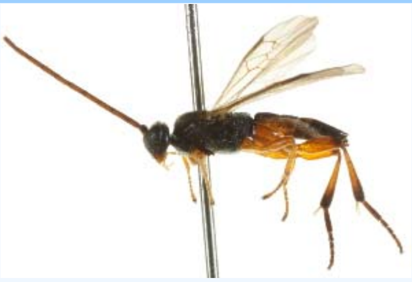
CNCHYM 01365 [Lateral]
Hygroplitis melligaster

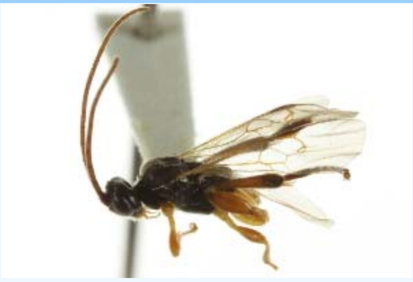
CNCHYM 01364 [Lateral]
Hygroplitis melligaster

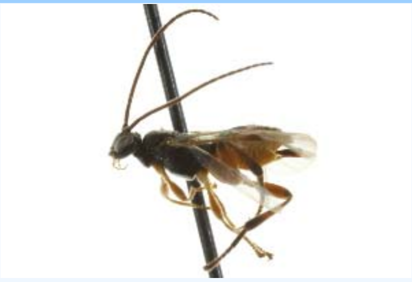
CNCHYM 01376 [Lateral]
Hygroplitis melligaster

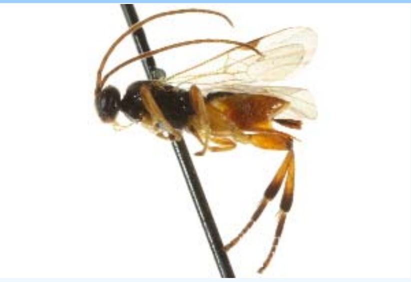
CNCHYM 01367 [Lateral]
Hygroplitis melligaster
BIN URI: BOLD:AAC1797

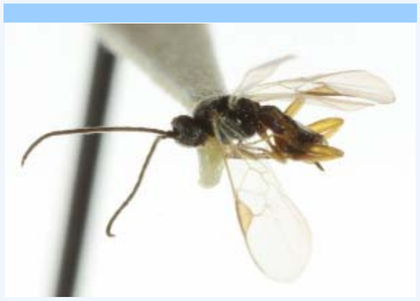
CNCHYM 03143 [Lateral]
Pholetesor bedelliae

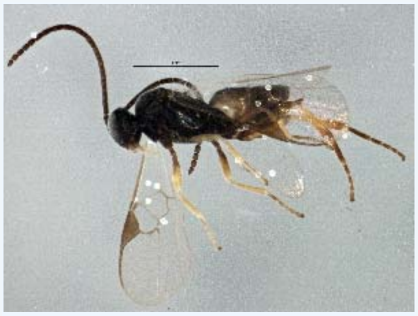

BIOUG03931-A10 [Lateral]
Pholetesor bedelliae
BIN URI: BOLD:AAA9172

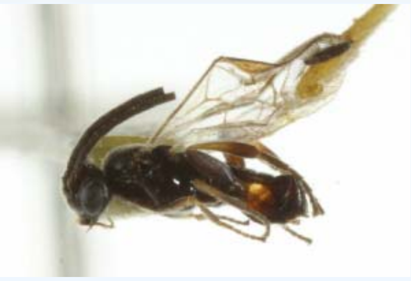

CNCHYM 03140 [Lateral]
Pholetesor bedelliae

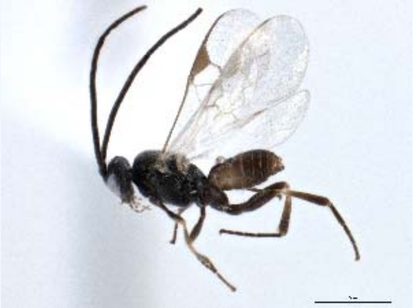

BIOUG04442-A12 [Lateral]
Pholetesor bedelliae
BIN URI: BOLD:AAA9172

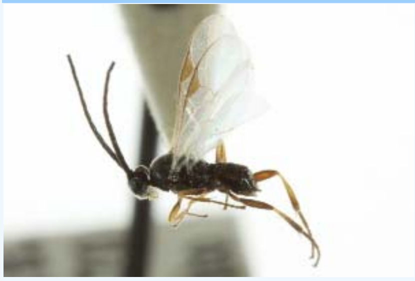

CNCHYM 03145 [Lateral]
Pholetesor bedelliae

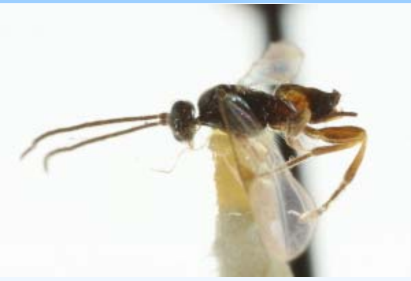

CNCHYM 03136 [Lateral]
Pholetesor bedelliae

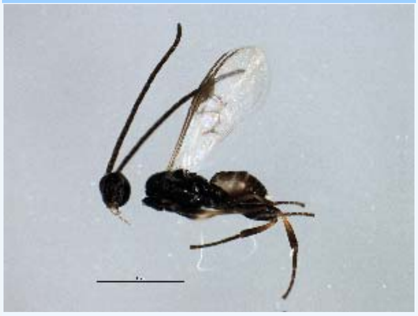

08BBHYM-1368 [Lateral]
Pholetesor bedelliae
BIN URI: BOLD:AAA9172

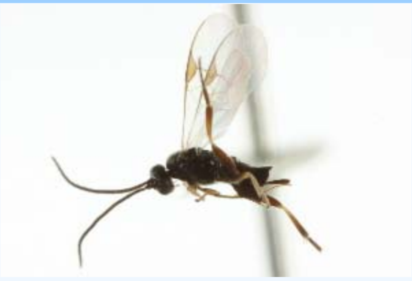

CNCHYM 03137 [Lateral]
Pholetesor bedelliae

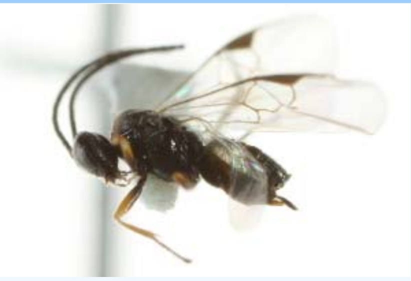

HYM00000819 [Lateral]
Pholetesor bedelliae

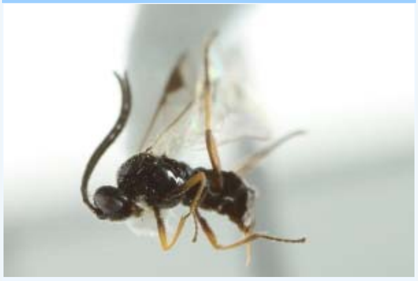

HYM00000920 [Lateral]
Pholetesor bedelliae
BIN URI: BOLD:AAA9172

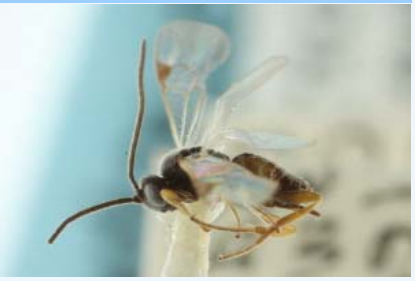

CNCHYM 03144 [Lateral]
Pholetesor bedelliae

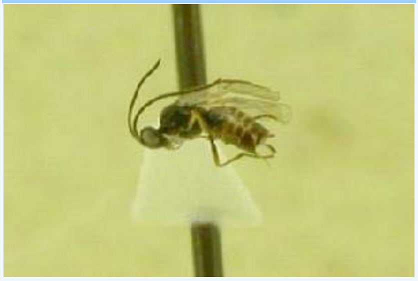

HYM00000331 [Lateral]
Pholetesor bedelliae
BIN URI: BOLD:AAA9172

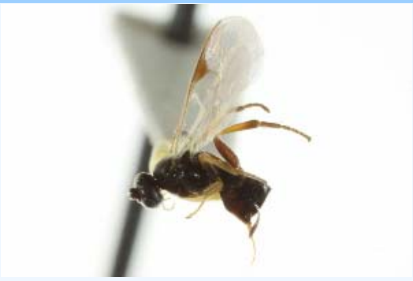

CNCHYM 03142 [Lateral]
Pholetesor bedelliae

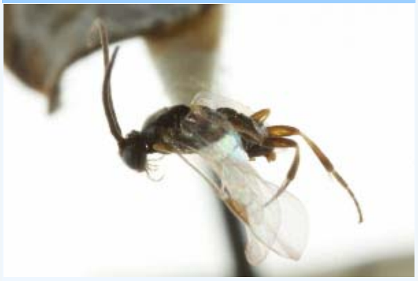

CNCHYM 03138 [Lateral]
Pholetesor bedelliae

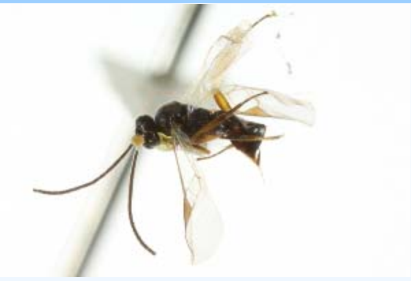

CNCHYM 03141 [Lateral]
Pholetesor bedelliae

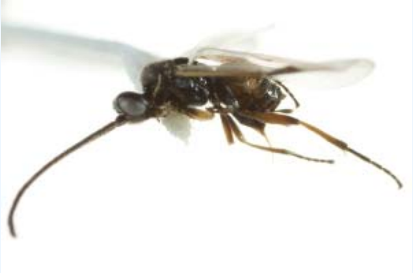

HYM00000818 [Lateral]
Pholetesor bedelliae

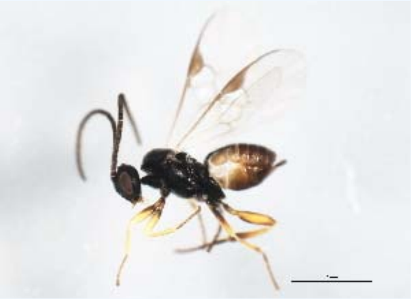

BIOUG00989-E07 [Lateral]
Pholetesor bedelliae
BIN URI: BOLD:AAA9172

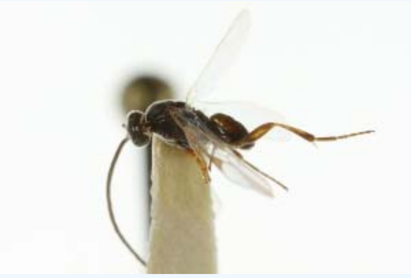

CNCHYM 03139 [Lateral]
Pholetesor bedelliae

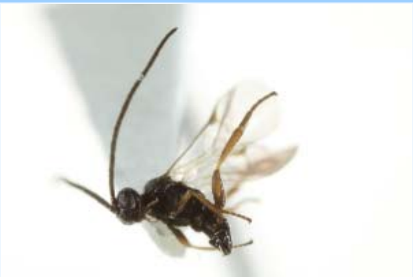

07PROBE-20808 [Lateral]
Pholetesor viminetorum
BIN URI: BOLD:AAA5660

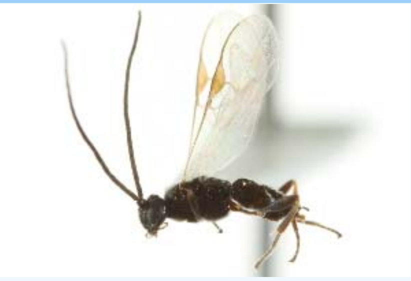

HYM00000810 [Lateral]
Pholetesor viminetorum

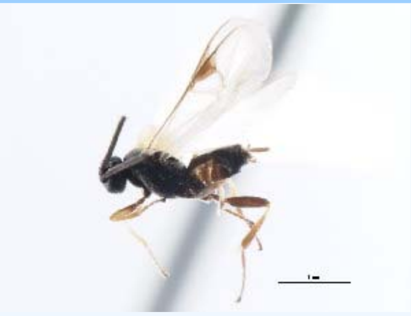

CNCHYM 03216 [Lateral]
Pholetesor viminetorum

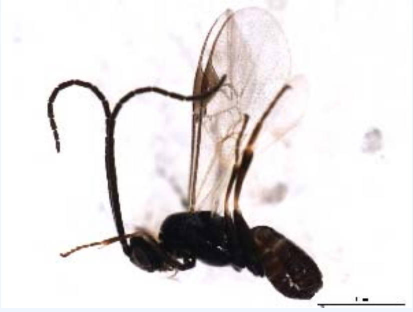

09PROBE-A0342 [Lateral]
Pholetesor viminetorum
BIN URI: BOLD:AAA5660

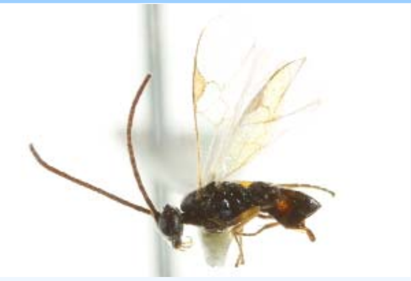

HYM00000800 [Lateral]
Pholetesor viminetorum

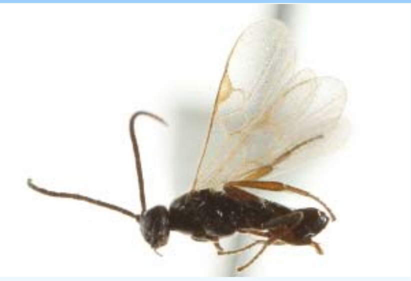

HYM00000801 [Lateral]
Pholetesor viminetorum

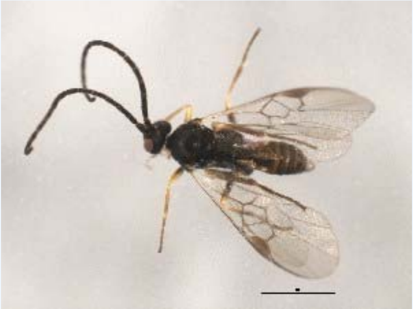

10BBCHY-2887 [Dorsal]
Pholetesor viminetorum
BIN URI: BOLD:AAA5660

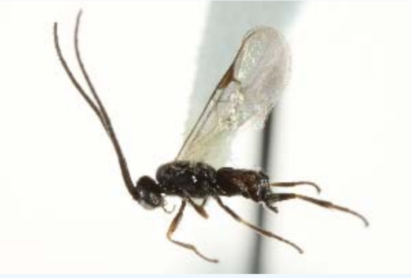

07PROBE-23399 [Lateral]
Pholetesor viminetorum
BIN URI: BOLD:AAA5660

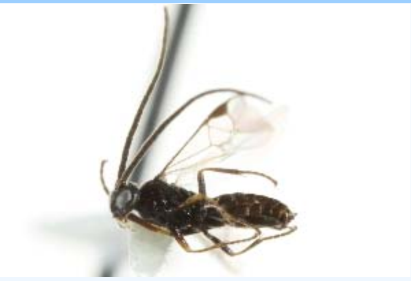

07PROBE-22423 [Lateral]
Pholetesor viminetorum
BIN URI: BOLD:AAA5660

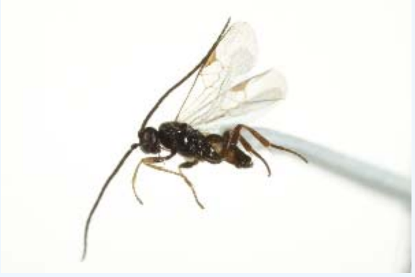

07PROBE-20789 [Lateral]
Pholetesor viminetorum
BIN URI: BOLD:AAA5660

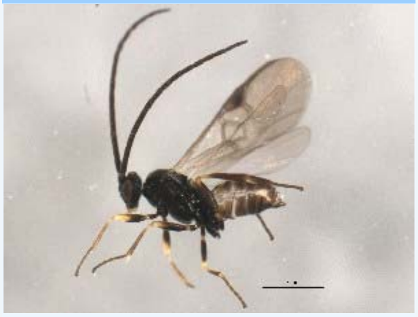

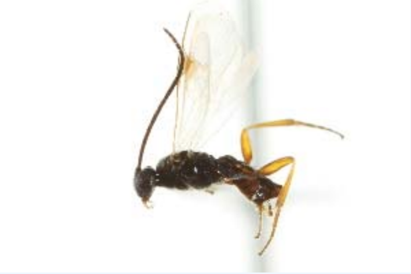

HYM00000921 [Lateral]
Pholetesor viminetorum

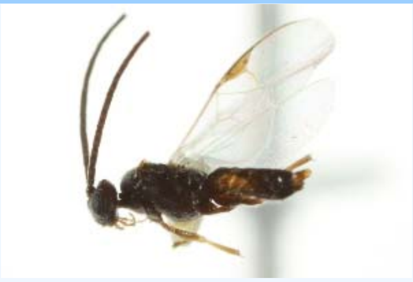

HYM00000816 [Lateral]
Pholetesor viminetorum

10BBCHY-2879 [Lateral]
Pholetesor viminetorum
BIN URI: BOLD:AAA5660

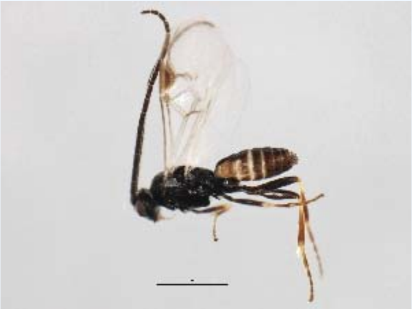

09BBEHY-1128 [Lateral]
Pholetesor viminetorum
BIN URI: BOLD:AAA5660

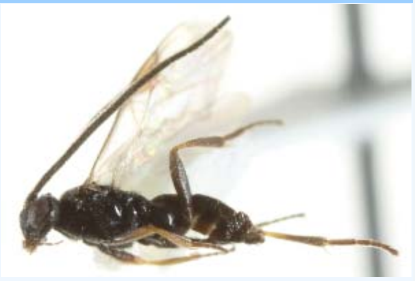

07PROBE-23413 [Lateral]
Pholetesor viminetorum
BIN URI: BOLD:AAA5660

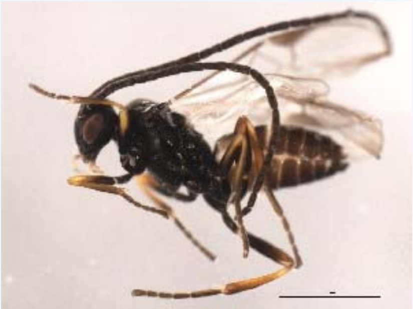

10PROBE-29276 [Lateral]
Pholetesor viminetorum
BIN URI: BOLD:AAA5660

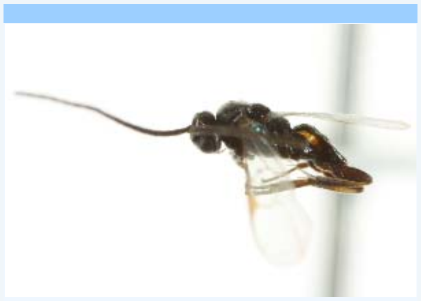

HYM00000812 [Lateral]
Pholetesor viminetorum

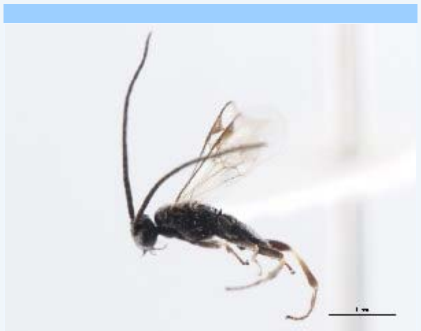

CNCHYM 03217 [Lateral]
Pholetesor viminetorum

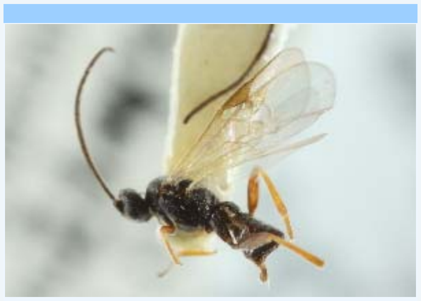

HYM00000792 [Lateral]
Pholetesor viminetorum

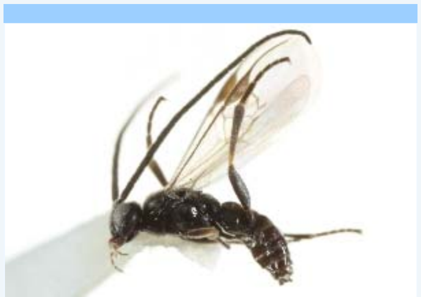

07PROBE-22417 [Lateral]
Pholetesor viminetorum
BIN URI: BOLD:AAA5660

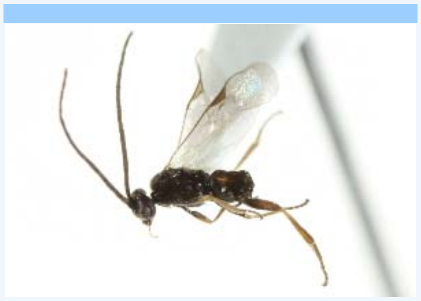

07PROBE-20818 [Lateral]
Pholetesor viminetorum
BIN URI: BOLD:AAA5660

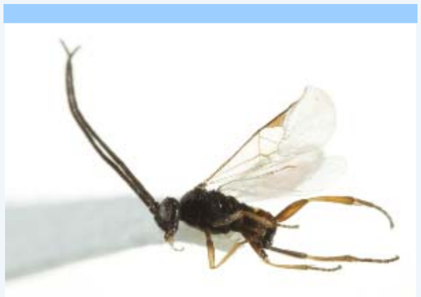

07PROBE-20806 [Lateral]
Pholetesor viminetorum
BIN URI: BOLD:AAA5660

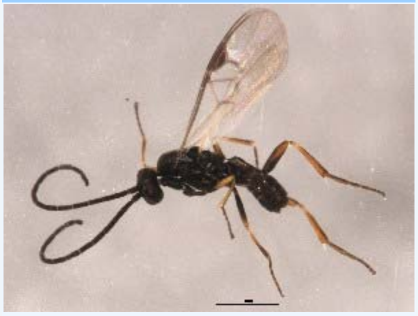

10PROBE-28643 [Lateral]
Pholetesor viminetorum
BIN URI: BOLD:AAA5660

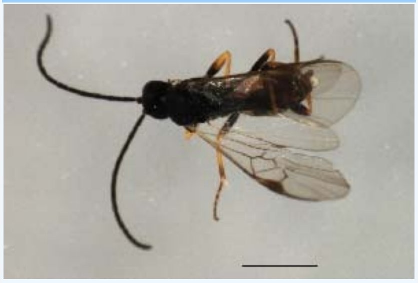

08BBHYM-1493 [Dorsal]
Pholetesor viminetorum
BIN URI: BOLD:AAA5660

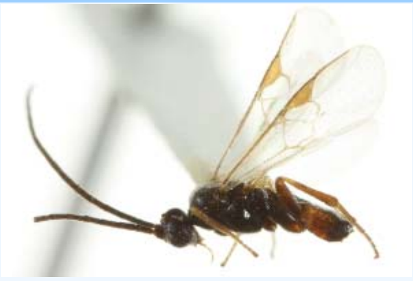

HYM00000809 [Lateral]
Pholetesor viminetorum

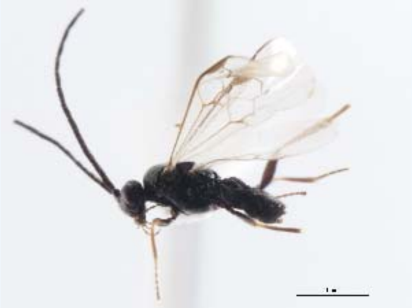

CNCHYM 03218 [Lateral]
Pholetesor viminetorum

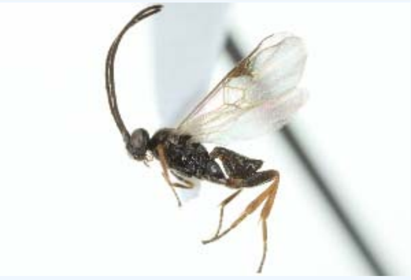

07PROBE-24572 [Lateral]
Pholetesor viminetorum
BIN URI: BOLD:AAA5660

Copyright © 2014 BOLD Systems. All rights reserved.
