# Supplementary material for: Streamlining the use of BOLD specimen data to record species distributions: a case study with ten Nearctic species of Microgastrinae (Hymenoptera: Braconidae)
Source: Biodivers Data J. 2014 Oct 29;(2):e4153. doi: 10.3897/BDJ.2.e4153 (PMC4251541; doi:10.3897/BDJ.2.e4153)
Supplement: Supplementary material 4 — Supplemental Appendix 4 [file biodiversity_data_journal-2-e4153-s004.pdf]

BOLD TaxonID Tree

Title	:	Nearctic Microgastrinae Case Study Specimens [DS-NEAMICCS]
Date	:	24-September-2014
Data Type	:	Nucleotide
Distance Model	:	Kimura 2 Parameter
Marker	:	COI-5P
Codon Positions	:	1st, 2nd, 3rd
Labels	:	ProcessID, Sequence Length, BIN uri
Filters	:	Length > 300, Contaminants, Stop Condons, Flagged,
Colorization	:	[blue]=Stop Codons [red]=Contamination or misidentification
Attachment	:	Photographs & Spreadsheet

Sequence Count	:	514
Species count	:	10
Genus count	:	5
Family count	:	1
Unidentified	:	0

BIN Count	:	13

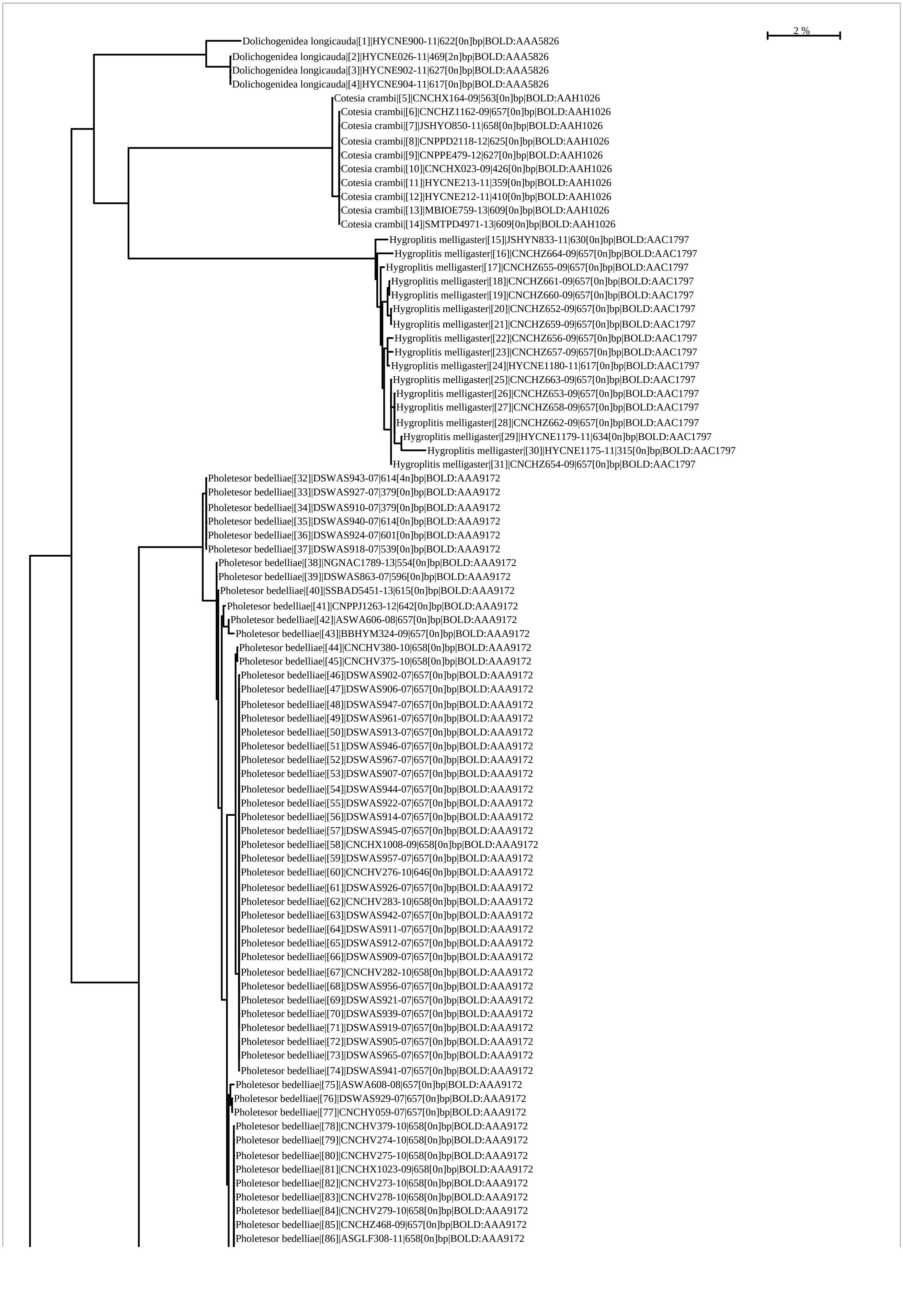
2 %
Dolichogenidea longicauda|[1]|HYCNE900-11|622[0n]bp|BOLD:AAA5826
Dolichogenidea longicauda|[2]|HYCNE026-11|469[2n]bp|BOLD:AAA5826
Dolichogenidea longicauda|[3]|HYCNE902-11|627[0n]bp|BOLD:AAA5826
Dolichogenidea longicauda|[4]|HYCNE904-11|617[0n]bp|BOLD:AAA5826
Cotesia crambi|[5]|CNCHX164-09|563[0n]bp|BOLD:AAH1026
Cotesia crambi|[6]|CNCHZ1162-09|657[0n]bp|BOLD:AAH1026
Cotesia crambi|[7]|JSHYO850-11|658[0n]bp|BOLD:AAH1026
Cotesia crambi|[8]|CNPPD2118-12|625[0n]bp|BOLD:AAH1026
Cotesia crambi|[9]|CNPPE479-12|627[0n]bp|BOLD:AAH1026
Cotesia crambi|[10]|CNCHX023-09|426[0n]bp|BOLD:AAH1026
Cotesia crambi|[11]|HYCNE213-11|359[0n]bp|BOLD:AAH1026
Cotesia crambi|[12]|HYCNE212-11|410[0n]bp|BOLD:AAH1026
Cotesia crambi|[13]|MBIOE759-13|609[0n]bp|BOLD:AAH1026
Cotesia crambi|[14]|SMTPD4971-13|609[0n]bp|BOLD:AAH1026
Hygroplitis melligaster|[15]|JSHYN833-11|630[0n]bp|BOLD:AAC1797
Hygroplitis melligaster|[16]|CNCHZ664-09|657[0n]bp|BOLD:AAC1797
Hygroplitis melligaster|[17]|CNCHZ655-09|657[0n]bp|BOLD:AAC1797
Hygroplitis melligaster|[18]|CNCHZ661-09|657[0n]bp|BOLD:AAC1797
Hygroplitis melligaster|[19]|CNCHZ660-09|657[0n]bp|BOLD:AAC1797
Hygroplitis melligaster|[20]|CNCHZ652-09|657[0n]bp|BOLD:AAC1797
Hygroplitis melligaster|[21]|CNCHZ659-09|657[0n]bp|BOLD:AAC1797
Hygroplitis melligaster|[22]|CNCHZ656-09|657[0n]bp|BOLD:AAC1797
Hygroplitis melligaster|[23]|CNCHZ657-09|657[0n]bp|BOLD:AAC1797
Hygroplitis melligaster|[24]|HYCNE1180-11|617[0n]bp|BOLD:AAC1797
Hygroplitis melligaster|[25]|CNCHZ663-09|657[0n]bp|BOLD:AAC1797
Hygroplitis melligaster|[26]|CNCHZ653-09|657[0n]bp|BOLD:AAC1797
Hygroplitis melligaster|[27]|CNCHZ658-09|657[0n]bp|BOLD:AAC1797
Hygroplitis melligaster|[28]|CNCHZ662-09|657[0n]bp|BOLD:AAC1797
Hygroplitis melligaster|[29]|HYCNE1179-11|634[0n]bp|BOLD:AAC1797
Hygroplitis melligaster|[30]|HYCNE1175-11|315[0n]bp|BOLD:AAC1797
Hygroplitis melligaster|[31]|CNCHZ654-09|657[0n]bp|BOLD:AAC1797
Pholetesor bedelliae|[32]|DSWAS943-07|614[4n]bp|BOLD:AAA9172
Pholetesor bedelliae|[33]|DSWAS927-07|379[0n]bp|BOLD:AAA9172
Pholetesor bedelliae|[34]|DSWAS910-07|379[0n]bp|BOLD:AAA9172
Pholetesor bedelliae|[35]|DSWAS940-07|614[0n]bp|BOLD:AAA9172
Pholetesor bedelliae|[36]|DSWAS924-07|601[0n]bp|BOLD:AAA9172
Pholetesor bedelliae|[37]|DSWAS918-07|539[0n]bp|BOLD:AAA9172
Pholetesor bedelliae|[38]|NGNAC1789-13|554[0n]bp|BOLD:AAA9172
Pholetesor bedelliae|[39]|DSWAS863-07|596[0n]bp|BOLD:AAA9172
Pholetesor bedelliae|[40]|SSBAD5451-13|615[0n]bp|BOLD:AAA9172
Pholetesor bedelliae|[41]|CNPPJ1263-12|642[0n]bp|BOLD:AAA9172
Pholetesor bedelliae|[42]|ASWA606-08|657[0n]bp|BOLD:AAA9172
Pholetesor bedelliae|[43]|BBHYM324-09|657[0n]bp|BOLD:AAA9172
Pholetesor bedelliae|[44]|CNCHV380-10|658[0n]bp|BOLD:AAA9172
Pholetesor bedelliae|[45]|CNCHV375-10|658[0n]bp|BOLD:AAA9172
Pholetesor bedelliae|[46]|DSWAS902-07|657[0n]bp|BOLD:AAA9172
Pholetesor bedelliae|[47]|DSWAS906-07|657[0n]bp|BOLD:AAA9172
Pholetesor bedelliae|[48]|DSWAS947-07|657[0n]bp|BOLD:AAA9172
Pholetesor bedelliae|[49]|DSWAS961-07|657[0n]bp|BOLD:AAA9172
Pholetesor bedelliae|[50]|DSWAS913-07|657[0n]bp|BOLD:AAA9172
Pholetesor bedelliae|[51]|DSWAS946-07|657[0n]bp|BOLD:AAA9172
Pholetesor bedelliae|[52]|DSWAS967-07|657[0n]bp|BOLD:AAA9172
Pholetesor bedelliae|[53]|DSWAS907-07|657[0n]bp|BOLD:AAA9172
Pholetesor bedelliae|[54]|DSWAS944-07|657[0n]bp|BOLD:AAA9172
Pholetesor bedelliae|[55]|DSWAS922-07|657[0n]bp|BOLD:AAA9172
Pholetesor bedelliae|[56]|DSWAS914-07|657[0n]bp|BOLD:AAA9172
Pholetesor bedelliae|[57]|DSWAS945-07|657[0n]bp|BOLD:AAA9172
Pholetesor bedelliae|[58]|CNCHX1008-09|658[0n]bp|BOLD:AAA9172
Pholetesor bedelliae|[59]|DSWAS957-07|657[0n]bp|BOLD:AAA9172
Pholetesor bedelliae|[60]|CNCHV276-10|646[0n]bp|BOLD:AAA9172
Pholetesor bedelliae|[61]|DSWAS926-07|657[0n]bp|BOLD:AAA9172
Pholetesor bedelliae|[62]|CNCHV283-10|658[0n]bp|BOLD:AAA9172
Pholetesor bedelliae|[63]|DSWAS942-07|657[0n]bp|BOLD:AAA9172
Pholetesor bedelliae|[64]|DSWAS911-07|657[0n]bp|BOLD:AAA9172
Pholetesor bedelliae|[65]|DSWAS912-07|657[0n]bp|BOLD:AAA9172
Pholetesor bedelliae|[66]|DSWAS909-07|657[0n]bp|BOLD:AAA9172
Pholetesor bedelliae|[67]|CNCHV282-10|658[0n]bp|BOLD:AAA9172
Pholetesor bedelliae|[68]|DSWAS956-07|657[0n]bp|BOLD:AAA9172
Pholetesor bedelliae|[69]|DSWAS921-07|657[0n]bp|BOLD:AAA9172
Pholetesor bedelliae|[70]|DSWAS939-07|657[0n]bp|BOLD:AAA9172
Pholetesor bedelliae|[71]|DSWAS919-07|657[0n]bp|BOLD:AAA9172
Pholetesor bedelliae|[72]|DSWAS905-07|657[0n]bp|BOLD:AAA9172
Pholetesor bedelliae|[73]|DSWAS965-07|657[0n]bp|BOLD:AAA9172
Pholetesor bedelliae|[74]|DSWAS941-07|657[0n]bp|BOLD:AAA9172
Pholetesor bedelliae|[75]|ASWA608-08|657[0n]bp|BOLD:AAA9172
Pholetesor bedelliae|[76]|DSWAS929-07|657[0n]bp|BOLD:AAA9172
Pholetesor bedelliae|[77]|CNCHY059-07|657[0n]bp|BOLD:AAA9172
Pholetesor bedelliae|[78]|CNCHV379-10|658[0n]bp|BOLD:AAA9172
Pholetesor bedelliae|[79]|CNCHV274-10|658[0n]bp|BOLD:AAA9172
Pholetesor bedelliae|[80]|CNCHV275-10|658[0n]bp|BOLD:AAA9172
Pholetesor bedelliae|[81]|CNCHX1023-09|658[0n]bp|BOLD:AAA9172
Pholetesor bedelliae|[82]|CNCHV273-10|658[0n]bp|BOLD:AAA9172
Pholetesor bedelliae|[83]|CNCHV278-10|658[0n]bp|BOLD:AAA9172
Pholetesor bedelliae|[84]|CNCHV279-10|658[0n]bp|BOLD:AAA9172
Pholetesor bedelliae|[85]|CNCHZ468-09|657[0n]bp|BOLD:AAA9172
Pholetesor bedelliae|[86]|ASGLF308-11|658[0n]bp|BOLD:AAA9172

Pholetesor bedelliae|[84]|CNCHV279-10|658[0n]bp|BOLD:AAA9172
Pholetesor bedelliae|[85]|CNCHZ468-09|657[0n]bp|BOLD:AAA9172
Pholetesor bedelliae|[86]|ASGLF308-11|658[0n]bp|BOLD:AAA9172
Pholetesor bedelliae|[87]|CNCHX1002-09|658[0n]bp|BOLD:AAA9172
Pholetesor bedelliae|[88]|CNCHX980-09|658[0n]bp|BOLD:AAA9172
Pholetesor bedelliae|[89]|CNCHX1010-09|648[0n]bp|BOLD:AAA9172
Pholetesor bedelliae|[90]|CNCHX970-09|652[0n]bp|BOLD:AAA9172
Pholetesor bedelliae|[91]|CNCHZ627-09|657[0n]bp|BOLD:AAA9172
Pholetesor bedelliae|[92]|DSWAS958-07|657[0n]bp|BOLD:AAA9172
Pholetesor viminetorum|[93]|SAHYM373-10|658[0n]bp|BOLD:AAA5660
Pholetesor viminetorum|[94]|ASWAX965-08|657[0n]bp|BOLD:AAA5660
Pholetesor viminetorum|[95]|ASWAV958-08|632[0n]bp|BOLD:AAA5660
Pholetesor viminetorum|[96]|CNCHY332-07|657[0n]bp|BOLD:AAA5660
Pholetesor viminetorum|[97]|DSWAS960-07|657[0n]bp|BOLD:AAA5660
Pholetesor viminetorum|[98]|CNCHY331-07|657[0n]bp|BOLD:AAA5660
Pholetesor viminetorum|[99]|CNEIB1042-12|633[0n]bp|BOLD:AAA5660
Pholetesor viminetorum|[100]|DSWAS903-07|657[0n]bp|BOLD:AAA5660
Pholetesor viminetorum|[101]|CNSLP346-13|575[0n]bp|BOLD:AAA5660
Pholetesor viminetorum|[102]|CNRME2000-12|613[0n]bp|BOLD:AAA5660
Pholetesor viminetorum|[103]|CNRMF2687-12|624[0n]bp|BOLD:AAA5660
Pholetesor viminetorum|[104]|CNCHZ915-09|657[0n]bp|BOLD:AAA5660
Pholetesor viminetorum|[105]|CNCHZ624-09|613[0n]bp|BOLD:AAA5660
Pholetesor viminetorum|[106]|CNCHX081-09|658[0n]bp|BOLD:AAA5660
Pholetesor viminetorum|[107]|CNCHX066-09|658[0n]bp|BOLD:AAA5660
Pholetesor viminetorum|[108]|CNCHZ576-09|657[0n]bp|BOLD:AAA5660
Pholetesor viminetorum|[109]|CNCHZ577-09|657[0n]bp|BOLD:AAA5660
Pholetesor viminetorum|[110]|CNCHZ617-09|657[0n]bp|BOLD:AAA5660
Pholetesor viminetorum|[111]|CNCHZ575-09|657[0n]bp|BOLD:AAA5660
Pholetesor viminetorum|[112]|CNCHZ602-09|657[0n]bp|BOLD:AAA5660
Pholetesor viminetorum|[113]|CNCHZ613-09|657[0n]bp|BOLD:AAA5660
Pholetesor viminetorum|[114]|CNCHZ606-09|657[0n]bp|BOLD:AAA5660
Pholetesor viminetorum|[115]|CNCHZ607-09|657[0n]bp|BOLD:AAA5660
Pholetesor viminetorum|[116]|CNCHZ625-09|657[0n]bp|BOLD:AAA5660
Pholetesor viminetorum|[117]|CNCHZ619-09|657[0n]bp|BOLD:AAA5660
Pholetesor viminetorum|[118]|CNCHZ914-09|657[0n]bp|BOLD:AAA5660
Pholetesor viminetorum|[119]|CNCHZ609-09|657[0n]bp|BOLD:AAA5660
Pholetesor viminetorum|[120]|CNCHZ608-09|657[0n]bp|BOLD:AAA5660
Pholetesor viminetorum|[121]|CNCHZ611-09|657[3n]bp|BOLD:AAA5660
Pholetesor viminetorum|[122]|CNCHZ612-09|657[0n]bp|BOLD:AAA5660
Pholetesor viminetorum|[123]|CNCHZ913-09|631[0n]bp|BOLD:AAA5660
Pholetesor viminetorum|[124]|ASWA598-08|657[0n]bp|BOLD:AAA5660
Pholetesor viminetorum|[125]|CNCHZ618-09|657[0n]bp|BOLD:AAA5660
Pholetesor viminetorum|[126]|CNCHZ555-09|657[0n]bp|BOLD:AAA5660
Pholetesor viminetorum|[127]|CNCHZ574-09|657[0n]bp|BOLD:AAA5660
Pholetesor viminetorum|[128]|CNCHZ628-09|657[0n]bp|BOLD:AAA5660
Pholetesor viminetorum|[129]|CNCHZ604-09|657[0n]bp|BOLD:AAA5660
Pholetesor viminetorum|[130]|CNCHZ620-09|657[0n]bp|BOLD:AAA5660
Pholetesor viminetorum|[131]|CNCHZ605-09|657[0n]bp|BOLD:AAA5660
Pholetesor viminetorum|[132]|CNCHZ621-09|657[0n]bp|BOLD:AAA5660
Pholetesor viminetorum|[133]|CNCHZ622-09|657[0n]bp|BOLD:AAA5660
Pholetesor viminetorum|[134]|CNCHZ616-09|657[0n]bp|BOLD:AAA5660
Pholetesor viminetorum|[135]|CNCHZ1092-09|612[0n]bp|BOLD:AAA5660
Pholetesor viminetorum|[136]|ASWAT348-08|654[0n]bp|BOLD:AAA5660
Pholetesor viminetorum|[137]|ASWAT346-08|657[0n]bp|BOLD:AAA5660
Pholetesor viminetorum|[138]|ASWAT329-08|644[0n]bp|BOLD:AAA5660
Pholetesor viminetorum|[139]|ASWAX979-08|657[0n]bp|BOLD:AAA5660
Pholetesor viminetorum|[140]|ASWA615-08|657[0n]bp|BOLD:AAA5660
Pholetesor viminetorum|[141]|CNCHX085-09|658[0n]bp|BOLD:AAA5660
Pholetesor viminetorum|[142]|CNCHZ1101-09|657[0n]bp|BOLD:AAA5660
Pholetesor viminetorum|[143]|CNCHX088-09|658[0n]bp|BOLD:AAA5660
Pholetesor viminetorum|[144]|CNCHX082-09|658[0n]bp|BOLD:AAA5660
Pholetesor viminetorum|[145]|CNCHX087-09|658[0n]bp|BOLD:AAA5660
Pholetesor viminetorum|[146]|SSGBB2792-14|600[0n]bp|BOLD:AAA5660
Pholetesor viminetorum|[147]|CNCHZ1135-09|657[0n]bp|BOLD:AAA5660
Pholetesor viminetorum|[148]|CNCHZ1115-09|632[0n]bp|BOLD:AAA5660
Pholetesor viminetorum|[149]|CNCHX083-09|658[0n]bp|BOLD:AAA5660
Pholetesor viminetorum|[150]|CNCHX089-09|658[0n]bp|BOLD:AAA5660
Pholetesor viminetorum|[151]|CNCHX074-09|658[0n]bp|BOLD:AAA5660
Pholetesor viminetorum|[152]|CNCHZ1139-09|657[0n]bp|BOLD:AAA5660
Pholetesor viminetorum|[153]|CNCHX078-09|658[0n]bp|BOLD:AAA5660
Pholetesor viminetorum|[154]|CNCHX075-09|658[0n]bp|BOLD:AAA5660
Pholetesor viminetorum|[155]|CNCHV431-10|658[0n]bp|BOLD:AAA5660
Pholetesor viminetorum|[156]|ASWAT358-08|657[0n]bp|BOLD:AAA5660
Pholetesor viminetorum|[157]|JBHCH335-10|632[0n]bp|BOLD:AAA5660
Pholetesor viminetorum|[158]|ASWAY590-08|305[0n]bp|
Pholetesor viminetorum|[159]|JBHCH968-10|658[0n]bp|BOLD:AAA5660
Pholetesor viminetorum|[160]|ASWAV964-08|613[0n]bp|BOLD:AAA5660
Pholetesor viminetorum|[161]|CNCHZ593-09|657[0n]bp|BOLD:AAA5660
Pholetesor viminetorum|[162]|BBHYE128-10|658[0n]bp|BOLD:AAA5660
Pholetesor viminetorum|[163]|CNCHZ573-09|657[0n]bp|BOLD:AAA5660
Pholetesor viminetorum|[164]|CNCHZ572-09|657[0n]bp|BOLD:AAA5660
Pholetesor viminetorum|[165]|WOMIA358-11|658[0n]bp|BOLD:AAA5660
Pholetesor viminetorum|[166]|WOMIA360-11|658[0n]bp|BOLD:AAA5660
Pholetesor viminetorum|[167]|DSWAS952-07|657[0n]bp|BOLD:AAA5660
Pholetesor viminetorum|[168]|DSWAS935-07|657[0n]bp|BOLD:AAA5660
Pholetesor viminetorum|[169]|DSWAS916-07|657[0n]bp|BOLD:AAA5660
Pholetesor viminetorum|[170]|DSWAS933-07|657[0n]bp|BOLD:AAA5660
Pholetesor viminetorum|[171]|WOMIA359-11|658[0n]bp|BOLD:AAA5660
Pholetesor viminetorum|[172]|DSWAS915-07|657[0n]bp|BOLD:AAA5660

Pholetesor viminetorum|[170]|DSWAS933-07|657[0n]bp|BOLD:AAA5660
Pholetesor viminetorum|[171]|WOMIA359-11|658[0n]bp|BOLD:AAA5660
Pholetesor viminetorum|[172]|DSWAS915-07|657[0n]bp|BOLD:AAA5660
Pholetesor viminetorum|[173]|DSWAS953-07|657[0n]bp|BOLD:AAA5660
Pholetesor viminetorum|[174]|WOMIA352-11|658[0n]bp|BOLD:AAA5660
Pholetesor viminetorum|[175]|BBHYK928-10|658[0n]bp|BOLD:AAA5660
Pholetesor viminetorum|[176]|BBHYK920-10|658[0n]bp|BOLD:AAA5660
Pholetesor viminetorum|[177]|BBHYM449-09|658[0n]bp|BOLD:AAA5660
Pholetesor viminetorum|[178]|SSWLB5177-13|567[0n]bp|BOLD:AAA5660
Apanteles sodalis|[179]|CNCHV582-10|658[1n]bp|BOLD:AAN1859
Apanteles sodalis|[180]|CNCHV587-10|658[0n]bp|BOLD:AAM7223
Apanteles sodalis|[181]|HYCNE025-11|617[0n]bp|BOLD:AAM7223
Apanteles sodalis|[182]|CNCHV585-10|658[0n]bp|BOLD:AAM7223
Apanteles sodalis|[183]|CNCHV586-10|658[0n]bp|BOLD:AAM7223
Apanteles ensiger|[184]|CNSLS106-13|579[0n]bp|BOLD:AAA3764
Apanteles ensiger|[185]|CNPKJ2479-14|594[0n]bp|BOLD:AAA3764
Apanteles ensiger|[186]|CNPKO2958-14|595[0n]bp|BOLD:AAA3764
Apanteles ensiger|[187]|CNPKO3073-14|594[0n]bp|BOLD:AAA3764
Apanteles ensiger|[188]|CNMIG324-14|594[0n]bp|BOLD:AAA3764
Apanteles ensiger|[189]|CNPKJ2122-14|597[0n]bp|BOLD:AAA3764
Apanteles ensiger|[190]|CNPKK1009-14|531[0n]bp|BOLD:AAA3764
Apanteles ensiger|[191]|CNMIG1286-14|585[0n]bp|BOLD:AAA3764
Apanteles ensiger|[192]|CNGBN2850-14|588[0n]bp|BOLD:AAA3764
Apanteles ensiger|[193]|CNSLS117-13|607[0n]bp|BOLD:AAA3764
Apanteles ensiger|[194]|RBINA1006-13|603[0n]bp|BOLD:AAA3764
Apanteles ensiger|[195]|CNPKJ648-14|590[0n]bp|BOLD:AAA3764
Apanteles ensiger|[196]|CNMIG137-14|588[0n]bp|BOLD:AAA3764
Apanteles ensiger|[197]|CNSLS115-13|607[0n]bp|BOLD:AAA3764
Apanteles ensiger|[198]|CNPKO1368-14|561[0n]bp|BOLD:AAA3764
Apanteles ensiger|[199]|CNPKO1489-14|576[0n]bp|BOLD:AAA3764
Apanteles ensiger|[200]|CNKJF1651-14|546[0n]bp|BOLD:AAA3764
Apanteles ensiger|[201]|CNMIG236-14|579[0n]bp|BOLD:AAA3764
Apanteles ensiger|[202]|CNLMG1225-14|579[0n]bp|BOLD:AAA3764
Apanteles ensiger|[203]|CNSLS119-13|598[0n]bp|BOLD:AAA3764
Apanteles ensiger|[204]|CNSLQ251-13|546[0n]bp|BOLD:AAA3764
Apanteles ensiger|[205]|CNKJN622-14|564[0n]bp|BOLD:AAA3764
Apanteles ensiger|[206]|CNSLI402-12|631[0n]bp|BOLD:AAA3764
Apanteles ensiger|[207]|CNPPC821-12|630[0n]bp|BOLD:AAA3764
Apanteles ensiger|[208]|CNSLJ175-12|633[0n]bp|BOLD:AAA3764
Apanteles ensiger|[209]|CNPKK1294-14|525[0n]bp|BOLD:AAA3764
Apanteles ensiger|[210]|CNLMP1391-14|555[0n]bp|BOLD:AAA3764
Apanteles ensiger|[211]|CNKJE1961-14|549[0n]bp|BOLD:AAA3764
Apanteles ensiger|[212]|CNLMG1235-14|555[0n]bp|BOLD:AAA3764
Apanteles ensiger|[213]|CNKJF1743-14|591[0n]bp|BOLD:AAA3764
Apanteles ensiger|[214]|SSBAF2145-13|577[0n]bp|BOLD:AAA3764
Apanteles ensiger|[215]|CNPKJ2482-14|582[0n]bp|BOLD:AAA3764
Apanteles ensiger|[216]|CNCHZ1151-09|657[0n]bp|BOLD:AAA3764
Apanteles ensiger|[217]|CNLMG906-14|564[0n]bp|BOLD:AAA3764
Apanteles ensiger|[218]|CNCHZ1146-09|657[0n]bp|BOLD:AAA3764
Apanteles ensiger|[219]|CNCHZ478-09|657[0n]bp|BOLD:AAA3764
Apanteles ensiger|[220]|CNCHX090-09|658[0n]bp|BOLD:AAA3764
Apanteles ensiger|[221]|CNCHZ304-09|657[0n]bp|BOLD:AAA3764
Apanteles ensiger|[222]|CNCHZ1149-09|657[0n]bp|BOLD:AAA3764
Apanteles ensiger|[223]|JSAUG449-11|658[0n]bp|BOLD:AAA3764
Apanteles ensiger|[224]|JBHCI247-11|658[0n]bp|BOLD:AAA3764
Apanteles ensiger|[225]|CNCHZ344-09|657[0n]bp|BOLD:AAA3764
Apanteles ensiger|[226]|ASWAV869-08|654[0n]bp|BOLD:AAA3764
Apanteles ensiger|[227]|CNCHZ264-09|657[0n]bp|BOLD:AAA3764
Apanteles ensiger|[228]|CNCHZ1102-09|657[0n]bp|BOLD:AAA3764
Apanteles ensiger|[229]|CNCHZ1142-09|657[0n]bp|BOLD:AAA3764
Apanteles ensiger|[230]|CNCHZ480-09|657[0n]bp|BOLD:AAA3764
Apanteles ensiger|[231]|JBHCI617-11|658[0n]bp|BOLD:AAA3764
Apanteles ensiger|[232]|CNCHZ476-09|657[0n]bp|BOLD:AAA3764
Apanteles ensiger|[233]|CNCHZ212-09|657[0n]bp|BOLD:AAA3764
Apanteles ensiger|[234]|CNCHZ829-09|657[0n]bp|BOLD:AAA3764
Apanteles ensiger|[235]|CNCHZ1148-09|657[0n]bp|BOLD:AAA3764
Apanteles ensiger|[236]|CNCHZ479-09|657[0n]bp|BOLD:AAA3764
Apanteles ensiger|[237]|CNCHZ199-09|657[0n]bp|BOLD:AAA3764
Apanteles ensiger|[238]|CNCHV264-10|658[0n]bp|BOLD:AAA3764
Apanteles ensiger|[239]|ASWAX959-08|657[0n]bp|BOLD:AAA3764
Apanteles ensiger|[240]|CNCHZ1143-09|657[0n]bp|BOLD:AAA3764
Apanteles ensiger|[241]|BBHYN072-10|658[0n]bp|BOLD:AAA3764
Apanteles ensiger|[242]|ASGLF363-11|658[0n]bp|BOLD:AAA3764
Apanteles ensiger|[243]|BBHYF031-10|658[0n]bp|BOLD:AAA3764
Apanteles ensiger|[244]|CNCHZ1144-09|632[0n]bp|BOLD:AAA3764
Apanteles ensiger|[245]|CNCHW328-09|541[0n]bp|BOLD:AAA3764
Apanteles ensiger|[246]|CNCHZ1147-09|621[1n]bp|BOLD:AAA3764
Apanteles ensiger|[247]|CNSLI401-12|635[0n]bp|BOLD:AAA3764
Apanteles ensiger|[248]|CNSLI089-12|636[0n]bp|BOLD:AAA3764
Apanteles ensiger|[249]|CNSLK104-12|633[0n]bp|BOLD:AAA3764
Apanteles ensiger|[250]|CNSLJ189-12|635[0n]bp|BOLD:AAA3764
Apanteles ensiger|[251]|CNCHZ1141-09|657[0n]bp|BOLD:AAA3764
Apanteles ensiger|[252]|CNCHZ638-09|657[0n]bp|BOLD:AAA3764
Apanteles ensiger|[253]|JSSEP758-11|658[0n]bp|BOLD:AAA3764
Apanteles ensiger|[254]|CNCHZ214-09|657[0n]bp|BOLD:AAA3764
Apanteles ensiger|[255]|BBHYH202-10|649[0n]bp|BOLD:AAA3764
Apanteles ensiger|[256]|DSWAS984-07|384[5n]bp|
Apanteles ensiger|[257]|NCCC037-11|673[1n]bp|BOLD:AAA3764
Apanteles ensiger|[258]|CNCHZ538-09|657[0n]bp|BOLD:AAA3764

Apanteles ensiger|[256]|DSWAS984-07|384[5n]bp|
Apanteles ensiger|[257]|NCCC037-11|673[1n]bp|BOLD:AAA3764
Apanteles ensiger|[258]|CNCHZ538-09|657[0n]bp|BOLD:AAA3764
Apanteles ensiger|[259]|NCCC087-11|673[0n]bp|BOLD:AAA3764
Apanteles ensiger|[260]|BBHYJ345-10|658[0n]bp|BOLD:AAA3764
Apanteles ensiger|[261]|CNCHZ525-09|657[0n]bp|BOLD:AAA3764
Apanteles ensiger|[262]|CNCHZ636-09|657[0n]bp|BOLD:AAA3764
Apanteles ensiger|[263]|BBHYJ346-10|658[0n]bp|BOLD:AAA3764
Apanteles ensiger|[264]|ASWA289-08|657[0n]bp|BOLD:AAA3764
Apanteles ensiger|[265]|CNCHZ1145-09|657[0n]bp|BOLD:AAA3764
Apanteles ensiger|[266]|CNSLH124-12|630[0n]bp|BOLD:AAA3764
Apanteles ensiger|[267]|CNSLJ045-12|621[0n]bp|BOLD:AAA3764
Apanteles ensiger|[268]|CNGBG1411-14|606[0n]bp|BOLD:AAA3764
Apanteles ensiger|[269]|RBINA3743-13|564[0n]bp|BOLD:AAA3764
Apanteles ensiger|[270]|CNROS470-13|591[0n]bp|BOLD:AAA3764
Apanteles ensiger|[271]|CNSLS104-13|607[0n]bp|BOLD:AAA3764
Apanteles ensiger|[272]|CNSLI393-12|631[0n]bp|BOLD:AAA3764
Apanteles ensiger|[273]|CNSLE365-12|631[0n]bp|BOLD:AAA3764
Apanteles ensiger|[274]|ASGLE690-10|658[0n]bp|BOLD:AAA3764
Apanteles ensiger|[275]|CNROJ908-13|597[0n]bp|BOLD:AAA3764
Apanteles ensiger|[276]|CNROS475-13|591[0n]bp|BOLD:AAA3764
Apanteles ensiger|[277]|RBINA3618-13|594[0n]bp|BOLD:AAA3764
Apanteles ensiger|[278]|CNROT251-13|576[0n]bp|BOLD:AAA3764
Apanteles ensiger|[279]|CNGBN1394-14|567[0n]bp|BOLD:AAA3764
Apanteles ensiger|[280]|RBINA3620-13|579[0n]bp|BOLD:AAA3764
Apanteles ensiger|[281]|HYCND1816-11|407[1n]bp|
Apanteles ensiger|[282]|NCCC149-11|673[0n]bp|BOLD:AAA3764
Apanteles ensiger|[283]|CNCHZ215-09|657[0n]bp|BOLD:AAA3764
Apanteles ensiger|[284]|JSAUG1436-11|658[0n]bp|BOLD:AAA3764
Apanteles ensiger|[285]|CNCHZ637-09|657[0n]bp|BOLD:AAA3764
Apanteles ensiger|[286]|JSAUG1440-11|658[0n]bp|BOLD:AAA3764
Apanteles ensiger|[287]|CNCHZ635-09|657[0n]bp|BOLD:AAA3764
Apanteles ensiger|[288]|NCCC130-11|673[0n]bp|BOLD:AAA3764
Apanteles ensiger|[289]|NCCC165-11|673[0n]bp|BOLD:AAA3764
Apanteles ensiger|[290]|CNCHZ1150-09|657[0n]bp|BOLD:AAA3764
Apanteles ensiger|[291]|NCCC249-11|673[0n]bp|BOLD:AAA3764
Apanteles ensiger|[292]|CNROS484-13|600[0n]bp|BOLD:AAA3764
Apanteles ensiger|[293]|CNWBH1210-13|601[0n]bp|BOLD:AAA3764
Apanteles ensiger|[294]|CNROJ688-13|573[0n]bp|BOLD:AAA3764
Apanteles ensiger|[295]|RBINA3615-13|591[0n]bp|BOLD:AAA3764
Apanteles ensiger|[296]|CNROS481-13|606[0n]bp|BOLD:AAA3764
Apanteles ensiger|[297]|NCCC157-11|673[0n]bp|BOLD:AAA3764
Apanteles ensiger|[298]|JSSEP718-11|658[0n]bp|BOLD:AAA3764
Apanteles ensiger|[299]|CNPKO3116-14|594[0n]bp|BOLD:AAA3764
Apanteles ensiger|[300]|CNPKO652-14|588[0n]bp|BOLD:AAA3764
Apanteles ensiger|[301]|CNPKO2903-14|546[0n]bp|BOLD:AAA3764
Apanteles ensiger|[302]|ASCNC020-09|658[0n]bp|BOLD:AAA3764
Apanteles ensiger|[303]|CNCHZ351-09|657[0n]bp|BOLD:AAA3764
Apanteles ensiger|[304]|CNCHZ589-09|657[0n]bp|BOLD:ACE6783
Apanteles ensiger|[305]|CNCHX069-09|658[0n]bp|BOLD:ACE6783
Apanteles ensiger|[306]|CNCHX094-09|658[0n]bp|BOLD:ACE6783
Apanteles ensiger|[307]|CNCHX091-09|658[0n]bp|BOLD:ACE6783
Apanteles ensiger|[308]|CNCHX068-09|658[0n]bp|BOLD:ACE6783
Apanteles ensiger|[309]|CNCHZ680-09|657[0n]bp|BOLD:ACE6783
Apanteles ensiger|[310]|CNCHX071-09|658[0n]bp|BOLD:ACE6783
Apanteles ensiger|[311]|WOMIA340-11|658[0n]bp|BOLD:ACE6783
Apanteles ensiger|[312]|CNCHX070-09|658[0n]bp|BOLD:ACE6783
Apanteles ensiger|[313]|CNCHZ1095-09|657[0n]bp|BOLD:ACE6783
Apanteles ensiger|[314]|CNCHX093-09|658[0n]bp|BOLD:ACE6783
Apanteles ensiger|[315]|CNCHZ685-09|657[0n]bp|BOLD:ACE6783
Apanteles ensiger|[316]|CNCHX072-09|637[0n]bp|BOLD:ACE6783
Apanteles ensiger|[317]|CNCHX092-09|623[0n]bp|BOLD:ACE6783
Apanteles ensiger|[318]|MBIOL310-14|590[0n]bp|BOLD:ACE6783
Apanteles ensiger|[319]|SMTPD4980-13|597[0n]bp|BOLD:ACE6783
Apanteles ensiger|[320]|MBION508-14|558[0n]bp|BOLD:ACE6783
Apanteles ensiger|[321]|CNCHZ252-09|657[0n]bp|BOLD:ACE6783
Apanteles ensiger|[322]|CNCHX1040-09|639[0n]bp|BOLD:ACE6783
Apanteles ensiger|[323]|CNCHZ203-09|657[0n]bp|BOLD:ACE6783
Apanteles ensiger|[324]|CNCHZ353-09|657[0n]bp|BOLD:ACE6783
Apanteles ensiger|[325]|CNCHZ336-09|657[0n]bp|BOLD:ACE6783
Apanteles ensiger|[326]|SMTPD4929-13|549[0n]bp|BOLD:ACE6783
Apanteles ensiger|[327]|CNPPJ1314-12|646[0n]bp|BOLD:ACE6783
Apanteles ensiger|[328]|CNCHZ356-09|657[0n]bp|BOLD:ACE6783
Apanteles ensiger|[329]|CNCHZ531-09|657[0n]bp|BOLD:ACE6783
Apanteles ensiger|[330]|CNCHZ244-09|657[0n]bp|BOLD:ACE6783
Apanteles ensiger|[331]|CNCHZ341-09|657[0n]bp|BOLD:ACE6783
Apanteles ensiger|[332]|CNCHZ293-09|657[0n]bp|BOLD:ACE6783
Apanteles ensiger|[333]|CNCHZ542-09|657[0n]bp|BOLD:ACE6783
Apanteles ensiger|[334]|MBIOE1410-13|609[0n]bp|BOLD:ACE6783
Apanteles ensiger|[335]|CNCHZ198-09|657[0n]bp|BOLD:ACE6783
Apanteles ensiger|[336]|CNGBJ1514-14|597[0n]bp|BOLD:ACE6783
Apanteles ensiger|[337]|NCCC726-11|673[0n]bp|BOLD:ACE6783
Apanteles ensiger|[338]|SMTPD1793-13|567[0n]bp|BOLD:ACE6783
Apanteles ensiger|[339]|CNCHZ535-09|657[0n]bp|BOLD:ACE6783
Apanteles ensiger|[340]|TDWGB052-10|658[0n]bp|BOLD:ACE6783
Apanteles ensiger|[341]|CNCHZ291-09|657[0n]bp|BOLD:ACE6783
Apanteles ensiger|[342]|CNCHZ289-09|657[0n]bp|BOLD:ACE6783
Apanteles ensiger|[343]|CNCHZ537-09|657[0n]bp|BOLD:ACE6783
Apanteles ensiger|[344]|CNCHZ546-09|657[0n]bp|BOLD:ACE6783

Apanteles ensiger|[342]|CNCHZ289-09|657[0n]bp|BOLD:ACE6783
Apanteles ensiger|[343]|CNCHZ537-09|657[0n]bp|BOLD:ACE6783
Apanteles ensiger|[344]|CNCHZ546-09|657[0n]bp|BOLD:ACE6783
Apanteles ensiger|[345]|TDWGB248-10|658[0n]bp|BOLD:ACE6783
Apanteles ensiger|[346]|CNCHZ262-09|657[0n]bp|BOLD:ACE6783
Apanteles ensiger|[347]|MBIOE1290-13|585[0n]bp|BOLD:ACE6783
Apanteles ensiger|[348]|SMTPD3273-13|606[0n]bp|BOLD:ACE6783
Apanteles ensiger|[349]|MBIOJ817-13|576[0n]bp|BOLD:ACE6783
Apanteles ensiger|[350]|SMTPD3941-13|591[0n]bp|BOLD:ACE6783
Apanteles ensiger|[351]|SMTPD3789-13|603[0n]bp|BOLD:ACE6783
Apanteles ensiger|[352]|SMTPD5253-13|603[0n]bp|BOLD:ACE6783
Apanteles ensiger|[353]|MBIOI1284-13|600[0n]bp|BOLD:ACE6783
Apanteles ensiger|[354]|MBIOL366-14|588[0n]bp|BOLD:ACE6783
Apanteles ensiger|[355]|MBION363-14|585[0n]bp|BOLD:ACE6783
Apanteles ensiger|[356]|MBION341-14|572[0n]bp|BOLD:ACE6783
Apanteles ensiger|[357]|MBIOL370-14|580[0n]bp|BOLD:ACE6783
Apanteles ensiger|[358]|SMTPD1460-13|567[0n]bp|BOLD:ACE6783
Apanteles ensiger|[359]|MBIOE2286-13|581[0n]bp|BOLD:ACE6783
Apanteles ensiger|[360]|SMTPD3831-13|576[0n]bp|BOLD:ACE6783
Apanteles ensiger|[361]|MBIOK461-14|577[0n]bp|BOLD:ACE6783
Apanteles ensiger|[362]|SMTPD4964-13|561[0n]bp|BOLD:ACE6783
Apanteles ensiger|[363]|SMTPD5202-13|570[0n]bp|BOLD:ACE6783
Apanteles ensiger|[364]|SMTPD3239-13|567[0n]bp|BOLD:ACE6783
Apanteles ensiger|[365]|SMTPD3777-13|567[0n]bp|BOLD:ACE6783
Apanteles ensiger|[366]|SMTPD4966-13|567[0n]bp|BOLD:ACE6783
Apanteles ensiger|[367]|MBION389-14|547[0n]bp|BOLD:ACE6783
Apanteles ensiger|[368]|MBIOD945-13|602[0n]bp|BOLD:ACE6783
Apanteles ensiger|[369]|SMTPD4869-13|567[0n]bp|BOLD:ACE6783
Apanteles ensiger|[370]|MBIOK431-14|559[0n]bp|BOLD:ACE6783
Apanteles ensiger|[371]|CNCHX802-09|658[0n]bp|BOLD:ACE6783
Apanteles ensiger|[372]|CNCHZ541-09|657[0n]bp|BOLD:ACE6783
Apanteles ensiger|[373]|CNCHZ603-09|657[0n]bp|BOLD:ACE6783
Apanteles ensiger|[374]|CNCHZ539-09|657[0n]bp|BOLD:ACE6783
Apanteles ensiger|[375]|CNCHZ579-09|657[0n]bp|BOLD:ACE6783
Apanteles ensiger|[376]|ASWA314-08|657[0n]bp|BOLD:ACE6783
Apanteles ensiger|[377]|CNCHZ550-09|657[0n]bp|BOLD:ACE6783
Apanteles ensiger|[378]|ASWA309-08|657[0n]bp|BOLD:ACE6783
Apanteles ensiger|[379]|CNCHZ305-09|657[0n]bp|BOLD:ACE6783
Apanteles ensiger|[380]|CNCHZ197-09|657[0n]bp|BOLD:ACE6783
Apanteles ensiger|[381]|ASWA305-08|657[0n]bp|BOLD:ACE6783
Apanteles ensiger|[382]|CNCHZ201-09|657[0n]bp|BOLD:ACE6783
Apanteles ensiger|[383]|CNCHZ296-09|657[0n]bp|BOLD:ACE6783
Apanteles ensiger|[384]|CNCHZ229-09|657[0n]bp|BOLD:ACE6783
Apanteles ensiger|[385]|ASWA311-08|657[0n]bp|BOLD:ACE6783
Apanteles ensiger|[386]|ASWA306-08|657[0n]bp|BOLD:ACE6783
Apanteles ensiger|[387]|CNCHZ540-09|657[0n]bp|BOLD:ACE6783
Apanteles ensiger|[388]|ASWA308-08|657[0n]bp|BOLD:ACE6783
Apanteles ensiger|[389]|ASWA290-08|657[0n]bp|BOLD:ACE6783
Apanteles ensiger|[390]|CNCHZ266-09|657[0n]bp|BOLD:ACE6783
Apanteles ensiger|[391]|ASWA313-08|657[0n]bp|BOLD:ACE6783
Apanteles ensiger|[392]|CNCHZ310-09|657[0n]bp|BOLD:ACE6783
Apanteles ensiger|[393]|CNCHZ530-09|657[0n]bp|BOLD:ACE6783
Apanteles ensiger|[394]|CNCHZ536-09|657[0n]bp|BOLD:ACE6783
Apanteles ensiger|[395]|CNCHZ534-09|657[0n]bp|BOLD:ACE6783
Apanteles ensiger|[396]|CNCHZ303-09|657[0n]bp|BOLD:ACE6783
Apanteles ensiger|[397]|ASWA310-08|657[0n]bp|BOLD:ACE6783
Apanteles ensiger|[398]|ASWA307-08|657[0n]bp|BOLD:ACE6783
Apanteles ensiger|[399]|ASWA312-08|657[0n]bp|BOLD:ACE6783
Apanteles ensiger|[400]|CNCHZ599-09|657[1n]bp|BOLD:ACE6783
Apanteles ensiger|[401]|CNCHZ858-09|657[0n]bp|BOLD:ACE6783
Apanteles ensiger|[402]|MBION369-14|587[0n]bp|BOLD:ACE6783
Apanteles ensiger|[403]|SMTPD3828-13|561[0n]bp|BOLD:ACE6783
Apanteles ensiger|[404]|SMTPD5208-13|609[0n]bp|BOLD:ACE6783
Apanteles ensiger|[405]|MBIOI1222-13|609[0n]bp|BOLD:ACE6783
Apanteles ensiger|[406]|MBIOK076-14|597[0n]bp|BOLD:ACE6783
Apanteles ensiger|[407]|MBIOH323-13|617[0n]bp|BOLD:ACE6783
Apanteles ensiger|[408]|CNPPC885-12|630[0n]bp|BOLD:ACE6783
Apanteles ensiger|[409]|SMTPD3455-13|543[0n]bp|BOLD:ACE6783
Apanteles ensiger|[410]|CNCHZ532-09|657[1n]bp|BOLD:ACE6783
Apanteles ensiger|[411]|CNCHZ600-09|657[0n]bp|BOLD:ACE6783
Apanteles conanchetorum|[412]|CNFNF871-14|525[0n]bp|BOLD:AAC5506
Apanteles conanchetorum|[413]|CNFNG557-14|530[0n]bp|BOLD:AAC5506
Apanteles conanchetorum|[414]|CNWLF652-12|658[0n]bp|BOLD:AAC5507
Apanteles conanchetorum|[415]|CNWLF272-12|658[0n]bp|BOLD:AAC5507
Apanteles conanchetorum|[416]|CNWLF646-12|658[0n]bp|BOLD:AAC5507
Apanteles conanchetorum|[417]|CNWLM287-13|597[0n]bp|BOLD:AAC5507
Apanteles conanchetorum|[418]|CNWLM799-13|674[0n]bp|BOLD:AAC5507
Apanteles conanchetorum|[419]|CNWLN171-13|577[0n]bp|BOLD:AAC5507
Apanteles conanchetorum|[420]|CNWLF656-12|627[0n]bp|BOLD:AAC5507
Apanteles conanchetorum|[421]|CNWLF631-12|631[0n]bp|BOLD:AAC5507
Apanteles conanchetorum|[422]|CNPEP1205-14|603[0n]bp|BOLD:AAC5507
Apanteles conanchetorum|[423]|CNWLM1411-13|622[0n]bp|BOLD:AAC5507
Apanteles conanchetorum|[424]|SSPAC3799-13|616[0n]bp|BOLD:AAC5507
Apanteles conanchetorum|[425]|CNWLM309-13|603[0n]bp|BOLD:AAC5507
Apanteles conanchetorum|[426]|SSPAC13669-13|600[0n]bp|BOLD:AAC5507
Apanteles conanchetorum|[427]|BBHYK285-10|658[0n]bp|BOLD:AAC5507
Apanteles conanchetorum|[428]|CNCHZ286-09|657[0n]bp|BOLD:AAC5507
Apanteles conanchetorum|[429]|CNFNF286-14|543[0n]bp|BOLD:AAC5506
Apanteles conanchetorum|[430]|CNFNB2706-14|519[0n]bp|BOLD:AAC5506

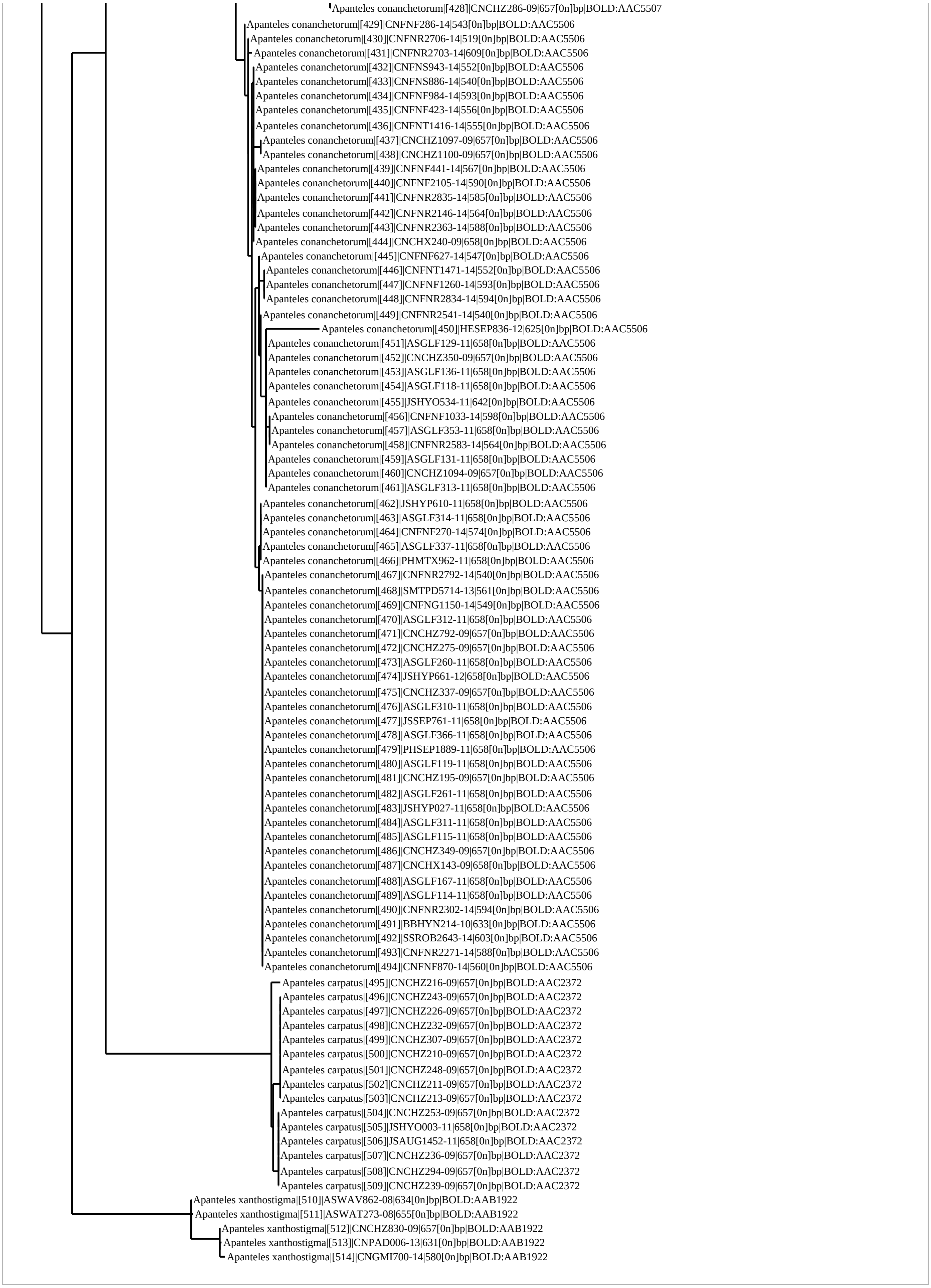
Apanteles conanchetorum|[428]|CNCHZ286-09|657[0n]bp|BOLD:AAC5507
Apanteles conanchetorum|[429]|CNFNF286-14|543[0n]bp|BOLD:AAC5506
Apanteles conanchetorum|[430]|CNFNR2706-14|519[0n]bp|BOLD:AAC5506
Apanteles conanchetorum|[431]|CNFNR2703-14|609[0n]bp|BOLD:AAC5506
Apanteles conanchetorum|[432]|CNFNS943-14|552[0n]bp|BOLD:AAC5506
Apanteles conanchetorum|[433]|CNFNS886-14|540[0n]bp|BOLD:AAC5506
Apanteles conanchetorum|[434]|CNFNF984-14|593[0n]bp|BOLD:AAC5506
Apanteles conanchetorum|[435]|CNFNF423-14|556[0n]bp|BOLD:AAC5506
Apanteles conanchetorum|[436]|CNFNT1416-14|555[0n]bp|BOLD:AAC5506
Apanteles conanchetorum|[437]|CNCHZ1097-09|657[0n]bp|BOLD:AAC5506
Apanteles conanchetorum|[438]|CNCHZ1100-09|657[0n]bp|BOLD:AAC5506
Apanteles conanchetorum|[439]|CNFNF441-14|567[0n]bp|BOLD:AAC5506
Apanteles conanchetorum|[440]|CNFNF2105-14|590[0n]bp|BOLD:AAC5506
Apanteles conanchetorum|[441]|CNFNR2835-14|585[0n]bp|BOLD:AAC5506
Apanteles conanchetorum|[442]|CNFNR2146-14|564[0n]bp|BOLD:AAC5506
Apanteles conanchetorum|[443]|CNFNR2363-14|588[0n]bp|BOLD:AAC5506
Apanteles conanchetorum|[444]|CNCHX240-09|658[0n]bp|BOLD:AAC5506
Apanteles conanchetorum|[445]|CNFNF627-14|547[0n]bp|BOLD:AAC5506
Apanteles conanchetorum|[446]|CNFNT1471-14|552[0n]bp|BOLD:AAC5506
Apanteles conanchetorum|[447]|CNFNF1260-14|593[0n]bp|BOLD:AAC5506
Apanteles conanchetorum|[448]|CNFNR2834-14|594[0n]bp|BOLD:AAC5506
Apanteles conanchetorum|[449]|CNFNR2541-14|540[0n]bp|BOLD:AAC5506
Apanteles conanchetorum|[450]|HESEP836-12|625[0n]bp|BOLD:AAC5506
Apanteles conanchetorum|[451]|ASGLF129-11|658[0n]bp|BOLD:AAC5506
Apanteles conanchetorum|[452]|CNCHZ350-09|657[0n]bp|BOLD:AAC5506
Apanteles conanchetorum|[453]|ASGLF136-11|658[0n]bp|BOLD:AAC5506
Apanteles conanchetorum|[454]|ASGLF118-11|658[0n]bp|BOLD:AAC5506
Apanteles conanchetorum|[455]|JSHYO534-11|642[0n]bp|BOLD:AAC5506
Apanteles conanchetorum|[456]|CNFNF1033-14|598[0n]bp|BOLD:AAC5506
Apanteles conanchetorum|[457]|ASGLF353-11|658[0n]bp|BOLD:AAC5506
Apanteles conanchetorum|[458]|CNFNR2583-14|564[0n]bp|BOLD:AAC5506
Apanteles conanchetorum|[459]|ASGLF131-11|658[0n]bp|BOLD:AAC5506
Apanteles conanchetorum|[460]|CNCHZ1094-09|657[0n]bp|BOLD:AAC5506
Apanteles conanchetorum|[461]|ASGLF313-11|658[0n]bp|BOLD:AAC5506
Apanteles conanchetorum|[462]|JSHYP610-11|658[0n]bp|BOLD:AAC5506
Apanteles conanchetorum|[463]|ASGLF314-11|658[0n]bp|BOLD:AAC5506
Apanteles conanchetorum|[464]|CNFNF270-14|574[0n]bp|BOLD:AAC5506
Apanteles conanchetorum|[465]|ASGLF337-11|658[0n]bp|BOLD:AAC5506
Apanteles conanchetorum|[466]|PHMTX962-11|658[0n]bp|BOLD:AAC5506
Apanteles conanchetorum|[467]|CNFNR2792-14|540[0n]bp|BOLD:AAC5506
Apanteles conanchetorum|[468]|SMTPD5714-13|561[0n]bp|BOLD:AAC5506
Apanteles conanchetorum|[469]|CNFNG1150-14|549[0n]bp|BOLD:AAC5506
Apanteles conanchetorum|[470]|ASGLF312-11|658[0n]bp|BOLD:AAC5506
Apanteles conanchetorum|[471]|CNCHZ792-09|657[0n]bp|BOLD:AAC5506
Apanteles conanchetorum|[472]|CNCHZ275-09|657[0n]bp|BOLD:AAC5506
Apanteles conanchetorum|[473]|ASGLF260-11|658[0n]bp|BOLD:AAC5506
Apanteles conanchetorum|[474]|JSHYP661-12|658[0n]bp|BOLD:AAC5506
Apanteles conanchetorum|[475]|CNCHZ337-09|657[0n]bp|BOLD:AAC5506
Apanteles conanchetorum|[476]|ASGLF310-11|658[0n]bp|BOLD:AAC5506
Apanteles conanchetorum|[477]|JSSEP761-11|658[0n]bp|BOLD:AAC5506
Apanteles conanchetorum|[478]|ASGLF366-11|658[0n]bp|BOLD:AAC5506
Apanteles conanchetorum|[479]|PHSEP1889-11|658[0n]bp|BOLD:AAC5506
Apanteles conanchetorum|[480]|ASGLF119-11|658[0n]bp|BOLD:AAC5506
Apanteles conanchetorum|[481]|CNCHZ195-09|657[0n]bp|BOLD:AAC5506
Apanteles conanchetorum|[482]|ASGLF261-11|658[0n]bp|BOLD:AAC5506
Apanteles conanchetorum|[483]|JSHYP027-11|658[0n]bp|BOLD:AAC5506
Apanteles conanchetorum|[484]|ASGLF311-11|658[0n]bp|BOLD:AAC5506
Apanteles conanchetorum|[485]|ASGLF115-11|658[0n]bp|BOLD:AAC5506
Apanteles conanchetorum|[486]|CNCHZ349-09|657[0n]bp|BOLD:AAC5506
Apanteles conanchetorum|[487]|CNCHX143-09|658[0n]bp|BOLD:AAC5506
Apanteles conanchetorum|[488]|ASGLF167-11|658[0n]bp|BOLD:AAC5506
Apanteles conanchetorum|[489]|ASGLF114-11|658[0n]bp|BOLD:AAC5506
Apanteles conanchetorum|[490]|CNFNR2302-14|594[0n]bp|BOLD:AAC5506
Apanteles conanchetorum|[491]|BBHYN214-10|633[0n]bp|BOLD:AAC5506
Apanteles conanchetorum|[492]|SSROB2643-14|603[0n]bp|BOLD:AAC5506
Apanteles conanchetorum|[493]|CNFNR2271-14|588[0n]bp|BOLD:AAC5506
Apanteles conanchetorum|[494]|CNFNF870-14|560[0n]bp|BOLD:AAC5506
Apanteles carpatus|[495]|CNCHZ216-09|657[0n]bp|BOLD:AAC2372
Apanteles carpatus|[496]|CNCHZ243-09|657[0n]bp|BOLD:AAC2372
Apanteles carpatus|[497]|CNCHZ226-09|657[0n]bp|BOLD:AAC2372
Apanteles carpatus|[498]|CNCHZ232-09|657[0n]bp|BOLD:AAC2372
Apanteles carpatus|[499]|CNCHZ307-09|657[0n]bp|BOLD:AAC2372
Apanteles carpatus|[500]|CNCHZ210-09|657[0n]bp|BOLD:AAC2372
Apanteles carpatus|[501]|CNCHZ248-09|657[0n]bp|BOLD:AAC2372
Apanteles carpatus|[502]|CNCHZ211-09|657[0n]bp|BOLD:AAC2372
Apanteles carpatus|[503]|CNCHZ213-09|657[0n]bp|BOLD:AAC2372
Apanteles carpatus|[504]|CNCHZ253-09|657[0n]bp|BOLD:AAC2372
Apanteles carpatus|[505]|JSHYO003-11|658[0n]bp|BOLD:AAC2372
Apanteles carpatus|[506]|JSAUG1452-11|658[0n]bp|BOLD:AAC2372
Apanteles carpatus|[507]|CNCHZ236-09|657[0n]bp|BOLD:AAC2372
Apanteles carpatus|[508]|CNCHZ294-09|657[0n]bp|BOLD:AAC2372
Apanteles carpatus|[509]|CNCHZ239-09|657[0n]bp|BOLD:AAC2372
Apanteles xanthostigma|[510]|ASWAV862-08|634[0n]bp|BOLD:AAB1922
Apanteles xanthostigma|[511]|ASWAT273-08|655[0n]bp|BOLD:AAB1922
Apanteles xanthostigma|[512]|CNCHZ830-09|657[0n]bp|BOLD:AAB1922
Apanteles xanthostigma|[513]|CNPAD006-13|631[0n]bp|BOLD:AAB1922
Apanteles xanthostigma|[514]|CNGMI700-14|580[0n]bp|BOLD:AAB1922
