# Supplementary figures and images for: Streamlining the use of BOLD specimen data to record species distributions: a case study with ten Nearctic species of Microgastrinae (Hymenoptera: Braconidae)
Source: Biodivers Data J. 2014 Oct 29;(2):e4153. doi: 10.3897/BDJ.2.e4153 (PMC4251541; doi:10.3897/BDJ.2.e4153)

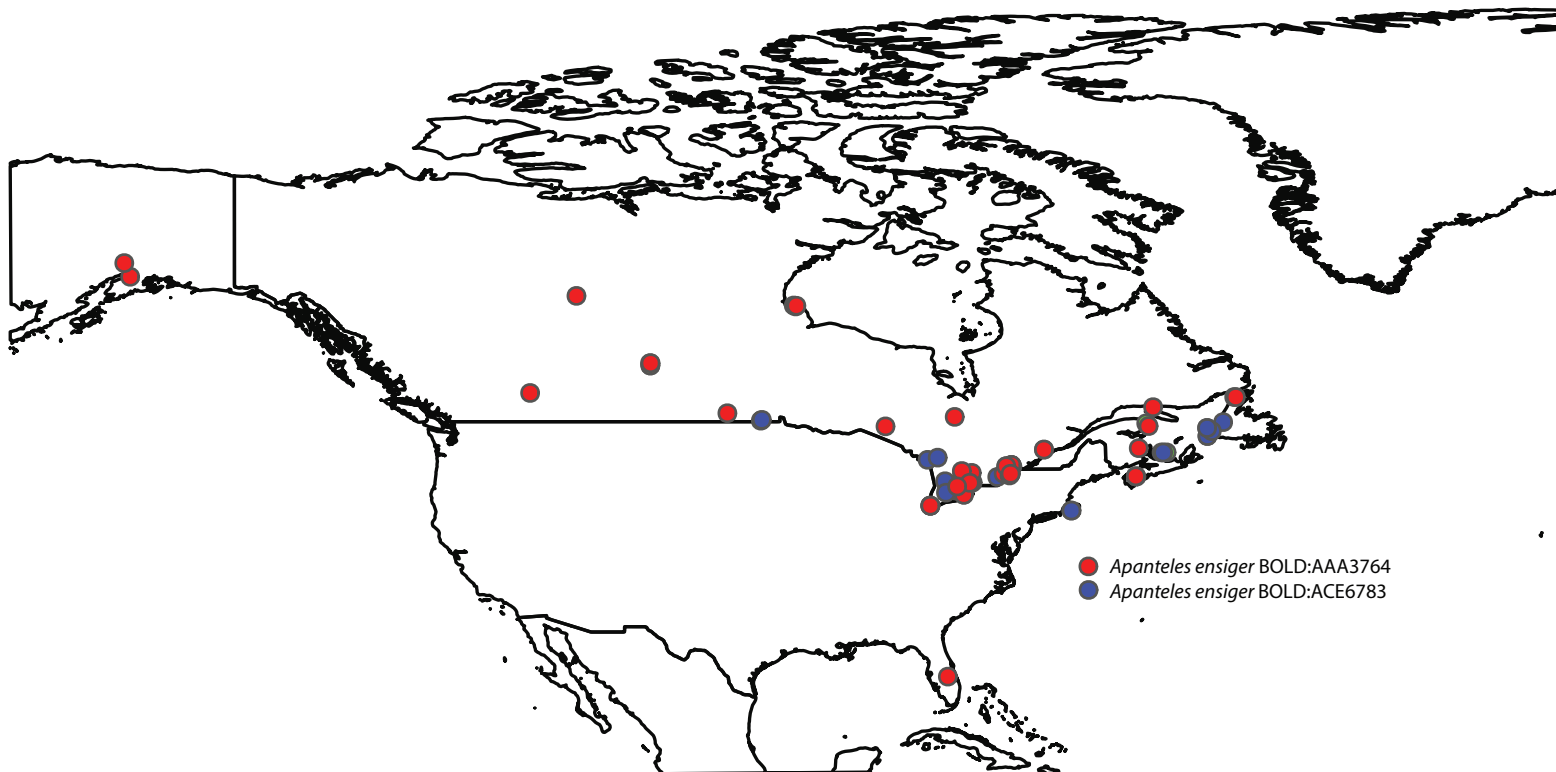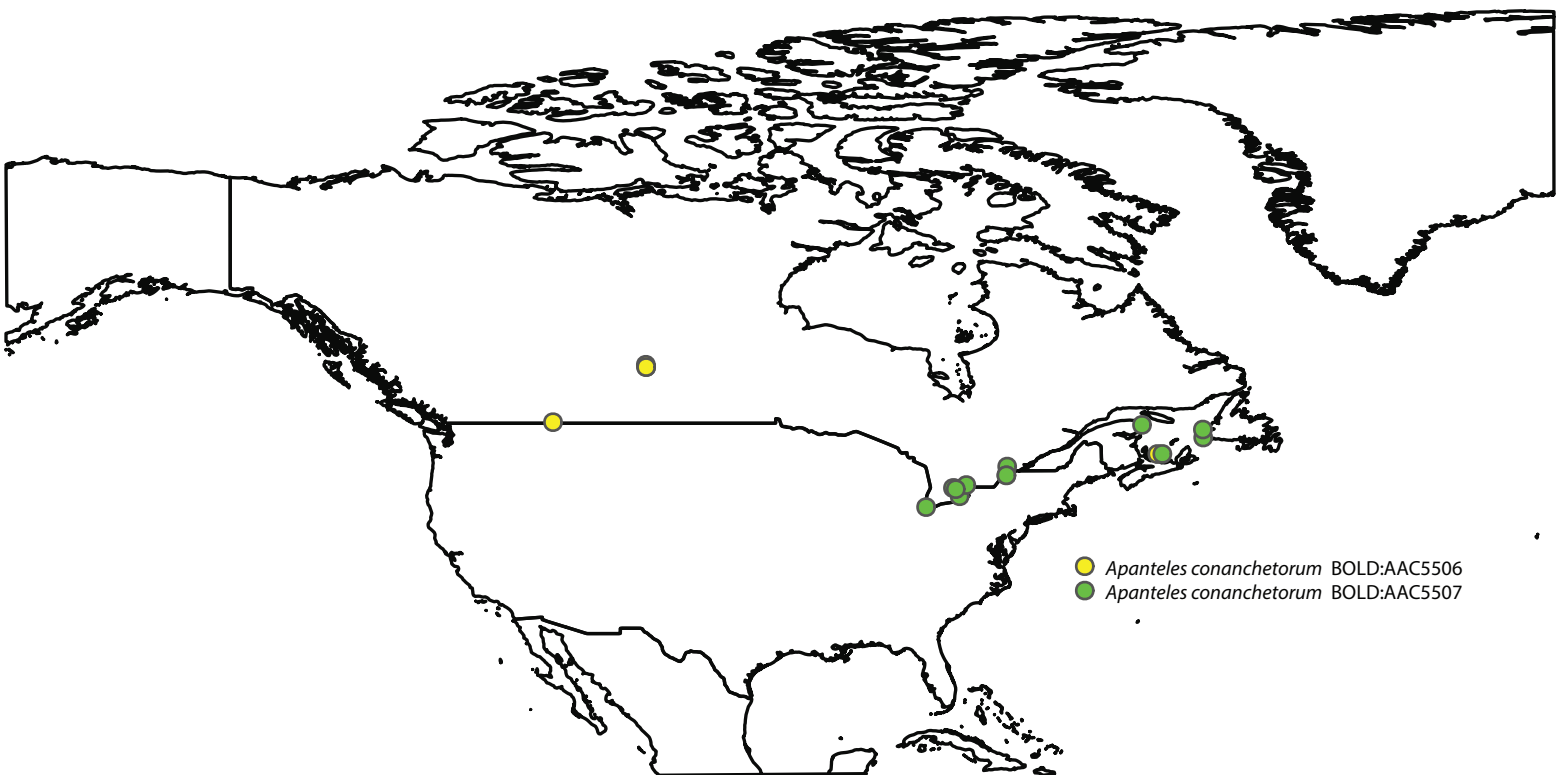

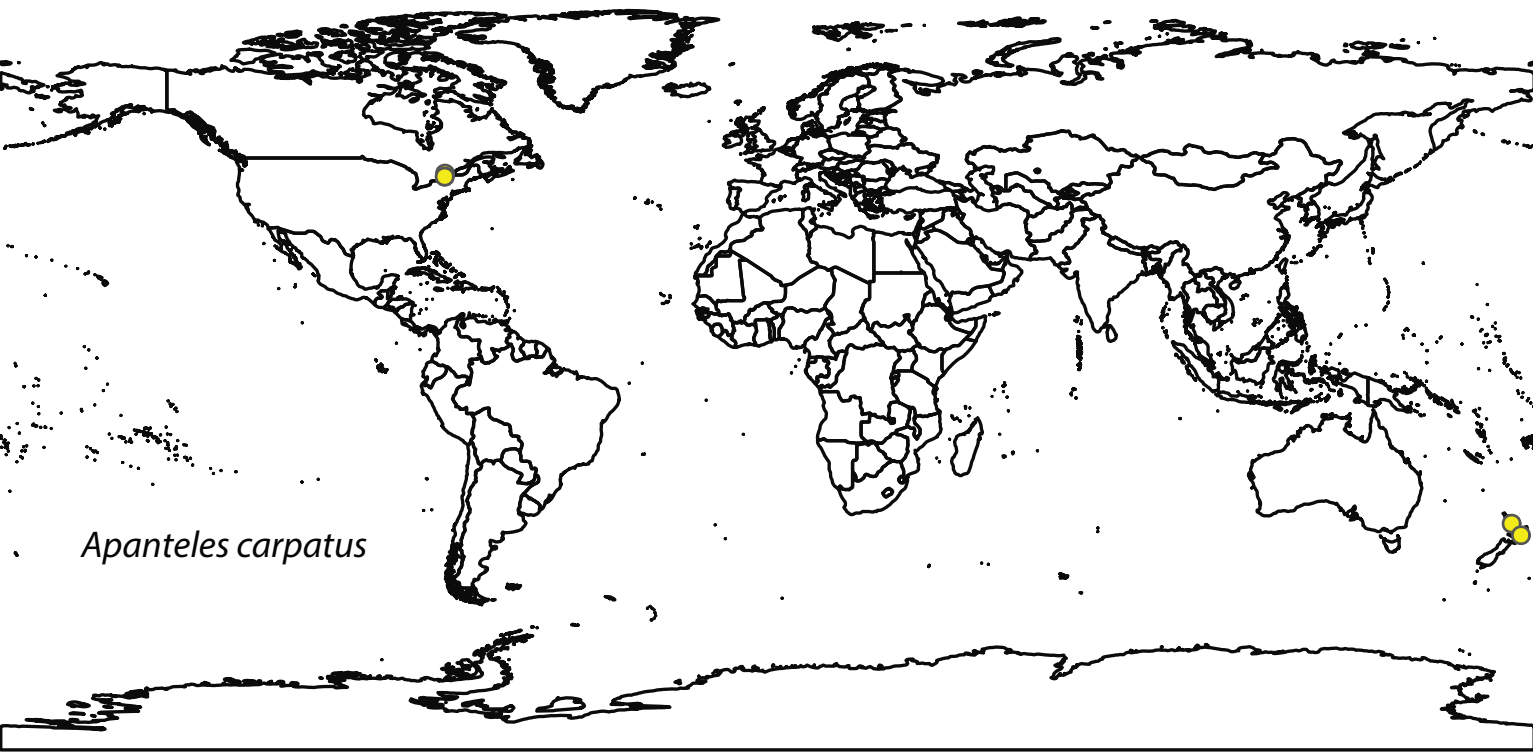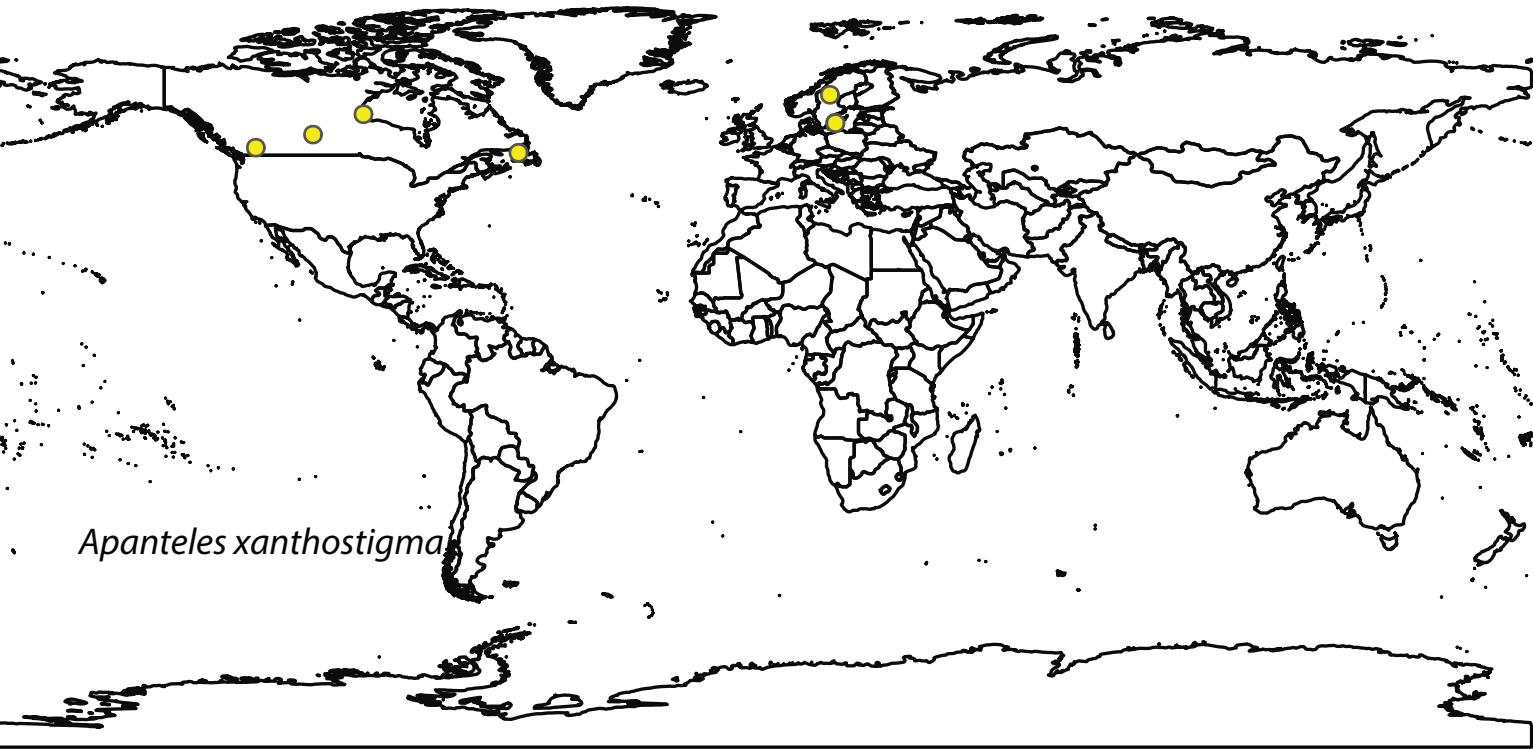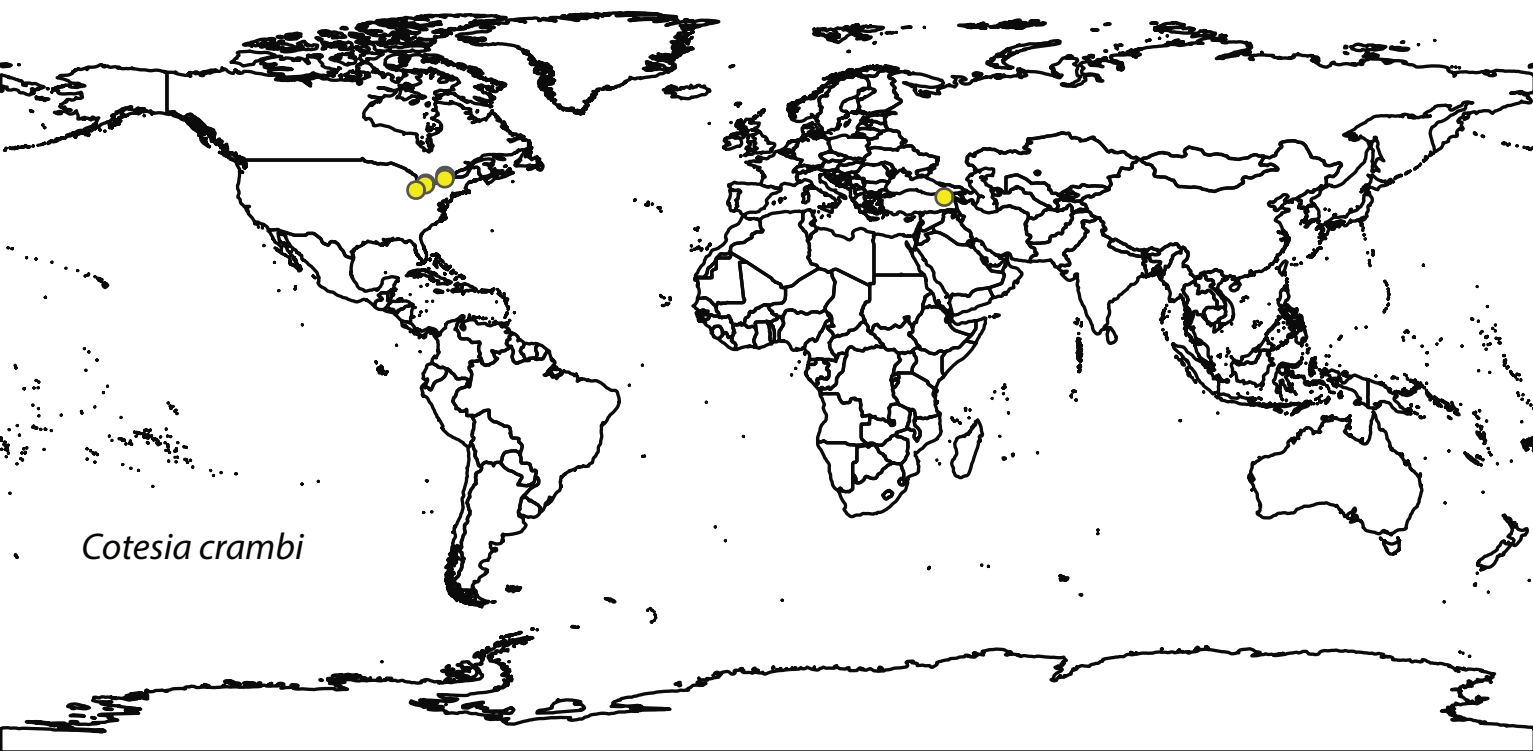

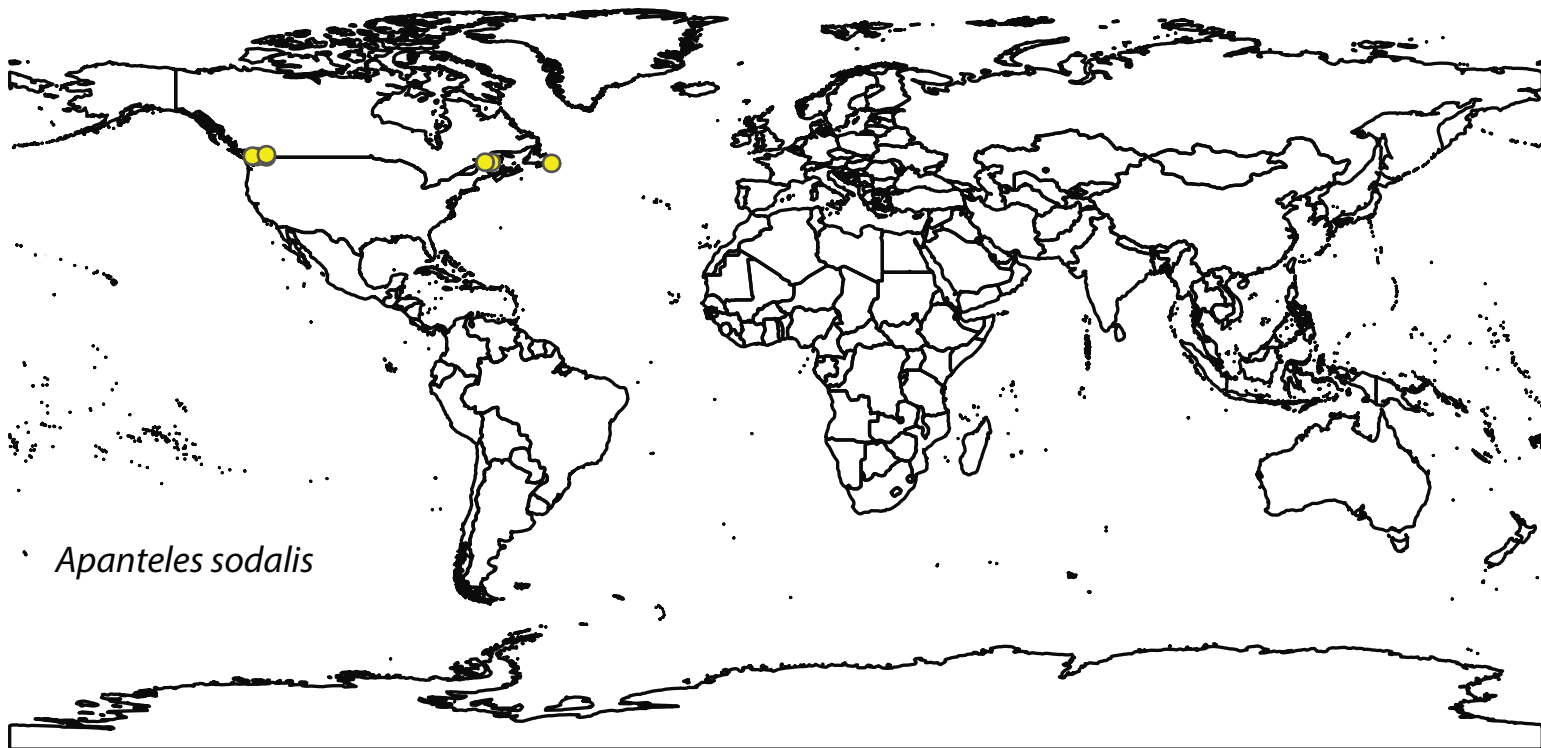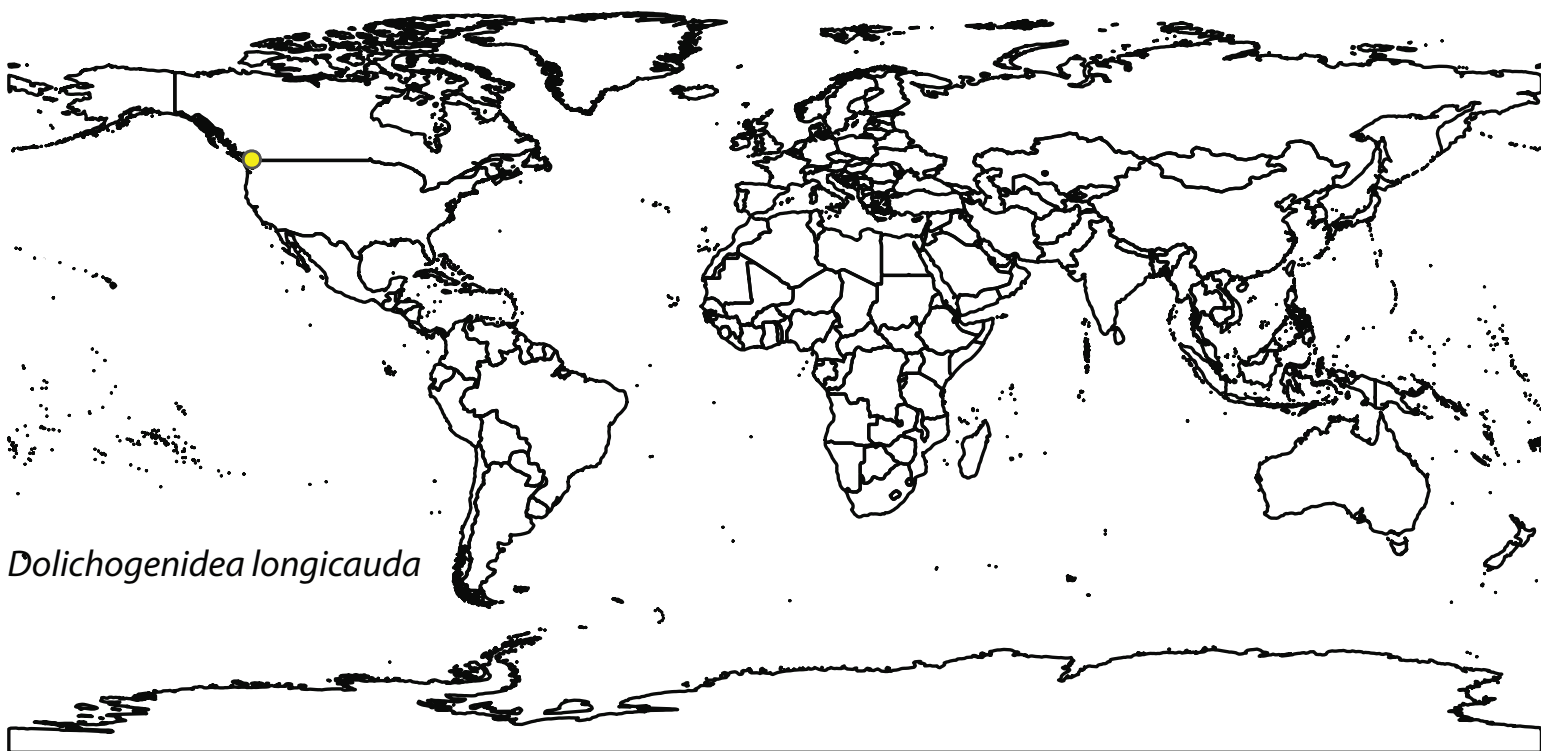

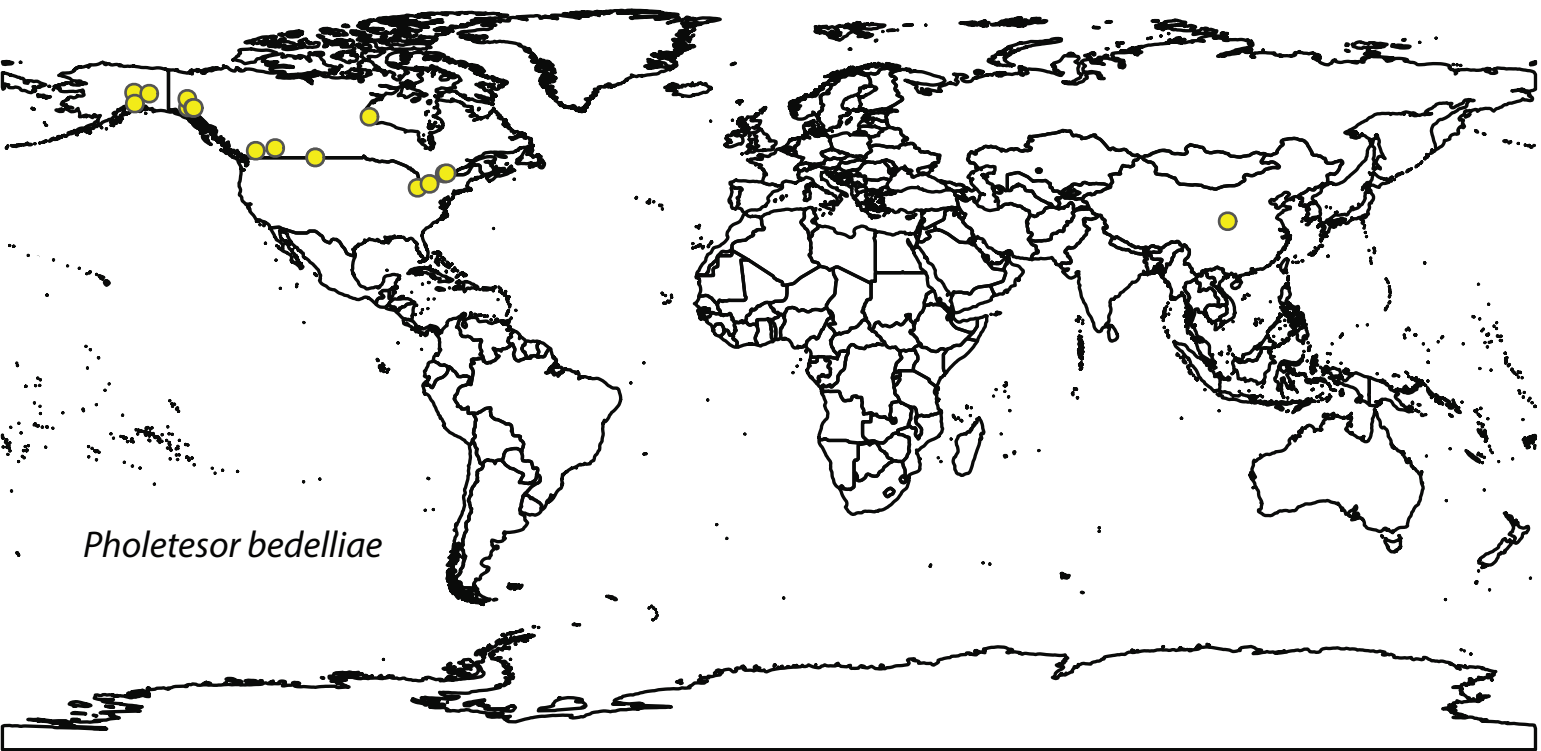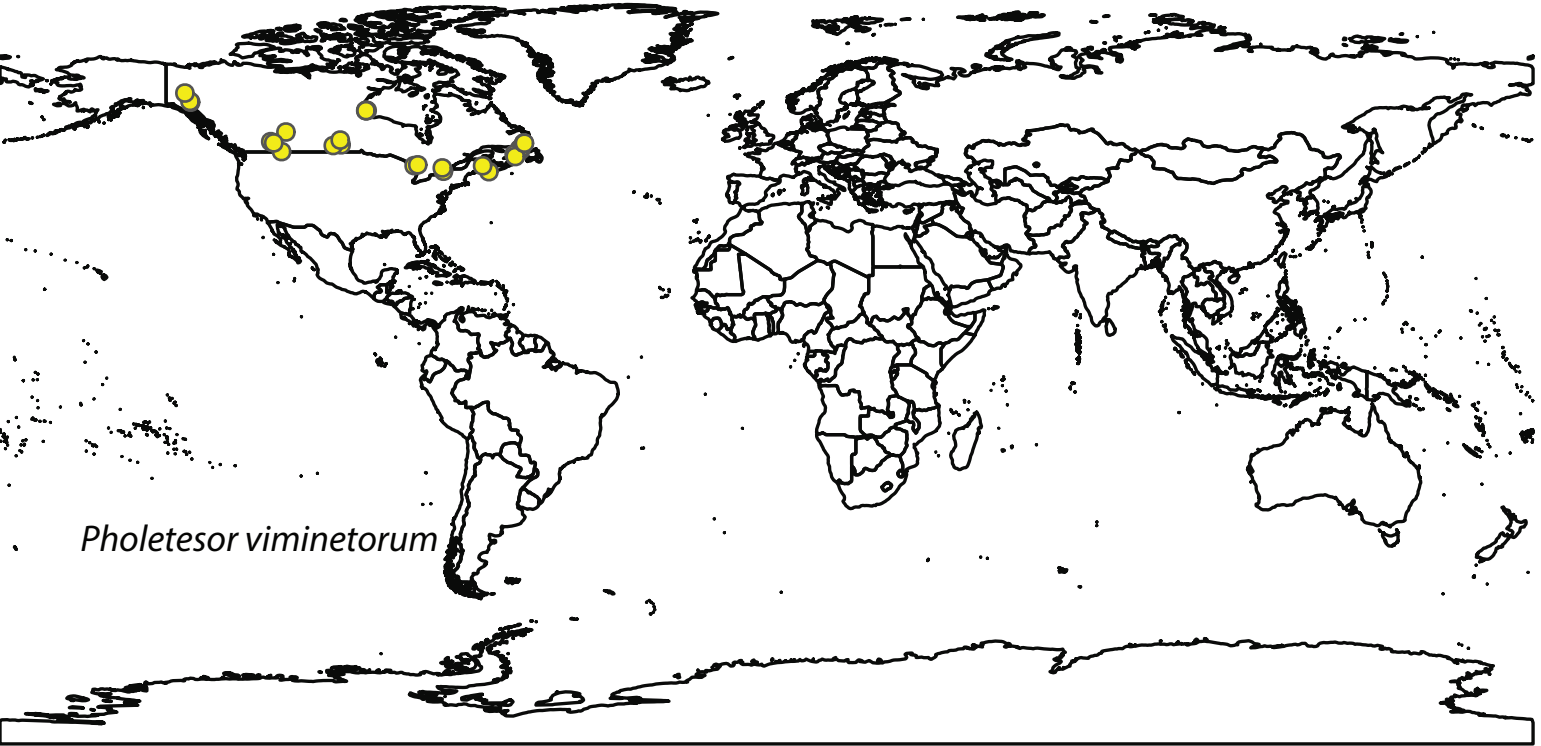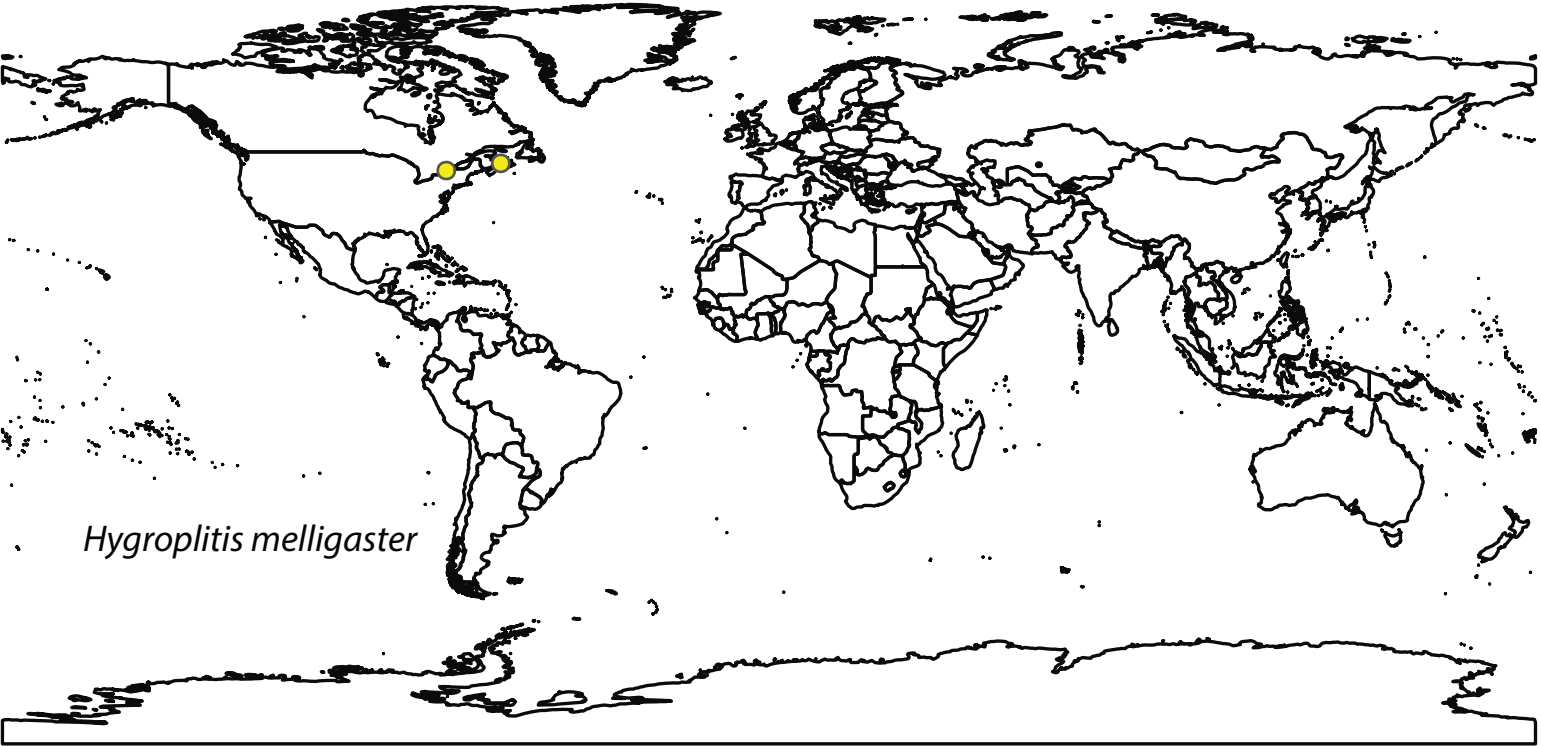

Supplement: Supplementary material 5 — Distribution of 10 Microgastrinae species based on BOLD records [file biodiversity_data_journal-2-e4153-s005.pdf]
